# Exploring the utility of a NGS multigene panel to predict BCG response in patients with non-muscle invasive bladder cancer

**DOI:** 10.32604/or.2024.056282

**Published:** 2025-02-28

**Authors:** BELARDINILLI FRANCESCA, MICHELA DE MEO, FRANCESCO DEL GIUDICE, CARLO MARIA SCORNAJENGHI, PAOLA GAZZANIGA, ETTORE DE BERARDINIS, LUCA MARINO, FABIO MASSIMO MAGLIOCCA, BENJAMIN INBEH CHUNG, JAN ŁASZKIEWICZ, VALENTINA MAGRI, GIUSEPPE GIANNINI, CHIARA NICOLAZZO

**Affiliations:** 1 Department of Molecular Medicine, Sapienza University of Rome, Rome, 00161, Italy; 2 Department of Maternal-Child and Urological Sciences, Sapienza University of Rome, Rome, 00161, Italy; 3 Department of Mechanical and Aerospace Engineering, Sapienza University of Rome, Rome, 00161, Italy; 4 Department of Radiological, Oncological and Pathological Sciences, Sapienza University of Rome, Rome, 00161, Italy; 5 Department of Urology, Stanford University School of Medicine, Stanford, CA 94305, USA; 6 University Center of Excellence in Urology, Wrocław Medical University, Wrocław, 50556, Poland; 7 Istituto Pasteur-Fondazione Cenci Bolognetti, Rome, 00161, Italy

**Keywords:** Non-muscle invasive bladder cancer (NMIBC), Bacillus Calmette-Guérin (BCG), Macropinocytosis, Molecular profile, Next-generation sequencing (NGS)

## Abstract

**Objectives:**

Intravesical Bacillus Calmette-Guérin (BCG) therapy is a gold standard for patients with high-risk non-muscle invasive bladder cancer (NMIBC). Although a long-lasting therapeutic response is observed in most patients, BCG failure occurs in 30%–50% of patients and a progression to muscle-invasive disease is found in 10%–15%. Therefore, predicting high-risk patients who might not benefit from BCG treatment is critical. The purpose of this study was to identify, whether the presence of specific oncogenic mutations might be indicative of BCG treatment response.

**Methods:**

Nineteen high-grade NMIBC patients who received intravesical BCG were retrospectively enrolled and divided into “responders” and “non-responders” groups. Tissue samples from transurethral resection of bladder cancer were performed before starting therapy and were examined using a multigene sequencing panel.

**Results:**

Mutations in TP53, FGFR3, PIK3CA, KRAS, CTNNB1, ALK and DDR2 genes were detected. TP53 and FGFR3 were found to be the most frequently mutated genes in our cohort (31.6% and 26.3%, respectively), followed by PIK3CA (15.8%). In the BCG-responsive patient group, 90% of samples were found to have mutated genes, with almost 50% of them showing mutations in tyrosine kinase receptors and CTNNB1 genes. On the other hand, in the BCG-unresponsive group, we found mutations in 44.4% of samples, mainly in TP53 gene.

**Conclusions:**

Our findings suggest that a Next-Generation Sequencing (NGS) multigene panel is useful in predicting BCG response in patients with NMIBC.

## Introduction

Non-muscle invasive bladder cancer (NMIBC), which accounts for 75% of bladder-confined tumors, is divided into four risk groups (low, intermediate, high, and very high) according to the probability of progression to muscle-invasive disease [1]. Intravesical Bacillus Calmette-Guérin (BCG) therapy is a gold standard for intermediate- and high-risk patients [2]. It involves a six-week induction period with weekly intravesical BCG instillations, followed by a maintenance period of 1–3 years [3]. Although a complete and long-lasting response is observed in most patients, BCG failure occurs in 30%–50% of cases and a progression to muscle-invasive disease is found in 10%–15% [4–6]. In BCG-relapsing and -refractory patients, an additional course of immunotherapy can be implemented. However, radical cystectomy has to be performed when the disease progresses to muscle-invasive [7].

To date, the mechanism of BCG’s antitumor activity is not completely clear. An *in vitro* study demonstrated a direct cytotoxicity against cancer cells [8]. Furthermore, an immune-mediated activity, due to the production of cytokines and the activation of immune system cells (macrophages, natural killer cells, dendritic cells, and T lymphocytes), was suggested [9]. Response to BCG seems to be partially mediated by its efficient adhesion and internalization into the urothelial tumor cells [2,10]. Regarding the adhesion to cancer cells, several studies showed that this step can be driven by both chemical-physical and receptor-ligand interactions [10]. *In vitro* studies suggested that the mechanism of BCG internalization might be micropinocytosis. This process is used by cancer cells, as a pathway of endocytic absorption, that allows them to supply themselves with extracellular proteins. It ensures survival in difficult environments, such as tumor microenvironment [11]. The most plausible theory is that macropinocytosis in tumor cells is attributable to oncogenic mutations in RAS (H/N/K-RAS), PTEN, and PIK3CA [12]. *In vitro* studies showed that cancerous cells with mutations in the PTEN/PIK3 and RAS signaling pathways were the most sensitive to BCG therapy [11]. Therefore, BCG instillations might be beneficial in cancers that display mutations activating micropinocytosis. Nonetheless, the identification of specific biomarkers associated with BCG response is still challenging.

Next Generation Sequencing (NGS) and omics approach provided significant data on the genomic basis of bladder cancer. Also, they introduced distinct classifications within the spectrum of urothelial cancers with therapeutic and prognostic implications [13]. NGS is a technology used to determine the exact sequence of DNA or RNA. It allows to study of genetic variations associated with diseases or other biological phenomena. Omics are approaches that facilitate a comprehensive and unbiased examination of the genome, epigenome, transcriptome, proteome, and metabolome of the cancer. However, translating these approaches into the diagnostic routine is challenging, due to long turnaround time, complex methodology, and high costs [14]. Therefore, despite a number of molecular details that NGS provides, very few of them have acquired clinical relevance [15]. Instead, targeted NGS multigene panels are widely used in clinical practice to assess eligibility for anti-tumor-targeted therapies [16].

The aim of the present study was to determine whether the response to BCG treatment might be predicted by specific oncogenic mutations. We compared the “responder” and “non-responder” patient groups, using multigene panel sequencing.

## Materials and Methods

### Patients and data collection

The pretreatment archival tumors of 19 patients with histopathological diagnosis of primary high-risk NMIBC, treated with BCG between March 2015 and March 2020 were collected at the Policlinico Umberto I (Rome, Italy). High-risk NMIBC features and BCG failure definitions were adopted according to the European Association of Urology (EAU) Guidelines criteria [1]. Our study protocol inclusion criteria were as follows: males or females; age > 18 years; urothelial carcinoma without any histological variant; high-risk patients treated with BCG; a BCG failure in the first year or no recurrence at all; at least 3 years of follow-up; no previous lines of treatment received. The clinicopathological characteristics of the patients are summarized in Table 1 and described in the results section. Tissue samples were obtained by transurethral resection of bladder tumor (TURBT) performed by an experienced senior urologist before BCG treatment. All procedures followed the surgical recommendation listed in the EAU Guidelines using bipolar energy for resection [1]. Each resected bladder lesion was then numbered and separately sent for final histopathological examination annotating tumor/s location. Tissue samples were fixed in formalin and included in paraffin (FFPE). Briefly, the fresh tissue specimen was placed in the formaldehyde solution and then dehydrated through ethanol washes. After the clearing process using xylene, the sample was embedded in the molten paraffin wax within a mold. After histopathology evaluation performed by dedicated uropathologists, one or two sections of FFPE specimens were examined by NGS using a panel of clinically relevant genes [17]. For this proof-of-concept study, all investigations were approved by the local Ethics Committee of Policlinico Umberto I of Rome (Prot.: 88/18; RIF.CE:4903, 31 January 2018). All information regarding human material was managed using anonymous numerical codes and all samples were handled in compliance with the declaration of Helsinki. Written informed consent was obtained from all patients.

**Table 1:** Characteristics of the study cohort

Characteristics	N (%)
Sex	
Male	17 (89.5%)
Female	2 (10.5%)
Age (yrs)	
Median age (IQR)	72 (58–79.5)
Tumor Stage	
Ta	2 (10.5%)
T1	17 (89.5%)
CIS	2 (11.8%)
BCG response	
Responders	10 (52.6%)
Non-responders	9 (47.4%)
BCG-unresponsive patients	
BCG-relapsing	4 (21.1%)
BCG-refractory	5 (26.3%)

Note: Yrs = years; BCG = Bacillus Calmette-Guérin; IQR: interquartile range.

### DNA extraction from FFPE samples

FFPE tumor tissue specimens were collected and evaluated by a pathologist to assess the samples’ quality and quantity. When necessary, macrodissection was performed to reach at least 50% of tumor cell content. DNA extraction was performed as previously described [18]. Briefly, all paraffin was removed from tissue samples by consecutive washes with xylene and ethanol. DNA was extracted using a QIAamp DNA FFPE Tissue kit (cat. no. 56404; Qiagen GmbH, Hilden, Germany), according to the manufacturer’s instructions.

### Targeted sequencing and variant calling

NGS analysis was performed using a gene panel (cat. no. Guilford, CT 06437, USA; Thermo Fisher Scientific, Waltham, MA, USA) containing a single primer pool to amplify hotspots and targeted regions of 22 cancer-related genes: RAS (K-/N-RAS), EGFR, BRAF, PIK3CA, AKT1, ERBB2, PTEN, STK11, MAP2K1, ALK, DDR2, CTNNB1, MET, TP53, SMAD4, FBXW7, FGFR3, NOTCH1, ERBB4, FGFR1, and FGFR2 (Table S1). 10 ng of DNA input per sample was loaded onto Ion Chef Instrument (cat. no. 4484177; Thermo Fisher Scientific, Waltham, MA, USA) that fully automated library preparation, template preparation, and Ion 510 chip (cat. no. A34292, Thermo Fisher Scientific, Waltham, MA, USA) loading for the downstream sequencing through the Ion GeneStudio™ S5 (cat. no. A38194; Thermo Fisher Scientific, Waltham, MA, USA), following the manufacturer’s instructions. Sequencing data were analyzed with the Ion Torrent Suite Software (Thermo Fisher Scientific, Waltham, MA, USA. http://github.com/iontorrent/TS) (accessed on 11 October 2024) using Coverage Analysis, Molecular Coverage Analysis, Variant Caller plugins, and Ion Reporter Software according to the company’s recommendations. Variants were verified using the Integrative Genomics Viewer visualization tool (http://www.broadinstitute.org/igv/) (accessed on 11 October 2024).

### Statistical analysis

Categorical variables were reported as frequency distribution, whereas continuous variables were expressed as median and interquartile range. A Mann-Whitney U test was adopted to compare the measured values between the groups and a *p*-value < 0.05 was assumed to be statistically significant. As a merely explorative primordial assessment of the diagnostic performance of the single percentage (%) of allelic frequency mutation among the selected genes on BCG failure, the area under the receiving operator characteristic (ROC) curve (AUC) and its confidence interval (95% CI) level were computed. The analysis was carried out by using the OBM software SPSS 25.

## Results

### Study population

Pretreatment archival tumors of 19 patients with primary high-risk NMIBC treated with BCG were collected at Policlinico Umberto I. The median age was 72 years (interquartile range: 58–79.5). 2/19 (10.5%) of the patients had non-invasive papillary carcinoma (Ta), 17/19 (89.5%) had invasive subepithelial connective tissue cancer (T1), of whom 2 had carcinoma *in situ* (CIS) (Table 1).

All tumors were high-grade (according to the most up-to-date EAU Guidelines) and had urothelial histology. 73% of patients had multifocal lesions, and 79% had a lesion greater than 3 cm. The induction cycle with BCG and maintenance therapy were followed for all patients. The induction cycle involved a weekly instillation of intravesical BCG for six weeks and was followed by three years of maintenance therapy. The course of maintenance therapy involved 3 weekly instillations at 3 months and then 3 weekly instillations every 6 months for the remaining duration of the treatment. The full dose of BCG was used for both induction and maintenance therapy. Each patient was followed with a rigorous long-term follow-up program, which included cystoscopy and urinary cytology examinations every 3 months for 3 years, as well as computed urological tomography every 12 months. All patients in whom no malignant tumor was detected during follow-up, were considered as BCG-responsive. The study population, based on response to BCG therapy, was divided into two groups: responders (10/19; 52.6%), and non-responders (9/19; 47.4%). Furthermore, BCG-unresponsive patients were divided into BCG-relapsing (4/19; 21.1%) and BCG-refractory (5/19; 26.3%) subgroups. Those who initially responded to therapy, but developed high-grade disease, after completion of at least 1 maintenance cycle, were considered BCG-relapsing. Moreover, those in whom recurrence of high-grade disease was observed within six months of starting therapy or developed CIS within 12 months were defined as BCG-refractory.

### Assessment of the primary tumor’s mutational profile

**Figure 1:**
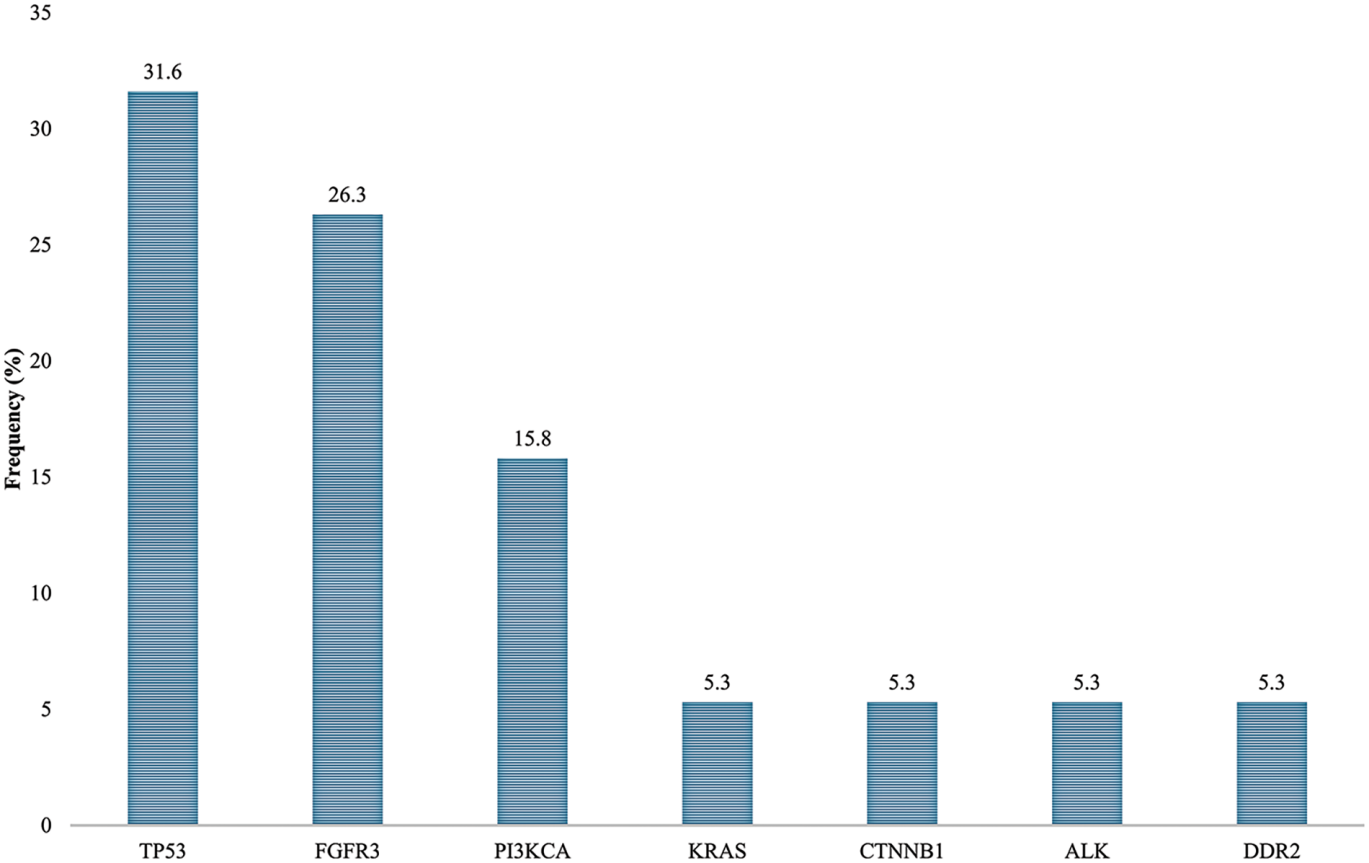
Mutation rates in non-muscle invasive bladder cancer patients.

In order to define the mutational profile of the primary tumor, 19 NMIBC samples were analyzed by multigene panel sequencing. In our cohort, TP53 and FGFR3 mutated genes were found the most frequently (TP53 in 6/19 cases, 31.6%; FGFR3 in 5/19, 26.3%), followed by PI3KCA (3/19, 15.8%) (Fig. 1; Table 2). Other somatic mutations were found with a lower rate in KRAS, CTNBB1, ALK, and DDR2 genes (1/19; 5.3%). Most of the mutations (18/19, 95%) were pathogenic or deleterious. Table 3 shows all the genetic variants found in 14 out of 19 NMIBC patients by targeted sequencing.

**Table 2:** Correlation between mutational profile and BGC response of non-muscle invasive bladder cancer patients

Patient ID	Sex	Stage	BCG response	22-gene panel sequencing						
				TP53	FGFR3	PIK3CA	KRAS	CTNNB1	ALK	DDR2
1	M	Ta	Responsive							
2	M	T1	Responsive							
3	M	T1	Responsive							
4	M	T1	Responsive							
5	M	T1	Responsive							
6	M	T1	Responsive							
7	M	T1	Responsive							
8	M	T1	Responsive							
9	M	T1	Responsive							
10	M	T1	Responsive							
11	M	Ta	Refractory							
12	F	T1	Relapsing							
13	M	T1	Refractory							
14	M	T1	Relapsing							
15	M	T1	Refractory							
16	M	T1	Refractory							
17	M	T1	Relapsing							
18	M	T1	Relapsing							
19	F	T1	Refractory							

Note: BCG = Bacillus Calmette-Guérin.

**Table 3:** Genetic variants found in non-muscle invasive bladder cancer patients by targeted sequencing

Patient	Gene	Mutations	AF (%)^A^	Relevance^B^	Prediction test^G^
1	TP53	c.750del p. I251Sfs*94	30.8	Not classified	PROVEAN: NA
					SIFT: NA
2	FGFR3	c.746C > G p.S249C	39.9	Pathogenic^C;^ ^D;^ ^E^	
3	PIK3CA	c.1633G > A p.E545K	33.4	Pathogenic; Likely pathogenic^C;^ ^D;^ ^E^	
4		None			
5	PIK3CA	c.1633G > C p. E545Q	34.9	Pathogenic; Likely pathogenic^C;^ ^D;^ ^E^	
	KRAS	c.351A > T p.K117N	57.3	Pathogenic^C;^ ^D;^ ^F^	
6	CTNBB1	c.110C > T p.S37F	23.0	Pathogenic; Likely pathogenic^C;^ ^D^	
7	FGFR3	c.742C > T p.R248C	46.9	Pathogenic^C;^ ^D;^ ^E^	
8	DDR2	c.106G > A p.V236M	53.1	Not classified	PolyPhen: possibly damaging; PROVEAN: neutral; SIFT: damaging
	ALK	c.3460G > A p.E1154K	20.2	Uncertain significance^D^	PolyPhen: possibly damaging; PROVEAN: deleterious; SIFT: tolerated
	FGFR3	c.1111A > T p.S371C	3.5	Pathogenic^C;^ ^D;^ ^E^	
	TP53	c.451C > T: p.P151S	18.7	Pathogenic/ Likely pathogenic^C;^ ^D;^ ^E;^ ^F^	
9	FGFR3	c.746C > G p.S249C	48.8	Pathogenic^C;^ ^D;^ ^E^	
10	PIK3CA	c.1633G > A p.E545K	25.0	Pathogenic; Likely pathogenic^C;^ ^D;^ ^E^	
	TP53	c.797G > A p.G266E	86.1	Pathogenic; Likely pathogenic^C;^ ^D;^ ^E;^ ^F^	
11	FGFR3	c.1118A > G p.Y373C	68.2	Likely pathogenic^C;^ ^D;^ ^E^	
12		None			
13	TP53	c.637C > T p.R213*	28.9	Pathogenic^C;^ ^D;^ ^E;^ ^F^	
		c.476C > A p.A159D	31.9	Pathogenic; Likely pathogenic^C;^ ^E;^ ^F^	
14	TP53	c.478_487delinsCTTTTCCATCAAC p.M160_A161delinsLFfs*	41.8	Not classified	PROVEAN: NA^H^
					SIFT: NA^H^
15	TP53	c.659A > G p.Y220C	13.2	Pathogenic^C;^ ^D;^ ^E;^ ^F^	PolyPhen: probably damaging; PROVEAN: neutral; SIFT: damaging
		c.466C > G p.R156G	26.2	Conflicting interpretations^C;^ ^D;^ ^E;^ ^F^	
16		None			
17		None			
18		None			
19		None			

Note: ^A^AF = Variant allele frequency in the tumor sample; ^B^Biological impact of the variant according to ^C^COSMIC (http://cancer.sanger.ac.uk/cosmic) (accessed on 11 October 2024); ^D^ClinVar (https://www.ncbi.nlm.nih.gov/clinvar/) (accessed on 11 October 2024), ^E^OncoKB (https://www.oncokb.org) (accessed on 11 October 2024), ^F^IARC database (https://tp53.isb-cgc.org) (accessed on 11 October 2024); ^G^*In silico* prediction on the biological impact of the variant according to PolyPhen-2 (http://genetics.bwh.harvard.edu/pph2/) (accessed on 11 October 2024) SIFT and PROVEAN (http://provean.jcvi.org/genome_submit_2.php?species=human) (accessed on 11 October 2024); ^H^Not Available: the prediction test could not be performed.

### Mutational profile and BCG response

In the group of BCG responders, at least one mutation was detected in all patients, except for one. PI3KCA mutations were identified in 3/10 (30%) samples. Furthermore, mutations of the TP53 gene were detected in 3/10 (30%) samples, while in 1/10 (10%) we detected CTNBB1 mutation. Five mutations related to tyrosine kinase receptors (RTKs) were identified in 4/10 (40%) patients. Specifically, in 1 out of 10 (10%) samples, we identified coexisting mutations of ALK, DDR2, and FGFR3. FGFR3 mutations were detected in 3 out of 5 samples. KRAS mutations were detected in 1/10 (10%) samples (Table 2; Table 3).

**Figure 2:**
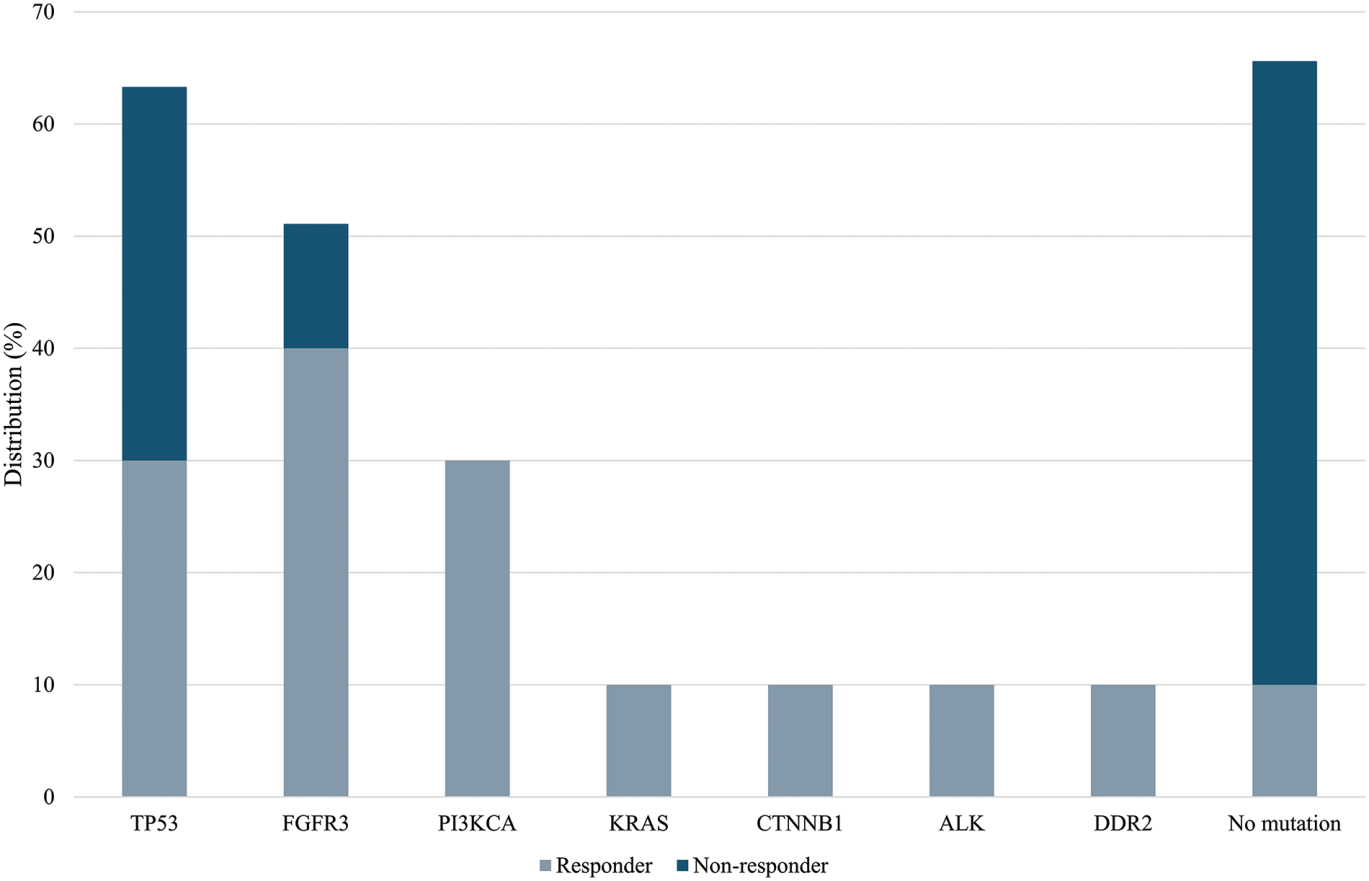
Frequency of gene mutation in BCG-responders and BCG-unresponsive groups. A comparison of mutations between the BCG-responder and -non-responder groups show a mean value of 1.44 ± 1.08 and 0.44 ± 0.52 (*p*-value = 0.028), respectively.

In the group of BCG-unresponsive patients, no RAS and PI3KCA mutations were identified. Overall, 6 mutations were identified in 4/9 (44.4%) patients. Two coexisting TP53 mutations were detected in 2/9 (22.2%) samples, while 1 specimen displayed a single TP53 mutation, for a total of 3 out of 9 (33.3%) samples showing TP53 mutations. FGFR3 mutation was detected in 1/9 (11.1%) samples (Table 2). Lastly, 5/9 (55.6%) of the unresponsive patients had no mutations. A comparison of the overall mutations between the BCG-responders and -non-responders groups show a mean value of 1.44 ± 1.08 and 0.44 ± 0.52 (*p*-value = 0.028), respectively. Fig. 2 shows the distribution of gene mutations in both groups. Explorative diagnostic AUC analysis of each gene tested for BCG failure outcome is presented in Fig. S1.

## Discussion

Intravesical BCG is a therapeutic gold standard for patients with high-risk NMIBC. Unfortunately, 25%–45% of these patients do not benefit from therapy, while 40% develop disease recurrence after initial therapeutic success [4]. The phenomenon of BCG unresponsive disease highlighted the need to identify predictive biomarkers that enable appropriate therapeutic choices for patients with high-risk NMIBC (TaG3/T1G3/CIS).

Although BCG effectiveness seems closely related to its internalization in cancer cells, its true mechanism of action remains elusive to date. *In vitro* and *in vivo* evidence showed BCG to selectively enter cancer cells through macropinocytosis, a process activated specifically by cancer cells, which is strongly associated with the acquisition of oncogenic mutations [11]. For example, Redelman-Sidi et al. observed that cell lines and animal models with oncogenic mutations in RAS and PI3K signaling pathways resulted in an increased response to BCG [11]. RAS mutant cell lines displayed significant morphological changes, such as specific alterations of the cytoplasmic membrane that might represent the onset of macropinocytosis. However, macropinosome formation exploits several biochemical pathways, including the activation of PI3K [19]. The PI3K pathway drives phosphorylation of phosphatidylinositol-4,5-bisphosphate (PIP2) to synthesize phosphatidylinositol-3,4,5-triphosphate (PIP3). High concentrations of PIP3 activate binding domains on several proteins, including serine-threonine kinase (AKT) and guanine-nucleotide exchange factor (GEF), which in turn activate GTPases as RAS proteins [19,20]. These signaling proteins, therefore, play a decisive role in the signaling associated with macropinocytosis [12].

Swanson et al. demonstrated that in some cell lines, the shutdown of the PI3K-driven signal pathway inhibits cell membrane wrinkling, suggesting that PIP3 deficiency does not lead to GTPase activation, thus impairing macropinocytosis [19]. However, in the majority of the analyzed cell lines, the inactivation of PI3K does not reduce the wrinkling of the membrane. Rather, it inhibits the closure of macropinosomes and the ability of the macropinocytosis to transport extracellular fluid inside. Nevertheless, macropinocytosis is not initiated when PI3K is constitutively activated, but only following PI3KCA mutations or PTEN deletions. The latter leads to a deregulation of the signaling pathway [21].

The use of NGS/omics approaches brought new insights into the genomic and immune dynamics of bladder cancer treated with BCG. Also, they provided a huge number of information regarding clinically relevant biomarkers [22,23]. Even though these wide-ranging methods are not readily suitable for the clinical setting, multigene panels (targeted NGS) are routinely used in the clinical setting nowadays [16].

To clarify the impact of specific oncogenic mutations on BCG efficacy, we analyzed tissue samples from 19 NMIBCs patients, classified according to their treatment response, using a multigene sequencing panel. We found TP53 and FGFR3 to be the most frequently mutated genes, followed by PI3KCA, recapitulating what other studies reported [24]. Our data did not evidence any relation between RAS mutations and therapeutic response, unlike what was reported in the literature [11]. Even though PIK3CA mutations seem to have a greater impact on BCG efficacy, mutations attributable to the PI3KCA oncogene were found only in 3/10 (30%) responsive patients. Moreover, no mutations of the onco-suppressor PTEN (a negative regulator of the PIK3CA signaling pathway) emerged. Unfortunately, due to the lack of sample material, PTEN loss was not assessed. Therefore, we were not able to confirm data describing RAS and PIK3CA mutations as the main determinant of micropinocytosis, as Redelman-Sidi et al. suggested. Nevertheless, we hypothesized that other mutations equally involved in the process of macropinocytosis activation might account for BCG efficacy [11]. Indeed, in our cohort of BCG-responsive patients, about 50% of the mutations were identified on genes encoding RTKs (i.e., FGFR3, ALK, and DDR2) and CTNNB1. This analysis provided a broader mutational profile, highlighting the important role of RTKs, PIK3CA, and Wnt/β-catenin mutations in the success of intravesical BCG therapy. In fact, it is already known that the signaling pathways of RAS, PAK1, PTEN/PIK3CA, Wnt/β-Catenin and the downstream activation of RTKs receptors are involved in the activation of macropinocytosis *in vitro* [11,25]. Nevertheless, a recent study about the role of common immune-related genes and tumor microenvironment (TME) on BCG response, showed the relation between FGFR3 overexpression/mutations and a “cold” TME to be associated with BCG resistance and recurrence, which is in contrast with our findings [26]. In another study, despite confirming the relation between FGFR3 mutations and a “cold” TME, the authors observed this T-cell exclusion associated with a better high-grade recurrence-free survival (RFS) post-BCG [27].

Moreover, in one BCG-responsive patient, a mutation of the gene encoding β-catenin (CTNNB1) was detected. β-catenin is a crucial element of the Wnt signaling pathway, a branched signal transduction pathway. In the absence of Wnt, the cytoplasmic β-catenin protein is phosphorylated by the degradation complex and degraded by a ubiquitin ligase. When Wnt binds its receptor, β-catenin accumulates and translocates into the nucleus with a consequent activation of target genes [28]. The Wnt signaling pathway undergoes a negative regulation through the internalization of the receptor by cell surface and through proteolytic control of the transcription factor CTNNB1 in the destruction complex. Consequently, mutations of the CTNNB1 gene are involved in hyperactivation of the Wnt/β-catenin signaling pathway [24,28].

In the group of unresponsive patients, 3 mutations of TP53 were identified. Mutations in the TP53 tumor suppressor gene, detected in 6/19 (31.6%) patients, are frequently detected in many human cancers and are the most common genetic alterations in invasive bladder tumors. A mutated p53 protein leads to the acquisition of pro-oncogenic properties, including increased cell proliferation, migration, invasion, the onset of drug resistance phenomena, as well as promotion of neo-angiogenesis. Patients with bladder cancers harboring TP53 mutations generally have a worse prognosis [29].

The major limitation of this study is constituted by the small sample size, which is mainly related to the strictness in patients’ selection, as well as the fact that this is a single center-based study. A future implementation is needed to consolidate the results obtained. Furthermore, the analysis of the mutational status of specific genes, which have been reported to be predictive of BCG responsiveness (i.e., mutations in telomerase reverse transcriptase gene promoter) [30] and unresponsiveness (i.e., mutations in AT-rich interactive domain-containing protein 1A gene) [31,32]. Also, the relation between BCG effectiveness and the immune system must be included in future investigations, to accurately identify NMIBC patients, who are more likely to respond to BCG treatment.

Globally, BGC-responsive patients show a greater number of mutations than unresponsive patients and this data is statistically significant. These results are in line with experimental evidence in the literature and confirm the role of oncogenic aberrations in patients’ response to intravesical BCG. These results suggest that genetic aberrations related to RTKs, PIK3CA, and Wnt/β-catenin might represent predictive biomarkers of the therapeutic efficacy of BCG. Our research is a proof-of-concept study and is currently far from any possible clinical application to further shape the therapeutic algorithm of high-risk NMIBCs. Our detected differences in allelic frequency mutations could serve as positive feedback to further expand our series and conduct novel observational prospective studies in the near future. Clinical implications that may be driven by additional positive feedback and a higher level of evidence may facilitate the decision between radical surgery and bladder preservation strategy including intravesical BCG continuation. This is especially important if we consider that progression to muscle-invasive disease after BCG failure in primary high-risk NMIBC is associated with significantly worse cancer-specific survival (CSS) when compared to those patients treated with radical cystectomy [33,34]. As a consequence, the identification of an oncogenic mutation profile may represent a potential game changer in the decision-making scenario shared with the patients. While these suggestions and potential perspectives remain attractive, higher confirmative levels of evidence studies, and additional prospective and randomized studies are necessary. Finally, our study is not devoid of limitations. First and most important, given the “proof of concept” and retrospective nature of the study our sample size is significantly limited and the level of evidence generating is purely descriptive with the final intent of exploring our panel in a later larger longitudinal series. Similarly, the inclusion criteria have been highly restricted to the sole higher-risk NMIBCs category thus potentially not reflecting the whole spectrum of patients. However, high-risk NMIBC represents the most clinically challenging sub-group with significant implications on decision-making thus being the most interesting category to further asses even in future investigations. More specifically, given the first analysis of our study is made on paraffined tissue, supplemental analysis including PTEN loss evaluation was missing in the light of exploring the process of macropinocytosis of BCG by bladder cancer cells. Future implementation of the current process should also include immune infiltrate analysis of the tumor resected tissue (i.e., hematoxylin-eosin staining or immunohistochemistry) to evaluate the contribution of immune cells in the tumor microenvironment. Finally, a larger series should balance the impact of therapeutic BCG factors such as differences across BCG strains and the extent of exposure over the maintenance schedule.

## Conclusions

In conclusion, assessment of the mutational status of the tissue samples through a multigene sequencing panel might potentially optimize the clinical decision-making process in the future. While our results are primordial, they are suggestive of the possible important differences in genetic mutational burden at bladder cancer diagnosis. These could drive the subsequent patient-physician therapeutic decisions. This initial experience will provide the evidence to further test our selected panel on a larger group in a prospective and longitudinal cohort study. The next steps of our planned strategy will be to focus on the diagnostic performance indicators, the prognostic influence of each mutated gene, and the overall accuracy of the panel.

## Supplementary Materials

Figure S1 Exploratory initial assessment of area under the receiving operator characteristic (ROC) curve (AUC), with its confidence interval (95%CI), of each gene tested for BCG failure outcome.

## Data Availability

The datasets generated during and/or analyzed during the current study are available from the corresponding author upon reasonable request.

## Supplementary Materials

The supplementary material is available online at https://doi.org/10.32604/or.2024.056282.
