# Supplementary figures and images for: Exploring the utility of a NGS multigene panel to predict BCG response in patients with non-muscle invasive bladder cancer
Source: Oncol Res. 2025 Feb 28;33(3):723–31. doi: 10.32604/or.2024.056282 (PMC11915050; doi:10.32604/or.2024.056282)

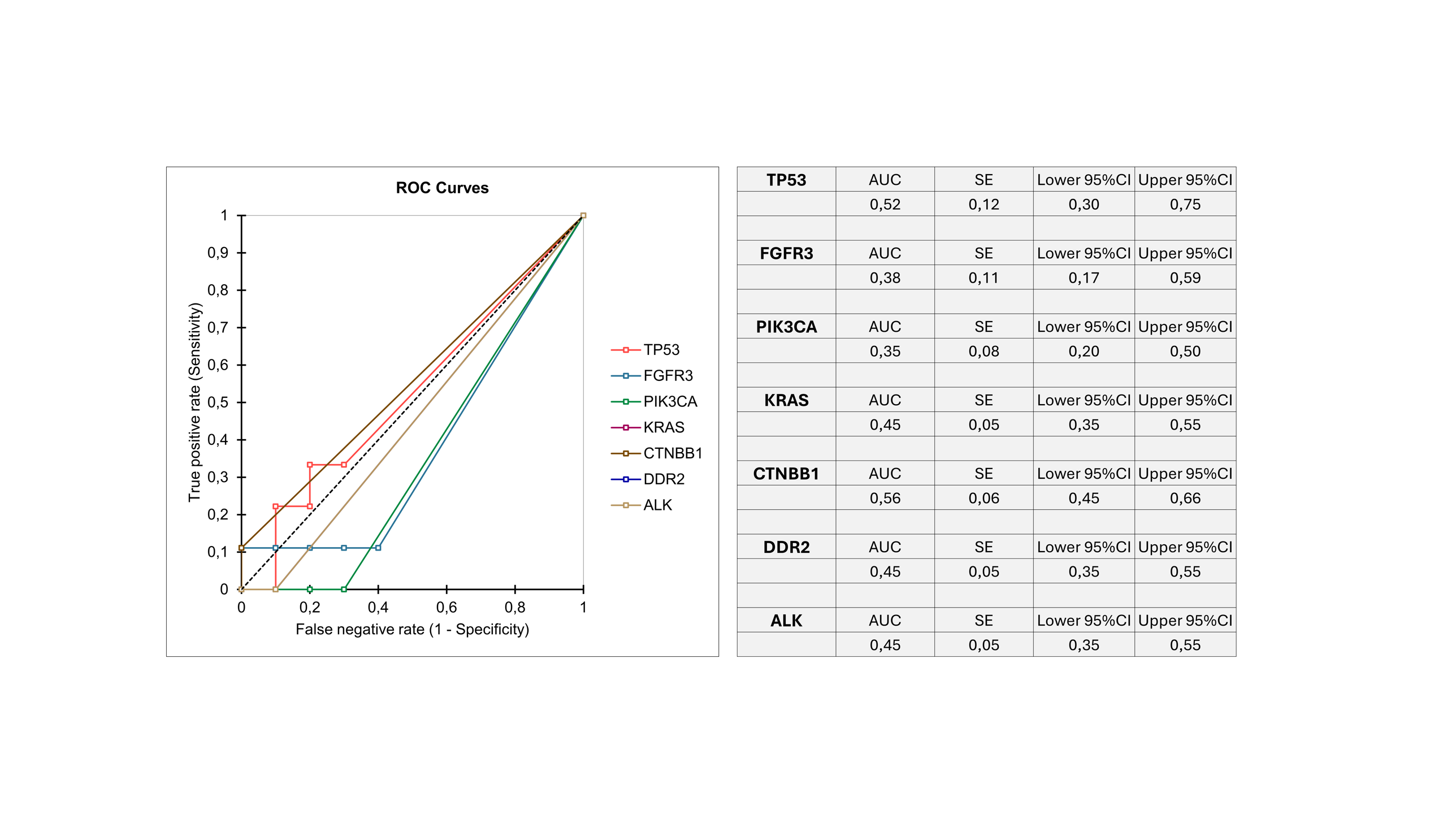

Supplement: Figure S1 [file OncolRes-33-56282-s001.tif]
