# Supplementary material for: Exploring the utility of a NGS multigene panel to predict BCG response in patients with non-muscle invasive bladder cancer
Source: Oncol Res. 2025 Feb 28;33(3):723–31. doi: 10.32604/or.2024.056282 (PMC11915050; doi:10.32604/or.2024.056282)
Supplement: Supplementary file 2 [file OncolRes-33-56282-s002.docx]

**Table S1.** Amplicon panel data sheet

| Amplicon_ID | Ion_AmpliSeq_Fwd_Primer | Ion_AmpliSeq_Rev_Primer | LineItem_Type | Gene_Symbol | Genome_Version | Chr | Amplicon_Start | Amplicon_Stop | Insert_Start | Insert_Stop | Pool_ID | Pool_Name |
| --- | --- | --- | --- | --- | --- | --- | --- | --- | --- | --- | --- | --- |
| CCP_EGFR_6 | CCACACTGACGTGCCTCTC | GTCTTTGTGTTCCCGGACATAGT | GENOME_REGION | COSM12377 | hg19 | chr7 | 55248956 | 5,5E+07 | 55248975 | 55249100 | 1 | Pool1 |
| CCP_EGFR_6 | CCACACTGACGTGCCTCTC | GTCTTTGTGTTCCCGGACATAGT | GENOME_REGION | COSM12378 | hg19 | chr7 | 55248956 | 5,5E+07 | 55248975 | 55249100 | 1 | Pool1 |
| CCP_EGFR_6 | CCACACTGACGTGCCTCTC | GTCTTTGTGTTCCCGGACATAGT | GENOME_REGION | COSM12427 | hg19 | chr7 | 55248956 | 5,5E+07 | 55248975 | 55249100 | 1 | Pool1 |
| CCP_EGFR_6 | CCACACTGACGTGCCTCTC | GTCTTTGTGTTCCCGGACATAGT | GENOME_REGION | COSM13005 | hg19 | chr7 | 55248956 | 5,5E+07 | 55248975 | 55249100 | 1 | Pool1 |
| CCP_EGFR_6 | CCACACTGACGTGCCTCTC | GTCTTTGTGTTCCCGGACATAGT | GENOME_REGION | COSM13006 | hg19 | chr7 | 55248956 | 5,5E+07 | 55248975 | 55249100 | 1 | Pool1 |
| CCP_EGFR_6 | CCACACTGACGTGCCTCTC | GTCTTTGTGTTCCCGGACATAGT | GENOME_REGION | COSM13007 | hg19 | chr7 | 55248956 | 5,5E+07 | 55248975 | 55249100 | 1 | Pool1 |
| CCP_EGFR_6 | CCACACTGACGTGCCTCTC | GTCTTTGTGTTCCCGGACATAGT | GENOME_REGION | COSM13189 | hg19 | chr7 | 55248956 | 5,5E+07 | 55248975 | 55249100 | 1 | Pool1 |
| CCP_EGFR_6 | CCACACTGACGTGCCTCTC | GTCTTTGTGTTCCCGGACATAGT | GENOME_REGION | COSM13190 | hg19 | chr7 | 55248956 | 5,5E+07 | 55248975 | 55249100 | 1 | Pool1 |
| CCP_EGFR_6 | CCACACTGACGTGCCTCTC | GTCTTTGTGTTCCCGGACATAGT | GENOME_REGION | COSM133565 | hg19 | chr7 | 55248956 | 5,5E+07 | 55248975 | 55249100 | 1 | Pool1 |
| CCP_EGFR_6 | CCACACTGACGTGCCTCTC | GTCTTTGTGTTCCCGGACATAGT | GENOME_REGION | COSM13433 | hg19 | chr7 | 55248956 | 5,5E+07 | 55248975 | 55249100 | 1 | Pool1 |
| CCP_EGFR_6 | CCACACTGACGTGCCTCTC | GTCTTTGTGTTCCCGGACATAGT | GENOME_REGION | COSM14068 | hg19 | chr7 | 55248956 | 5,5E+07 | 55248975 | 55249100 | 1 | Pool1 |
| CCP_EGFR_6 | CCACACTGACGTGCCTCTC | GTCTTTGTGTTCCCGGACATAGT | GENOME_REGION | COSM20891 | hg19 | chr7 | 55248956 | 5,5E+07 | 55248975 | 55249100 | 1 | Pool1 |
| CCP_EGFR_6 | CCACACTGACGTGCCTCTC | GTCTTTGTGTTCCCGGACATAGT | GENOME_REGION | COSM22940 | hg19 | chr7 | 55248956 | 5,5E+07 | 55248975 | 55249100 | 1 | Pool1 |
| CCP_EGFR_6 | CCACACTGACGTGCCTCTC | GTCTTTGTGTTCCCGGACATAGT | GENOME_REGION | COSM22951 | hg19 | chr7 | 55248956 | 5,5E+07 | 55248975 | 55249100 | 1 | Pool1 |
| CCP_EGFR_6 | CCACACTGACGTGCCTCTC | GTCTTTGTGTTCCCGGACATAGT | GENOME_REGION | COSM22954 | hg19 | chr7 | 55248956 | 5,5E+07 | 55248975 | 55249100 | 1 | Pool1 |
| CCP_EGFR_6 | CCACACTGACGTGCCTCTC | GTCTTTGTGTTCCCGGACATAGT | GENOME_REGION | COSM26445 | hg19 | chr7 | 55248956 | 5,5E+07 | 55248975 | 55249100 | 1 | Pool1 |
| CCP_EGFR_6 | CCACACTGACGTGCCTCTC | GTCTTTGTGTTCCCGGACATAGT | GENOME_REGION | COSM27110 | hg19 | chr7 | 55248956 | 5,5E+07 | 55248975 | 55249100 | 1 | Pool1 |
| CCP_EGFR_6 | CCACACTGACGTGCCTCTC | GTCTTTGTGTTCCCGGACATAGT | GENOME_REGION | COSM27568 | hg19 | chr7 | 55248956 | 5,5E+07 | 55248975 | 55249100 | 1 | Pool1 |
| CCP_EGFR_6 | CCACACTGACGTGCCTCTC | GTCTTTGTGTTCCCGGACATAGT | GENOME_REGION | COSM28513 | hg19 | chr7 | 55248956 | 5,5E+07 | 55248975 | 55249100 | 1 | Pool1 |
| CCP_EGFR_6 | CCACACTGACGTGCCTCTC | GTCTTTGTGTTCCCGGACATAGT | GENOME_REGION | COSM28603 | hg19 | chr7 | 55248956 | 5,5E+07 | 55248975 | 55249100 | 1 | Pool1 |
| CCP_EGFR_6 | CCACACTGACGTGCCTCTC | GTCTTTGTGTTCCCGGACATAGT | GENOME_REGION | COSM48922 | hg19 | chr7 | 55248956 | 5,5E+07 | 55248975 | 55249100 | 1 | Pool1 |
| CCP_EGFR_6 | CCACACTGACGTGCCTCTC | GTCTTTGTGTTCCCGGACATAGT | GENOME_REGION | COSM6226 | hg19 | chr7 | 55248956 | 5,5E+07 | 55248975 | 55249100 | 1 | Pool1 |
| CCP_EGFR_6 | CCACACTGACGTGCCTCTC | GTCTTTGTGTTCCCGGACATAGT | GENOME_REGION | COSM6240 | hg19 | chr7 | 55248956 | 5,5E+07 | 55248975 | 55249100 | 1 | Pool1 |
| CCP_EGFR_6 | CCACACTGACGTGCCTCTC | GTCTTTGTGTTCCCGGACATAGT | GENOME_REGION | COSM6241 | hg19 | chr7 | 55248956 | 5,5E+07 | 55248975 | 55249100 | 1 | Pool1 |
| CCP_EGFR_6 | CCACACTGACGTGCCTCTC | GTCTTTGTGTTCCCGGACATAGT | GENOME_REGION | COSM6242 | hg19 | chr7 | 55248956 | 5,5E+07 | 55248975 | 55249100 | 1 | Pool1 |
| CHP2_AKT1_1 | GCGCCACAGAGAAGTTGTTGA | GGGTCTGACGGGTAGAGTGT | GENOME_REGION | COSM33765 | hg19 | chr14 | 105246425 | 1,1E+08 | 105246446 | 105246583 | 1 | Pool1 |
| CHP2_BRAF_1 | CATACTTACCATGCCACTTTCCCTT | TTTCTTTTTCTGTTTGGCTTGACTTGA | GENOME_REGION | COSM1112 | hg19 | chr7 | 140481367 | 1,4E+08 | 140481392 | 140481515 | 1 | Pool1 |
| CHP2_BRAF_1 | CATACTTACCATGCCACTTTCCCTT | TTTCTTTTTCTGTTTGGCTTGACTTGA | GENOME_REGION | COSM21492 | hg19 | chr7 | 140481367 | 1,4E+08 | 140481392 | 140481515 | 1 | Pool1 |
| CHP2_BRAF_1 | CATACTTACCATGCCACTTTCCCTT | TTTCTTTTTCTGTTTGGCTTGACTTGA | GENOME_REGION | COSM447 | hg19 | chr7 | 140481367 | 1,4E+08 | 140481392 | 140481515 | 1 | Pool1 |
| CHP2_BRAF_1 | CATACTTACCATGCCACTTTCCCTT | TTTCTTTTTCTGTTTGGCTTGACTTGA | GENOME_REGION | COSM449 | hg19 | chr7 | 140481367 | 1,4E+08 | 140481392 | 140481515 | 1 | Pool1 |
| CHP2_BRAF_1 | CATACTTACCATGCCACTTTCCCTT | TTTCTTTTTCTGTTTGGCTTGACTTGA | GENOME_REGION | COSM450 | hg19 | chr7 | 140481367 | 1,4E+08 | 140481392 | 140481515 | 1 | Pool1 |
| CHP2_BRAF_1 | CATACTTACCATGCCACTTTCCCTT | TTTCTTTTTCTGTTTGGCTTGACTTGA | GENOME_REGION | COSM451 | hg19 | chr7 | 140481367 | 1,4E+08 | 140481392 | 140481515 | 1 | Pool1 |
| CHP2_BRAF_1 | CATACTTACCATGCCACTTTCCCTT | TTTCTTTTTCTGTTTGGCTTGACTTGA | GENOME_REGION | COSM453 | hg19 | chr7 | 140481367 | 1,4E+08 | 140481392 | 140481515 | 1 | Pool1 |
| CHP2_BRAF_1 | CATACTTACCATGCCACTTTCCCTT | TTTCTTTTTCTGTTTGGCTTGACTTGA | GENOME_REGION | COSM457 | hg19 | chr7 | 140481367 | 1,4E+08 | 140481392 | 140481515 | 1 | Pool1 |
| CHP2_BRAF_1 | CATACTTACCATGCCACTTTCCCTT | TTTCTTTTTCTGTTTGGCTTGACTTGA | GENOME_REGION | COSM458 | hg19 | chr7 | 140481367 | 1,4E+08 | 140481392 | 140481515 | 1 | Pool1 |
| CHP2_BRAF_1 | CATACTTACCATGCCACTTTCCCTT | TTTCTTTTTCTGTTTGGCTTGACTTGA | GENOME_REGION | COSM459 | hg19 | chr7 | 140481367 | 1,4E+08 | 140481392 | 140481515 | 1 | Pool1 |
| CHP2_BRAF_1 | CATACTTACCATGCCACTTTCCCTT | TTTCTTTTTCTGTTTGGCTTGACTTGA | GENOME_REGION | COSM460 | hg19 | chr7 | 140481367 | 1,4E+08 | 140481392 | 140481515 | 1 | Pool1 |
| CHP2_BRAF_1 | CATACTTACCATGCCACTTTCCCTT | TTTCTTTTTCTGTTTGGCTTGACTTGA | GENOME_REGION | COSM461 | hg19 | chr7 | 140481367 | 1,4E+08 | 140481392 | 140481515 | 1 | Pool1 |
| CHP2_BRAF_1 | CATACTTACCATGCCACTTTCCCTT | TTTCTTTTTCTGTTTGGCTTGACTTGA | GENOME_REGION | COSM6262 | hg19 | chr7 | 140481367 | 1,4E+08 | 140481392 | 140481515 | 1 | Pool1 |
| CHP2_BRAF_2 | CCACAAAATGGATCCAGACAACTGT | GCTTGCTCTGATAGGAAAATGAGATCTA | GENOME_REGION | COSM1115 | hg19 | chr7 | 140453078 | 1,4E+08 | 140453103 | 140453221 | 1 | Pool1 |
| CHP2_BRAF_2 | CCACAAAATGGATCCAGACAACTGT | GCTTGCTCTGATAGGAAAATGAGATCTA | GENOME_REGION | COSM1116 | hg19 | chr7 | 140453078 | 1,4E+08 | 140453103 | 140453221 | 1 | Pool1 |
| CHP2_BRAF_2 | CCACAAAATGGATCCAGACAACTGT | GCTTGCTCTGATAGGAAAATGAGATCTA | GENOME_REGION | COSM1117 | hg19 | chr7 | 140453078 | 1,4E+08 | 140453103 | 140453221 | 1 | Pool1 |
| CHP2_BRAF_2 | CCACAAAATGGATCCAGACAACTGT | GCTTGCTCTGATAGGAAAATGAGATCTA | GENOME_REGION | COSM1118 | hg19 | chr7 | 140453078 | 1,4E+08 | 140453103 | 140453221 | 1 | Pool1 |
| CHP2_BRAF_2 | CCACAAAATGGATCCAGACAACTGT | GCTTGCTCTGATAGGAAAATGAGATCTA | GENOME_REGION | COSM1119 | hg19 | chr7 | 140453078 | 1,4E+08 | 140453103 | 140453221 | 1 | Pool1 |
| CHP2_BRAF_2 | CCACAAAATGGATCCAGACAACTGT | GCTTGCTCTGATAGGAAAATGAGATCTA | GENOME_REGION | COSM1120 | hg19 | chr7 | 140453078 | 1,4E+08 | 140453103 | 140453221 | 1 | Pool1 |
| CHP2_BRAF_2 | CCACAAAATGGATCCAGACAACTGT | GCTTGCTCTGATAGGAAAATGAGATCTA | GENOME_REGION | COSM1123 | hg19 | chr7 | 140453078 | 1,4E+08 | 140453103 | 140453221 | 1 | Pool1 |
| CHP2_BRAF_2 | CCACAAAATGGATCCAGACAACTGT | GCTTGCTCTGATAGGAAAATGAGATCTA | GENOME_REGION | COSM1124 | hg19 | chr7 | 140453078 | 1,4E+08 | 140453103 | 140453221 | 1 | Pool1 |
| CHP2_BRAF_2 | CCACAAAATGGATCCAGACAACTGT | GCTTGCTCTGATAGGAAAATGAGATCTA | GENOME_REGION | COSM1125 | hg19 | chr7 | 140453078 | 1,4E+08 | 140453103 | 140453221 | 1 | Pool1 |
| CHP2_BRAF_2 | CCACAAAATGGATCCAGACAACTGT | GCTTGCTCTGATAGGAAAATGAGATCTA | GENOME_REGION | COSM1126 | hg19 | chr7 | 140453078 | 1,4E+08 | 140453103 | 140453221 | 1 | Pool1 |
| CHP2_BRAF_2 | CCACAAAATGGATCCAGACAACTGT | GCTTGCTCTGATAGGAAAATGAGATCTA | GENOME_REGION | COSM1128 | hg19 | chr7 | 140453078 | 1,4E+08 | 140453103 | 140453221 | 1 | Pool1 |
| CHP2_BRAF_2 | CCACAAAATGGATCCAGACAACTGT | GCTTGCTCTGATAGGAAAATGAGATCTA | GENOME_REGION | COSM1130 | hg19 | chr7 | 140453078 | 1,4E+08 | 140453103 | 140453221 | 1 | Pool1 |
| CHP2_BRAF_2 | CCACAAAATGGATCCAGACAACTGT | GCTTGCTCTGATAGGAAAATGAGATCTA | GENOME_REGION | COSM1132 | hg19 | chr7 | 140453078 | 1,4E+08 | 140453103 | 140453221 | 1 | Pool1 |
| CHP2_BRAF_2 | CCACAAAATGGATCCAGACAACTGT | GCTTGCTCTGATAGGAAAATGAGATCTA | GENOME_REGION | COSM1133 | hg19 | chr7 | 140453078 | 1,4E+08 | 140453103 | 140453221 | 1 | Pool1 |
| CHP2_BRAF_2 | CCACAAAATGGATCCAGACAACTGT | GCTTGCTCTGATAGGAAAATGAGATCTA | GENOME_REGION | COSM1134 | hg19 | chr7 | 140453078 | 1,4E+08 | 140453103 | 140453221 | 1 | Pool1 |
| CHP2_BRAF_2 | CCACAAAATGGATCCAGACAACTGT | GCTTGCTCTGATAGGAAAATGAGATCTA | GENOME_REGION | COSM1135 | hg19 | chr7 | 140453078 | 1,4E+08 | 140453103 | 140453221 | 1 | Pool1 |
| CHP2_BRAF_2 | CCACAAAATGGATCCAGACAACTGT | GCTTGCTCTGATAGGAAAATGAGATCTA | GENOME_REGION | COSM1136 | hg19 | chr7 | 140453078 | 1,4E+08 | 140453103 | 140453221 | 1 | Pool1 |
| CHP2_BRAF_2 | CCACAAAATGGATCCAGACAACTGT | GCTTGCTCTGATAGGAAAATGAGATCTA | GENOME_REGION | COSM1137 | hg19 | chr7 | 140453078 | 1,4E+08 | 140453103 | 140453221 | 1 | Pool1 |
| CHP2_BRAF_2 | CCACAAAATGGATCCAGACAACTGT | GCTTGCTCTGATAGGAAAATGAGATCTA | GENOME_REGION | COSM1138 | hg19 | chr7 | 140453078 | 1,4E+08 | 140453103 | 140453221 | 1 | Pool1 |
| CHP2_BRAF_2 | CCACAAAATGGATCCAGACAACTGT | GCTTGCTCTGATAGGAAAATGAGATCTA | GENOME_REGION | COSM144982 | hg19 | chr7 | 140453078 | 1,4E+08 | 140453103 | 140453221 | 1 | Pool1 |
| CHP2_BRAF_2 | CCACAAAATGGATCCAGACAACTGT | GCTTGCTCTGATAGGAAAATGAGATCTA | GENOME_REGION | COSM18443 | hg19 | chr7 | 140453078 | 1,4E+08 | 140453103 | 140453221 | 1 | Pool1 |
| CHP2_BRAF_2 | CCACAAAATGGATCCAGACAACTGT | GCTTGCTCTGATAGGAAAATGAGATCTA | GENOME_REGION | COSM21542 | hg19 | chr7 | 140453078 | 1,4E+08 | 140453103 | 140453221 | 1 | Pool1 |
| CHP2_BRAF_2 | CCACAAAATGGATCCAGACAACTGT | GCTTGCTCTGATAGGAAAATGAGATCTA | GENOME_REGION | COSM21549 | hg19 | chr7 | 140453078 | 1,4E+08 | 140453103 | 140453221 | 1 | Pool1 |
| CHP2_BRAF_2 | CCACAAAATGGATCCAGACAACTGT | GCTTGCTCTGATAGGAAAATGAGATCTA | GENOME_REGION | COSM219798 | hg19 | chr7 | 140453078 | 1,4E+08 | 140453103 | 140453221 | 1 | Pool1 |
| CHP2_BRAF_2 | CCACAAAATGGATCCAGACAACTGT | GCTTGCTCTGATAGGAAAATGAGATCTA | GENOME_REGION | COSM249889 | hg19 | chr7 | 140453078 | 1,4E+08 | 140453103 | 140453221 | 1 | Pool1 |
| CHP2_BRAF_2 | CCACAAAATGGATCCAGACAACTGT | GCTTGCTCTGATAGGAAAATGAGATCTA | GENOME_REGION | COSM26506 | hg19 | chr7 | 140453078 | 1,4E+08 | 140453103 | 140453221 | 1 | Pool1 |
| CHP2_BRAF_2 | CCACAAAATGGATCCAGACAACTGT | GCTTGCTCTGATAGGAAAATGAGATCTA | GENOME_REGION | COSM26625 | hg19 | chr7 | 140453078 | 1,4E+08 | 140453103 | 140453221 | 1 | Pool1 |
| CHP2_BRAF_2 | CCACAAAATGGATCCAGACAACTGT | GCTTGCTCTGATAGGAAAATGAGATCTA | GENOME_REGION | COSM27639 | hg19 | chr7 | 140453078 | 1,4E+08 | 140453103 | 140453221 | 1 | Pool1 |
| CHP2_BRAF_2 | CCACAAAATGGATCCAGACAACTGT | GCTTGCTCTGATAGGAAAATGAGATCTA | GENOME_REGION | COSM28010 | hg19 | chr7 | 140453078 | 1,4E+08 | 140453103 | 140453221 | 1 | Pool1 |
| CHP2_BRAF_2 | CCACAAAATGGATCCAGACAACTGT | GCTTGCTCTGATAGGAAAATGAGATCTA | GENOME_REGION | COSM30730 | hg19 | chr7 | 140453078 | 1,4E+08 | 140453103 | 140453221 | 1 | Pool1 |
| CHP2_BRAF_2 | CCACAAAATGGATCCAGACAACTGT | GCTTGCTCTGATAGGAAAATGAGATCTA | GENOME_REGION | COSM33729 | hg19 | chr7 | 140453078 | 1,4E+08 | 140453103 | 140453221 | 1 | Pool1 |
| CHP2_BRAF_2 | CCACAAAATGGATCCAGACAACTGT | GCTTGCTCTGATAGGAAAATGAGATCTA | GENOME_REGION | COSM33808 | hg19 | chr7 | 140453078 | 1,4E+08 | 140453103 | 140453221 | 1 | Pool1 |
| CHP2_BRAF_2 | CCACAAAATGGATCCAGACAACTGT | GCTTGCTCTGATAGGAAAATGAGATCTA | GENOME_REGION | COSM462 | hg19 | chr7 | 140453078 | 1,4E+08 | 140453103 | 140453221 | 1 | Pool1 |
| CHP2_BRAF_2 | CCACAAAATGGATCCAGACAACTGT | GCTTGCTCTGATAGGAAAATGAGATCTA | GENOME_REGION | COSM463 | hg19 | chr7 | 140453078 | 1,4E+08 | 140453103 | 140453221 | 1 | Pool1 |
| CHP2_BRAF_2 | CCACAAAATGGATCCAGACAACTGT | GCTTGCTCTGATAGGAAAATGAGATCTA | GENOME_REGION | COSM466 | hg19 | chr7 | 140453078 | 1,4E+08 | 140453103 | 140453221 | 1 | Pool1 |
| CHP2_BRAF_2 | CCACAAAATGGATCCAGACAACTGT | GCTTGCTCTGATAGGAAAATGAGATCTA | GENOME_REGION | COSM467 | hg19 | chr7 | 140453078 | 1,4E+08 | 140453103 | 140453221 | 1 | Pool1 |
| CHP2_BRAF_2 | CCACAAAATGGATCCAGACAACTGT | GCTTGCTCTGATAGGAAAATGAGATCTA | GENOME_REGION | COSM468 | hg19 | chr7 | 140453078 | 1,4E+08 | 140453103 | 140453221 | 1 | Pool1 |
| CHP2_BRAF_2 | CCACAAAATGGATCCAGACAACTGT | GCTTGCTCTGATAGGAAAATGAGATCTA | GENOME_REGION | COSM469 | hg19 | chr7 | 140453078 | 1,4E+08 | 140453103 | 140453221 | 1 | Pool1 |
| CHP2_BRAF_2 | CCACAAAATGGATCCAGACAACTGT | GCTTGCTCTGATAGGAAAATGAGATCTA | GENOME_REGION | COSM470 | hg19 | chr7 | 140453078 | 1,4E+08 | 140453103 | 140453221 | 1 | Pool1 |
| CHP2_BRAF_2 | CCACAAAATGGATCCAGACAACTGT | GCTTGCTCTGATAGGAAAATGAGATCTA | GENOME_REGION | COSM471 | hg19 | chr7 | 140453078 | 1,4E+08 | 140453103 | 140453221 | 1 | Pool1 |
| CHP2_BRAF_2 | CCACAAAATGGATCCAGACAACTGT | GCTTGCTCTGATAGGAAAATGAGATCTA | GENOME_REGION | COSM472 | hg19 | chr7 | 140453078 | 1,4E+08 | 140453103 | 140453221 | 1 | Pool1 |
| CHP2_BRAF_2 | CCACAAAATGGATCCAGACAACTGT | GCTTGCTCTGATAGGAAAATGAGATCTA | GENOME_REGION | COSM473 | hg19 | chr7 | 140453078 | 1,4E+08 | 140453103 | 140453221 | 1 | Pool1 |
| CHP2_BRAF_2 | CCACAAAATGGATCCAGACAACTGT | GCTTGCTCTGATAGGAAAATGAGATCTA | GENOME_REGION | COSM474 | hg19 | chr7 | 140453078 | 1,4E+08 | 140453103 | 140453221 | 1 | Pool1 |
| CHP2_BRAF_2 | CCACAAAATGGATCCAGACAACTGT | GCTTGCTCTGATAGGAAAATGAGATCTA | GENOME_REGION | COSM475 | hg19 | chr7 | 140453078 | 1,4E+08 | 140453103 | 140453221 | 1 | Pool1 |
| CHP2_BRAF_2 | CCACAAAATGGATCCAGACAACTGT | GCTTGCTCTGATAGGAAAATGAGATCTA | GENOME_REGION | COSM476 | hg19 | chr7 | 140453078 | 1,4E+08 | 140453103 | 140453221 | 1 | Pool1 |
| CHP2_BRAF_2 | CCACAAAATGGATCCAGACAACTGT | GCTTGCTCTGATAGGAAAATGAGATCTA | GENOME_REGION | COSM477 | hg19 | chr7 | 140453078 | 1,4E+08 | 140453103 | 140453221 | 1 | Pool1 |
| CHP2_BRAF_2 | CCACAAAATGGATCCAGACAACTGT | GCTTGCTCTGATAGGAAAATGAGATCTA | GENOME_REGION | COSM478 | hg19 | chr7 | 140453078 | 1,4E+08 | 140453103 | 140453221 | 1 | Pool1 |
| CHP2_BRAF_2 | CCACAAAATGGATCCAGACAACTGT | GCTTGCTCTGATAGGAAAATGAGATCTA | GENOME_REGION | COSM6137 | hg19 | chr7 | 140453078 | 1,4E+08 | 140453103 | 140453221 | 1 | Pool1 |
| CHP2_BRAF_2 | CCACAAAATGGATCCAGACAACTGT | GCTTGCTCTGATAGGAAAATGAGATCTA | GENOME_REGION | COSM6265 | hg19 | chr7 | 140453078 | 1,4E+08 | 140453103 | 140453221 | 1 | Pool1 |
| CHP2_BRAF_2 | CCACAAAATGGATCCAGACAACTGT | GCTTGCTCTGATAGGAAAATGAGATCTA | GENOME_REGION | COSM6267 | hg19 | chr7 | 140453078 | 1,4E+08 | 140453103 | 140453221 | 1 | Pool1 |
| CHP2_CTNNB1_1 | ACTGTTTCGTATTTATAGCTGATTTGATGGA | CCTCTTCCTCAGGATTGCCTTT | GENOME_REGION | COSM13168 | hg19 | chr3 | 41265999 | 4,1E+07 | 41266030 | 41266147 | 1 | Pool1 |
| CHP2_CTNNB1_1 | ACTGTTTCGTATTTATAGCTGATTTGATGGA | CCTCTTCCTCAGGATTGCCTTT | GENOME_REGION | COSM13175 | hg19 | chr3 | 41265999 | 4,1E+07 | 41266030 | 41266147 | 1 | Pool1 |
| CHP2_CTNNB1_1 | ACTGTTTCGTATTTATAGCTGATTTGATGGA | CCTCTTCCTCAGGATTGCCTTT | GENOME_REGION | COSM14256 | hg19 | chr3 | 41265999 | 4,1E+07 | 41266030 | 41266147 | 1 | Pool1 |
| CHP2_CTNNB1_1 | ACTGTTTCGTATTTATAGCTGATTTGATGGA | CCTCTTCCTCAGGATTGCCTTT | GENOME_REGION | COSM17661 | hg19 | chr3 | 41265999 | 4,1E+07 | 41266030 | 41266147 | 1 | Pool1 |
| CHP2_CTNNB1_1 | ACTGTTTCGTATTTATAGCTGATTTGATGGA | CCTCTTCCTCAGGATTGCCTTT | GENOME_REGION | COSM17941 | hg19 | chr3 | 41265999 | 4,1E+07 | 41266030 | 41266147 | 1 | Pool1 |
| CHP2_CTNNB1_1 | ACTGTTTCGTATTTATAGCTGATTTGATGGA | CCTCTTCCTCAGGATTGCCTTT | GENOME_REGION | COSM22566 | hg19 | chr3 | 41265999 | 4,1E+07 | 41266030 | 41266147 | 1 | Pool1 |
| CHP2_CTNNB1_1 | ACTGTTTCGTATTTATAGCTGATTTGATGGA | CCTCTTCCTCAGGATTGCCTTT | GENOME_REGION | COSM27378 | hg19 | chr3 | 41265999 | 4,1E+07 | 41266030 | 41266147 | 1 | Pool1 |
| CHP2_CTNNB1_1 | ACTGTTTCGTATTTATAGCTGATTTGATGGA | CCTCTTCCTCAGGATTGCCTTT | GENOME_REGION | COSM29289 | hg19 | chr3 | 41265999 | 4,1E+07 | 41266030 | 41266147 | 1 | Pool1 |
| CHP2_CTNNB1_1 | ACTGTTTCGTATTTATAGCTGATTTGATGGA | CCTCTTCCTCAGGATTGCCTTT | GENOME_REGION | COSM34125 | hg19 | chr3 | 41265999 | 4,1E+07 | 41266030 | 41266147 | 1 | Pool1 |
| CHP2_CTNNB1_1 | ACTGTTTCGTATTTATAGCTGATTTGATGGA | CCTCTTCCTCAGGATTGCCTTT | GENOME_REGION | COSM49161 | hg19 | chr3 | 41265999 | 4,1E+07 | 41266030 | 41266147 | 1 | Pool1 |
| CHP2_CTNNB1_1 | ACTGTTTCGTATTTATAGCTGATTTGATGGA | CCTCTTCCTCAGGATTGCCTTT | GENOME_REGION | COSM5661 | hg19 | chr3 | 41265999 | 4,1E+07 | 41266030 | 41266147 | 1 | Pool1 |
| CHP2_CTNNB1_1 | ACTGTTTCGTATTTATAGCTGATTTGATGGA | CCTCTTCCTCAGGATTGCCTTT | GENOME_REGION | COSM5662 | hg19 | chr3 | 41265999 | 4,1E+07 | 41266030 | 41266147 | 1 | Pool1 |
| CHP2_CTNNB1_1 | ACTGTTTCGTATTTATAGCTGATTTGATGGA | CCTCTTCCTCAGGATTGCCTTT | GENOME_REGION | COSM5663 | hg19 | chr3 | 41265999 | 4,1E+07 | 41266030 | 41266147 | 1 | Pool1 |
| CHP2_CTNNB1_1 | ACTGTTTCGTATTTATAGCTGATTTGATGGA | CCTCTTCCTCAGGATTGCCTTT | GENOME_REGION | COSM5664 | hg19 | chr3 | 41265999 | 4,1E+07 | 41266030 | 41266147 | 1 | Pool1 |
| CHP2_CTNNB1_1 | ACTGTTTCGTATTTATAGCTGATTTGATGGA | CCTCTTCCTCAGGATTGCCTTT | GENOME_REGION | COSM5666 | hg19 | chr3 | 41265999 | 4,1E+07 | 41266030 | 41266147 | 1 | Pool1 |
| CHP2_CTNNB1_1 | ACTGTTTCGTATTTATAGCTGATTTGATGGA | CCTCTTCCTCAGGATTGCCTTT | GENOME_REGION | COSM5667 | hg19 | chr3 | 41265999 | 4,1E+07 | 41266030 | 41266147 | 1 | Pool1 |
| CHP2_CTNNB1_1 | ACTGTTTCGTATTTATAGCTGATTTGATGGA | CCTCTTCCTCAGGATTGCCTTT | GENOME_REGION | COSM5668 | hg19 | chr3 | 41265999 | 4,1E+07 | 41266030 | 41266147 | 1 | Pool1 |
| CHP2_CTNNB1_1 | ACTGTTTCGTATTTATAGCTGATTTGATGGA | CCTCTTCCTCAGGATTGCCTTT | GENOME_REGION | COSM5669 | hg19 | chr3 | 41265999 | 4,1E+07 | 41266030 | 41266147 | 1 | Pool1 |
| CHP2_CTNNB1_1 | ACTGTTTCGTATTTATAGCTGATTTGATGGA | CCTCTTCCTCAGGATTGCCTTT | GENOME_REGION | COSM5670 | hg19 | chr3 | 41265999 | 4,1E+07 | 41266030 | 41266147 | 1 | Pool1 |
| CHP2_CTNNB1_1 | ACTGTTTCGTATTTATAGCTGATTTGATGGA | CCTCTTCCTCAGGATTGCCTTT | GENOME_REGION | COSM5671 | hg19 | chr3 | 41265999 | 4,1E+07 | 41266030 | 41266147 | 1 | Pool1 |
| CHP2_CTNNB1_1 | ACTGTTTCGTATTTATAGCTGATTTGATGGA | CCTCTTCCTCAGGATTGCCTTT | GENOME_REGION | COSM5672 | hg19 | chr3 | 41265999 | 4,1E+07 | 41266030 | 41266147 | 1 | Pool1 |
| CHP2_CTNNB1_1 | ACTGTTTCGTATTTATAGCTGATTTGATGGA | CCTCTTCCTCAGGATTGCCTTT | GENOME_REGION | COSM5673 | hg19 | chr3 | 41265999 | 4,1E+07 | 41266030 | 41266147 | 1 | Pool1 |
| CHP2_CTNNB1_1 | ACTGTTTCGTATTTATAGCTGATTTGATGGA | CCTCTTCCTCAGGATTGCCTTT | GENOME_REGION | COSM5674 | hg19 | chr3 | 41265999 | 4,1E+07 | 41266030 | 41266147 | 1 | Pool1 |
| CHP2_CTNNB1_1 | ACTGTTTCGTATTTATAGCTGATTTGATGGA | CCTCTTCCTCAGGATTGCCTTT | GENOME_REGION | COSM5675 | hg19 | chr3 | 41265999 | 4,1E+07 | 41266030 | 41266147 | 1 | Pool1 |
| CHP2_CTNNB1_1 | ACTGTTTCGTATTTATAGCTGATTTGATGGA | CCTCTTCCTCAGGATTGCCTTT | GENOME_REGION | COSM5676 | hg19 | chr3 | 41265999 | 4,1E+07 | 41266030 | 41266147 | 1 | Pool1 |
| CHP2_CTNNB1_1 | ACTGTTTCGTATTTATAGCTGATTTGATGGA | CCTCTTCCTCAGGATTGCCTTT | GENOME_REGION | COSM5677 | hg19 | chr3 | 41265999 | 4,1E+07 | 41266030 | 41266147 | 1 | Pool1 |
| CHP2_CTNNB1_1 | ACTGTTTCGTATTTATAGCTGATTTGATGGA | CCTCTTCCTCAGGATTGCCTTT | GENOME_REGION | COSM5678 | hg19 | chr3 | 41265999 | 4,1E+07 | 41266030 | 41266147 | 1 | Pool1 |
| CHP2_CTNNB1_1 | ACTGTTTCGTATTTATAGCTGATTTGATGGA | CCTCTTCCTCAGGATTGCCTTT | GENOME_REGION | COSM5679 | hg19 | chr3 | 41265999 | 4,1E+07 | 41266030 | 41266147 | 1 | Pool1 |
| CHP2_CTNNB1_1 | ACTGTTTCGTATTTATAGCTGATTTGATGGA | CCTCTTCCTCAGGATTGCCTTT | GENOME_REGION | COSM5681 | hg19 | chr3 | 41265999 | 4,1E+07 | 41266030 | 41266147 | 1 | Pool1 |
| CHP2_CTNNB1_1 | ACTGTTTCGTATTTATAGCTGATTTGATGGA | CCTCTTCCTCAGGATTGCCTTT | GENOME_REGION | COSM5682 | hg19 | chr3 | 41265999 | 4,1E+07 | 41266030 | 41266147 | 1 | Pool1 |
| CHP2_CTNNB1_1 | ACTGTTTCGTATTTATAGCTGATTTGATGGA | CCTCTTCCTCAGGATTGCCTTT | GENOME_REGION | COSM5683 | hg19 | chr3 | 41265999 | 4,1E+07 | 41266030 | 41266147 | 1 | Pool1 |
| CHP2_CTNNB1_1 | ACTGTTTCGTATTTATAGCTGATTTGATGGA | CCTCTTCCTCAGGATTGCCTTT | GENOME_REGION | COSM5684 | hg19 | chr3 | 41265999 | 4,1E+07 | 41266030 | 41266147 | 1 | Pool1 |
| CHP2_CTNNB1_1 | ACTGTTTCGTATTTATAGCTGATTTGATGGA | CCTCTTCCTCAGGATTGCCTTT | GENOME_REGION | COSM5685 | hg19 | chr3 | 41265999 | 4,1E+07 | 41266030 | 41266147 | 1 | Pool1 |
| CHP2_CTNNB1_1 | ACTGTTTCGTATTTATAGCTGATTTGATGGA | CCTCTTCCTCAGGATTGCCTTT | GENOME_REGION | COSM5686 | hg19 | chr3 | 41265999 | 4,1E+07 | 41266030 | 41266147 | 1 | Pool1 |
| CHP2_CTNNB1_1 | ACTGTTTCGTATTTATAGCTGATTTGATGGA | CCTCTTCCTCAGGATTGCCTTT | GENOME_REGION | COSM5687 | hg19 | chr3 | 41265999 | 4,1E+07 | 41266030 | 41266147 | 1 | Pool1 |
| CHP2_CTNNB1_1 | ACTGTTTCGTATTTATAGCTGATTTGATGGA | CCTCTTCCTCAGGATTGCCTTT | GENOME_REGION | COSM5688 | hg19 | chr3 | 41265999 | 4,1E+07 | 41266030 | 41266147 | 1 | Pool1 |
| CHP2_CTNNB1_1 | ACTGTTTCGTATTTATAGCTGATTTGATGGA | CCTCTTCCTCAGGATTGCCTTT | GENOME_REGION | COSM5689 | hg19 | chr3 | 41265999 | 4,1E+07 | 41266030 | 41266147 | 1 | Pool1 |
| CHP2_CTNNB1_1 | ACTGTTTCGTATTTATAGCTGATTTGATGGA | CCTCTTCCTCAGGATTGCCTTT | GENOME_REGION | COSM5690 | hg19 | chr3 | 41265999 | 4,1E+07 | 41266030 | 41266147 | 1 | Pool1 |
| CHP2_CTNNB1_1 | ACTGTTTCGTATTTATAGCTGATTTGATGGA | CCTCTTCCTCAGGATTGCCTTT | GENOME_REGION | COSM5691 | hg19 | chr3 | 41265999 | 4,1E+07 | 41266030 | 41266147 | 1 | Pool1 |
| CHP2_CTNNB1_1 | ACTGTTTCGTATTTATAGCTGATTTGATGGA | CCTCTTCCTCAGGATTGCCTTT | GENOME_REGION | COSM5692 | hg19 | chr3 | 41265999 | 4,1E+07 | 41266030 | 41266147 | 1 | Pool1 |
| CHP2_CTNNB1_1 | ACTGTTTCGTATTTATAGCTGATTTGATGGA | CCTCTTCCTCAGGATTGCCTTT | GENOME_REGION | COSM5694 | hg19 | chr3 | 41265999 | 4,1E+07 | 41266030 | 41266147 | 1 | Pool1 |
| CHP2_CTNNB1_1 | ACTGTTTCGTATTTATAGCTGATTTGATGGA | CCTCTTCCTCAGGATTGCCTTT | GENOME_REGION | COSM5696 | hg19 | chr3 | 41265999 | 4,1E+07 | 41266030 | 41266147 | 1 | Pool1 |
| CHP2_CTNNB1_1 | ACTGTTTCGTATTTATAGCTGATTTGATGGA | CCTCTTCCTCAGGATTGCCTTT | GENOME_REGION | COSM5699 | hg19 | chr3 | 41265999 | 4,1E+07 | 41266030 | 41266147 | 1 | Pool1 |
| CHP2_CTNNB1_1 | ACTGTTTCGTATTTATAGCTGATTTGATGGA | CCTCTTCCTCAGGATTGCCTTT | GENOME_REGION | COSM5701 | hg19 | chr3 | 41265999 | 4,1E+07 | 41266030 | 41266147 | 1 | Pool1 |
| CHP2_CTNNB1_1 | ACTGTTTCGTATTTATAGCTGATTTGATGGA | CCTCTTCCTCAGGATTGCCTTT | GENOME_REGION | COSM5702 | hg19 | chr3 | 41265999 | 4,1E+07 | 41266030 | 41266147 | 1 | Pool1 |
| CHP2_CTNNB1_1 | ACTGTTTCGTATTTATAGCTGATTTGATGGA | CCTCTTCCTCAGGATTGCCTTT | GENOME_REGION | COSM5703 | hg19 | chr3 | 41265999 | 4,1E+07 | 41266030 | 41266147 | 1 | Pool1 |
| CHP2_CTNNB1_1 | ACTGTTTCGTATTTATAGCTGATTTGATGGA | CCTCTTCCTCAGGATTGCCTTT | GENOME_REGION | COSM5704 | hg19 | chr3 | 41265999 | 4,1E+07 | 41266030 | 41266147 | 1 | Pool1 |
| CHP2_CTNNB1_1 | ACTGTTTCGTATTTATAGCTGATTTGATGGA | CCTCTTCCTCAGGATTGCCTTT | GENOME_REGION | COSM5706 | hg19 | chr3 | 41265999 | 4,1E+07 | 41266030 | 41266147 | 1 | Pool1 |
| CHP2_CTNNB1_1 | ACTGTTTCGTATTTATAGCTGATTTGATGGA | CCTCTTCCTCAGGATTGCCTTT | GENOME_REGION | COSM5708 | hg19 | chr3 | 41265999 | 4,1E+07 | 41266030 | 41266147 | 1 | Pool1 |
| CHP2_CTNNB1_1 | ACTGTTTCGTATTTATAGCTGATTTGATGGA | CCTCTTCCTCAGGATTGCCTTT | GENOME_REGION | COSM5713 | hg19 | chr3 | 41265999 | 4,1E+07 | 41266030 | 41266147 | 1 | Pool1 |
| CHP2_CTNNB1_1 | ACTGTTTCGTATTTATAGCTGATTTGATGGA | CCTCTTCCTCAGGATTGCCTTT | GENOME_REGION | COSM5714 | hg19 | chr3 | 41265999 | 4,1E+07 | 41266030 | 41266147 | 1 | Pool1 |
| CHP2_CTNNB1_1 | ACTGTTTCGTATTTATAGCTGATTTGATGGA | CCTCTTCCTCAGGATTGCCTTT | GENOME_REGION | COSM5716 | hg19 | chr3 | 41265999 | 4,1E+07 | 41266030 | 41266147 | 1 | Pool1 |
| CHP2_CTNNB1_1 | ACTGTTTCGTATTTATAGCTGATTTGATGGA | CCTCTTCCTCAGGATTGCCTTT | GENOME_REGION | COSM5717 | hg19 | chr3 | 41265999 | 4,1E+07 | 41266030 | 41266147 | 1 | Pool1 |
| CHP2_CTNNB1_1 | ACTGTTTCGTATTTATAGCTGATTTGATGGA | CCTCTTCCTCAGGATTGCCTTT | GENOME_REGION | COSM5721 | hg19 | chr3 | 41265999 | 4,1E+07 | 41266030 | 41266147 | 1 | Pool1 |
| CHP2_CTNNB1_1 | ACTGTTTCGTATTTATAGCTGATTTGATGGA | CCTCTTCCTCAGGATTGCCTTT | GENOME_REGION | COSM5730 | hg19 | chr3 | 41265999 | 4,1E+07 | 41266030 | 41266147 | 1 | Pool1 |
| CHP2_CTNNB1_1 | ACTGTTTCGTATTTATAGCTGATTTGATGGA | CCTCTTCCTCAGGATTGCCTTT | GENOME_REGION | COSM5732 | hg19 | chr3 | 41265999 | 4,1E+07 | 41266030 | 41266147 | 1 | Pool1 |
| CHP2_CTNNB1_1 | ACTGTTTCGTATTTATAGCTGATTTGATGGA | CCTCTTCCTCAGGATTGCCTTT | GENOME_REGION | COSM5738 | hg19 | chr3 | 41265999 | 4,1E+07 | 41266030 | 41266147 | 1 | Pool1 |
| CHP2_CTNNB1_1 | ACTGTTTCGTATTTATAGCTGATTTGATGGA | CCTCTTCCTCAGGATTGCCTTT | GENOME_REGION | COSM5744 | hg19 | chr3 | 41265999 | 4,1E+07 | 41266030 | 41266147 | 1 | Pool1 |
| CHP2_CTNNB1_1 | ACTGTTTCGTATTTATAGCTGATTTGATGGA | CCTCTTCCTCAGGATTGCCTTT | GENOME_REGION | COSM5747 | hg19 | chr3 | 41265999 | 4,1E+07 | 41266030 | 41266147 | 1 | Pool1 |
| CHP2_CTNNB1_1 | ACTGTTTCGTATTTATAGCTGATTTGATGGA | CCTCTTCCTCAGGATTGCCTTT | GENOME_REGION | COSM5749 | hg19 | chr3 | 41265999 | 4,1E+07 | 41266030 | 41266147 | 1 | Pool1 |
| CHP2_CTNNB1_1 | ACTGTTTCGTATTTATAGCTGATTTGATGGA | CCTCTTCCTCAGGATTGCCTTT | GENOME_REGION | COSM5753 | hg19 | chr3 | 41265999 | 4,1E+07 | 41266030 | 41266147 | 1 | Pool1 |
| CHP2_CTNNB1_1 | ACTGTTTCGTATTTATAGCTGATTTGATGGA | CCTCTTCCTCAGGATTGCCTTT | GENOME_REGION | COSM5758 | hg19 | chr3 | 41265999 | 4,1E+07 | 41266030 | 41266147 | 1 | Pool1 |
| CHP2_CTNNB1_1 | ACTGTTTCGTATTTATAGCTGATTTGATGGA | CCTCTTCCTCAGGATTGCCTTT | GENOME_REGION | COSM5761 | hg19 | chr3 | 41265999 | 4,1E+07 | 41266030 | 41266147 | 1 | Pool1 |
| CHP2_CTNNB1_1 | ACTGTTTCGTATTTATAGCTGATTTGATGGA | CCTCTTCCTCAGGATTGCCTTT | GENOME_REGION | COSM5762 | hg19 | chr3 | 41265999 | 4,1E+07 | 41266030 | 41266147 | 1 | Pool1 |
| CHP2_CTNNB1_1 | ACTGTTTCGTATTTATAGCTGATTTGATGGA | CCTCTTCCTCAGGATTGCCTTT | GENOME_REGION | COSM6050 | hg19 | chr3 | 41265999 | 4,1E+07 | 41266030 | 41266147 | 1 | Pool1 |
| CHP2_CTNNB1_1 | ACTGTTTCGTATTTATAGCTGATTTGATGGA | CCTCTTCCTCAGGATTGCCTTT | GENOME_REGION | COSM6052 | hg19 | chr3 | 41265999 | 4,1E+07 | 41266030 | 41266147 | 1 | Pool1 |
| CHP2_CTNNB1_1 | ACTGTTTCGTATTTATAGCTGATTTGATGGA | CCTCTTCCTCAGGATTGCCTTT | GENOME_REGION | COSM6057 | hg19 | chr3 | 41265999 | 4,1E+07 | 41266030 | 41266147 | 1 | Pool1 |
| CHP2_CTNNB1_1 | ACTGTTTCGTATTTATAGCTGATTTGATGGA | CCTCTTCCTCAGGATTGCCTTT | GENOME_REGION | COSM6064 | hg19 | chr3 | 41265999 | 4,1E+07 | 41266030 | 41266147 | 1 | Pool1 |
| CHP2_CTNNB1_1 | ACTGTTTCGTATTTATAGCTGATTTGATGGA | CCTCTTCCTCAGGATTGCCTTT | GENOME_REGION | COSM6076 | hg19 | chr3 | 41265999 | 4,1E+07 | 41266030 | 41266147 | 1 | Pool1 |
| CHP2_CTNNB1_1 | ACTGTTTCGTATTTATAGCTGATTTGATGGA | CCTCTTCCTCAGGATTGCCTTT | GENOME_REGION | COSM6098 | hg19 | chr3 | 41265999 | 4,1E+07 | 41266030 | 41266147 | 1 | Pool1 |
| CHP2_CTNNB1_1 | ACTGTTTCGTATTTATAGCTGATTTGATGGA | CCTCTTCCTCAGGATTGCCTTT | GENOME_REGION | COSM6099 | hg19 | chr3 | 41265999 | 4,1E+07 | 41266030 | 41266147 | 1 | Pool1 |
| CHP2_CTNNB1_1 | ACTGTTTCGTATTTATAGCTGATTTGATGGA | CCTCTTCCTCAGGATTGCCTTT | GENOME_REGION | COSM6140 | hg19 | chr3 | 41265999 | 4,1E+07 | 41266030 | 41266147 | 1 | Pool1 |
| CHP2_EGFR_4 | TGTGGAGCCTCTTACACCCA | GTGCCAGGGACCTTACCTTATAC | GENOME_REGION | COSM12371 | hg19 | chr7 | 55241616 | 5,5E+07 | 55241636 | 55241729 | 1 | Pool1 |
| CHP2_EGFR_4 | TGTGGAGCCTCTTACACCCA | GTGCCAGGGACCTTACCTTATAC | GENOME_REGION | COSM12373 | hg19 | chr7 | 55241616 | 5,5E+07 | 55241636 | 55241729 | 1 | Pool1 |
| CHP2_EGFR_4 | TGTGGAGCCTCTTACACCCA | GTGCCAGGGACCTTACCTTATAC | GENOME_REGION | COSM12988 | hg19 | chr7 | 55241616 | 5,5E+07 | 55241636 | 55241729 | 1 | Pool1 |
| CHP2_EGFR_4 | TGTGGAGCCTCTTACACCCA | GTGCCAGGGACCTTACCTTATAC | GENOME_REGION | COSM13009 | hg19 | chr7 | 55241616 | 5,5E+07 | 55241636 | 55241729 | 1 | Pool1 |
| CHP2_EGFR_4 | TGTGGAGCCTCTTACACCCA | GTGCCAGGGACCTTACCTTATAC | GENOME_REGION | COSM13427 | hg19 | chr7 | 55241616 | 5,5E+07 | 55241636 | 55241729 | 1 | Pool1 |
| CHP2_EGFR_4 | TGTGGAGCCTCTTACACCCA | GTGCCAGGGACCTTACCTTATAC | GENOME_REGION | COSM13979 | hg19 | chr7 | 55241616 | 5,5E+07 | 55241636 | 55241729 | 1 | Pool1 |
| CHP2_EGFR_4 | TGTGGAGCCTCTTACACCCA | GTGCCAGGGACCTTACCTTATAC | GENOME_REGION | COSM18425 | hg19 | chr7 | 55241616 | 5,5E+07 | 55241636 | 55241729 | 1 | Pool1 |
| CHP2_EGFR_4 | TGTGGAGCCTCTTACACCCA | GTGCCAGGGACCTTACCTTATAC | GENOME_REGION | COSM18441 | hg19 | chr7 | 55241616 | 5,5E+07 | 55241636 | 55241729 | 1 | Pool1 |
| CHP2_EGFR_4 | TGTGGAGCCTCTTACACCCA | GTGCCAGGGACCTTACCTTATAC | GENOME_REGION | COSM22992 | hg19 | chr7 | 55241616 | 5,5E+07 | 55241636 | 55241729 | 1 | Pool1 |
| CHP2_EGFR_4 | TGTGGAGCCTCTTACACCCA | GTGCCAGGGACCTTACCTTATAC | GENOME_REGION | COSM28508 | hg19 | chr7 | 55241616 | 5,5E+07 | 55241636 | 55241729 | 1 | Pool1 |
| CHP2_EGFR_4 | TGTGGAGCCTCTTACACCCA | GTGCCAGGGACCTTACCTTATAC | GENOME_REGION | COSM28510 | hg19 | chr7 | 55241616 | 5,5E+07 | 55241636 | 55241729 | 1 | Pool1 |
| CHP2_EGFR_4 | TGTGGAGCCTCTTACACCCA | GTGCCAGGGACCTTACCTTATAC | GENOME_REGION | COSM28511 | hg19 | chr7 | 55241616 | 5,5E+07 | 55241636 | 55241729 | 1 | Pool1 |
| CHP2_EGFR_4 | TGTGGAGCCTCTTACACCCA | GTGCCAGGGACCTTACCTTATAC | GENOME_REGION | COSM28601 | hg19 | chr7 | 55241616 | 5,5E+07 | 55241636 | 55241729 | 1 | Pool1 |
| CHP2_EGFR_4 | TGTGGAGCCTCTTACACCCA | GTGCCAGGGACCTTACCTTATAC | GENOME_REGION | COSM41603 | hg19 | chr7 | 55241616 | 5,5E+07 | 55241636 | 55241729 | 1 | Pool1 |
| CHP2_EGFR_4 | TGTGGAGCCTCTTACACCCA | GTGCCAGGGACCTTACCTTATAC | GENOME_REGION | COSM41905 | hg19 | chr7 | 55241616 | 5,5E+07 | 55241636 | 55241729 | 1 | Pool1 |
| CHP2_EGFR_4 | TGTGGAGCCTCTTACACCCA | GTGCCAGGGACCTTACCTTATAC | GENOME_REGION | COSM6239 | hg19 | chr7 | 55241616 | 5,5E+07 | 55241636 | 55241729 | 1 | Pool1 |
| CHP2_EGFR_4 | TGTGGAGCCTCTTACACCCA | GTGCCAGGGACCTTACCTTATAC | GENOME_REGION | COSM6252 | hg19 | chr7 | 55241616 | 5,5E+07 | 55241636 | 55241729 | 1 | Pool1 |
| CHP2_EGFR_4 | TGTGGAGCCTCTTACACCCA | GTGCCAGGGACCTTACCTTATAC | GENOME_REGION | COSM6253 | hg19 | chr7 | 55241616 | 5,5E+07 | 55241636 | 55241729 | 1 | Pool1 |
| CHP2_EGFR_5 | ACGTCTTCCTTCTCTCTCTGTCA | CTGAGGTTCAGAGCCATGGA | GENOME_REGION | COSM12367 | hg19 | chr7 | 55242389 | 5,5E+07 | 55242412 | 55242540 | 1 | Pool1 |
| CHP2_EGFR_5 | ACGTCTTCCTTCTCTCTCTGTCA | CTGAGGTTCAGAGCCATGGA | GENOME_REGION | COSM12369 | hg19 | chr7 | 55242389 | 5,5E+07 | 55242412 | 55242540 | 1 | Pool1 |
| CHP2_EGFR_5 | ACGTCTTCCTTCTCTCTCTGTCA | CTGAGGTTCAGAGCCATGGA | GENOME_REGION | COSM12370 | hg19 | chr7 | 55242389 | 5,5E+07 | 55242412 | 55242540 | 1 | Pool1 |
| CHP2_EGFR_5 | ACGTCTTCCTTCTCTCTCTGTCA | CTGAGGTTCAGAGCCATGGA | GENOME_REGION | COSM12382 | hg19 | chr7 | 55242389 | 5,5E+07 | 55242412 | 55242540 | 1 | Pool1 |
| CHP2_EGFR_5 | ACGTCTTCCTTCTCTCTCTGTCA | CTGAGGTTCAGAGCCATGGA | GENOME_REGION | COSM12383 | hg19 | chr7 | 55242389 | 5,5E+07 | 55242412 | 55242540 | 1 | Pool1 |
| CHP2_EGFR_5 | ACGTCTTCCTTCTCTCTCTGTCA | CTGAGGTTCAGAGCCATGGA | GENOME_REGION | COSM12384 | hg19 | chr7 | 55242389 | 5,5E+07 | 55242412 | 55242540 | 1 | Pool1 |
| CHP2_EGFR_5 | ACGTCTTCCTTCTCTCTCTGTCA | CTGAGGTTCAGAGCCATGGA | GENOME_REGION | COSM12386 | hg19 | chr7 | 55242389 | 5,5E+07 | 55242412 | 55242540 | 1 | Pool1 |
| CHP2_EGFR_5 | ACGTCTTCCTTCTCTCTCTGTCA | CTGAGGTTCAGAGCCATGGA | GENOME_REGION | COSM12678 | hg19 | chr7 | 55242389 | 5,5E+07 | 55242412 | 55242540 | 1 | Pool1 |
| CHP2_EGFR_5 | ACGTCTTCCTTCTCTCTCTGTCA | CTGAGGTTCAGAGCCATGGA | GENOME_REGION | COSM12728 | hg19 | chr7 | 55242389 | 5,5E+07 | 55242412 | 55242540 | 1 | Pool1 |
| CHP2_EGFR_5 | ACGTCTTCCTTCTCTCTCTGTCA | CTGAGGTTCAGAGCCATGGA | GENOME_REGION | COSM13181 | hg19 | chr7 | 55242389 | 5,5E+07 | 55242412 | 55242540 | 1 | Pool1 |
| CHP2_EGFR_5 | ACGTCTTCCTTCTCTCTCTGTCA | CTGAGGTTCAGAGCCATGGA | GENOME_REGION | COSM13182 | hg19 | chr7 | 55242389 | 5,5E+07 | 55242412 | 55242540 | 1 | Pool1 |
| CHP2_EGFR_5 | ACGTCTTCCTTCTCTCTCTGTCA | CTGAGGTTCAGAGCCATGGA | GENOME_REGION | COSM13184 | hg19 | chr7 | 55242389 | 5,5E+07 | 55242412 | 55242540 | 1 | Pool1 |
| CHP2_EGFR_5 | ACGTCTTCCTTCTCTCTCTGTCA | CTGAGGTTCAGAGCCATGGA | GENOME_REGION | COSM13185 | hg19 | chr7 | 55242389 | 5,5E+07 | 55242412 | 55242540 | 1 | Pool1 |
| CHP2_EGFR_5 | ACGTCTTCCTTCTCTCTCTGTCA | CTGAGGTTCAGAGCCATGGA | GENOME_REGION | COSM133189 | hg19 | chr7 | 55242389 | 5,5E+07 | 55242412 | 55242540 | 1 | Pool1 |
| CHP2_EGFR_5 | ACGTCTTCCTTCTCTCTCTGTCA | CTGAGGTTCAGAGCCATGGA | GENOME_REGION | COSM133197 | hg19 | chr7 | 55242389 | 5,5E+07 | 55242412 | 55242540 | 1 | Pool1 |
| CHP2_EGFR_5 | ACGTCTTCCTTCTCTCTCTGTCA | CTGAGGTTCAGAGCCATGGA | GENOME_REGION | COSM133207 | hg19 | chr7 | 55242389 | 5,5E+07 | 55242412 | 55242540 | 1 | Pool1 |
| CHP2_EGFR_5 | ACGTCTTCCTTCTCTCTCTGTCA | CTGAGGTTCAGAGCCATGGA | GENOME_REGION | COSM13432 | hg19 | chr7 | 55242389 | 5,5E+07 | 55242412 | 55242540 | 1 | Pool1 |
| CHP2_EGFR_5 | ACGTCTTCCTTCTCTCTCTGTCA | CTGAGGTTCAGAGCCATGGA | GENOME_REGION | COSM13556 | hg19 | chr7 | 55242389 | 5,5E+07 | 55242412 | 55242540 | 1 | Pool1 |
| CHP2_EGFR_5 | ACGTCTTCCTTCTCTCTCTGTCA | CTGAGGTTCAGAGCCATGGA | GENOME_REGION | COSM17570 | hg19 | chr7 | 55242389 | 5,5E+07 | 55242412 | 55242540 | 1 | Pool1 |
| CHP2_EGFR_5 | ACGTCTTCCTTCTCTCTCTGTCA | CTGAGGTTCAGAGCCATGGA | GENOME_REGION | COSM21984 | hg19 | chr7 | 55242389 | 5,5E+07 | 55242412 | 55242540 | 1 | Pool1 |
| CHP2_EGFR_5 | ACGTCTTCCTTCTCTCTCTGTCA | CTGAGGTTCAGAGCCATGGA | GENOME_REGION | COSM23571 | hg19 | chr7 | 55242389 | 5,5E+07 | 55242412 | 55242540 | 1 | Pool1 |
| CHP2_EGFR_5 | ACGTCTTCCTTCTCTCTCTGTCA | CTGAGGTTCAGAGCCATGGA | GENOME_REGION | COSM24267 | hg19 | chr7 | 55242389 | 5,5E+07 | 55242412 | 55242540 | 1 | Pool1 |
| CHP2_EGFR_5 | ACGTCTTCCTTCTCTCTCTGTCA | CTGAGGTTCAGAGCCATGGA | GENOME_REGION | COSM26038 | hg19 | chr7 | 55242389 | 5,5E+07 | 55242412 | 55242540 | 1 | Pool1 |
| CHP2_EGFR_5 | ACGTCTTCCTTCTCTCTCTGTCA | CTGAGGTTCAGAGCCATGGA | GENOME_REGION | COSM26509 | hg19 | chr7 | 55242389 | 5,5E+07 | 55242412 | 55242540 | 1 | Pool1 |
| CHP2_EGFR_5 | ACGTCTTCCTTCTCTCTCTGTCA | CTGAGGTTCAGAGCCATGGA | GENOME_REGION | COSM26704 | hg19 | chr7 | 55242389 | 5,5E+07 | 55242412 | 55242540 | 1 | Pool1 |
| CHP2_EGFR_5 | ACGTCTTCCTTCTCTCTCTGTCA | CTGAGGTTCAGAGCCATGGA | GENOME_REGION | COSM27041 | hg19 | chr7 | 55242389 | 5,5E+07 | 55242412 | 55242540 | 1 | Pool1 |
| CHP2_EGFR_5 | ACGTCTTCCTTCTCTCTCTGTCA | CTGAGGTTCAGAGCCATGGA | GENOME_REGION | COSM27042 | hg19 | chr7 | 55242389 | 5,5E+07 | 55242412 | 55242540 | 1 | Pool1 |
| CHP2_EGFR_5 | ACGTCTTCCTTCTCTCTCTGTCA | CTGAGGTTCAGAGCCATGGA | GENOME_REGION | COSM28517 | hg19 | chr7 | 55242389 | 5,5E+07 | 55242412 | 55242540 | 1 | Pool1 |
| CHP2_EGFR_5 | ACGTCTTCCTTCTCTCTCTGTCA | CTGAGGTTCAGAGCCATGGA | GENOME_REGION | COSM29274 | hg19 | chr7 | 55242389 | 5,5E+07 | 55242412 | 55242540 | 1 | Pool1 |
| CHP2_EGFR_5 | ACGTCTTCCTTCTCTCTCTGTCA | CTGAGGTTCAGAGCCATGGA | GENOME_REGION | COSM53194 | hg19 | chr7 | 55242389 | 5,5E+07 | 55242412 | 55242540 | 1 | Pool1 |
| CHP2_EGFR_5 | ACGTCTTCCTTCTCTCTCTGTCA | CTGAGGTTCAGAGCCATGGA | GENOME_REGION | COSM6210 | hg19 | chr7 | 55242389 | 5,5E+07 | 55242412 | 55242540 | 1 | Pool1 |
| CHP2_EGFR_5 | ACGTCTTCCTTCTCTCTCTGTCA | CTGAGGTTCAGAGCCATGGA | GENOME_REGION | COSM6218 | hg19 | chr7 | 55242389 | 5,5E+07 | 55242412 | 55242540 | 1 | Pool1 |
| CHP2_EGFR_5 | ACGTCTTCCTTCTCTCTCTGTCA | CTGAGGTTCAGAGCCATGGA | GENOME_REGION | COSM6220 | hg19 | chr7 | 55242389 | 5,5E+07 | 55242412 | 55242540 | 1 | Pool1 |
| CHP2_EGFR_5 | ACGTCTTCCTTCTCTCTCTGTCA | CTGAGGTTCAGAGCCATGGA | GENOME_REGION | COSM6223 | hg19 | chr7 | 55242389 | 5,5E+07 | 55242412 | 55242540 | 1 | Pool1 |
| CHP2_EGFR_5 | ACGTCTTCCTTCTCTCTCTGTCA | CTGAGGTTCAGAGCCATGGA | GENOME_REGION | COSM6225 | hg19 | chr7 | 55242389 | 5,5E+07 | 55242412 | 55242540 | 1 | Pool1 |
| CHP2_EGFR_5 | ACGTCTTCCTTCTCTCTCTGTCA | CTGAGGTTCAGAGCCATGGA | GENOME_REGION | COSM6254 | hg19 | chr7 | 55242389 | 5,5E+07 | 55242412 | 55242540 | 1 | Pool1 |
| CHP2_EGFR_5 | ACGTCTTCCTTCTCTCTCTGTCA | CTGAGGTTCAGAGCCATGGA | GENOME_REGION | COSM6255 | hg19 | chr7 | 55242389 | 5,5E+07 | 55242412 | 55242540 | 1 | Pool1 |
| CHP2_EGFR_5 | ACGTCTTCCTTCTCTCTCTGTCA | CTGAGGTTCAGAGCCATGGA | GENOME_REGION | COSM6256 | hg19 | chr7 | 55242389 | 5,5E+07 | 55242412 | 55242540 | 1 | Pool1 |
| CHP2_EGFR_5 | ACGTCTTCCTTCTCTCTCTGTCA | CTGAGGTTCAGAGCCATGGA | GENOME_REGION | COSM6268 | hg19 | chr7 | 55242389 | 5,5E+07 | 55242412 | 55242540 | 1 | Pool1 |
| CHP2_EGFR_5 | ACGTCTTCCTTCTCTCTCTGTCA | CTGAGGTTCAGAGCCATGGA | GENOME_REGION | COSM85993 | hg19 | chr7 | 55242389 | 5,5E+07 | 55242412 | 55242540 | 1 | Pool1 |
| CHP2_EGFR_5 | ACGTCTTCCTTCTCTCTCTGTCA | CTGAGGTTCAGAGCCATGGA | GENOME_REGION | COSM96856 | hg19 | chr7 | 55242389 | 5,5E+07 | 55242412 | 55242540 | 1 | Pool1 |
| CHP2_EGFR_8 | CGCAGCATGTCAAGATCACAGAT | GCATGTGTTAAACAATACAGCTAGTG | GENOME_REGION | COSM12366 | hg19 | chr7 | 55259485 | 5,5E+07 | 55259508 | 55259628 | 1 | Pool1 |
| CHP2_EGFR_8 | CGCAGCATGTCAAGATCACAGAT | GCATGTGTTAAACAATACAGCTAGTG | GENOME_REGION | COSM12374 | hg19 | chr7 | 55259485 | 5,5E+07 | 55259508 | 55259628 | 1 | Pool1 |
| CHP2_EGFR_8 | CGCAGCATGTCAAGATCACAGAT | GCATGTGTTAAACAATACAGCTAGTG | GENOME_REGION | COSM12429 | hg19 | chr7 | 55259485 | 5,5E+07 | 55259508 | 55259628 | 1 | Pool1 |
| CHP2_EGFR_8 | CGCAGCATGTCAAGATCACAGAT | GCATGTGTTAAACAATACAGCTAGTG | GENOME_REGION | COSM12675 | hg19 | chr7 | 55259485 | 5,5E+07 | 55259508 | 55259628 | 1 | Pool1 |
| CHP2_EGFR_8 | CGCAGCATGTCAAGATCACAGAT | GCATGTGTTAAACAATACAGCTAGTG | GENOME_REGION | COSM13008 | hg19 | chr7 | 55259485 | 5,5E+07 | 55259508 | 55259628 | 1 | Pool1 |
| CHP2_EGFR_8 | CGCAGCATGTCAAGATCACAGAT | GCATGTGTTAAACAATACAGCTAGTG | GENOME_REGION | COSM13197 | hg19 | chr7 | 55259485 | 5,5E+07 | 55259508 | 55259628 | 1 | Pool1 |
| CHP2_EGFR_8 | CGCAGCATGTCAAGATCACAGAT | GCATGTGTTAAACAATACAGCTAGTG | GENOME_REGION | COSM13199 | hg19 | chr7 | 55259485 | 5,5E+07 | 55259508 | 55259628 | 1 | Pool1 |
| CHP2_EGFR_8 | CGCAGCATGTCAAGATCACAGAT | GCATGTGTTAAACAATACAGCTAGTG | GENOME_REGION | COSM14070 | hg19 | chr7 | 55259485 | 5,5E+07 | 55259508 | 55259628 | 1 | Pool1 |
| CHP2_EGFR_8 | CGCAGCATGTCAAGATCACAGAT | GCATGTGTTAAACAATACAGCTAGTG | GENOME_REGION | COSM26129 | hg19 | chr7 | 55259485 | 5,5E+07 | 55259508 | 55259628 | 1 | Pool1 |
| CHP2_EGFR_8 | CGCAGCATGTCAAGATCACAGAT | GCATGTGTTAAACAATACAGCTAGTG | GENOME_REGION | COSM26438 | hg19 | chr7 | 55259485 | 5,5E+07 | 55259508 | 55259628 | 1 | Pool1 |
| CHP2_EGFR_8 | CGCAGCATGTCAAGATCACAGAT | GCATGTGTTAAACAATACAGCTAGTG | GENOME_REGION | COSM28605 | hg19 | chr7 | 55259485 | 5,5E+07 | 55259508 | 55259628 | 1 | Pool1 |
| CHP2_EGFR_8 | CGCAGCATGTCAAGATCACAGAT | GCATGTGTTAAACAATACAGCTAGTG | GENOME_REGION | COSM28607 | hg19 | chr7 | 55259485 | 5,5E+07 | 55259508 | 55259628 | 1 | Pool1 |
| CHP2_EGFR_8 | CGCAGCATGTCAAGATCACAGAT | GCATGTGTTAAACAATACAGCTAGTG | GENOME_REGION | COSM33725 | hg19 | chr7 | 55259485 | 5,5E+07 | 55259508 | 55259628 | 1 | Pool1 |
| CHP2_EGFR_8 | CGCAGCATGTCAAGATCACAGAT | GCATGTGTTAAACAATACAGCTAGTG | GENOME_REGION | COSM53292 | hg19 | chr7 | 55259485 | 5,5E+07 | 55259508 | 55259628 | 1 | Pool1 |
| CHP2_EGFR_8 | CGCAGCATGTCAAGATCACAGAT | GCATGTGTTAAACAATACAGCTAGTG | GENOME_REGION | COSM6213 | hg19 | chr7 | 55259485 | 5,5E+07 | 55259508 | 55259628 | 1 | Pool1 |
| CHP2_EGFR_8 | CGCAGCATGTCAAGATCACAGAT | GCATGTGTTAAACAATACAGCTAGTG | GENOME_REGION | COSM6224 | hg19 | chr7 | 55259485 | 5,5E+07 | 55259508 | 55259628 | 1 | Pool1 |
| CHP2_ERBB2_1 | GAATGTGAAAATTCCAGTGGCCATC | GTCATATCTCCCCAAACCCCAAT | GENOME_REGION | COSM13170 | hg19 | chr17 | 37880188 | 3,8E+07 | 37880213 | 37880340 | 1 | Pool1 |
| CHP2_ERBB2_1 | GAATGTGAAAATTCCAGTGGCCATC | GTCATATCTCCCCAAACCCCAAT | GENOME_REGION | COSM14060 | hg19 | chr17 | 37880188 | 3,8E+07 | 37880213 | 37880340 | 1 | Pool1 |
| CHP2_ERBB2_1 | GAATGTGAAAATTCCAGTGGCCATC | GTCATATCTCCCCAAACCCCAAT | GENOME_REGION | COSM51317 | hg19 | chr17 | 37880188 | 3,8E+07 | 37880213 | 37880340 | 1 | Pool1 |
| CHP2_ERBB2_1 | GAATGTGAAAATTCCAGTGGCCATC | GTCATATCTCCCCAAACCCCAAT | GENOME_REGION | COSM683 | hg19 | chr17 | 37880188 | 3,8E+07 | 37880213 | 37880340 | 1 | Pool1 |
| CHP2_ERBB2_2 | GGGTGTGTGGTCTCCCATAC | GCCATAGGGCATAAGCTGTGTC | GENOME_REGION | COSM12552 | hg19 | chr17 | 37880934 | 3,8E+07 | 37880954 | 37881061 | 1 | Pool1 |
| CHP2_ERBB2_2 | GGGTGTGTGGTCTCCCATAC | GCCATAGGGCATAAGCTGTGTC | GENOME_REGION | COSM12553 | hg19 | chr17 | 37880934 | 3,8E+07 | 37880954 | 37881061 | 1 | Pool1 |
| CHP2_ERBB2_2 | GGGTGTGTGGTCTCCCATAC | GCCATAGGGCATAAGCTGTGTC | GENOME_REGION | COSM14062 | hg19 | chr17 | 37880934 | 3,8E+07 | 37880954 | 37881061 | 1 | Pool1 |
| CHP2_ERBB2_2 | GGGTGTGTGGTCTCCCATAC | GCCATAGGGCATAAGCTGTGTC | GENOME_REGION | COSM18609 | hg19 | chr17 | 37880934 | 3,8E+07 | 37880954 | 37881061 | 1 | Pool1 |
| CHP2_ERBB2_2 | GGGTGTGTGGTCTCCCATAC | GCCATAGGGCATAAGCTGTGTC | GENOME_REGION | COSM26681 | hg19 | chr17 | 37880934 | 3,8E+07 | 37880954 | 37881061 | 1 | Pool1 |
| CHP2_ERBB2_2 | GGGTGTGTGGTCTCCCATAC | GCCATAGGGCATAAGCTGTGTC | GENOME_REGION | COSM35496 | hg19 | chr17 | 37880934 | 3,8E+07 | 37880954 | 37881061 | 1 | Pool1 |
| CHP2_ERBB2_3 | GGATGAGCTACCTGGAGGATGT | CCTTGGTCCTTCACCTAACCTTG | GENOME_REGION | COSM14065 | hg19 | chr17 | 37881303 | 3,8E+07 | 37881325 | 37881453 | 1 | Pool1 |
| CHP2_ERBB2_3 | GGATGAGCTACCTGGAGGATGT | CCTTGGTCCTTCACCTAACCTTG | GENOME_REGION | COSM21985 | hg19 | chr17 | 37881303 | 3,8E+07 | 37881325 | 37881453 | 1 | Pool1 |
| CHP2_ERBB4_1 | GCCTTAGAGTGTTCCTCAATGTAACAA | GAAACTTTGGACTTCAAGAACTTGGAT | GENOME_REGION | COSM20392 | hg19 | chr2 | 212812049 | 2,1E+08 | 212812076 | 212812169 | 1 | Pool1 |
| CHP2_ERBB4_1 | GCCTTAGAGTGTTCCTCAATGTAACAA | GAAACTTTGGACTTCAAGAACTTGGAT | GENOME_REGION | COSM95705 | hg19 | chr2 | 212812049 | 2,1E+08 | 212812076 | 212812169 | 1 | Pool1 |
| CHP2_ERBB4_2 | CATCGCCACATAGGGTAGAACATTT | CAGACACCATTCATTGGCAAGATATTG | GENOME_REGION | COSM48369 | hg19 | chr2 | 212652695 | 2,1E+08 | 212652720 | 212652806 | 1 | Pool1 |
| CHP2_ERBB4_3 | CCTGAATCAAATAGGGAAGGAAAGGA | GGCAGATGCTACGGACCTTA | GENOME_REGION | COSM48368 | hg19 | chr2 | 212589739 | 2,1E+08 | 212589765 | 212589867 | 1 | Pool1 |
| CHP2_ERBB4_4 | TCTGTTACTTACGTGGACATTTCTTGAC | CAGGCCTGCATGAATTTCAATGA | GENOME_REGION | COSM209862 | hg19 | chr2 | 212587106 | 2,1E+08 | 212587134 | 212587239 | 1 | Pool1 |
| CHP2_ERBB4_4 | TCTGTTACTTACGTGGACATTTCTTGAC | CAGGCCTGCATGAATTTCAATGA | GENOME_REGION | COSM48367 | hg19 | chr2 | 212587106 | 2,1E+08 | 212587134 | 212587239 | 1 | Pool1 |
| CHP2_ERBB4_5 | GGCAAATGTCAGTGCAAGGTTTA | TGTTTTGAGCTTGTTTGCTGAATGT | GENOME_REGION | COSM12833 | hg19 | chr2 | 212578266 | 2,1E+08 | 212578289 | 212578415 | 1 | Pool1 |
| CHP2_ERBB4_5 | GGCAAATGTCAGTGCAAGGTTTA | TGTTTTGAGCTTGTTTGCTGAATGT | GENOME_REGION | COSM131764 | hg19 | chr2 | 212578266 | 2,1E+08 | 212578289 | 212578415 | 1 | Pool1 |
| CHP2_ERBB4_5 | GGCAAATGTCAGTGCAAGGTTTA | TGTTTTGAGCTTGTTTGCTGAATGT | GENOME_REGION | COSM131765 | hg19 | chr2 | 212578266 | 2,1E+08 | 212578289 | 212578415 | 1 | Pool1 |
| CHP2_ERBB4_5 | GGCAAATGTCAGTGCAAGGTTTA | TGTTTTGAGCTTGTTTGCTGAATGT | GENOME_REGION | COSM160825 | hg19 | chr2 | 212578266 | 2,1E+08 | 212578289 | 212578415 | 1 | Pool1 |
| CHP2_ERBB4_5 | GGCAAATGTCAGTGCAAGGTTTA | TGTTTTGAGCTTGTTTGCTGAATGT | GENOME_REGION | COSM48366 | hg19 | chr2 | 212578266 | 2,1E+08 | 212578289 | 212578415 | 1 | Pool1 |
| CHP2_ERBB4_6 | ACCCATGAATACCAGTGACTAGAAAGA | CTCAATCCCCTAACTCTGAGTCTTG | GENOME_REGION | COSM110095 | hg19 | chr2 | 212576773 | 2,1E+08 | 212576800 | 212576910 | 1 | Pool1 |
| CHP2_ERBB4_7 | GCCAGCAAGAATGCTTACCCTT | GGGTCCTGACAACTGTACAAAGT | GENOME_REGION | COSM131772 | hg19 | chr2 | 212530030 | 2,1E+08 | 212530052 | 212530180 | 1 | Pool1 |
| CHP2_ERBB4_7 | GCCAGCAAGAATGCTTACCCTT | GGGTCCTGACAACTGTACAAAGT | GENOME_REGION | COSM138342 | hg19 | chr2 | 212530030 | 2,1E+08 | 212530052 | 212530180 | 1 | Pool1 |
| CHP2_ERBB4_7 | GCCAGCAAGAATGCTTACCCTT | GGGTCCTGACAACTGTACAAAGT | GENOME_REGION | COSM170797 | hg19 | chr2 | 212530030 | 2,1E+08 | 212530052 | 212530180 | 1 | Pool1 |
| CHP2_ERBB4_7 | GCCAGCAAGAATGCTTACCCTT | GGGTCCTGACAACTGTACAAAGT | GENOME_REGION | COSM232263 | hg19 | chr2 | 212530030 | 2,1E+08 | 212530052 | 212530180 | 1 | Pool1 |
| CHP2_ERBB4_7 | GCCAGCAAGAATGCTTACCCTT | GGGTCCTGACAACTGTACAAAGT | GENOME_REGION | COSM48363 | hg19 | chr2 | 212530030 | 2,1E+08 | 212530052 | 212530180 | 1 | Pool1 |
| CHP2_ERBB4_7 | GCCAGCAAGAATGCTTACCCTT | GGGTCCTGACAACTGTACAAAGT | GENOME_REGION | COSM48364 | hg19 | chr2 | 212530030 | 2,1E+08 | 212530052 | 212530180 | 1 | Pool1 |
| CHP2_ERBB4_7 | GCCAGCAAGAATGCTTACCCTT | GGGTCCTGACAACTGTACAAAGT | GENOME_REGION | COSM96313 | hg19 | chr2 | 212530030 | 2,1E+08 | 212530052 | 212530180 | 1 | Pool1 |
| CHP2_ERBB4_8 | CATTTGACCATGACCATGTAAACGTC | GGAACTGATGACCTTTGGAGGAA | GENOME_REGION | COSM108015 | hg19 | chr2 | 212288879 | 2,1E+08 | 212288905 | 212288990 | 1 | Pool1 |
| CHP2_FBXW7_1 | TGACAATGTTTAAAGGTGGTAGCTGTT | ACTCATTGATAGTTGTGAACCAACACA | GENOME_REGION | COSM22971 | hg19 | chr4 | 153258875 | 1,5E+08 | 153258902 | 153259023 | 1 | Pool1 |
| CHP2_FBXW7_2 | CCTGTGACTGCTGACCAAACTTTTA | CACATCTTTCTTATAGGTGCTGAAAGG | GENOME_REGION | COSM22973 | hg19 | chr4 | 153250828 | 1,5E+08 | 153250853 | 153250926 | 1 | Pool1 |
| CHP2_FBXW7_3 | CCCAACCATGACAAGATTTTCCC | GGTCATCACAAATGAGAGACAACATCA | GENOME_REGION | COSM117308 | hg19 | chr4 | 153249333 | 1,5E+08 | 153249356 | 153249477 | 1 | Pool1 |
| CHP2_FBXW7_3 | CCCAACCATGACAAGATTTTCCC | GGTCATCACAAATGAGAGACAACATCA | GENOME_REGION | COSM117309 | hg19 | chr4 | 153249333 | 1,5E+08 | 153249356 | 153249477 | 1 | Pool1 |
| CHP2_FBXW7_3 | CCCAACCATGACAAGATTTTCCC | GGTCATCACAAATGAGAGACAACATCA | GENOME_REGION | COSM117310 | hg19 | chr4 | 153249333 | 1,5E+08 | 153249356 | 153249477 | 1 | Pool1 |
| CHP2_FBXW7_3 | CCCAACCATGACAAGATTTTCCC | GGTCATCACAAATGAGAGACAACATCA | GENOME_REGION | COSM133115 | hg19 | chr4 | 153249333 | 1,5E+08 | 153249356 | 153249477 | 1 | Pool1 |
| CHP2_FBXW7_3 | CCCAACCATGACAAGATTTTCCC | GGTCATCACAAATGAGAGACAACATCA | GENOME_REGION | COSM170725 | hg19 | chr4 | 153249333 | 1,5E+08 | 153249356 | 153249477 | 1 | Pool1 |
| CHP2_FBXW7_3 | CCCAACCATGACAAGATTTTCCC | GGTCATCACAAATGAGAGACAACATCA | GENOME_REGION | COSM170726 | hg19 | chr4 | 153249333 | 1,5E+08 | 153249356 | 153249477 | 1 | Pool1 |
| CHP2_FBXW7_3 | CCCAACCATGACAAGATTTTCCC | GGTCATCACAAATGAGAGACAACATCA | GENOME_REGION | COSM170727 | hg19 | chr4 | 153249333 | 1,5E+08 | 153249356 | 153249477 | 1 | Pool1 |
| CHP2_FBXW7_3 | CCCAACCATGACAAGATTTTCCC | GGTCATCACAAATGAGAGACAACATCA | GENOME_REGION | COSM22932 | hg19 | chr4 | 153249333 | 1,5E+08 | 153249356 | 153249477 | 1 | Pool1 |
| CHP2_FBXW7_3 | CCCAACCATGACAAGATTTTCCC | GGTCATCACAAATGAGAGACAACATCA | GENOME_REGION | COSM22965 | hg19 | chr4 | 153249333 | 1,5E+08 | 153249356 | 153249477 | 1 | Pool1 |
| CHP2_FBXW7_3 | CCCAACCATGACAAGATTTTCCC | GGTCATCACAAATGAGAGACAACATCA | GENOME_REGION | COSM33762 | hg19 | chr4 | 153249333 | 1,5E+08 | 153249356 | 153249477 | 1 | Pool1 |
| CHP2_FBXW7_4 | ACTAACAACCCTCCTGCCATCATA | TCTGCAGAGTTGTTAGCGGTT | GENOME_REGION | COSM108571 | hg19 | chr4 | 153247254 | 1,5E+08 | 153247278 | 153247369 | 1 | Pool1 |
| CHP2_FBXW7_4 | ACTAACAACCCTCCTGCCATCATA | TCTGCAGAGTTGTTAGCGGTT | GENOME_REGION | COSM108572 | hg19 | chr4 | 153247254 | 1,5E+08 | 153247278 | 153247369 | 1 | Pool1 |
| CHP2_FBXW7_4 | ACTAACAACCCTCCTGCCATCATA | TCTGCAGAGTTGTTAGCGGTT | GENOME_REGION | COSM22975 | hg19 | chr4 | 153247254 | 1,5E+08 | 153247278 | 153247369 | 1 | Pool1 |
| CHP2_FBXW7_4 | ACTAACAACCCTCCTGCCATCATA | TCTGCAGAGTTGTTAGCGGTT | GENOME_REGION | COSM23000 | hg19 | chr4 | 153247254 | 1,5E+08 | 153247278 | 153247369 | 1 | Pool1 |
| CHP2_FBXW7_4 | ACTAACAACCCTCCTGCCATCATA | TCTGCAGAGTTGTTAGCGGTT | GENOME_REGION | COSM25812 | hg19 | chr4 | 153247254 | 1,5E+08 | 153247278 | 153247369 | 1 | Pool1 |
| CHP2_FBXW7_4 | ACTAACAACCCTCCTGCCATCATA | TCTGCAGAGTTGTTAGCGGTT | GENOME_REGION | COSM27055 | hg19 | chr4 | 153247254 | 1,5E+08 | 153247278 | 153247369 | 1 | Pool1 |
| CHP2_FBXW7_4 | ACTAACAACCCTCCTGCCATCATA | TCTGCAGAGTTGTTAGCGGTT | GENOME_REGION | COSM74637 | hg19 | chr4 | 153247254 | 1,5E+08 | 153247278 | 153247369 | 1 | Pool1 |
| CHP2_FBXW7_4 | ACTAACAACCCTCCTGCCATCATA | TCTGCAGAGTTGTTAGCGGTT | GENOME_REGION | COSM99603 | hg19 | chr4 | 153247254 | 1,5E+08 | 153247278 | 153247369 | 1 | Pool1 |
| CHP2_FBXW7_4 | ACTAACAACCCTCCTGCCATCATA | TCTGCAGAGTTGTTAGCGGTT | GENOME_REGION | COSM99604 | hg19 | chr4 | 153247254 | 1,5E+08 | 153247278 | 153247369 | 1 | Pool1 |
| CHP2_FBXW7_4 | ACTAACAACCCTCCTGCCATCATA | TCTGCAGAGTTGTTAGCGGTT | GENOME_REGION | COSM99605 | hg19 | chr4 | 153247254 | 1,5E+08 | 153247278 | 153247369 | 1 | Pool1 |
| CHP2_FBXW7_4 | ACTAACAACCCTCCTGCCATCATA | TCTGCAGAGTTGTTAGCGGTT | GENOME_REGION | COSM99606 | hg19 | chr4 | 153247254 | 1,5E+08 | 153247278 | 153247369 | 1 | Pool1 |
| CHP2_FBXW7_5 | GTAGAATCTGCATTCCCAGAGACAA | TCTCTTGATACATCAATCCGTGTTTGG | GENOME_REGION | COSM22979 | hg19 | chr4 | 153245386 | 1,5E+08 | 153245411 | 153245492 | 1 | Pool1 |
| CHP2_FGFR1_1 | ACCCAAAGGGCAGTAAGATAGGAA | GGTCCCTAGGAGGAACCTCA | GENOME_REGION | COSM187237 | hg19 | chr8 | 38285828 | 3,8E+07 | 38285852 | 38285975 | 1 | Pool1 |
| CHP2_FGFR1_1 | ACCCAAAGGGCAGTAAGATAGGAA | GGTCCCTAGGAGGAACCTCA | GENOME_REGION | COSM187238 | hg19 | chr8 | 38285828 | 3,8E+07 | 38285852 | 38285975 | 1 | Pool1 |
| CHP2_FGFR1_1 | ACCCAAAGGGCAGTAAGATAGGAA | GGTCCCTAGGAGGAACCTCA | GENOME_REGION | COSM187239 | hg19 | chr8 | 38285828 | 3,8E+07 | 38285852 | 38285975 | 1 | Pool1 |
| CHP2_FGFR1_1 | ACCCAAAGGGCAGTAAGATAGGAA | GGTCCCTAGGAGGAACCTCA | GENOME_REGION | COSM48380 | hg19 | chr8 | 38285828 | 3,8E+07 | 38285852 | 38285975 | 1 | Pool1 |
| CHP2_FGFR1_1 | ACCCAAAGGGCAGTAAGATAGGAA | GGTCCCTAGGAGGAACCTCA | GENOME_REGION | COSM601 | hg19 | chr8 | 38285828 | 3,8E+07 | 38285852 | 38285975 | 1 | Pool1 |
| CHP2_FGFR1_1 | ACCCAAAGGGCAGTAAGATAGGAA | GGTCCCTAGGAGGAACCTCA | GENOME_REGION | COSM98900 | hg19 | chr8 | 38285828 | 3,8E+07 | 38285852 | 38285975 | 1 | Pool1 |
| CHP2_FGFR1_1 | ACCCAAAGGGCAGTAAGATAGGAA | GGTCCCTAGGAGGAACCTCA | GENOME_REGION | COSM98901 | hg19 | chr8 | 38285828 | 3,8E+07 | 38285852 | 38285975 | 1 | Pool1 |
| CHP2_FGFR1_1 | ACCCAAAGGGCAGTAAGATAGGAA | GGTCCCTAGGAGGAACCTCA | GENOME_REGION | COSM98902 | hg19 | chr8 | 38285828 | 3,8E+07 | 38285852 | 38285975 | 1 | Pool1 |
| CHP2_FGFR1_1 | ACCCAAAGGGCAGTAAGATAGGAA | GGTCCCTAGGAGGAACCTCA | GENOME_REGION | COSM98903 | hg19 | chr8 | 38285828 | 3,8E+07 | 38285852 | 38285975 | 1 | Pool1 |
| CHP2_FGFR1_2 | GGTCACTGTACACCTTACACATGAA | CCCTCTTTAGCCATGGCAAGG | GENOME_REGION | COSM12834 | hg19 | chr8 | 38282116 | 3,8E+07 | 38282141 | 38282254 | 1 | Pool1 |
| CHP2_FGFR2_1 | CATCACTGTAAACCTTGCAGACAAAC | TGGTCTCTCATTCTCCCATCCC | GENOME_REGION | COSM36903 | hg19 | chr10 | 123279582 | 1,2E+08 | 123279608 | 123279713 | 1 | Pool1 |
| CHP2_FGFR2_1 | CATCACTGTAAACCTTGCAGACAAAC | TGGTCTCTCATTCTCCCATCCC | GENOME_REGION | COSM49170 | hg19 | chr10 | 123279582 | 1,2E+08 | 123279608 | 123279713 | 1 | Pool1 |
| CHP2_FGFR2_2 | CATCCTCTCTCAACTCCAACAGG | AGTGGATCAAGCACGTGGAAAA | GENOME_REGION | COSM36901 | hg19 | chr10 | 123279395 | 1,2E+08 | 123279418 | 123279544 | 1 | Pool1 |
| CHP2_FGFR2_3 | GCTTCTTGGTCGTGTTCTTCATT | CTCCTCCTGTGATCTGCAATCT | GENOME_REGION | COSM36904 | hg19 | chr10 | 123274699 | 1,2E+08 | 123274722 | 123274835 | 1 | Pool1 |
| CHP2_FGFR2_3 | GCTTCTTGGTCGTGTTCTTCATT | CTCCTCCTGTGATCTGCAATCT | GENOME_REGION | COSM36906 | hg19 | chr10 | 123274699 | 1,2E+08 | 123274722 | 123274835 | 1 | Pool1 |
| CHP2_FGFR2_4 | TGGAAGCCCAGCCATTTCTAAA | GATGATGAAGATGATTGGGAAACACAAG | GENOME_REGION | COSM36902 | hg19 | chr10 | 123257931 | 1,2E+08 | 123257953 | 123258045 | 1 | Pool1 |
| CHP2_FGFR2_4 | TGGAAGCCCAGCCATTTCTAAA | GATGATGAAGATGATTGGGAAACACAAG | GENOME_REGION | COSM36912 | hg19 | chr10 | 123257931 | 1,2E+08 | 123257953 | 123258045 | 1 | Pool1 |
| CHP2_FGFR3_1 | gCCCCTGAGCGTCATCT | GGGCTGTGCGTCACTGTA | GENOME_REGION | COSM29446 | hg19 | chr4 | 1803535 | 1803671 | 1803552 | 1803653 | 1 | Pool1 |
| CHP2_FGFR3_1 | gCCCCTGAGCGTCATCT | GGGCTGTGCGTCACTGTA | GENOME_REGION | COSM714 | hg19 | chr4 | 1803535 | 1803671 | 1803552 | 1803653 | 1 | Pool1 |
| CHP2_FGFR3_1 | gCCCCTGAGCGTCATCT | GGGCTGTGCGTCACTGTA | GENOME_REGION | COSM715 | hg19 | chr4 | 1803535 | 1803671 | 1803552 | 1803653 | 1 | Pool1 |
| CHP2_FGFR3_2 | GAGCTGGTGGAGGCTGA | GGAGCCCAGGCCTTTCTT | GENOME_REGION | COSM17461 | hg19 | chr4 | 1806065 | 1806205 | 1806082 | 1806187 | 1 | Pool1 |
| CHP2_FGFR3_2 | GAGCTGGTGGAGGCTGA | GGAGCCCAGGCCTTTCTT | GENOME_REGION | COSM24842 | hg19 | chr4 | 1806065 | 1806205 | 1806082 | 1806187 | 1 | Pool1 |
| CHP2_FGFR3_2 | GAGCTGGTGGAGGCTGA | GGAGCCCAGGCCTTTCTT | GENOME_REGION | COSM716 | hg19 | chr4 | 1806065 | 1806205 | 1806082 | 1806187 | 1 | Pool1 |
| CHP2_FGFR3_2 | GAGCTGGTGGAGGCTGA | GGAGCCCAGGCCTTTCTT | GENOME_REGION | COSM718 | hg19 | chr4 | 1806065 | 1806205 | 1806082 | 1806187 | 1 | Pool1 |
| CHP2_FGFR3_2 | GAGCTGGTGGAGGCTGA | GGAGCCCAGGCCTTTCTT | GENOME_REGION | COSM721 | hg19 | chr4 | 1806065 | 1806205 | 1806082 | 1806187 | 1 | Pool1 |
| CHP2_FGFR3_2 | GAGCTGGTGGAGGCTGA | GGAGCCCAGGCCTTTCTT | GENOME_REGION | COSM722 | hg19 | chr4 | 1806065 | 1806205 | 1806082 | 1806187 | 1 | Pool1 |
| CHP2_FGFR3_2 | GAGCTGGTGGAGGCTGA | GGAGCCCAGGCCTTTCTT | GENOME_REGION | COSM724 | hg19 | chr4 | 1806065 | 1806205 | 1806082 | 1806187 | 1 | Pool1 |
| CHP2_FGFR3_3 | GTGACCGAGGACAACGTGAT | GCGTCCTACTGGCATGACC | GENOME_REGION | COSM29438 | hg19 | chr4 | 1807814 | 1807949 | 1807834 | 1807930 | 1 | Pool1 |
| CHP2_FGFR3_3 | GTGACCGAGGACAACGTGAT | GCGTCCTACTGGCATGACC | GENOME_REGION | COSM719 | hg19 | chr4 | 1807814 | 1807949 | 1807834 | 1807930 | 1 | Pool1 |
| CHP2_FGFR3_3 | GTGACCGAGGACAACGTGAT | GCGTCCTACTGGCATGACC | GENOME_REGION | COSM720 | hg19 | chr4 | 1807814 | 1807949 | 1807834 | 1807930 | 1 | Pool1 |
| CHP2_FGFR3_3 | GTGACCGAGGACAACGTGAT | GCGTCCTACTGGCATGACC | GENOME_REGION | COSM726 | hg19 | chr4 | 1807814 | 1807949 | 1807834 | 1807930 | 1 | Pool1 |
| CHP2_FGFR3_3 | GTGACCGAGGACAACGTGAT | GCGTCCTACTGGCATGACC | GENOME_REGION | COSM731 | hg19 | chr4 | 1807814 | 1807949 | 1807834 | 1807930 | 1 | Pool1 |
| CHP2_FGFR3_4 | CTCTGGGAGATCTTCACGCT | CCACTCACAGGTCGTGTGT | GENOME_REGION | COSM24802 | hg19 | chr4 | 1808292 | 1808418 | 1808312 | 1808399 | 1 | Pool1 |
| CHP2_FGFR3_5 | CGCCTTTCGAGCAGTACTCC | GCTAGGGACCCCTCACATTGT | GENOME_REGION | COSM27139 | hg19 | chr4 | 1808862 | 1809027 | 1808882 | 1809006 | 1 | Pool1 |
| CHP2_FGFR3_5 | CGCCTTTCGAGCAGTACTCC | GCTAGGGACCCCTCACATTGT | GENOME_REGION | COSM725 | hg19 | chr4 | 1808862 | 1809027 | 1808882 | 1809006 | 1 | Pool1 |
| CHP2_FGFR3_5 | CGCCTTTCGAGCAGTACTCC | GCTAGGGACCCCTCACATTGT | GENOME_REGION | COSM729 | hg19 | chr4 | 1808862 | 1809027 | 1808882 | 1809006 | 1 | Pool1 |
| CHP2_KRAS_1 | CAAAGAATGGTCCTGCACCAGTAATAT | AGGCCTGCTGAAAATGACTGAATATAA | GENOME_REGION | COSM12654 | hg19 | chr12 | 25398160 | 2,5E+07 | 25398187 | 25398304 | 1 | Pool1 |
| CHP2_KRAS_1 | CAAAGAATGGTCCTGCACCAGTAATAT | AGGCCTGCTGAAAATGACTGAATATAA | GENOME_REGION | COSM12655 | hg19 | chr12 | 25398160 | 2,5E+07 | 25398187 | 25398304 | 1 | Pool1 |
| CHP2_KRAS_1 | CAAAGAATGGTCCTGCACCAGTAATAT | AGGCCTGCTGAAAATGACTGAATATAA | GENOME_REGION | COSM12703 | hg19 | chr12 | 25398160 | 2,5E+07 | 25398187 | 25398304 | 1 | Pool1 |
| CHP2_KRAS_1 | CAAAGAATGGTCCTGCACCAGTAATAT | AGGCCTGCTGAAAATGACTGAATATAA | GENOME_REGION | COSM12721 | hg19 | chr12 | 25398160 | 2,5E+07 | 25398187 | 25398304 | 1 | Pool1 |
| CHP2_KRAS_1 | CAAAGAATGGTCCTGCACCAGTAATAT | AGGCCTGCTGAAAATGACTGAATATAA | GENOME_REGION | COSM12722 | hg19 | chr12 | 25398160 | 2,5E+07 | 25398187 | 25398304 | 1 | Pool1 |
| CHP2_KRAS_1 | CAAAGAATGGTCCTGCACCAGTAATAT | AGGCCTGCTGAAAATGACTGAATATAA | GENOME_REGION | COSM14209 | hg19 | chr12 | 25398160 | 2,5E+07 | 25398187 | 25398304 | 1 | Pool1 |
| CHP2_KRAS_1 | CAAAGAATGGTCCTGCACCAGTAATAT | AGGCCTGCTGAAAATGACTGAATATAA | GENOME_REGION | COSM20818 | hg19 | chr12 | 25398160 | 2,5E+07 | 25398187 | 25398304 | 1 | Pool1 |
| CHP2_KRAS_1 | CAAAGAATGGTCCTGCACCAGTAATAT | AGGCCTGCTGAAAATGACTGAATATAA | GENOME_REGION | COSM219781 | hg19 | chr12 | 25398160 | 2,5E+07 | 25398187 | 25398304 | 1 | Pool1 |
| CHP2_KRAS_1 | CAAAGAATGGTCCTGCACCAGTAATAT | AGGCCTGCTGAAAATGACTGAATATAA | GENOME_REGION | COSM25081 | hg19 | chr12 | 25398160 | 2,5E+07 | 25398187 | 25398304 | 1 | Pool1 |
| CHP2_KRAS_1 | CAAAGAATGGTCCTGCACCAGTAATAT | AGGCCTGCTGAAAATGACTGAATATAA | GENOME_REGION | COSM34144 | hg19 | chr12 | 25398160 | 2,5E+07 | 25398187 | 25398304 | 1 | Pool1 |
| CHP2_KRAS_1 | CAAAGAATGGTCCTGCACCAGTAATAT | AGGCCTGCTGAAAATGACTGAATATAA | GENOME_REGION | COSM507 | hg19 | chr12 | 25398160 | 2,5E+07 | 25398187 | 25398304 | 1 | Pool1 |
| CHP2_KRAS_1 | CAAAGAATGGTCCTGCACCAGTAATAT | AGGCCTGCTGAAAATGACTGAATATAA | GENOME_REGION | COSM510 | hg19 | chr12 | 25398160 | 2,5E+07 | 25398187 | 25398304 | 1 | Pool1 |
| CHP2_KRAS_1 | CAAAGAATGGTCCTGCACCAGTAATAT | AGGCCTGCTGAAAATGACTGAATATAA | GENOME_REGION | COSM511 | hg19 | chr12 | 25398160 | 2,5E+07 | 25398187 | 25398304 | 1 | Pool1 |
| CHP2_KRAS_1 | CAAAGAATGGTCCTGCACCAGTAATAT | AGGCCTGCTGAAAATGACTGAATATAA | GENOME_REGION | COSM512 | hg19 | chr12 | 25398160 | 2,5E+07 | 25398187 | 25398304 | 1 | Pool1 |
| CHP2_KRAS_1 | CAAAGAATGGTCCTGCACCAGTAATAT | AGGCCTGCTGAAAATGACTGAATATAA | GENOME_REGION | COSM514 | hg19 | chr12 | 25398160 | 2,5E+07 | 25398187 | 25398304 | 1 | Pool1 |
| CHP2_KRAS_1 | CAAAGAATGGTCCTGCACCAGTAATAT | AGGCCTGCTGAAAATGACTGAATATAA | GENOME_REGION | COSM515 | hg19 | chr12 | 25398160 | 2,5E+07 | 25398187 | 25398304 | 1 | Pool1 |
| CHP2_KRAS_1 | CAAAGAATGGTCCTGCACCAGTAATAT | AGGCCTGCTGAAAATGACTGAATATAA | GENOME_REGION | COSM516 | hg19 | chr12 | 25398160 | 2,5E+07 | 25398187 | 25398304 | 1 | Pool1 |
| CHP2_KRAS_1 | CAAAGAATGGTCCTGCACCAGTAATAT | AGGCCTGCTGAAAATGACTGAATATAA | GENOME_REGION | COSM517 | hg19 | chr12 | 25398160 | 2,5E+07 | 25398187 | 25398304 | 1 | Pool1 |
| CHP2_KRAS_1 | CAAAGAATGGTCCTGCACCAGTAATAT | AGGCCTGCTGAAAATGACTGAATATAA | GENOME_REGION | COSM518 | hg19 | chr12 | 25398160 | 2,5E+07 | 25398187 | 25398304 | 1 | Pool1 |
| CHP2_KRAS_1 | CAAAGAATGGTCCTGCACCAGTAATAT | AGGCCTGCTGAAAATGACTGAATATAA | GENOME_REGION | COSM519 | hg19 | chr12 | 25398160 | 2,5E+07 | 25398187 | 25398304 | 1 | Pool1 |
| CHP2_KRAS_1 | CAAAGAATGGTCCTGCACCAGTAATAT | AGGCCTGCTGAAAATGACTGAATATAA | GENOME_REGION | COSM520 | hg19 | chr12 | 25398160 | 2,5E+07 | 25398187 | 25398304 | 1 | Pool1 |
| CHP2_KRAS_1 | CAAAGAATGGTCCTGCACCAGTAATAT | AGGCCTGCTGAAAATGACTGAATATAA | GENOME_REGION | COSM521 | hg19 | chr12 | 25398160 | 2,5E+07 | 25398187 | 25398304 | 1 | Pool1 |
| CHP2_KRAS_1 | CAAAGAATGGTCCTGCACCAGTAATAT | AGGCCTGCTGAAAATGACTGAATATAA | GENOME_REGION | COSM522 | hg19 | chr12 | 25398160 | 2,5E+07 | 25398187 | 25398304 | 1 | Pool1 |
| CHP2_KRAS_1 | CAAAGAATGGTCCTGCACCAGTAATAT | AGGCCTGCTGAAAATGACTGAATATAA | GENOME_REGION | COSM523 | hg19 | chr12 | 25398160 | 2,5E+07 | 25398187 | 25398304 | 1 | Pool1 |
| CHP2_KRAS_1 | CAAAGAATGGTCCTGCACCAGTAATAT | AGGCCTGCTGAAAATGACTGAATATAA | GENOME_REGION | COSM524 | hg19 | chr12 | 25398160 | 2,5E+07 | 25398187 | 25398304 | 1 | Pool1 |
| CHP2_KRAS_1 | CAAAGAATGGTCCTGCACCAGTAATAT | AGGCCTGCTGAAAATGACTGAATATAA | GENOME_REGION | COSM527 | hg19 | chr12 | 25398160 | 2,5E+07 | 25398187 | 25398304 | 1 | Pool1 |
| CHP2_KRAS_1 | CAAAGAATGGTCCTGCACCAGTAATAT | AGGCCTGCTGAAAATGACTGAATATAA | GENOME_REGION | COSM528 | hg19 | chr12 | 25398160 | 2,5E+07 | 25398187 | 25398304 | 1 | Pool1 |
| CHP2_KRAS_1 | CAAAGAATGGTCCTGCACCAGTAATAT | AGGCCTGCTGAAAATGACTGAATATAA | GENOME_REGION | COSM529 | hg19 | chr12 | 25398160 | 2,5E+07 | 25398187 | 25398304 | 1 | Pool1 |
| CHP2_KRAS_1 | CAAAGAATGGTCCTGCACCAGTAATAT | AGGCCTGCTGAAAATGACTGAATATAA | GENOME_REGION | COSM531 | hg19 | chr12 | 25398160 | 2,5E+07 | 25398187 | 25398304 | 1 | Pool1 |
| CHP2_KRAS_1 | CAAAGAATGGTCCTGCACCAGTAATAT | AGGCCTGCTGAAAATGACTGAATATAA | GENOME_REGION | COSM532 | hg19 | chr12 | 25398160 | 2,5E+07 | 25398187 | 25398304 | 1 | Pool1 |
| CHP2_KRAS_1 | CAAAGAATGGTCCTGCACCAGTAATAT | AGGCCTGCTGAAAATGACTGAATATAA | GENOME_REGION | COSM533 | hg19 | chr12 | 25398160 | 2,5E+07 | 25398187 | 25398304 | 1 | Pool1 |
| CHP2_KRAS_1 | CAAAGAATGGTCCTGCACCAGTAATAT | AGGCCTGCTGAAAATGACTGAATATAA | GENOME_REGION | COSM534 | hg19 | chr12 | 25398160 | 2,5E+07 | 25398187 | 25398304 | 1 | Pool1 |
| CHP2_KRAS_1 | CAAAGAATGGTCCTGCACCAGTAATAT | AGGCCTGCTGAAAATGACTGAATATAA | GENOME_REGION | COSM535 | hg19 | chr12 | 25398160 | 2,5E+07 | 25398187 | 25398304 | 1 | Pool1 |
| CHP2_KRAS_1 | CAAAGAATGGTCCTGCACCAGTAATAT | AGGCCTGCTGAAAATGACTGAATATAA | GENOME_REGION | COSM536 | hg19 | chr12 | 25398160 | 2,5E+07 | 25398187 | 25398304 | 1 | Pool1 |
| CHP2_KRAS_1 | CAAAGAATGGTCCTGCACCAGTAATAT | AGGCCTGCTGAAAATGACTGAATATAA | GENOME_REGION | COSM537 | hg19 | chr12 | 25398160 | 2,5E+07 | 25398187 | 25398304 | 1 | Pool1 |
| CHP2_KRAS_1 | CAAAGAATGGTCCTGCACCAGTAATAT | AGGCCTGCTGAAAATGACTGAATATAA | GENOME_REGION | COSM538 | hg19 | chr12 | 25398160 | 2,5E+07 | 25398187 | 25398304 | 1 | Pool1 |
| CHP2_KRAS_1 | CAAAGAATGGTCCTGCACCAGTAATAT | AGGCCTGCTGAAAATGACTGAATATAA | GENOME_REGION | COSM542 | hg19 | chr12 | 25398160 | 2,5E+07 | 25398187 | 25398304 | 1 | Pool1 |
| CHP2_KRAS_1 | CAAAGAATGGTCCTGCACCAGTAATAT | AGGCCTGCTGAAAATGACTGAATATAA | GENOME_REGION | COSM543 | hg19 | chr12 | 25398160 | 2,5E+07 | 25398187 | 25398304 | 1 | Pool1 |
| CHP2_KRAS_1 | CAAAGAATGGTCCTGCACCAGTAATAT | AGGCCTGCTGAAAATGACTGAATATAA | GENOME_REGION | COSM87280 | hg19 | chr12 | 25398160 | 2,5E+07 | 25398187 | 25398304 | 1 | Pool1 |
| CHP2_KRAS_1 | CAAAGAATGGTCCTGCACCAGTAATAT | AGGCCTGCTGAAAATGACTGAATATAA | GENOME_REGION | COSM87301 | hg19 | chr12 | 25398160 | 2,5E+07 | 25398187 | 25398304 | 1 | Pool1 |
| CHP2_KRAS_2 | TCCTCATGTACTGGTCCCTCATT | GTAAAAGGTGCACTGTAATAATCCAGACT | GENOME_REGION | COSM28518 | hg19 | chr12 | 25380238 | 2,5E+07 | 25380261 | 25380364 | 1 | Pool1 |
| CHP2_KRAS_2 | TCCTCATGTACTGGTCCCTCATT | GTAAAAGGTGCACTGTAATAATCCAGACT | GENOME_REGION | COSM546 | hg19 | chr12 | 25380238 | 2,5E+07 | 25380261 | 25380364 | 1 | Pool1 |
| CHP2_KRAS_2 | TCCTCATGTACTGGTCCCTCATT | GTAAAAGGTGCACTGTAATAATCCAGACT | GENOME_REGION | COSM547 | hg19 | chr12 | 25380238 | 2,5E+07 | 25380261 | 25380364 | 1 | Pool1 |
| CHP2_KRAS_2 | TCCTCATGTACTGGTCCCTCATT | GTAAAAGGTGCACTGTAATAATCCAGACT | GENOME_REGION | COSM549 | hg19 | chr12 | 25380238 | 2,5E+07 | 25380261 | 25380364 | 1 | Pool1 |
| CHP2_KRAS_2 | TCCTCATGTACTGGTCCCTCATT | GTAAAAGGTGCACTGTAATAATCCAGACT | GENOME_REGION | COSM550 | hg19 | chr12 | 25380238 | 2,5E+07 | 25380261 | 25380364 | 1 | Pool1 |
| CHP2_KRAS_2 | TCCTCATGTACTGGTCCCTCATT | GTAAAAGGTGCACTGTAATAATCCAGACT | GENOME_REGION | COSM551 | hg19 | chr12 | 25380238 | 2,5E+07 | 25380261 | 25380364 | 1 | Pool1 |
| CHP2_KRAS_2 | TCCTCATGTACTGGTCCCTCATT | GTAAAAGGTGCACTGTAATAATCCAGACT | GENOME_REGION | COSM552 | hg19 | chr12 | 25380238 | 2,5E+07 | 25380261 | 25380364 | 1 | Pool1 |
| CHP2_KRAS_2 | TCCTCATGTACTGGTCCCTCATT | GTAAAAGGTGCACTGTAATAATCCAGACT | GENOME_REGION | COSM553 | hg19 | chr12 | 25380238 | 2,5E+07 | 25380261 | 25380364 | 1 | Pool1 |
| CHP2_KRAS_2 | TCCTCATGTACTGGTCCCTCATT | GTAAAAGGTGCACTGTAATAATCCAGACT | GENOME_REGION | COSM554 | hg19 | chr12 | 25380238 | 2,5E+07 | 25380261 | 25380364 | 1 | Pool1 |
| CHP2_KRAS_2 | TCCTCATGTACTGGTCCCTCATT | GTAAAAGGTGCACTGTAATAATCCAGACT | GENOME_REGION | COSM555 | hg19 | chr12 | 25380238 | 2,5E+07 | 25380261 | 25380364 | 1 | Pool1 |
| CHP2_KRAS_2 | TCCTCATGTACTGGTCCCTCATT | GTAAAAGGTGCACTGTAATAATCCAGACT | GENOME_REGION | COSM87288 | hg19 | chr12 | 25380238 | 2,5E+07 | 25380261 | 25380364 | 1 | Pool1 |
| CHP2_KRAS_2 | TCCTCATGTACTGGTCCCTCATT | GTAAAAGGTGCACTGTAATAATCCAGACT | GENOME_REGION | COSM87298 | hg19 | chr12 | 25380238 | 2,5E+07 | 25380261 | 25380364 | 1 | Pool1 |
| CHP2_KRAS_3 | CAGATCTGTATTTATTTCAGTGTTACTTACCT | GACTCTGAAGATGTACCTATGGTCCTA | GENOME_REGION | COSM19404 | hg19 | chr12 | 25378518 | 2,5E+07 | 25378550 | 25378658 | 1 | Pool1 |
| CHP2_KRAS_3 | CAGATCTGTATTTATTTCAGTGTTACTTACCT | GACTCTGAAGATGTACCTATGGTCCTA | GENOME_REGION | COSM19900 | hg19 | chr12 | 25378518 | 2,5E+07 | 25378550 | 25378658 | 1 | Pool1 |
| CHP2_KRAS_3 | CAGATCTGTATTTATTTCAGTGTTACTTACCT | GACTCTGAAGATGTACCTATGGTCCTA | GENOME_REGION | COSM19905 | hg19 | chr12 | 25378518 | 2,5E+07 | 25378550 | 25378658 | 1 | Pool1 |
| CHP2_KRAS_3 | CAGATCTGTATTTATTTCAGTGTTACTTACCT | GACTCTGAAGATGTACCTATGGTCCTA | GENOME_REGION | COSM19940 | hg19 | chr12 | 25378518 | 2,5E+07 | 25378550 | 25378658 | 1 | Pool1 |
| CHP2_KRAS_3 | CAGATCTGTATTTATTTCAGTGTTACTTACCT | GACTCTGAAGATGTACCTATGGTCCTA | GENOME_REGION | COSM28519 | hg19 | chr12 | 25378518 | 2,5E+07 | 25378550 | 25378658 | 1 | Pool1 |
| CHP2_MET_1 | CTGACATACAGTCGGAGGTTCAC | AGAAGTTGATGAACCGGTCCTTT | GENOME_REGION | COSM706 | hg19 | chr7 | 116339593 | 1,2E+08 | 116339616 | 116339701 | 1 | Pool1 |
| CHP2_MET_2 | CAAATAGGAGCCAGCCTGAATGAT | GGAGACATCTCACATTGTTTTTGTTGA | GENOME_REGION | COSM710 | hg19 | chr7 | 116340132 | 1,2E+08 | 116340156 | 116340270 | 1 | Pool1 |
| CHP2_MET_4 | CCCATGATAGCCGTCTTTAACAAG | CGGTAGTCTACAGATTCATTTGAAACCAT | GENOME_REGION | COSM707 | hg19 | chr7 | 116411855 | 1,2E+08 | 116411879 | 116411997 | 1 | Pool1 |
| CHP2_MET_5 | TGTTACGCAGTGCTAACCAAGTT | GCAAACCACAAAAGTATACTCCATGGT | GENOME_REGION | COSM201908 | hg19 | chr7 | 116417405 | 1,2E+08 | 116417428 | 116417542 | 1 | Pool1 |
| CHP2_MET_5 | TGTTACGCAGTGCTAACCAAGTT | GCAAACCACAAAAGTATACTCCATGGT | GENOME_REGION | COSM696 | hg19 | chr7 | 116417405 | 1,2E+08 | 116417428 | 116417542 | 1 | Pool1 |
| CHP2_MET_5 | TGTTACGCAGTGCTAACCAAGTT | GCAAACCACAAAAGTATACTCCATGGT | GENOME_REGION | COSM697 | hg19 | chr7 | 116417405 | 1,2E+08 | 116417428 | 116417542 | 1 | Pool1 |
| CHP2_MET_5 | TGTTACGCAGTGCTAACCAAGTT | GCAAACCACAAAAGTATACTCCATGGT | GENOME_REGION | COSM698 | hg19 | chr7 | 116417405 | 1,2E+08 | 116417428 | 116417542 | 1 | Pool1 |
| CHP2_MET_5 | TGTTACGCAGTGCTAACCAAGTT | GCAAACCACAAAAGTATACTCCATGGT | GENOME_REGION | COSM701 | hg19 | chr7 | 116417405 | 1,2E+08 | 116417428 | 116417542 | 1 | Pool1 |
| CHP2_MET_5 | TGTTACGCAGTGCTAACCAAGTT | GCAAACCACAAAAGTATACTCCATGGT | GENOME_REGION | COSM702 | hg19 | chr7 | 116417405 | 1,2E+08 | 116417428 | 116417542 | 1 | Pool1 |
| CHP2_MET_5 | TGTTACGCAGTGCTAACCAAGTT | GCAAACCACAAAAGTATACTCCATGGT | GENOME_REGION | COSM703 | hg19 | chr7 | 116417405 | 1,2E+08 | 116417428 | 116417542 | 1 | Pool1 |
| CHP2_MET_6 | GCTGATTTTGGTCTTGCCAGAG | TCTGACTTGGTGGTAAACTTTTGAGTT | GENOME_REGION | COSM691 | hg19 | chr7 | 116423386 | 1,2E+08 | 116423408 | 116423492 | 1 | Pool1 |
| CHP2_MET_6 | GCTGATTTTGGTCTTGCCAGAG | TCTGACTTGGTGGTAAACTTTTGAGTT | GENOME_REGION | COSM699 | hg19 | chr7 | 116423386 | 1,2E+08 | 116423408 | 116423492 | 1 | Pool1 |
| CHP2_MET_6 | GCTGATTTTGGTCTTGCCAGAG | TCTGACTTGGTGGTAAACTTTTGAGTT | GENOME_REGION | COSM700 | hg19 | chr7 | 116423386 | 1,2E+08 | 116423408 | 116423492 | 1 | Pool1 |
| CHP2_NOTCH1_1 | CACGCTTGAAGACCACGTTG | GGACTGTGCGGAGCATGTA | GENOME_REGION | COSM12771 | hg19 | chr9 | 139399318 | 1,4E+08 | 139399338 | 139399447 | 1 | Pool1 |
| CHP2_NOTCH1_1 | CACGCTTGAAGACCACGTTG | GGACTGTGCGGAGCATGTA | GENOME_REGION | COSM12772 | hg19 | chr9 | 139399318 | 1,4E+08 | 139399338 | 139399447 | 1 | Pool1 |
| CHP2_NOTCH1_1 | CACGCTTGAAGACCACGTTG | GGACTGTGCGGAGCATGTA | GENOME_REGION | COSM13042 | hg19 | chr9 | 139399318 | 1,4E+08 | 139399338 | 139399447 | 1 | Pool1 |
| CHP2_NOTCH1_1 | CACGCTTGAAGACCACGTTG | GGACTGTGCGGAGCATGTA | GENOME_REGION | COSM13046 | hg19 | chr9 | 139399318 | 1,4E+08 | 139399338 | 139399447 | 1 | Pool1 |
| CHP2_NOTCH1_1 | CACGCTTGAAGACCACGTTG | GGACTGTGCGGAGCATGTA | GENOME_REGION | COSM13047 | hg19 | chr9 | 139399318 | 1,4E+08 | 139399338 | 139399447 | 1 | Pool1 |
| CHP2_NOTCH1_1 | CACGCTTGAAGACCACGTTG | GGACTGTGCGGAGCATGTA | GENOME_REGION | COSM13050 | hg19 | chr9 | 139399318 | 1,4E+08 | 139399338 | 139399447 | 1 | Pool1 |
| CHP2_NOTCH1_1 | CACGCTTGAAGACCACGTTG | GGACTGTGCGGAGCATGTA | GENOME_REGION | COSM13053 | hg19 | chr9 | 139399318 | 1,4E+08 | 139399338 | 139399447 | 1 | Pool1 |
| CHP2_NOTCH1_1 | CACGCTTGAAGACCACGTTG | GGACTGTGCGGAGCATGTA | GENOME_REGION | COSM24673 | hg19 | chr9 | 139399318 | 1,4E+08 | 139399338 | 139399447 | 1 | Pool1 |
| CHP2_NOTCH1_1 | CACGCTTGAAGACCACGTTG | GGACTGTGCGGAGCATGTA | GENOME_REGION | COSM24888 | hg19 | chr9 | 139399318 | 1,4E+08 | 139399338 | 139399447 | 1 | Pool1 |
| CHP2_NOTCH1_1 | CACGCTTGAAGACCACGTTG | GGACTGTGCGGAGCATGTA | GENOME_REGION | COSM25836 | hg19 | chr9 | 139399318 | 1,4E+08 | 139399338 | 139399447 | 1 | Pool1 |
| CHP2_NOTCH1_1 | CACGCTTGAAGACCACGTTG | GGACTGTGCGGAGCATGTA | GENOME_REGION | COSM25839 | hg19 | chr9 | 139399318 | 1,4E+08 | 139399338 | 139399447 | 1 | Pool1 |
| CHP2_NOTCH1_1 | CACGCTTGAAGACCACGTTG | GGACTGTGCGGAGCATGTA | GENOME_REGION | COSM28524 | hg19 | chr9 | 139399318 | 1,4E+08 | 139399338 | 139399447 | 1 | Pool1 |
| CHP2_NOTCH1_2 | ACACACTGCCGGTTGTCAA | CCTCACCATGTCCTGACTGTG | GENOME_REGION | COSM13040 | hg19 | chr9 | 139397744 | 1,4E+08 | 139397763 | 139397879 | 1 | Pool1 |
| CHP2_NRAS_1 | CCTCACCTCTATGGTGGGATCATAT | GTTCTTGCTGGTGTGAAATGACTG | GENOME_REGION | COSM12723 | hg19 | chr1 | 115258665 | 1,2E+08 | 115258690 | 115258774 | 1 | Pool1 |
| CHP2_NRAS_1 | CCTCACCTCTATGGTGGGATCATAT | GTTCTTGCTGGTGTGAAATGACTG | GENOME_REGION | COSM558 | hg19 | chr1 | 115258665 | 1,2E+08 | 115258690 | 115258774 | 1 | Pool1 |
| CHP2_NRAS_1 | CCTCACCTCTATGGTGGGATCATAT | GTTCTTGCTGGTGTGAAATGACTG | GENOME_REGION | COSM561 | hg19 | chr1 | 115258665 | 1,2E+08 | 115258690 | 115258774 | 1 | Pool1 |
| CHP2_NRAS_1 | CCTCACCTCTATGGTGGGATCATAT | GTTCTTGCTGGTGTGAAATGACTG | GENOME_REGION | COSM562 | hg19 | chr1 | 115258665 | 1,2E+08 | 115258690 | 115258774 | 1 | Pool1 |
| CHP2_NRAS_1 | CCTCACCTCTATGGTGGGATCATAT | GTTCTTGCTGGTGTGAAATGACTG | GENOME_REGION | COSM563 | hg19 | chr1 | 115258665 | 1,2E+08 | 115258690 | 115258774 | 1 | Pool1 |
| CHP2_NRAS_1 | CCTCACCTCTATGGTGGGATCATAT | GTTCTTGCTGGTGTGAAATGACTG | GENOME_REGION | COSM564 | hg19 | chr1 | 115258665 | 1,2E+08 | 115258690 | 115258774 | 1 | Pool1 |
| CHP2_NRAS_1 | CCTCACCTCTATGGTGGGATCATAT | GTTCTTGCTGGTGTGAAATGACTG | GENOME_REGION | COSM565 | hg19 | chr1 | 115258665 | 1,2E+08 | 115258690 | 115258774 | 1 | Pool1 |
| CHP2_NRAS_1 | CCTCACCTCTATGGTGGGATCATAT | GTTCTTGCTGGTGTGAAATGACTG | GENOME_REGION | COSM566 | hg19 | chr1 | 115258665 | 1,2E+08 | 115258690 | 115258774 | 1 | Pool1 |
| CHP2_NRAS_1 | CCTCACCTCTATGGTGGGATCATAT | GTTCTTGCTGGTGTGAAATGACTG | GENOME_REGION | COSM567 | hg19 | chr1 | 115258665 | 1,2E+08 | 115258690 | 115258774 | 1 | Pool1 |
| CHP2_NRAS_1 | CCTCACCTCTATGGTGGGATCATAT | GTTCTTGCTGGTGTGAAATGACTG | GENOME_REGION | COSM569 | hg19 | chr1 | 115258665 | 1,2E+08 | 115258690 | 115258774 | 1 | Pool1 |
| CHP2_NRAS_1 | CCTCACCTCTATGGTGGGATCATAT | GTTCTTGCTGGTGTGAAATGACTG | GENOME_REGION | COSM570 | hg19 | chr1 | 115258665 | 1,2E+08 | 115258690 | 115258774 | 1 | Pool1 |
| CHP2_NRAS_1 | CCTCACCTCTATGGTGGGATCATAT | GTTCTTGCTGGTGTGAAATGACTG | GENOME_REGION | COSM571 | hg19 | chr1 | 115258665 | 1,2E+08 | 115258690 | 115258774 | 1 | Pool1 |
| CHP2_NRAS_1 | CCTCACCTCTATGGTGGGATCATAT | GTTCTTGCTGGTGTGAAATGACTG | GENOME_REGION | COSM573 | hg19 | chr1 | 115258665 | 1,2E+08 | 115258690 | 115258774 | 1 | Pool1 |
| CHP2_NRAS_1 | CCTCACCTCTATGGTGGGATCATAT | GTTCTTGCTGGTGTGAAATGACTG | GENOME_REGION | COSM574 | hg19 | chr1 | 115258665 | 1,2E+08 | 115258690 | 115258774 | 1 | Pool1 |
| CHP2_NRAS_1 | CCTCACCTCTATGGTGGGATCATAT | GTTCTTGCTGGTGTGAAATGACTG | GENOME_REGION | COSM575 | hg19 | chr1 | 115258665 | 1,2E+08 | 115258690 | 115258774 | 1 | Pool1 |
| CHP2_NRAS_1 | CCTCACCTCTATGGTGGGATCATAT | GTTCTTGCTGGTGTGAAATGACTG | GENOME_REGION | COSM576 | hg19 | chr1 | 115258665 | 1,2E+08 | 115258690 | 115258774 | 1 | Pool1 |
| CHP2_NRAS_1 | CCTCACCTCTATGGTGGGATCATAT | GTTCTTGCTGGTGTGAAATGACTG | GENOME_REGION | COSM577 | hg19 | chr1 | 115258665 | 1,2E+08 | 115258690 | 115258774 | 1 | Pool1 |
| CHP2_NRAS_2 | TTCGCCTGTCCTCATGTATTGG | CACCCCCAGGATTCTTACAGAAAA | GENOME_REGION | COSM12725 | hg19 | chr1 | 115256483 | 1,2E+08 | 115256505 | 115256584 | 1 | Pool1 |
| CHP2_NRAS_2 | TTCGCCTGTCCTCATGTATTGG | CACCCCCAGGATTCTTACAGAAAA | GENOME_REGION | COSM12730 | hg19 | chr1 | 115256483 | 1,2E+08 | 115256505 | 115256584 | 1 | Pool1 |
| CHP2_NRAS_2 | TTCGCCTGTCCTCATGTATTGG | CACCCCCAGGATTCTTACAGAAAA | GENOME_REGION | COSM28673 | hg19 | chr1 | 115256483 | 1,2E+08 | 115256505 | 115256584 | 1 | Pool1 |
| CHP2_NRAS_2 | TTCGCCTGTCCTCATGTATTGG | CACCCCCAGGATTCTTACAGAAAA | GENOME_REGION | COSM30646 | hg19 | chr1 | 115256483 | 1,2E+08 | 115256505 | 115256584 | 1 | Pool1 |
| CHP2_NRAS_2 | TTCGCCTGTCCTCATGTATTGG | CACCCCCAGGATTCTTACAGAAAA | GENOME_REGION | COSM33693 | hg19 | chr1 | 115256483 | 1,2E+08 | 115256505 | 115256584 | 1 | Pool1 |
| CHP2_NRAS_2 | TTCGCCTGTCCTCATGTATTGG | CACCCCCAGGATTCTTACAGAAAA | GENOME_REGION | COSM579 | hg19 | chr1 | 115256483 | 1,2E+08 | 115256505 | 115256584 | 1 | Pool1 |
| CHP2_NRAS_2 | TTCGCCTGTCCTCATGTATTGG | CACCCCCAGGATTCTTACAGAAAA | GENOME_REGION | COSM580 | hg19 | chr1 | 115256483 | 1,2E+08 | 115256505 | 115256584 | 1 | Pool1 |
| CHP2_NRAS_2 | TTCGCCTGTCCTCATGTATTGG | CACCCCCAGGATTCTTACAGAAAA | GENOME_REGION | COSM581 | hg19 | chr1 | 115256483 | 1,2E+08 | 115256505 | 115256584 | 1 | Pool1 |
| CHP2_NRAS_2 | TTCGCCTGTCCTCATGTATTGG | CACCCCCAGGATTCTTACAGAAAA | GENOME_REGION | COSM582 | hg19 | chr1 | 115256483 | 1,2E+08 | 115256505 | 115256584 | 1 | Pool1 |
| CHP2_NRAS_2 | TTCGCCTGTCCTCATGTATTGG | CACCCCCAGGATTCTTACAGAAAA | GENOME_REGION | COSM583 | hg19 | chr1 | 115256483 | 1,2E+08 | 115256505 | 115256584 | 1 | Pool1 |
| CHP2_NRAS_2 | TTCGCCTGTCCTCATGTATTGG | CACCCCCAGGATTCTTACAGAAAA | GENOME_REGION | COSM584 | hg19 | chr1 | 115256483 | 1,2E+08 | 115256505 | 115256584 | 1 | Pool1 |
| CHP2_NRAS_2 | TTCGCCTGTCCTCATGTATTGG | CACCCCCAGGATTCTTACAGAAAA | GENOME_REGION | COSM585 | hg19 | chr1 | 115256483 | 1,2E+08 | 115256505 | 115256584 | 1 | Pool1 |
| CHP2_NRAS_2 | TTCGCCTGTCCTCATGTATTGG | CACCCCCAGGATTCTTACAGAAAA | GENOME_REGION | COSM586 | hg19 | chr1 | 115256483 | 1,2E+08 | 115256505 | 115256584 | 1 | Pool1 |
| CHP2_NRAS_2 | TTCGCCTGTCCTCATGTATTGG | CACCCCCAGGATTCTTACAGAAAA | GENOME_REGION | COSM587 | hg19 | chr1 | 115256483 | 1,2E+08 | 115256505 | 115256584 | 1 | Pool1 |
| CHP2_NRAS_2 | TTCGCCTGTCCTCATGTATTGG | CACCCCCAGGATTCTTACAGAAAA | GENOME_REGION | COSM589 | hg19 | chr1 | 115256483 | 1,2E+08 | 115256505 | 115256584 | 1 | Pool1 |
| CHP2_PIK3CA_10 | TGGAATGCCAGAACTACAATCTTTTGAT | GTGGAAGATCCAATCCATTTTTGTTGTC | GENOME_REGION | COSM12461 | hg19 | chr3 | 178951969 | 1,8E+08 | 178951997 | 178952097 | 1 | Pool1 |
| CHP2_PIK3CA_10 | TGGAATGCCAGAACTACAATCTTTTGAT | GTGGAAGATCCAATCCATTTTTGTTGTC | GENOME_REGION | COSM12463 | hg19 | chr3 | 178951969 | 1,8E+08 | 178951997 | 178952097 | 1 | Pool1 |
| CHP2_PIK3CA_10 | TGGAATGCCAGAACTACAATCTTTTGAT | GTGGAAGATCCAATCCATTTTTGTTGTC | GENOME_REGION | COSM12590 | hg19 | chr3 | 178951969 | 1,8E+08 | 178951997 | 178952097 | 1 | Pool1 |
| CHP2_PIK3CA_10 | TGGAATGCCAGAACTACAATCTTTTGAT | GTGGAAGATCCAATCCATTTTTGTTGTC | GENOME_REGION | COSM12591 | hg19 | chr3 | 178951969 | 1,8E+08 | 178951997 | 178952097 | 1 | Pool1 |
| CHP2_PIK3CA_10 | TGGAATGCCAGAACTACAATCTTTTGAT | GTGGAAGATCCAATCCATTTTTGTTGTC | GENOME_REGION | COSM12592 | hg19 | chr3 | 178951969 | 1,8E+08 | 178951997 | 178952097 | 1 | Pool1 |
| CHP2_PIK3CA_10 | TGGAATGCCAGAACTACAATCTTTTGAT | GTGGAAGATCCAATCCATTTTTGTTGTC | GENOME_REGION | COSM12597 | hg19 | chr3 | 178951969 | 1,8E+08 | 178951997 | 178952097 | 1 | Pool1 |
| CHP2_PIK3CA_10 | TGGAATGCCAGAACTACAATCTTTTGAT | GTGGAAGATCCAATCCATTTTTGTTGTC | GENOME_REGION | COSM13594 | hg19 | chr3 | 178951969 | 1,8E+08 | 178951997 | 178952097 | 1 | Pool1 |
| CHP2_PIK3CA_10 | TGGAATGCCAGAACTACAATCTTTTGAT | GTGGAAGATCCAATCCATTTTTGTTGTC | GENOME_REGION | COSM17444 | hg19 | chr3 | 178951969 | 1,8E+08 | 178951997 | 178952097 | 1 | Pool1 |
| CHP2_PIK3CA_10 | TGGAATGCCAGAACTACAATCTTTTGAT | GTGGAAGATCCAATCCATTTTTGTTGTC | GENOME_REGION | COSM17445 | hg19 | chr3 | 178951969 | 1,8E+08 | 178951997 | 178952097 | 1 | Pool1 |
| CHP2_PIK3CA_10 | TGGAATGCCAGAACTACAATCTTTTGAT | GTGGAAGATCCAATCCATTTTTGTTGTC | GENOME_REGION | COSM21451 | hg19 | chr3 | 178951969 | 1,8E+08 | 178951997 | 178952097 | 1 | Pool1 |
| CHP2_PIK3CA_10 | TGGAATGCCAGAACTACAATCTTTTGAT | GTGGAAGATCCAATCCATTTTTGTTGTC | GENOME_REGION | COSM24714 | hg19 | chr3 | 178951969 | 1,8E+08 | 178951997 | 178952097 | 1 | Pool1 |
| CHP2_PIK3CA_10 | TGGAATGCCAGAACTACAATCTTTTGAT | GTGGAAGATCCAATCCATTTTTGTTGTC | GENOME_REGION | COSM25085 | hg19 | chr3 | 178951969 | 1,8E+08 | 178951997 | 178952097 | 1 | Pool1 |
| CHP2_PIK3CA_10 | TGGAATGCCAGAACTACAATCTTTTGAT | GTGGAAGATCCAATCCATTTTTGTTGTC | GENOME_REGION | COSM25086 | hg19 | chr3 | 178951969 | 1,8E+08 | 178951997 | 178952097 | 1 | Pool1 |
| CHP2_PIK3CA_10 | TGGAATGCCAGAACTACAATCTTTTGAT | GTGGAAGATCCAATCCATTTTTGTTGTC | GENOME_REGION | COSM27134 | hg19 | chr3 | 178951969 | 1,8E+08 | 178951997 | 178952097 | 1 | Pool1 |
| CHP2_PIK3CA_10 | TGGAATGCCAGAACTACAATCTTTTGAT | GTGGAAGATCCAATCCATTTTTGTTGTC | GENOME_REGION | COSM27156 | hg19 | chr3 | 178951969 | 1,8E+08 | 178951997 | 178952097 | 1 | Pool1 |
| CHP2_PIK3CA_10 | TGGAATGCCAGAACTACAATCTTTTGAT | GTGGAAGATCCAATCCATTTTTGTTGTC | GENOME_REGION | COSM27158 | hg19 | chr3 | 178951969 | 1,8E+08 | 178951997 | 178952097 | 1 | Pool1 |
| CHP2_PIK3CA_10 | TGGAATGCCAGAACTACAATCTTTTGAT | GTGGAAGATCCAATCCATTTTTGTTGTC | GENOME_REGION | COSM27273 | hg19 | chr3 | 178951969 | 1,8E+08 | 178951997 | 178952097 | 1 | Pool1 |
| CHP2_PIK3CA_10 | TGGAATGCCAGAACTACAATCTTTTGAT | GTGGAAGATCCAATCCATTTTTGTTGTC | GENOME_REGION | COSM28938 | hg19 | chr3 | 178951969 | 1,8E+08 | 178951997 | 178952097 | 1 | Pool1 |
| CHP2_PIK3CA_10 | TGGAATGCCAGAACTACAATCTTTTGAT | GTGGAAGATCCAATCCATTTTTGTTGTC | GENOME_REGION | COSM29110 | hg19 | chr3 | 178951969 | 1,8E+08 | 178951997 | 178952097 | 1 | Pool1 |
| CHP2_PIK3CA_10 | TGGAATGCCAGAACTACAATCTTTTGAT | GTGGAAGATCCAATCCATTTTTGTTGTC | GENOME_REGION | COSM29313 | hg19 | chr3 | 178951969 | 1,8E+08 | 178951997 | 178952097 | 1 | Pool1 |
| CHP2_PIK3CA_10 | TGGAATGCCAGAACTACAATCTTTTGAT | GTGGAAGATCCAATCCATTTTTGTTGTC | GENOME_REGION | COSM36285 | hg19 | chr3 | 178951969 | 1,8E+08 | 178951997 | 178952097 | 1 | Pool1 |
| CHP2_PIK3CA_10 | TGGAATGCCAGAACTACAATCTTTTGAT | GTGGAAGATCCAATCCATTTTTGTTGTC | GENOME_REGION | COSM36286 | hg19 | chr3 | 178951969 | 1,8E+08 | 178951997 | 178952097 | 1 | Pool1 |
| CHP2_PIK3CA_10 | TGGAATGCCAGAACTACAATCTTTTGAT | GTGGAAGATCCAATCCATTTTTGTTGTC | GENOME_REGION | COSM36289 | hg19 | chr3 | 178951969 | 1,8E+08 | 178951997 | 178952097 | 1 | Pool1 |
| CHP2_PIK3CA_10 | TGGAATGCCAGAACTACAATCTTTTGAT | GTGGAAGATCCAATCCATTTTTGTTGTC | GENOME_REGION | COSM771 | hg19 | chr3 | 178951969 | 1,8E+08 | 178951997 | 178952097 | 1 | Pool1 |
| CHP2_PIK3CA_10 | TGGAATGCCAGAACTACAATCTTTTGAT | GTGGAAGATCCAATCCATTTTTGTTGTC | GENOME_REGION | COSM772 | hg19 | chr3 | 178951969 | 1,8E+08 | 178951997 | 178952097 | 1 | Pool1 |
| CHP2_PIK3CA_10 | TGGAATGCCAGAACTACAATCTTTTGAT | GTGGAAGATCCAATCCATTTTTGTTGTC | GENOME_REGION | COSM773 | hg19 | chr3 | 178951969 | 1,8E+08 | 178951997 | 178952097 | 1 | Pool1 |
| CHP2_PIK3CA_10 | TGGAATGCCAGAACTACAATCTTTTGAT | GTGGAAGATCCAATCCATTTTTGTTGTC | GENOME_REGION | COSM774 | hg19 | chr3 | 178951969 | 1,8E+08 | 178951997 | 178952097 | 1 | Pool1 |
| CHP2_PIK3CA_10 | TGGAATGCCAGAACTACAATCTTTTGAT | GTGGAAGATCCAATCCATTTTTGTTGTC | GENOME_REGION | COSM775 | hg19 | chr3 | 178951969 | 1,8E+08 | 178951997 | 178952097 | 1 | Pool1 |
| CHP2_PIK3CA_10 | TGGAATGCCAGAACTACAATCTTTTGAT | GTGGAAGATCCAATCCATTTTTGTTGTC | GENOME_REGION | COSM776 | hg19 | chr3 | 178951969 | 1,8E+08 | 178951997 | 178952097 | 1 | Pool1 |
| CHP2_PIK3CA_10 | TGGAATGCCAGAACTACAATCTTTTGAT | GTGGAAGATCCAATCCATTTTTGTTGTC | GENOME_REGION | COSM777 | hg19 | chr3 | 178951969 | 1,8E+08 | 178951997 | 178952097 | 1 | Pool1 |
| CHP2_PIK3CA_10 | TGGAATGCCAGAACTACAATCTTTTGAT | GTGGAAGATCCAATCCATTTTTGTTGTC | GENOME_REGION | COSM94984 | hg19 | chr3 | 178951969 | 1,8E+08 | 178951997 | 178952097 | 1 | Pool1 |
| CHP2_PIK3CA_10 | TGGAATGCCAGAACTACAATCTTTTGAT | GTGGAAGATCCAATCCATTTTTGTTGTC | GENOME_REGION | COSM94985 | hg19 | chr3 | 178951969 | 1,8E+08 | 178951997 | 178952097 | 1 | Pool1 |
| CHP2_PIK3CA_10 | TGGAATGCCAGAACTACAATCTTTTGAT | GTGGAAGATCCAATCCATTTTTGTTGTC | GENOME_REGION | COSM94986 | hg19 | chr3 | 178951969 | 1,8E+08 | 178951997 | 178952097 | 1 | Pool1 |
| CHP2_PIK3CA_10 | TGGAATGCCAGAACTACAATCTTTTGAT | GTGGAAGATCCAATCCATTTTTGTTGTC | GENOME_REGION | COSM94987 | hg19 | chr3 | 178951969 | 1,8E+08 | 178951997 | 178952097 | 1 | Pool1 |
| CHP2_PIK3CA_11 | TGGATCTTCCACACAATTAAACAGCAT | TGCTGTTCATGGATTGTGCAATTC | GENOME_REGION | COSM17449 | hg19 | chr3 | 178952114 | 1,8E+08 | 178952141 | 178952237 | 1 | Pool1 |
| CHP2_PIK3CA_11 | TGGATCTTCCACACAATTAAACAGCAT | TGCTGTTCATGGATTGTGCAATTC | GENOME_REGION | COSM249908 | hg19 | chr3 | 178952114 | 1,8E+08 | 178952141 | 178952237 | 1 | Pool1 |
| CHP2_PIK3CA_7 | CAGAGTAACAGACTAGCTAGAGACAATGA | GCACTTACCTGTGACTCCATAGAAA | GENOME_REGION | COSM12458 | hg19 | chr3 | 178935995 | 1,8E+08 | 178936024 | 178936105 | 1 | Pool1 |
| CHP2_PIK3CA_7 | CAGAGTAACAGACTAGCTAGAGACAATGA | GCACTTACCTGTGACTCCATAGAAA | GENOME_REGION | COSM12459 | hg19 | chr3 | 178935995 | 1,8E+08 | 178936024 | 178936105 | 1 | Pool1 |
| CHP2_PIK3CA_7 | CAGAGTAACAGACTAGCTAGAGACAATGA | GCACTTACCTGTGACTCCATAGAAA | GENOME_REGION | COSM125370 | hg19 | chr3 | 178935995 | 1,8E+08 | 178936024 | 178936105 | 1 | Pool1 |
| CHP2_PIK3CA_7 | CAGAGTAACAGACTAGCTAGAGACAATGA | GCACTTACCTGTGACTCCATAGAAA | GENOME_REGION | COSM17442 | hg19 | chr3 | 178935995 | 1,8E+08 | 178936024 | 178936105 | 1 | Pool1 |
| CHP2_PIK3CA_7 | CAGAGTAACAGACTAGCTAGAGACAATGA | GCACTTACCTGTGACTCCATAGAAA | GENOME_REGION | COSM24712 | hg19 | chr3 | 178935995 | 1,8E+08 | 178936024 | 178936105 | 1 | Pool1 |
| CHP2_PIK3CA_7 | CAGAGTAACAGACTAGCTAGAGACAATGA | GCACTTACCTGTGACTCCATAGAAA | GENOME_REGION | COSM249872 | hg19 | chr3 | 178935995 | 1,8E+08 | 178936024 | 178936105 | 1 | Pool1 |
| CHP2_PIK3CA_7 | CAGAGTAACAGACTAGCTAGAGACAATGA | GCACTTACCTGTGACTCCATAGAAA | GENOME_REGION | COSM25041 | hg19 | chr3 | 178935995 | 1,8E+08 | 178936024 | 178936105 | 1 | Pool1 |
| CHP2_PIK3CA_7 | CAGAGTAACAGACTAGCTAGAGACAATGA | GCACTTACCTGTGACTCCATAGAAA | GENOME_REGION | COSM27133 | hg19 | chr3 | 178935995 | 1,8E+08 | 178936024 | 178936105 | 1 | Pool1 |
| CHP2_PIK3CA_7 | CAGAGTAACAGACTAGCTAGAGACAATGA | GCACTTACCTGTGACTCCATAGAAA | GENOME_REGION | COSM27155 | hg19 | chr3 | 178935995 | 1,8E+08 | 178936024 | 178936105 | 1 | Pool1 |
| CHP2_PIK3CA_7 | CAGAGTAACAGACTAGCTAGAGACAATGA | GCACTTACCTGTGACTCCATAGAAA | GENOME_REGION | COSM27374 | hg19 | chr3 | 178935995 | 1,8E+08 | 178936024 | 178936105 | 1 | Pool1 |
| CHP2_PIK3CA_7 | CAGAGTAACAGACTAGCTAGAGACAATGA | GCACTTACCTGTGACTCCATAGAAA | GENOME_REGION | COSM6147 | hg19 | chr3 | 178935995 | 1,8E+08 | 178936024 | 178936105 | 1 | Pool1 |
| CHP2_PIK3CA_7 | CAGAGTAACAGACTAGCTAGAGACAATGA | GCACTTACCTGTGACTCCATAGAAA | GENOME_REGION | COSM759 | hg19 | chr3 | 178935995 | 1,8E+08 | 178936024 | 178936105 | 1 | Pool1 |
| CHP2_PIK3CA_7 | CAGAGTAACAGACTAGCTAGAGACAATGA | GCACTTACCTGTGACTCCATAGAAA | GENOME_REGION | COSM760 | hg19 | chr3 | 178935995 | 1,8E+08 | 178936024 | 178936105 | 1 | Pool1 |
| CHP2_PIK3CA_7 | CAGAGTAACAGACTAGCTAGAGACAATGA | GCACTTACCTGTGACTCCATAGAAA | GENOME_REGION | COSM762 | hg19 | chr3 | 178935995 | 1,8E+08 | 178936024 | 178936105 | 1 | Pool1 |
| CHP2_PIK3CA_7 | CAGAGTAACAGACTAGCTAGAGACAATGA | GCACTTACCTGTGACTCCATAGAAA | GENOME_REGION | COSM763 | hg19 | chr3 | 178935995 | 1,8E+08 | 178936024 | 178936105 | 1 | Pool1 |
| CHP2_PIK3CA_7 | CAGAGTAACAGACTAGCTAGAGACAATGA | GCACTTACCTGTGACTCCATAGAAA | GENOME_REGION | COSM764 | hg19 | chr3 | 178935995 | 1,8E+08 | 178936024 | 178936105 | 1 | Pool1 |
| CHP2_PIK3CA_7 | CAGAGTAACAGACTAGCTAGAGACAATGA | GCACTTACCTGTGACTCCATAGAAA | GENOME_REGION | COSM765 | hg19 | chr3 | 178935995 | 1,8E+08 | 178936024 | 178936105 | 1 | Pool1 |
| CHP2_PIK3CA_7 | CAGAGTAACAGACTAGCTAGAGACAATGA | GCACTTACCTGTGACTCCATAGAAA | GENOME_REGION | COSM766 | hg19 | chr3 | 178935995 | 1,8E+08 | 178936024 | 178936105 | 1 | Pool1 |
| CHP2_PIK3CA_7 | CAGAGTAACAGACTAGCTAGAGACAATGA | GCACTTACCTGTGACTCCATAGAAA | GENOME_REGION | COSM767 | hg19 | chr3 | 178935995 | 1,8E+08 | 178936024 | 178936105 | 1 | Pool1 |
| CHP2_PIK3CA_8 | CACGATTCTTTTAGATCTGAGATGCACA | CCTTTTGTGTTTCATCCTTCTTCTCCTG | GENOME_REGION | COSM778 | hg19 | chr3 | 178938760 | 1,8E+08 | 178938788 | 178938918 | 1 | Pool1 |
| CHP2_PTEN_1 | GCCATCTCTCTCCTCCTTTTTCTT | GCCGCAGAAATGGATACAGGTC | GENOME_REGION | COSM4929 | hg19 | chr10 | 89624184 | 9E+07 | 89624208 | 89624300 | 1 | Pool1 |
| CHP2_PTEN_1 | GCCATCTCTCTCCTCCTTTTTCTT | GCCGCAGAAATGGATACAGGTC | GENOME_REGION | COSM4937 | hg19 | chr10 | 89624184 | 9E+07 | 89624208 | 89624300 | 1 | Pool1 |
| CHP2_PTEN_1 | GCCATCTCTCTCCTCCTTTTTCTT | GCCGCAGAAATGGATACAGGTC | GENOME_REGION | COSM4976 | hg19 | chr10 | 89624184 | 9E+07 | 89624208 | 89624300 | 1 | Pool1 |
| CHP2_PTEN_1 | GCCATCTCTCTCCTCCTTTTTCTT | GCCGCAGAAATGGATACAGGTC | GENOME_REGION | COSM5037 | hg19 | chr10 | 89624184 | 9E+07 | 89624208 | 89624300 | 1 | Pool1 |
| CHP2_PTEN_1 | GCCATCTCTCTCCTCCTTTTTCTT | GCCGCAGAAATGGATACAGGTC | GENOME_REGION | COSM5049 | hg19 | chr10 | 89624184 | 9E+07 | 89624208 | 89624300 | 1 | Pool1 |
| CHP2_PTEN_1 | GCCATCTCTCTCCTCCTTTTTCTT | GCCGCAGAAATGGATACAGGTC | GENOME_REGION | COSM5101 | hg19 | chr10 | 89624184 | 9E+07 | 89624208 | 89624300 | 1 | Pool1 |
| CHP2_PTEN_1 | GCCATCTCTCTCCTCCTTTTTCTT | GCCGCAGAAATGGATACAGGTC | GENOME_REGION | COSM5133 | hg19 | chr10 | 89624184 | 9E+07 | 89624208 | 89624300 | 1 | Pool1 |
| CHP2_PTEN_1 | GCCATCTCTCTCCTCCTTTTTCTT | GCCGCAGAAATGGATACAGGTC | GENOME_REGION | COSM5153 | hg19 | chr10 | 89624184 | 9E+07 | 89624208 | 89624300 | 1 | Pool1 |
| CHP2_PTEN_1 | GCCATCTCTCTCCTCCTTTTTCTT | GCCGCAGAAATGGATACAGGTC | GENOME_REGION | COSM5232 | hg19 | chr10 | 89624184 | 9E+07 | 89624208 | 89624300 | 1 | Pool1 |
| CHP2_PTEN_1 | GCCATCTCTCTCCTCCTTTTTCTT | GCCGCAGAAATGGATACAGGTC | GENOME_REGION | COSM5270 | hg19 | chr10 | 89624184 | 9E+07 | 89624208 | 89624300 | 1 | Pool1 |
| CHP2_PTEN_1 | GCCATCTCTCTCCTCCTTTTTCTT | GCCGCAGAAATGGATACAGGTC | GENOME_REGION | COSM5298 | hg19 | chr10 | 89624184 | 9E+07 | 89624208 | 89624300 | 1 | Pool1 |
| CHP2_PTEN_1 | GCCATCTCTCTCCTCCTTTTTCTT | GCCGCAGAAATGGATACAGGTC | GENOME_REGION | COSM5878 | hg19 | chr10 | 89624184 | 9E+07 | 89624208 | 89624300 | 1 | Pool1 |
| CHP2_PTEN_1 | GCCATCTCTCTCCTCCTTTTTCTT | GCCGCAGAAATGGATACAGGTC | GENOME_REGION | COSM5915 | hg19 | chr10 | 89624184 | 9E+07 | 89624208 | 89624300 | 1 | Pool1 |
| CHP2_PTEN_2 | TGTTAATGGTGGCTTTTTGTTTGTTTGT | TCTACCTCACTCTAACAAGCAGATAACT | GENOME_REGION | COSM14087 | hg19 | chr10 | 89685231 | 9E+07 | 89685259 | 89685374 | 1 | Pool1 |
| CHP2_PTEN_2 | TGTTAATGGTGGCTTTTTGTTTGTTTGT | TCTACCTCACTCTAACAAGCAGATAACT | GENOME_REGION | COSM41768 | hg19 | chr10 | 89685231 | 9E+07 | 89685259 | 89685374 | 1 | Pool1 |
| CHP2_PTEN_2 | TGTTAATGGTGGCTTTTTGTTTGTTTGT | TCTACCTCACTCTAACAAGCAGATAACT | GENOME_REGION | COSM43077 | hg19 | chr10 | 89685231 | 9E+07 | 89685259 | 89685374 | 1 | Pool1 |
| CHP2_PTEN_2 | TGTTAATGGTGGCTTTTTGTTTGTTTGT | TCTACCTCACTCTAACAAGCAGATAACT | GENOME_REGION | COSM4889 | hg19 | chr10 | 89685231 | 9E+07 | 89685259 | 89685374 | 1 | Pool1 |
| CHP2_PTEN_2 | TGTTAATGGTGGCTTTTTGTTTGTTTGT | TCTACCTCACTCTAACAAGCAGATAACT | GENOME_REGION | COSM4942 | hg19 | chr10 | 89685231 | 9E+07 | 89685259 | 89685374 | 1 | Pool1 |
| CHP2_PTEN_2 | TGTTAATGGTGGCTTTTTGTTTGTTTGT | TCTACCTCACTCTAACAAGCAGATAACT | GENOME_REGION | COSM5000 | hg19 | chr10 | 89685231 | 9E+07 | 89685259 | 89685374 | 1 | Pool1 |
| CHP2_PTEN_2 | TGTTAATGGTGGCTTTTTGTTTGTTTGT | TCTACCTCACTCTAACAAGCAGATAACT | GENOME_REGION | COSM5036 | hg19 | chr10 | 89685231 | 9E+07 | 89685259 | 89685374 | 1 | Pool1 |
| CHP2_PTEN_2 | TGTTAATGGTGGCTTTTTGTTTGTTTGT | TCTACCTCACTCTAACAAGCAGATAACT | GENOME_REGION | COSM5042 | hg19 | chr10 | 89685231 | 9E+07 | 89685259 | 89685374 | 1 | Pool1 |
| CHP2_PTEN_2 | TGTTAATGGTGGCTTTTTGTTTGTTTGT | TCTACCTCACTCTAACAAGCAGATAACT | GENOME_REGION | COSM5048 | hg19 | chr10 | 89685231 | 9E+07 | 89685259 | 89685374 | 1 | Pool1 |
| CHP2_PTEN_2 | TGTTAATGGTGGCTTTTTGTTTGTTTGT | TCTACCTCACTCTAACAAGCAGATAACT | GENOME_REGION | COSM5127 | hg19 | chr10 | 89685231 | 9E+07 | 89685259 | 89685374 | 1 | Pool1 |
| CHP2_PTEN_2 | TGTTAATGGTGGCTTTTTGTTTGTTTGT | TCTACCTCACTCTAACAAGCAGATAACT | GENOME_REGION | COSM5191 | hg19 | chr10 | 89685231 | 9E+07 | 89685259 | 89685374 | 1 | Pool1 |
| CHP2_PTEN_2 | TGTTAATGGTGGCTTTTTGTTTGTTTGT | TCTACCTCACTCTAACAAGCAGATAACT | GENOME_REGION | COSM5253 | hg19 | chr10 | 89685231 | 9E+07 | 89685259 | 89685374 | 1 | Pool1 |
| CHP2_PTEN_2 | TGTTAATGGTGGCTTTTTGTTTGTTTGT | TCTACCTCACTCTAACAAGCAGATAACT | GENOME_REGION | COSM5257 | hg19 | chr10 | 89685231 | 9E+07 | 89685259 | 89685374 | 1 | Pool1 |
| CHP2_PTEN_2 | TGTTAATGGTGGCTTTTTGTTTGTTTGT | TCTACCTCACTCTAACAAGCAGATAACT | GENOME_REGION | COSM5296 | hg19 | chr10 | 89685231 | 9E+07 | 89685259 | 89685374 | 1 | Pool1 |
| CHP2_PTEN_2 | TGTTAATGGTGGCTTTTTGTTTGTTTGT | TCTACCTCACTCTAACAAGCAGATAACT | GENOME_REGION | COSM5313 | hg19 | chr10 | 89685231 | 9E+07 | 89685259 | 89685374 | 1 | Pool1 |
| CHP2_PTEN_2 | TGTTAATGGTGGCTTTTTGTTTGTTTGT | TCTACCTCACTCTAACAAGCAGATAACT | GENOME_REGION | COSM5317 | hg19 | chr10 | 89685231 | 9E+07 | 89685259 | 89685374 | 1 | Pool1 |
| CHP2_PTEN_2 | TGTTAATGGTGGCTTTTTGTTTGTTTGT | TCTACCTCACTCTAACAAGCAGATAACT | GENOME_REGION | COSM5811 | hg19 | chr10 | 89685231 | 9E+07 | 89685259 | 89685374 | 1 | Pool1 |
| CHP2_PTEN_2 | TGTTAATGGTGGCTTTTTGTTTGTTTGT | TCTACCTCACTCTAACAAGCAGATAACT | GENOME_REGION | COSM5916 | hg19 | chr10 | 89685231 | 9E+07 | 89685259 | 89685374 | 1 | Pool1 |
| CHP2_PTEN_2 | TGTTAATGGTGGCTTTTTGTTTGTTTGT | TCTACCTCACTCTAACAAGCAGATAACT | GENOME_REGION | COSM5958 | hg19 | chr10 | 89685231 | 9E+07 | 89685259 | 89685374 | 1 | Pool1 |
| CHP2_PTEN_2 | TGTTAATGGTGGCTTTTTGTTTGTTTGT | TCTACCTCACTCTAACAAGCAGATAACT | GENOME_REGION | COSM5959 | hg19 | chr10 | 89685231 | 9E+07 | 89685259 | 89685374 | 1 | Pool1 |
| CHP2_PTEN_2 | TGTTAATGGTGGCTTTTTGTTTGTTTGT | TCTACCTCACTCTAACAAGCAGATAACT | GENOME_REGION | COSM5960 | hg19 | chr10 | 89685231 | 9E+07 | 89685259 | 89685374 | 1 | Pool1 |
| CHP2_PTEN_2 | TGTTAATGGTGGCTTTTTGTTTGTTTGT | TCTACCTCACTCTAACAAGCAGATAACT | GENOME_REGION | COSM5974 | hg19 | chr10 | 89685231 | 9E+07 | 89685259 | 89685374 | 1 | Pool1 |
| CHP2_PTEN_2 | TGTTAATGGTGGCTTTTTGTTTGTTTGT | TCTACCTCACTCTAACAAGCAGATAACT | GENOME_REGION | COSM5975 | hg19 | chr10 | 89685231 | 9E+07 | 89685259 | 89685374 | 1 | Pool1 |
| CHP2_PTEN_2 | TGTTAATGGTGGCTTTTTGTTTGTTTGT | TCTACCTCACTCTAACAAGCAGATAACT | GENOME_REGION | COSM5976 | hg19 | chr10 | 89685231 | 9E+07 | 89685259 | 89685374 | 1 | Pool1 |
| CHP2_PTEN_2 | TGTTAATGGTGGCTTTTTGTTTGTTTGT | TCTACCTCACTCTAACAAGCAGATAACT | GENOME_REGION | COSM5979 | hg19 | chr10 | 89685231 | 9E+07 | 89685259 | 89685374 | 1 | Pool1 |
| CHP2_PTEN_4 | GGCTACGACCCAGTTACCATAG | TGCCACTGGTCTATAATCCAGATGAT | GENOME_REGION | COSM28897 | hg19 | chr10 | 89711783 | 9E+07 | 89711805 | 89711932 | 1 | Pool1 |
| CHP2_PTEN_4 | GGCTACGACCCAGTTACCATAG | TGCCACTGGTCTATAATCCAGATGAT | GENOME_REGION | COSM33702 | hg19 | chr10 | 89711783 | 9E+07 | 89711805 | 89711932 | 1 | Pool1 |
| CHP2_PTEN_4 | GGCTACGACCCAGTTACCATAG | TGCCACTGGTCTATAATCCAGATGAT | GENOME_REGION | COSM4969 | hg19 | chr10 | 89711783 | 9E+07 | 89711805 | 89711932 | 1 | Pool1 |
| CHP2_PTEN_4 | GGCTACGACCCAGTTACCATAG | TGCCACTGGTCTATAATCCAGATGAT | GENOME_REGION | COSM5039 | hg19 | chr10 | 89711783 | 9E+07 | 89711805 | 89711932 | 1 | Pool1 |
| CHP2_PTEN_4 | GGCTACGACCCAGTTACCATAG | TGCCACTGGTCTATAATCCAGATGAT | GENOME_REGION | COSM5045 | hg19 | chr10 | 89711783 | 9E+07 | 89711805 | 89711932 | 1 | Pool1 |
| CHP2_PTEN_4 | GGCTACGACCCAGTTACCATAG | TGCCACTGGTCTATAATCCAGATGAT | GENOME_REGION | COSM5052 | hg19 | chr10 | 89711783 | 9E+07 | 89711805 | 89711932 | 1 | Pool1 |
| CHP2_PTEN_4 | GGCTACGACCCAGTTACCATAG | TGCCACTGGTCTATAATCCAGATGAT | GENOME_REGION | COSM5089 | hg19 | chr10 | 89711783 | 9E+07 | 89711805 | 89711932 | 1 | Pool1 |
| CHP2_PTEN_4 | GGCTACGACCCAGTTACCATAG | TGCCACTGGTCTATAATCCAGATGAT | GENOME_REGION | COSM5091 | hg19 | chr10 | 89711783 | 9E+07 | 89711805 | 89711932 | 1 | Pool1 |
| CHP2_PTEN_4 | GGCTACGACCCAGTTACCATAG | TGCCACTGGTCTATAATCCAGATGAT | GENOME_REGION | COSM5114 | hg19 | chr10 | 89711783 | 9E+07 | 89711805 | 89711932 | 1 | Pool1 |
| CHP2_PTEN_4 | GGCTACGACCCAGTTACCATAG | TGCCACTGGTCTATAATCCAGATGAT | GENOME_REGION | COSM5149 | hg19 | chr10 | 89711783 | 9E+07 | 89711805 | 89711932 | 1 | Pool1 |
| CHP2_PTEN_4 | GGCTACGACCCAGTTACCATAG | TGCCACTGGTCTATAATCCAGATGAT | GENOME_REGION | COSM5200 | hg19 | chr10 | 89711783 | 9E+07 | 89711805 | 89711932 | 1 | Pool1 |
| CHP2_PTEN_4 | GGCTACGACCCAGTTACCATAG | TGCCACTGGTCTATAATCCAGATGAT | GENOME_REGION | COSM5218 | hg19 | chr10 | 89711783 | 9E+07 | 89711805 | 89711932 | 1 | Pool1 |
| CHP2_PTEN_4 | GGCTACGACCCAGTTACCATAG | TGCCACTGGTCTATAATCCAGATGAT | GENOME_REGION | COSM5244 | hg19 | chr10 | 89711783 | 9E+07 | 89711805 | 89711932 | 1 | Pool1 |
| CHP2_PTEN_4 | GGCTACGACCCAGTTACCATAG | TGCCACTGGTCTATAATCCAGATGAT | GENOME_REGION | COSM5825 | hg19 | chr10 | 89711783 | 9E+07 | 89711805 | 89711932 | 1 | Pool1 |
| CHP2_PTEN_4 | GGCTACGACCCAGTTACCATAG | TGCCACTGGTCTATAATCCAGATGAT | GENOME_REGION | COSM5907 | hg19 | chr10 | 89711783 | 9E+07 | 89711805 | 89711932 | 1 | Pool1 |
| CHP2_PTEN_4 | GGCTACGACCCAGTTACCATAG | TGCCACTGGTCTATAATCCAGATGAT | GENOME_REGION | COSM5961 | hg19 | chr10 | 89711783 | 9E+07 | 89711805 | 89711932 | 1 | Pool1 |
| CHP2_PTEN_5 | TGAGATCAAGATTGCAGATACAGAATCC | ACCTTTAGCTGGCAGACCAC | GENOME_REGION | COSM17561 | hg19 | chr10 | 89717476 | 9E+07 | 89717504 | 89717620 | 1 | Pool1 |
| CHP2_PTEN_5 | TGAGATCAAGATTGCAGATACAGAATCC | ACCTTTAGCTGGCAGACCAC | GENOME_REGION | COSM241294 | hg19 | chr10 | 89717476 | 9E+07 | 89717504 | 89717620 | 1 | Pool1 |
| CHP2_PTEN_5 | TGAGATCAAGATTGCAGATACAGAATCC | ACCTTTAGCTGGCAGACCAC | GENOME_REGION | COSM249834 | hg19 | chr10 | 89717476 | 9E+07 | 89717504 | 89717620 | 1 | Pool1 |
| CHP2_PTEN_5 | TGAGATCAAGATTGCAGATACAGAATCC | ACCTTTAGCTGGCAGACCAC | GENOME_REGION | COSM27365 | hg19 | chr10 | 89717476 | 9E+07 | 89717504 | 89717620 | 1 | Pool1 |
| CHP2_PTEN_5 | TGAGATCAAGATTGCAGATACAGAATCC | ACCTTTAGCTGGCAGACCAC | GENOME_REGION | COSM28920 | hg19 | chr10 | 89717476 | 9E+07 | 89717504 | 89717620 | 1 | Pool1 |
| CHP2_PTEN_5 | TGAGATCAAGATTGCAGATACAGAATCC | ACCTTTAGCTGGCAGACCAC | GENOME_REGION | COSM30729 | hg19 | chr10 | 89717476 | 9E+07 | 89717504 | 89717620 | 1 | Pool1 |
| CHP2_PTEN_5 | TGAGATCAAGATTGCAGATACAGAATCC | ACCTTTAGCTGGCAGACCAC | GENOME_REGION | COSM5932 | hg19 | chr10 | 89717476 | 9E+07 | 89717504 | 89717620 | 1 | Pool1 |
| CHP2_PTEN_5 | TGAGATCAAGATTGCAGATACAGAATCC | ACCTTTAGCTGGCAGACCAC | GENOME_REGION | COSM5950 | hg19 | chr10 | 89717476 | 9E+07 | 89717504 | 89717620 | 1 | Pool1 |
| CHP2_PTEN_5 | TGAGATCAAGATTGCAGATACAGAATCC | ACCTTTAGCTGGCAGACCAC | GENOME_REGION | COSM5951 | hg19 | chr10 | 89717476 | 9E+07 | 89717504 | 89717620 | 1 | Pool1 |
| CHP2_PTEN_5 | TGAGATCAAGATTGCAGATACAGAATCC | ACCTTTAGCTGGCAGACCAC | GENOME_REGION | COSM5971 | hg19 | chr10 | 89717476 | 9E+07 | 89717504 | 89717620 | 1 | Pool1 |
| CHP2_PTEN_6 | AGGTGAAGATATATTCCTCCAATTCAGGAC | TTGGATATTTCTCCCAATGAAAGTAAAGTAC | GENOME_REGION | COSM133713 | hg19 | chr10 | 89717637 | 9E+07 | 89717667 | 89717780 | 1 | Pool1 |
| CHP2_PTEN_6 | AGGTGAAGATATATTCCTCCAATTCAGGAC | TTGGATATTTCTCCCAATGAAAGTAAAGTAC | GENOME_REGION | COSM13981 | hg19 | chr10 | 89717637 | 9E+07 | 89717667 | 89717780 | 1 | Pool1 |
| CHP2_PTEN_6 | AGGTGAAGATATATTCCTCCAATTCAGGAC | TTGGATATTTCTCCCAATGAAAGTAAAGTAC | GENOME_REGION | COSM17564 | hg19 | chr10 | 89717637 | 9E+07 | 89717667 | 89717780 | 1 | Pool1 |
| CHP2_PTEN_6 | AGGTGAAGATATATTCCTCCAATTCAGGAC | TTGGATATTTCTCCCAATGAAAGTAAAGTAC | GENOME_REGION | COSM23644 | hg19 | chr10 | 89717637 | 9E+07 | 89717667 | 89717780 | 1 | Pool1 |
| CHP2_PTEN_6 | AGGTGAAGATATATTCCTCCAATTCAGGAC | TTGGATATTTCTCCCAATGAAAGTAAAGTAC | GENOME_REGION | COSM26404 | hg19 | chr10 | 89717637 | 9E+07 | 89717667 | 89717780 | 1 | Pool1 |
| CHP2_PTEN_6 | AGGTGAAGATATATTCCTCCAATTCAGGAC | TTGGATATTTCTCCCAATGAAAGTAAAGTAC | GENOME_REGION | COSM43075 | hg19 | chr10 | 89717637 | 9E+07 | 89717667 | 89717780 | 1 | Pool1 |
| CHP2_PTEN_6 | AGGTGAAGATATATTCCTCCAATTCAGGAC | TTGGATATTTCTCCCAATGAAAGTAAAGTAC | GENOME_REGION | COSM4908 | hg19 | chr10 | 89717637 | 9E+07 | 89717667 | 89717780 | 1 | Pool1 |
| CHP2_PTEN_6 | AGGTGAAGATATATTCCTCCAATTCAGGAC | TTGGATATTTCTCCCAATGAAAGTAAAGTAC | GENOME_REGION | COSM4912 | hg19 | chr10 | 89717637 | 9E+07 | 89717667 | 89717780 | 1 | Pool1 |
| CHP2_PTEN_6 | AGGTGAAGATATATTCCTCCAATTCAGGAC | TTGGATATTTCTCCCAATGAAAGTAAAGTAC | GENOME_REGION | COSM4986 | hg19 | chr10 | 89717637 | 9E+07 | 89717667 | 89717780 | 1 | Pool1 |
| CHP2_PTEN_6 | AGGTGAAGATATATTCCTCCAATTCAGGAC | TTGGATATTTCTCCCAATGAAAGTAAAGTAC | GENOME_REGION | COSM5111 | hg19 | chr10 | 89717637 | 9E+07 | 89717667 | 89717780 | 1 | Pool1 |
| CHP2_PTEN_6 | AGGTGAAGATATATTCCTCCAATTCAGGAC | TTGGATATTTCTCCCAATGAAAGTAAAGTAC | GENOME_REGION | COSM5125 | hg19 | chr10 | 89717637 | 9E+07 | 89717667 | 89717780 | 1 | Pool1 |
| CHP2_PTEN_6 | AGGTGAAGATATATTCCTCCAATTCAGGAC | TTGGATATTTCTCCCAATGAAAGTAAAGTAC | GENOME_REGION | COSM5159 | hg19 | chr10 | 89717637 | 9E+07 | 89717667 | 89717780 | 1 | Pool1 |
| CHP2_PTEN_6 | AGGTGAAGATATATTCCTCCAATTCAGGAC | TTGGATATTTCTCCCAATGAAAGTAAAGTAC | GENOME_REGION | COSM5160 | hg19 | chr10 | 89717637 | 9E+07 | 89717667 | 89717780 | 1 | Pool1 |
| CHP2_PTEN_6 | AGGTGAAGATATATTCCTCCAATTCAGGAC | TTGGATATTTCTCCCAATGAAAGTAAAGTAC | GENOME_REGION | COSM5220 | hg19 | chr10 | 89717637 | 9E+07 | 89717667 | 89717780 | 1 | Pool1 |
| CHP2_PTEN_6 | AGGTGAAGATATATTCCTCCAATTCAGGAC | TTGGATATTTCTCCCAATGAAAGTAAAGTAC | GENOME_REGION | COSM5230 | hg19 | chr10 | 89717637 | 9E+07 | 89717667 | 89717780 | 1 | Pool1 |
| CHP2_PTEN_6 | AGGTGAAGATATATTCCTCCAATTCAGGAC | TTGGATATTTCTCCCAATGAAAGTAAAGTAC | GENOME_REGION | COSM5246 | hg19 | chr10 | 89717637 | 9E+07 | 89717667 | 89717780 | 1 | Pool1 |
| CHP2_PTEN_6 | AGGTGAAGATATATTCCTCCAATTCAGGAC | TTGGATATTTCTCCCAATGAAAGTAAAGTAC | GENOME_REGION | COSM5292 | hg19 | chr10 | 89717637 | 9E+07 | 89717667 | 89717780 | 1 | Pool1 |
| CHP2_PTEN_6 | AGGTGAAGATATATTCCTCCAATTCAGGAC | TTGGATATTTCTCCCAATGAAAGTAAAGTAC | GENOME_REGION | COSM5822 | hg19 | chr10 | 89717637 | 9E+07 | 89717667 | 89717780 | 1 | Pool1 |
| CHP2_PTEN_6 | AGGTGAAGATATATTCCTCCAATTCAGGAC | TTGGATATTTCTCCCAATGAAAGTAAAGTAC | GENOME_REGION | COSM5887 | hg19 | chr10 | 89717637 | 9E+07 | 89717667 | 89717780 | 1 | Pool1 |
| CHP2_PTEN_6 | AGGTGAAGATATATTCCTCCAATTCAGGAC | TTGGATATTTCTCCCAATGAAAGTAAAGTAC | GENOME_REGION | COSM5888 | hg19 | chr10 | 89717637 | 9E+07 | 89717667 | 89717780 | 1 | Pool1 |
| CHP2_PTEN_6 | AGGTGAAGATATATTCCTCCAATTCAGGAC | TTGGATATTTCTCCCAATGAAAGTAAAGTAC | GENOME_REGION | COSM88109 | hg19 | chr10 | 89717637 | 9E+07 | 89717667 | 89717780 | 1 | Pool1 |
| CHP2_PTEN_7 | CACTTTTGGGTAAATACATTCTTCATACCAGGA | CACGCTCTATACTGCAAATGCTATCGA | GENOME_REGION | COSM13452 | hg19 | chr10 | 89720663 | 9E+07 | 89720696 | 89720747 | 1 | Pool1 |
| CHP2_PTEN_7 | CACTTTTGGGTAAATACATTCTTCATACCAGGA | CACGCTCTATACTGCAAATGCTATCGA | GENOME_REGION | COSM28906 | hg19 | chr10 | 89720663 | 9E+07 | 89720696 | 89720747 | 1 | Pool1 |
| CHP2_PTEN_7 | CACTTTTGGGTAAATACATTCTTCATACCAGGA | CACGCTCTATACTGCAAATGCTATCGA | GENOME_REGION | COSM28914 | hg19 | chr10 | 89720663 | 9E+07 | 89720696 | 89720747 | 1 | Pool1 |
| CHP2_PTEN_7 | CACTTTTGGGTAAATACATTCTTCATACCAGGA | CACGCTCTATACTGCAAATGCTATCGA | GENOME_REGION | COSM4931 | hg19 | chr10 | 89720663 | 9E+07 | 89720696 | 89720747 | 1 | Pool1 |
| CHP2_PTEN_7 | CACTTTTGGGTAAATACATTCTTCATACCAGGA | CACGCTCTATACTGCAAATGCTATCGA | GENOME_REGION | COSM5156 | hg19 | chr10 | 89720663 | 9E+07 | 89720696 | 89720747 | 1 | Pool1 |
| CHP2_PTEN_7 | CACTTTTGGGTAAATACATTCTTCATACCAGGA | CACGCTCTATACTGCAAATGCTATCGA | GENOME_REGION | COSM5314 | hg19 | chr10 | 89720663 | 9E+07 | 89720696 | 89720747 | 1 | Pool1 |
| CHP2_PTEN_7 | CACTTTTGGGTAAATACATTCTTCATACCAGGA | CACGCTCTATACTGCAAATGCTATCGA | GENOME_REGION | COSM5816 | hg19 | chr10 | 89720663 | 9E+07 | 89720696 | 89720747 | 1 | Pool1 |
| CHP2_PTEN_8 | GCAGTATAGAGCGTGCAGATAATGA | CATCACATACATACAAGTCAACAACCC | GENOME_REGION | COSM19564 | hg19 | chr10 | 89720760 | 9E+07 | 89720785 | 89720900 | 1 | Pool1 |
| CHP2_PTEN_8 | GCAGTATAGAGCGTGCAGATAATGA | CATCACATACATACAAGTCAACAACCC | GENOME_REGION | COSM23626 | hg19 | chr10 | 89720760 | 9E+07 | 89720785 | 89720900 | 1 | Pool1 |
| CHP2_PTEN_8 | GCAGTATAGAGCGTGCAGATAATGA | CATCACATACATACAAGTCAACAACCC | GENOME_REGION | COSM23657 | hg19 | chr10 | 89720760 | 9E+07 | 89720785 | 89720900 | 1 | Pool1 |
| CHP2_PTEN_8 | GCAGTATAGAGCGTGCAGATAATGA | CATCACATACATACAAGTCAACAACCC | GENOME_REGION | COSM39615 | hg19 | chr10 | 89720760 | 9E+07 | 89720785 | 89720900 | 1 | Pool1 |
| CHP2_PTEN_8 | GCAGTATAGAGCGTGCAGATAATGA | CATCACATACATACAAGTCAACAACCC | GENOME_REGION | COSM4885 | hg19 | chr10 | 89720760 | 9E+07 | 89720785 | 89720900 | 1 | Pool1 |
| CHP2_PTEN_8 | GCAGTATAGAGCGTGCAGATAATGA | CATCACATACATACAAGTCAACAACCC | GENOME_REGION | COSM4894 | hg19 | chr10 | 89720760 | 9E+07 | 89720785 | 89720900 | 1 | Pool1 |
| CHP2_PTEN_8 | GCAGTATAGAGCGTGCAGATAATGA | CATCACATACATACAAGTCAACAACCC | GENOME_REGION | COSM4896 | hg19 | chr10 | 89720760 | 9E+07 | 89720785 | 89720900 | 1 | Pool1 |
| CHP2_PTEN_8 | GCAGTATAGAGCGTGCAGATAATGA | CATCACATACATACAAGTCAACAACCC | GENOME_REGION | COSM4898 | hg19 | chr10 | 89720760 | 9E+07 | 89720785 | 89720900 | 1 | Pool1 |
| CHP2_PTEN_8 | GCAGTATAGAGCGTGCAGATAATGA | CATCACATACATACAAGTCAACAACCC | GENOME_REGION | COSM4899 | hg19 | chr10 | 89720760 | 9E+07 | 89720785 | 89720900 | 1 | Pool1 |
| CHP2_PTEN_8 | GCAGTATAGAGCGTGCAGATAATGA | CATCACATACATACAAGTCAACAACCC | GENOME_REGION | COSM4903 | hg19 | chr10 | 89720760 | 9E+07 | 89720785 | 89720900 | 1 | Pool1 |
| CHP2_PTEN_8 | GCAGTATAGAGCGTGCAGATAATGA | CATCACATACATACAAGTCAACAACCC | GENOME_REGION | COSM4916 | hg19 | chr10 | 89720760 | 9E+07 | 89720785 | 89720900 | 1 | Pool1 |
| CHP2_PTEN_8 | GCAGTATAGAGCGTGCAGATAATGA | CATCACATACATACAAGTCAACAACCC | GENOME_REGION | COSM4932 | hg19 | chr10 | 89720760 | 9E+07 | 89720785 | 89720900 | 1 | Pool1 |
| CHP2_PTEN_8 | GCAGTATAGAGCGTGCAGATAATGA | CATCACATACATACAAGTCAACAACCC | GENOME_REGION | COSM4943 | hg19 | chr10 | 89720760 | 9E+07 | 89720785 | 89720900 | 1 | Pool1 |
| CHP2_PTEN_8 | GCAGTATAGAGCGTGCAGATAATGA | CATCACATACATACAAGTCAACAACCC | GENOME_REGION | COSM4958 | hg19 | chr10 | 89720760 | 9E+07 | 89720785 | 89720900 | 1 | Pool1 |
| CHP2_PTEN_8 | GCAGTATAGAGCGTGCAGATAATGA | CATCACATACATACAAGTCAACAACCC | GENOME_REGION | COSM4982 | hg19 | chr10 | 89720760 | 9E+07 | 89720785 | 89720900 | 1 | Pool1 |
| CHP2_PTEN_8 | GCAGTATAGAGCGTGCAGATAATGA | CATCACATACATACAAGTCAACAACCC | GENOME_REGION | COSM4990 | hg19 | chr10 | 89720760 | 9E+07 | 89720785 | 89720900 | 1 | Pool1 |
| CHP2_PTEN_8 | GCAGTATAGAGCGTGCAGATAATGA | CATCACATACATACAAGTCAACAACCC | GENOME_REGION | COSM4994 | hg19 | chr10 | 89720760 | 9E+07 | 89720785 | 89720900 | 1 | Pool1 |
| CHP2_PTEN_8 | GCAGTATAGAGCGTGCAGATAATGA | CATCACATACATACAAGTCAACAACCC | GENOME_REGION | COSM5008 | hg19 | chr10 | 89720760 | 9E+07 | 89720785 | 89720900 | 1 | Pool1 |
| CHP2_PTEN_8 | GCAGTATAGAGCGTGCAGATAATGA | CATCACATACATACAAGTCAACAACCC | GENOME_REGION | COSM5093 | hg19 | chr10 | 89720760 | 9E+07 | 89720785 | 89720900 | 1 | Pool1 |
| CHP2_PTEN_8 | GCAGTATAGAGCGTGCAGATAATGA | CATCACATACATACAAGTCAACAACCC | GENOME_REGION | COSM5151 | hg19 | chr10 | 89720760 | 9E+07 | 89720785 | 89720900 | 1 | Pool1 |
| CHP2_PTEN_8 | GCAGTATAGAGCGTGCAGATAATGA | CATCACATACATACAAGTCAACAACCC | GENOME_REGION | COSM5255 | hg19 | chr10 | 89720760 | 9E+07 | 89720785 | 89720900 | 1 | Pool1 |
| CHP2_PTEN_8 | GCAGTATAGAGCGTGCAGATAATGA | CATCACATACATACAAGTCAACAACCC | GENOME_REGION | COSM5290 | hg19 | chr10 | 89720760 | 9E+07 | 89720785 | 89720900 | 1 | Pool1 |
| CHP2_PTEN_8 | GCAGTATAGAGCGTGCAGATAATGA | CATCACATACATACAAGTCAACAACCC | GENOME_REGION | COSM5775 | hg19 | chr10 | 89720760 | 9E+07 | 89720785 | 89720900 | 1 | Pool1 |
| CHP2_PTEN_8 | GCAGTATAGAGCGTGCAGATAATGA | CATCACATACATACAAGTCAACAACCC | GENOME_REGION | COSM5801 | hg19 | chr10 | 89720760 | 9E+07 | 89720785 | 89720900 | 1 | Pool1 |
| CHP2_PTEN_8 | GCAGTATAGAGCGTGCAGATAATGA | CATCACATACATACAAGTCAACAACCC | GENOME_REGION | COSM5814 | hg19 | chr10 | 89720760 | 9E+07 | 89720785 | 89720900 | 1 | Pool1 |
| CHP2_PTEN_8 | GCAGTATAGAGCGTGCAGATAATGA | CATCACATACATACAAGTCAACAACCC | GENOME_REGION | COSM5823 | hg19 | chr10 | 89720760 | 9E+07 | 89720785 | 89720900 | 1 | Pool1 |
| CHP2_PTEN_8 | GCAGTATAGAGCGTGCAGATAATGA | CATCACATACATACAAGTCAACAACCC | GENOME_REGION | COSM5869 | hg19 | chr10 | 89720760 | 9E+07 | 89720785 | 89720900 | 1 | Pool1 |
| CHP2_PTEN_8 | GCAGTATAGAGCGTGCAGATAATGA | CATCACATACATACAAGTCAACAACCC | GENOME_REGION | COSM5957 | hg19 | chr10 | 89720760 | 9E+07 | 89720785 | 89720900 | 1 | Pool1 |
| CHP2_SMAD4_1 | CTCATGTGATCTATGCCCGTCT | AGTCTACTTACCAATTCCAGGTGATACA | GENOME_REGION | COSM14215 | hg19 | chr18 | 48575078 | 4,9E+07 | 48575100 | 48575213 | 1 | Pool1 |
| CHP2_SMAD4_1 | CTCATGTGATCTATGCCCGTCT | AGTCTACTTACCAATTCCAGGTGATACA | GENOME_REGION | COSM14216 | hg19 | chr18 | 48575078 | 4,9E+07 | 48575100 | 48575213 | 1 | Pool1 |
| CHP2_SMAD4_1 | CTCATGTGATCTATGCCCGTCT | AGTCTACTTACCAATTCCAGGTGATACA | GENOME_REGION | COSM25274 | hg19 | chr18 | 48575078 | 4,9E+07 | 48575100 | 48575213 | 1 | Pool1 |
| CHP2_SMAD4_3 | ATGGTGAAGGATGAATATGTGCATGA | GCTGGTAGCATTAGACTCAGATGG | GENOME_REGION | COSM14118 | hg19 | chr18 | 48581165 | 4,9E+07 | 48581191 | 48581302 | 1 | Pool1 |
| CHP2_SMAD4_4 | GTGAAGGACTGTTGCAGATAGCAT | AAGGCCCACATGGGTTAATTTG | GENOME_REGION | COSM14057 | hg19 | chr18 | 48584528 | 4,9E+07 | 48584552 | 48584678 | 1 | Pool1 |
| CHP2_SMAD4_4 | GTGAAGGACTGTTGCAGATAGCAT | AAGGCCCACATGGGTTAATTTG | GENOME_REGION | COSM14217 | hg19 | chr18 | 48584528 | 4,9E+07 | 48584552 | 48584678 | 1 | Pool1 |
| CHP2_SMAD4_5 | TTTCTTTAGGGCCTGTTCACAATGA | CTGAGAAGTGACCCCATAATTCCATT | GENOME_REGION | COSM14163 | hg19 | chr18 | 48586227 | 4,9E+07 | 48586252 | 48586361 | 1 | Pool1 |
| CHP2_SMAD4_5 | TTTCTTTAGGGCCTGTTCACAATGA | CTGAGAAGTGACCCCATAATTCCATT | GENOME_REGION | COSM14167 | hg19 | chr18 | 48586227 | 4,9E+07 | 48586252 | 48586361 | 1 | Pool1 |
| CHP2_SMAD4_6 | GCTCCTGAGTATTGGTGTTCCAT | CCTGTGGACATTGGAGAGTTGA | GENOME_REGION | COSM14110 | hg19 | chr18 | 48591792 | 4,9E+07 | 48591815 | 48591931 | 1 | Pool1 |
| CHP2_SMAD4_6 | GCTCCTGAGTATTGGTGTTCCAT | CCTGTGGACATTGGAGAGTTGA | GENOME_REGION | COSM14111 | hg19 | chr18 | 48591792 | 4,9E+07 | 48591815 | 48591931 | 1 | Pool1 |
| CHP2_SMAD4_6 | GCTCCTGAGTATTGGTGTTCCAT | CCTGTGGACATTGGAGAGTTGA | GENOME_REGION | COSM14121 | hg19 | chr18 | 48591792 | 4,9E+07 | 48591815 | 48591931 | 1 | Pool1 |
| CHP2_SMAD4_6 | GCTCCTGAGTATTGGTGTTCCAT | CCTGTGGACATTGGAGAGTTGA | GENOME_REGION | COSM14122 | hg19 | chr18 | 48591792 | 4,9E+07 | 48591815 | 48591931 | 1 | Pool1 |
| CHP2_SMAD4_6 | GCTCCTGAGTATTGGTGTTCCAT | CCTGTGGACATTGGAGAGTTGA | GENOME_REGION | COSM14135 | hg19 | chr18 | 48591792 | 4,9E+07 | 48591815 | 48591931 | 1 | Pool1 |
| CHP2_SMAD4_6 | GCTCCTGAGTATTGGTGTTCCAT | CCTGTGGACATTGGAGAGTTGA | GENOME_REGION | COSM14140 | hg19 | chr18 | 48591792 | 4,9E+07 | 48591815 | 48591931 | 1 | Pool1 |
| CHP2_SMAD4_6 | GCTCCTGAGTATTGGTGTTCCAT | CCTGTGGACATTGGAGAGTTGA | GENOME_REGION | COSM14174 | hg19 | chr18 | 48591792 | 4,9E+07 | 48591815 | 48591931 | 1 | Pool1 |
| CHP2_SMAD4_6 | GCTCCTGAGTATTGGTGTTCCAT | CCTGTGGACATTGGAGAGTTGA | GENOME_REGION | COSM14220 | hg19 | chr18 | 48591792 | 4,9E+07 | 48591815 | 48591931 | 1 | Pool1 |
| CHP2_SMAD4_6 | GCTCCTGAGTATTGGTGTTCCAT | CCTGTGGACATTGGAGAGTTGA | GENOME_REGION | COSM14232 | hg19 | chr18 | 48591792 | 4,9E+07 | 48591815 | 48591931 | 1 | Pool1 |
| CHP2_SMAD4_7 | TGTAATTTCTTTTTTCTTCCTAAGGTTGCACATAG | ACTTGGGTAGATCTTATGAACAGCAT | GENOME_REGION | COSM14175 | hg19 | chr18 | 48593365 | 4,9E+07 | 48593400 | 48593519 | 1 | Pool1 |
| CHP2_SMAD4_7 | TGTAATTTCTTTTTTCTTCCTAAGGTTGCACATAG | ACTTGGGTAGATCTTATGAACAGCAT | GENOME_REGION | COSM14223 | hg19 | chr18 | 48593365 | 4,9E+07 | 48593400 | 48593519 | 1 | Pool1 |
| CHP2_SMAD4_8 | AGGTCTTTGATTTGCGTCAGTGT | GCTGGAGCTATTCCACCTACTG | GENOME_REGION | COSM14105 | hg19 | chr18 | 48603006 | 4,9E+07 | 48603029 | 48603119 | 1 | Pool1 |
| CHP2_SMAD4_8 | AGGTCTTTGATTTGCGTCAGTGT | GCTGGAGCTATTCCACCTACTG | GENOME_REGION | COSM14124 | hg19 | chr18 | 48603006 | 4,9E+07 | 48603029 | 48603119 | 1 | Pool1 |
| CHP2_SMAD4_9 | GCTGCTGGAATTGGTGTTGATG | AGTACTTCGTCTAGGAGCTGGAG | GENOME_REGION | COSM14113 | hg19 | chr18 | 48604637 | 4,9E+07 | 48604659 | 48604774 | 1 | Pool1 |
| CHP2_SMAD4_9 | GCTGCTGGAATTGGTGTTGATG | AGTACTTCGTCTAGGAGCTGGAG | GENOME_REGION | COSM14115 | hg19 | chr18 | 48604637 | 4,9E+07 | 48604659 | 48604774 | 1 | Pool1 |
| CHP2_SMAD4_9 | GCTGCTGGAATTGGTGTTGATG | AGTACTTCGTCTAGGAGCTGGAG | GENOME_REGION | COSM14126 | hg19 | chr18 | 48604637 | 4,9E+07 | 48604659 | 48604774 | 1 | Pool1 |
| CHP2_SMAD4_9 | GCTGCTGGAATTGGTGTTGATG | AGTACTTCGTCTAGGAGCTGGAG | GENOME_REGION | COSM14129 | hg19 | chr18 | 48604637 | 4,9E+07 | 48604659 | 48604774 | 1 | Pool1 |
| CHP2_SMAD4_9 | GCTGCTGGAATTGGTGTTGATG | AGTACTTCGTCTAGGAGCTGGAG | GENOME_REGION | COSM14134 | hg19 | chr18 | 48604637 | 4,9E+07 | 48604659 | 48604774 | 1 | Pool1 |
| CHP2_SMAD4_9 | GCTGCTGGAATTGGTGTTGATG | AGTACTTCGTCTAGGAGCTGGAG | GENOME_REGION | COSM14177 | hg19 | chr18 | 48604637 | 4,9E+07 | 48604659 | 48604774 | 1 | Pool1 |
| CHP2_SMAD4_9 | GCTGCTGGAATTGGTGTTGATG | AGTACTTCGTCTAGGAGCTGGAG | GENOME_REGION | COSM14221 | hg19 | chr18 | 48604637 | 4,9E+07 | 48604659 | 48604774 | 1 | Pool1 |
| CHP2_STK11_1 | GAGCTGATGTCGGTGGGTAT | CTCCGAGTCCAGCACCTC | GENOME_REGION | COSM12925 | hg19 | chr19 | 1206958 | 1207122 | 1206978 | 1207104 | 1 | Pool1 |
| CHP2_STK11_1 | GAGCTGATGTCGGTGGGTAT | CTCCGAGTCCAGCACCTC | GENOME_REGION | COSM21212 | hg19 | chr19 | 1206958 | 1207122 | 1206978 | 1207104 | 1 | Pool1 |
| CHP2_STK11_1 | GAGCTGATGTCGGTGGGTAT | CTCCGAGTCCAGCACCTC | GENOME_REGION | COSM21378 | hg19 | chr19 | 1206958 | 1207122 | 1206978 | 1207104 | 1 | Pool1 |
| CHP2_STK11_1 | GAGCTGATGTCGGTGGGTAT | CTCCGAGTCCAGCACCTC | GENOME_REGION | COSM27322 | hg19 | chr19 | 1206958 | 1207122 | 1206978 | 1207104 | 1 | Pool1 |
| CHP2_STK11_3 | CCGGTGGCACCCTCAAA | CTGGTCCGGCAGGTGTC | GENOME_REGION | COSM20944 | hg19 | chr19 | 1220464 | 1220620 | 1220481 | 1220603 | 1 | Pool1 |
| CHP2_STK11_3 | CCGGTGGCACCCTCAAA | CTGGTCCGGCAGGTGTC | GENOME_REGION | COSM25229 | hg19 | chr19 | 1220464 | 1220620 | 1220481 | 1220603 | 1 | Pool1 |
| CHP2_STK11_3 | CCGGTGGCACCCTCAAA | CTGGTCCGGCAGGTGTC | GENOME_REGION | COSM25847 | hg19 | chr19 | 1220464 | 1220620 | 1220481 | 1220603 | 1 | Pool1 |
| CHP2_STK11_4 | AACATCACCACGGGTCTGTAC | GATGAGGCTCCCACCTTTCAG | GENOME_REGION | COSM12924 | hg19 | chr19 | 1221216 | 1221353 | 1221237 | 1221332 | 1 | Pool1 |
| CHP2_STK11_4 | AACATCACCACGGGTCTGTAC | GATGAGGCTCCCACCTTTCAG | GENOME_REGION | COSM20857 | hg19 | chr19 | 1221216 | 1221353 | 1221237 | 1221332 | 1 | Pool1 |
| CHP2_STK11_4 | AACATCACCACGGGTCTGTAC | GATGAGGCTCCCACCTTTCAG | GENOME_REGION | COSM20871 | hg19 | chr19 | 1221216 | 1221353 | 1221237 | 1221332 | 1 | Pool1 |
| CHP2_STK11_4 | AACATCACCACGGGTCTGTAC | GATGAGGCTCCCACCTTTCAG | GENOME_REGION | COSM21355 | hg19 | chr19 | 1221216 | 1221353 | 1221237 | 1221332 | 1 | Pool1 |
| CHP2_STK11_4 | AACATCACCACGGGTCTGTAC | GATGAGGCTCCCACCTTTCAG | GENOME_REGION | COSM25851 | hg19 | chr19 | 1221216 | 1221353 | 1221237 | 1221332 | 1 | Pool1 |
| CHP2_STK11_4 | AACATCACCACGGGTCTGTAC | GATGAGGCTCCCACCTTTCAG | GENOME_REGION | COSM29005 | hg19 | chr19 | 1221216 | 1221353 | 1221237 | 1221332 | 1 | Pool1 |
| CHP2_STK11_5 | GAAGAAACATCCTCCGGCTGAA | ACCGTGAAGTCCTGAGTGTAGA | GENOME_REGION | COSM18652 | hg19 | chr19 | 1222993 | 1223166 | 1223015 | 1223144 | 1 | Pool1 |
| CHP2_STK11_5 | GAAGAAACATCCTCCGGCTGAA | ACCGTGAAGTCCTGAGTGTAGA | GENOME_REGION | COSM21360 | hg19 | chr19 | 1222993 | 1223166 | 1223015 | 1223144 | 1 | Pool1 |
| CHP2_TP53_1 | TCCACTCACAGTTTCCATAGGTCT | GTTGGAAGTGTCTCATGCTGGAT | GENOME_REGION | COSM11606 | hg19 | chr17 | 7579830 | 7579983 | 7579854 | 7579960 | 1 | Pool1 |
| CHP2_TP53_2 | GGCTGTCCCAGAATGCAAGAA | GATGAAGCTCCCAGAATGCCA | GENOME_REGION | COSM10716 | hg19 | chr17 | 7579330 | 7579506 | 7579351 | 7579485 | 1 | Pool1 |
| CHP2_TP53_2 | GGCTGTCCCAGAATGCAAGAA | GATGAAGCTCCCAGAATGCCA | GENOME_REGION | COSM10886 | hg19 | chr17 | 7579330 | 7579506 | 7579351 | 7579485 | 1 | Pool1 |
| CHP2_TP53_2 | GGCTGTCCCAGAATGCAAGAA | GATGAAGCTCCCAGAATGCCA | GENOME_REGION | COSM11250 | hg19 | chr17 | 7579330 | 7579506 | 7579351 | 7579485 | 1 | Pool1 |
| CHP2_TP53_2 | GGCTGTCCCAGAATGCAAGAA | GATGAAGCTCCCAGAATGCCA | GENOME_REGION | COSM11448 | hg19 | chr17 | 7579330 | 7579506 | 7579351 | 7579485 | 1 | Pool1 |
| CHP2_TP53_2 | GGCTGTCCCAGAATGCAAGAA | GATGAAGCTCCCAGAATGCCA | GENOME_REGION | COSM12296 | hg19 | chr17 | 7579330 | 7579506 | 7579351 | 7579485 | 1 | Pool1 |
| CHP2_TP53_2 | GGCTGTCCCAGAATGCAAGAA | GATGAAGCTCCCAGAATGCCA | GENOME_REGION | COSM13119 | hg19 | chr17 | 7579330 | 7579506 | 7579351 | 7579485 | 1 | Pool1 |
| CHP2_TP53_2 | GGCTGTCCCAGAATGCAAGAA | GATGAAGCTCCCAGAATGCCA | GENOME_REGION | COSM18610 | hg19 | chr17 | 7579330 | 7579506 | 7579351 | 7579485 | 1 | Pool1 |
| CHP2_TP53_2 | GGCTGTCCCAGAATGCAAGAA | GATGAAGCTCCCAGAATGCCA | GENOME_REGION | COSM220765 | hg19 | chr17 | 7579330 | 7579506 | 7579351 | 7579485 | 1 | Pool1 |
| CHP2_TP53_2 | GGCTGTCCCAGAATGCAAGAA | GATGAAGCTCCCAGAATGCCA | GENOME_REGION | COSM220766 | hg19 | chr17 | 7579330 | 7579506 | 7579351 | 7579485 | 1 | Pool1 |
| CHP2_TP53_2 | GGCTGTCCCAGAATGCAAGAA | GATGAAGCTCCCAGAATGCCA | GENOME_REGION | COSM42813 | hg19 | chr17 | 7579330 | 7579506 | 7579351 | 7579485 | 1 | Pool1 |
| CHP2_TP53_2 | GGCTGTCCCAGAATGCAAGAA | GATGAAGCTCCCAGAATGCCA | GENOME_REGION | COSM43544 | hg19 | chr17 | 7579330 | 7579506 | 7579351 | 7579485 | 1 | Pool1 |
| CHP2_TP53_2 | GGCTGTCCCAGAATGCAAGAA | GATGAAGCTCCCAGAATGCCA | GENOME_REGION | COSM43678 | hg19 | chr17 | 7579330 | 7579506 | 7579351 | 7579485 | 1 | Pool1 |
| CHP2_TP53_2 | GGCTGTCCCAGAATGCAAGAA | GATGAAGCTCCCAGAATGCCA | GENOME_REGION | COSM43682 | hg19 | chr17 | 7579330 | 7579506 | 7579351 | 7579485 | 1 | Pool1 |
| CHP2_TP53_2 | GGCTGTCCCAGAATGCAAGAA | GATGAAGCTCCCAGAATGCCA | GENOME_REGION | COSM43688 | hg19 | chr17 | 7579330 | 7579506 | 7579351 | 7579485 | 1 | Pool1 |
| CHP2_TP53_2 | GGCTGTCCCAGAATGCAAGAA | GATGAAGCTCCCAGAATGCCA | GENOME_REGION | COSM43787 | hg19 | chr17 | 7579330 | 7579506 | 7579351 | 7579485 | 1 | Pool1 |
| CHP2_TP53_2 | GGCTGTCCCAGAATGCAAGAA | GATGAAGCTCCCAGAATGCCA | GENOME_REGION | COSM43910 | hg19 | chr17 | 7579330 | 7579506 | 7579351 | 7579485 | 1 | Pool1 |
| CHP2_TP53_2 | GGCTGTCCCAGAATGCAAGAA | GATGAAGCTCCCAGAATGCCA | GENOME_REGION | COSM44018 | hg19 | chr17 | 7579330 | 7579506 | 7579351 | 7579485 | 1 | Pool1 |
| CHP2_TP53_2 | GGCTGTCCCAGAATGCAAGAA | GATGAAGCTCCCAGAATGCCA | GENOME_REGION | COSM44019 | hg19 | chr17 | 7579330 | 7579506 | 7579351 | 7579485 | 1 | Pool1 |
| CHP2_TP53_2 | GGCTGTCCCAGAATGCAAGAA | GATGAAGCTCCCAGAATGCCA | GENOME_REGION | COSM44032 | hg19 | chr17 | 7579330 | 7579506 | 7579351 | 7579485 | 1 | Pool1 |
| CHP2_TP53_2 | GGCTGTCCCAGAATGCAAGAA | GATGAAGCTCCCAGAATGCCA | GENOME_REGION | COSM44036 | hg19 | chr17 | 7579330 | 7579506 | 7579351 | 7579485 | 1 | Pool1 |
| CHP2_TP53_2 | GGCTGTCCCAGAATGCAAGAA | GATGAAGCTCCCAGAATGCCA | GENOME_REGION | COSM44048 | hg19 | chr17 | 7579330 | 7579506 | 7579351 | 7579485 | 1 | Pool1 |
| CHP2_TP53_2 | GGCTGTCCCAGAATGCAAGAA | GATGAAGCTCCCAGAATGCCA | GENOME_REGION | COSM44075 | hg19 | chr17 | 7579330 | 7579506 | 7579351 | 7579485 | 1 | Pool1 |
| CHP2_TP53_2 | GGCTGTCCCAGAATGCAAGAA | GATGAAGCTCCCAGAATGCCA | GENOME_REGION | COSM44192 | hg19 | chr17 | 7579330 | 7579506 | 7579351 | 7579485 | 1 | Pool1 |
| CHP2_TP53_2 | GGCTGTCCCAGAATGCAAGAA | GATGAAGCTCCCAGAATGCCA | GENOME_REGION | COSM44194 | hg19 | chr17 | 7579330 | 7579506 | 7579351 | 7579485 | 1 | Pool1 |
| CHP2_TP53_2 | GGCTGTCCCAGAATGCAAGAA | GATGAAGCTCCCAGAATGCCA | GENOME_REGION | COSM44200 | hg19 | chr17 | 7579330 | 7579506 | 7579351 | 7579485 | 1 | Pool1 |
| CHP2_TP53_2 | GGCTGTCCCAGAATGCAAGAA | GATGAAGCTCCCAGAATGCCA | GENOME_REGION | COSM44231 | hg19 | chr17 | 7579330 | 7579506 | 7579351 | 7579485 | 1 | Pool1 |
| CHP2_TP53_2 | GGCTGTCCCAGAATGCAAGAA | GATGAAGCTCCCAGAATGCCA | GENOME_REGION | COSM44257 | hg19 | chr17 | 7579330 | 7579506 | 7579351 | 7579485 | 1 | Pool1 |
| CHP2_TP53_2 | GGCTGTCCCAGAATGCAAGAA | GATGAAGCTCCCAGAATGCCA | GENOME_REGION | COSM44287 | hg19 | chr17 | 7579330 | 7579506 | 7579351 | 7579485 | 1 | Pool1 |
| CHP2_TP53_2 | GGCTGTCCCAGAATGCAAGAA | GATGAAGCTCCCAGAATGCCA | GENOME_REGION | COSM44447 | hg19 | chr17 | 7579330 | 7579506 | 7579351 | 7579485 | 1 | Pool1 |
| CHP2_TP53_2 | GGCTGTCCCAGAATGCAAGAA | GATGAAGCTCCCAGAATGCCA | GENOME_REGION | COSM44453 | hg19 | chr17 | 7579330 | 7579506 | 7579351 | 7579485 | 1 | Pool1 |
| CHP2_TP53_2 | GGCTGTCCCAGAATGCAAGAA | GATGAAGCTCCCAGAATGCCA | GENOME_REGION | COSM44481 | hg19 | chr17 | 7579330 | 7579506 | 7579351 | 7579485 | 1 | Pool1 |
| CHP2_TP53_2 | GGCTGTCCCAGAATGCAAGAA | GATGAAGCTCCCAGAATGCCA | GENOME_REGION | COSM44492 | hg19 | chr17 | 7579330 | 7579506 | 7579351 | 7579485 | 1 | Pool1 |
| CHP2_TP53_2 | GGCTGTCCCAGAATGCAAGAA | GATGAAGCTCCCAGAATGCCA | GENOME_REGION | COSM44673 | hg19 | chr17 | 7579330 | 7579506 | 7579351 | 7579485 | 1 | Pool1 |
| CHP2_TP53_2 | GGCTGTCCCAGAATGCAAGAA | GATGAAGCTCCCAGAATGCCA | GENOME_REGION | COSM44681 | hg19 | chr17 | 7579330 | 7579506 | 7579351 | 7579485 | 1 | Pool1 |
| CHP2_TP53_2 | GGCTGTCCCAGAATGCAAGAA | GATGAAGCTCCCAGAATGCCA | GENOME_REGION | COSM44986 | hg19 | chr17 | 7579330 | 7579506 | 7579351 | 7579485 | 1 | Pool1 |
| CHP2_TP53_2 | GGCTGTCCCAGAATGCAAGAA | GATGAAGCTCCCAGAATGCCA | GENOME_REGION | COSM45040 | hg19 | chr17 | 7579330 | 7579506 | 7579351 | 7579485 | 1 | Pool1 |
| CHP2_TP53_2 | GGCTGTCCCAGAATGCAAGAA | GATGAAGCTCCCAGAATGCCA | GENOME_REGION | COSM45169 | hg19 | chr17 | 7579330 | 7579506 | 7579351 | 7579485 | 1 | Pool1 |
| CHP2_TP53_2 | GGCTGTCCCAGAATGCAAGAA | GATGAAGCTCCCAGAATGCCA | GENOME_REGION | COSM45179 | hg19 | chr17 | 7579330 | 7579506 | 7579351 | 7579485 | 1 | Pool1 |
| CHP2_TP53_2 | GGCTGTCCCAGAATGCAAGAA | GATGAAGCTCCCAGAATGCCA | GENOME_REGION | COSM45200 | hg19 | chr17 | 7579330 | 7579506 | 7579351 | 7579485 | 1 | Pool1 |
| CHP2_TP53_2 | GGCTGTCCCAGAATGCAAGAA | GATGAAGCTCCCAGAATGCCA | GENOME_REGION | COSM45288 | hg19 | chr17 | 7579330 | 7579506 | 7579351 | 7579485 | 1 | Pool1 |
| CHP2_TP53_2 | GGCTGTCCCAGAATGCAAGAA | GATGAAGCTCCCAGAATGCCA | GENOME_REGION | COSM45500 | hg19 | chr17 | 7579330 | 7579506 | 7579351 | 7579485 | 1 | Pool1 |
| CHP2_TP53_2 | GGCTGTCCCAGAATGCAAGAA | GATGAAGCTCCCAGAATGCCA | GENOME_REGION | COSM45509 | hg19 | chr17 | 7579330 | 7579506 | 7579351 | 7579485 | 1 | Pool1 |
| CHP2_TP53_2 | GGCTGTCCCAGAATGCAAGAA | GATGAAGCTCCCAGAATGCCA | GENOME_REGION | COSM45801 | hg19 | chr17 | 7579330 | 7579506 | 7579351 | 7579485 | 1 | Pool1 |
| CHP2_TP53_2 | GGCTGTCCCAGAATGCAAGAA | GATGAAGCTCCCAGAATGCCA | GENOME_REGION | COSM45918 | hg19 | chr17 | 7579330 | 7579506 | 7579351 | 7579485 | 1 | Pool1 |
| CHP2_TP53_2 | GGCTGTCCCAGAATGCAAGAA | GATGAAGCTCCCAGAATGCCA | GENOME_REGION | COSM45944 | hg19 | chr17 | 7579330 | 7579506 | 7579351 | 7579485 | 1 | Pool1 |
| CHP2_TP53_2 | GGCTGTCCCAGAATGCAAGAA | GATGAAGCTCCCAGAATGCCA | GENOME_REGION | COSM45985 | hg19 | chr17 | 7579330 | 7579506 | 7579351 | 7579485 | 1 | Pool1 |
| CHP2_TP53_2 | GGCTGTCCCAGAATGCAAGAA | GATGAAGCTCCCAGAATGCCA | GENOME_REGION | COSM46103 | hg19 | chr17 | 7579330 | 7579506 | 7579351 | 7579485 | 1 | Pool1 |
| CHP2_TP53_2 | GGCTGTCCCAGAATGCAAGAA | GATGAAGCTCCCAGAATGCCA | GENOME_REGION | COSM46115 | hg19 | chr17 | 7579330 | 7579506 | 7579351 | 7579485 | 1 | Pool1 |
| CHP2_TP53_2 | GGCTGTCCCAGAATGCAAGAA | GATGAAGCTCCCAGAATGCCA | GENOME_REGION | COSM46265 | hg19 | chr17 | 7579330 | 7579506 | 7579351 | 7579485 | 1 | Pool1 |
| CHP2_TP53_2 | GGCTGTCCCAGAATGCAAGAA | GATGAAGCTCCCAGAATGCCA | GENOME_REGION | COSM85574 | hg19 | chr17 | 7579330 | 7579506 | 7579351 | 7579485 | 1 | Pool1 |
| CHP2_TP53_2 | GGCTGTCCCAGAATGCAAGAA | GATGAAGCTCCCAGAATGCCA | GENOME_REGION | COSM99928 | hg19 | chr17 | 7579330 | 7579506 | 7579351 | 7579485 | 1 | Pool1 |
| CHP2_TP53_2 | GGCTGTCCCAGAATGCAAGAA | GATGAAGCTCCCAGAATGCCA | GENOME_REGION | COSM99929 | hg19 | chr17 | 7579330 | 7579506 | 7579351 | 7579485 | 1 | Pool1 |
| CHP2_TP53_3 | TGCACAGGGCAGGTCTTG | CCGTCTTCCAGTTGCTTTATCTGT | GENOME_REGION | COSM10647 | hg19 | chr17 | 7578499 | 7578625 | 7578517 | 7578601 | 1 | Pool1 |
| CHP2_TP53_3 | TGCACAGGGCAGGTCTTG | CCGTCTTCCAGTTGCTTTATCTGT | GENOME_REGION | COSM10684 | hg19 | chr17 | 7578499 | 7578625 | 7578517 | 7578601 | 1 | Pool1 |
| CHP2_TP53_3 | TGCACAGGGCAGGTCTTG | CCGTCTTCCAGTTGCTTTATCTGT | GENOME_REGION | COSM10801 | hg19 | chr17 | 7578499 | 7578625 | 7578517 | 7578601 | 1 | Pool1 |
| CHP2_TP53_3 | TGCACAGGGCAGGTCTTG | CCGTCTTCCAGTTGCTTTATCTGT | GENOME_REGION | COSM10813 | hg19 | chr17 | 7578499 | 7578625 | 7578517 | 7578601 | 1 | Pool1 |
| CHP2_TP53_3 | TGCACAGGGCAGGTCTTG | CCGTCTTCCAGTTGCTTTATCTGT | GENOME_REGION | COSM10862 | hg19 | chr17 | 7578499 | 7578625 | 7578517 | 7578601 | 1 | Pool1 |
| CHP2_TP53_3 | TGCACAGGGCAGGTCTTG | CCGTCTTCCAGTTGCTTTATCTGT | GENOME_REGION | COSM10888 | hg19 | chr17 | 7578499 | 7578625 | 7578517 | 7578601 | 1 | Pool1 |
| CHP2_TP53_3 | TGCACAGGGCAGGTCTTG | CCGTCTTCCAGTTGCTTTATCTGT | GENOME_REGION | COSM10991 | hg19 | chr17 | 7578499 | 7578625 | 7578517 | 7578601 | 1 | Pool1 |
| CHP2_TP53_3 | TGCACAGGGCAGGTCTTG | CCGTCTTCCAGTTGCTTTATCTGT | GENOME_REGION | COSM11166 | hg19 | chr17 | 7578499 | 7578625 | 7578517 | 7578601 | 1 | Pool1 |
| CHP2_TP53_3 | TGCACAGGGCAGGTCTTG | CCGTCTTCCAGTTGCTTTATCTGT | GENOME_REGION | COSM11224 | hg19 | chr17 | 7578499 | 7578625 | 7578517 | 7578601 | 1 | Pool1 |
| CHP2_TP53_3 | TGCACAGGGCAGGTCTTG | CCGTCTTCCAGTTGCTTTATCTGT | GENOME_REGION | COSM11319 | hg19 | chr17 | 7578499 | 7578625 | 7578517 | 7578601 | 1 | Pool1 |
| CHP2_TP53_3 | TGCACAGGGCAGGTCTTG | CCGTCTTCCAGTTGCTTTATCTGT | GENOME_REGION | COSM11449 | hg19 | chr17 | 7578499 | 7578625 | 7578517 | 7578601 | 1 | Pool1 |
| CHP2_TP53_3 | TGCACAGGGCAGGTCTTG | CCGTCTTCCAGTTGCTTTATCTGT | GENOME_REGION | COSM11462 | hg19 | chr17 | 7578499 | 7578625 | 7578517 | 7578601 | 1 | Pool1 |
| CHP2_TP53_3 | TGCACAGGGCAGGTCTTG | CCGTCTTCCAGTTGCTTTATCTGT | GENOME_REGION | COSM11517 | hg19 | chr17 | 7578499 | 7578625 | 7578517 | 7578601 | 1 | Pool1 |
| CHP2_TP53_3 | TGCACAGGGCAGGTCTTG | CCGTCTTCCAGTTGCTTTATCTGT | GENOME_REGION | COSM11582 | hg19 | chr17 | 7578499 | 7578625 | 7578517 | 7578601 | 1 | Pool1 |
| CHP2_TP53_3 | TGCACAGGGCAGGTCTTG | CCGTCTTCCAGTTGCTTTATCTGT | GENOME_REGION | COSM11781 | hg19 | chr17 | 7578499 | 7578625 | 7578517 | 7578601 | 1 | Pool1 |
| CHP2_TP53_3 | TGCACAGGGCAGGTCTTG | CCGTCTTCCAGTTGCTTTATCTGT | GENOME_REGION | COSM21572 | hg19 | chr17 | 7578499 | 7578625 | 7578517 | 7578601 | 1 | Pool1 |
| CHP2_TP53_3 | TGCACAGGGCAGGTCTTG | CCGTCTTCCAGTTGCTTTATCTGT | GENOME_REGION | COSM220782 | hg19 | chr17 | 7578499 | 7578625 | 7578517 | 7578601 | 1 | Pool1 |
| CHP2_TP53_3 | TGCACAGGGCAGGTCTTG | CCGTCTTCCAGTTGCTTTATCTGT | GENOME_REGION | COSM220783 | hg19 | chr17 | 7578499 | 7578625 | 7578517 | 7578601 | 1 | Pool1 |
| CHP2_TP53_3 | TGCACAGGGCAGGTCTTG | CCGTCTTCCAGTTGCTTTATCTGT | GENOME_REGION | COSM220784 | hg19 | chr17 | 7578499 | 7578625 | 7578517 | 7578601 | 1 | Pool1 |
| CHP2_TP53_3 | TGCACAGGGCAGGTCTTG | CCGTCTTCCAGTTGCTTTATCTGT | GENOME_REGION | COSM22908 | hg19 | chr17 | 7578499 | 7578625 | 7578517 | 7578601 | 1 | Pool1 |
| CHP2_TP53_3 | TGCACAGGGCAGGTCTTG | CCGTCTTCCAGTTGCTTTATCTGT | GENOME_REGION | COSM249845 | hg19 | chr17 | 7578499 | 7578625 | 7578517 | 7578601 | 1 | Pool1 |
| CHP2_TP53_3 | TGCACAGGGCAGGTCTTG | CCGTCTTCCAGTTGCTTTATCTGT | GENOME_REGION | COSM40942 | hg19 | chr17 | 7578499 | 7578625 | 7578517 | 7578601 | 1 | Pool1 |
| CHP2_TP53_3 | TGCACAGGGCAGGTCTTG | CCGTCTTCCAGTTGCTTTATCTGT | GENOME_REGION | COSM43533 | hg19 | chr17 | 7578499 | 7578625 | 7578517 | 7578601 | 1 | Pool1 |
| CHP2_TP53_3 | TGCACAGGGCAGGTCTTG | CCGTCTTCCAGTTGCTTTATCTGT | GENOME_REGION | COSM43535 | hg19 | chr17 | 7578499 | 7578625 | 7578517 | 7578601 | 1 | Pool1 |
| CHP2_TP53_3 | TGCACAGGGCAGGTCTTG | CCGTCTTCCAGTTGCTTTATCTGT | GENOME_REGION | COSM43592 | hg19 | chr17 | 7578499 | 7578625 | 7578517 | 7578601 | 1 | Pool1 |
| CHP2_TP53_3 | TGCACAGGGCAGGTCTTG | CCGTCTTCCAGTTGCTTTATCTGT | GENOME_REGION | COSM43661 | hg19 | chr17 | 7578499 | 7578625 | 7578517 | 7578601 | 1 | Pool1 |
| CHP2_TP53_3 | TGCACAGGGCAGGTCTTG | CCGTCTTCCAGTTGCTTTATCTGT | GENOME_REGION | COSM43704 | hg19 | chr17 | 7578499 | 7578625 | 7578517 | 7578601 | 1 | Pool1 |
| CHP2_TP53_3 | TGCACAGGGCAGGTCTTG | CCGTCTTCCAGTTGCTTTATCTGT | GENOME_REGION | COSM43723 | hg19 | chr17 | 7578499 | 7578625 | 7578517 | 7578601 | 1 | Pool1 |
| CHP2_TP53_3 | TGCACAGGGCAGGTCTTG | CCGTCTTCCAGTTGCTTTATCTGT | GENOME_REGION | COSM43730 | hg19 | chr17 | 7578499 | 7578625 | 7578517 | 7578601 | 1 | Pool1 |
| CHP2_TP53_3 | TGCACAGGGCAGGTCTTG | CCGTCTTCCAGTTGCTTTATCTGT | GENOME_REGION | COSM43767 | hg19 | chr17 | 7578499 | 7578625 | 7578517 | 7578601 | 1 | Pool1 |
| CHP2_TP53_3 | TGCACAGGGCAGGTCTTG | CCGTCTTCCAGTTGCTTTATCTGT | GENOME_REGION | COSM43900 | hg19 | chr17 | 7578499 | 7578625 | 7578517 | 7578601 | 1 | Pool1 |
| CHP2_TP53_3 | TGCACAGGGCAGGTCTTG | CCGTCTTCCAGTTGCTTTATCTGT | GENOME_REGION | COSM43912 | hg19 | chr17 | 7578499 | 7578625 | 7578517 | 7578601 | 1 | Pool1 |
| CHP2_TP53_3 | TGCACAGGGCAGGTCTTG | CCGTCTTCCAGTTGCTTTATCTGT | GENOME_REGION | COSM43941 | hg19 | chr17 | 7578499 | 7578625 | 7578517 | 7578601 | 1 | Pool1 |
| CHP2_TP53_3 | TGCACAGGGCAGGTCTTG | CCGTCTTCCAGTTGCTTTATCTGT | GENOME_REGION | COSM43949 | hg19 | chr17 | 7578499 | 7578625 | 7578517 | 7578601 | 1 | Pool1 |
| CHP2_TP53_3 | TGCACAGGGCAGGTCTTG | CCGTCTTCCAGTTGCTTTATCTGT | GENOME_REGION | COSM43963 | hg19 | chr17 | 7578499 | 7578625 | 7578517 | 7578601 | 1 | Pool1 |
| CHP2_TP53_3 | TGCACAGGGCAGGTCTTG | CCGTCTTCCAGTTGCTTTATCTGT | GENOME_REGION | COSM43970 | hg19 | chr17 | 7578499 | 7578625 | 7578517 | 7578601 | 1 | Pool1 |
| CHP2_TP53_3 | TGCACAGGGCAGGTCTTG | CCGTCTTCCAGTTGCTTTATCTGT | GENOME_REGION | COSM44063 | hg19 | chr17 | 7578499 | 7578625 | 7578517 | 7578601 | 1 | Pool1 |
| CHP2_TP53_3 | TGCACAGGGCAGGTCTTG | CCGTCTTCCAGTTGCTTTATCTGT | GENOME_REGION | COSM44142 | hg19 | chr17 | 7578499 | 7578625 | 7578517 | 7578601 | 1 | Pool1 |
| CHP2_TP53_3 | TGCACAGGGCAGGTCTTG | CCGTCTTCCAGTTGCTTTATCTGT | GENOME_REGION | COSM44206 | hg19 | chr17 | 7578499 | 7578625 | 7578517 | 7578601 | 1 | Pool1 |
| CHP2_TP53_3 | TGCACAGGGCAGGTCTTG | CCGTCTTCCAGTTGCTTTATCTGT | GENOME_REGION | COSM44212 | hg19 | chr17 | 7578499 | 7578625 | 7578517 | 7578601 | 1 | Pool1 |
| CHP2_TP53_3 | TGCACAGGGCAGGTCTTG | CCGTCTTCCAGTTGCTTTATCTGT | GENOME_REGION | COSM44219 | hg19 | chr17 | 7578499 | 7578625 | 7578517 | 7578601 | 1 | Pool1 |
| CHP2_TP53_3 | TGCACAGGGCAGGTCTTG | CCGTCTTCCAGTTGCTTTATCTGT | GENOME_REGION | COSM44226 | hg19 | chr17 | 7578499 | 7578625 | 7578517 | 7578601 | 1 | Pool1 |
| CHP2_TP53_3 | TGCACAGGGCAGGTCTTG | CCGTCTTCCAGTTGCTTTATCTGT | GENOME_REGION | COSM44297 | hg19 | chr17 | 7578499 | 7578625 | 7578517 | 7578601 | 1 | Pool1 |
| CHP2_TP53_3 | TGCACAGGGCAGGTCTTG | CCGTCTTCCAGTTGCTTTATCTGT | GENOME_REGION | COSM44319 | hg19 | chr17 | 7578499 | 7578625 | 7578517 | 7578601 | 1 | Pool1 |
| CHP2_TP53_3 | TGCACAGGGCAGGTCTTG | CCGTCTTCCAGTTGCTTTATCTGT | GENOME_REGION | COSM44380 | hg19 | chr17 | 7578499 | 7578625 | 7578517 | 7578601 | 1 | Pool1 |
| CHP2_TP53_3 | TGCACAGGGCAGGTCTTG | CCGTCTTCCAGTTGCTTTATCTGT | GENOME_REGION | COSM44396 | hg19 | chr17 | 7578499 | 7578625 | 7578517 | 7578601 | 1 | Pool1 |
| CHP2_TP53_3 | TGCACAGGGCAGGTCTTG | CCGTCTTCCAGTTGCTTTATCTGT | GENOME_REGION | COSM44397 | hg19 | chr17 | 7578499 | 7578625 | 7578517 | 7578601 | 1 | Pool1 |
| CHP2_TP53_3 | TGCACAGGGCAGGTCTTG | CCGTCTTCCAGTTGCTTTATCTGT | GENOME_REGION | COSM44405 | hg19 | chr17 | 7578499 | 7578625 | 7578517 | 7578601 | 1 | Pool1 |
| CHP2_TP53_3 | TGCACAGGGCAGGTCTTG | CCGTCTTCCAGTTGCTTTATCTGT | GENOME_REGION | COSM44474 | hg19 | chr17 | 7578499 | 7578625 | 7578517 | 7578601 | 1 | Pool1 |
| CHP2_TP53_3 | TGCACAGGGCAGGTCTTG | CCGTCTTCCAGTTGCTTTATCTGT | GENOME_REGION | COSM44506 | hg19 | chr17 | 7578499 | 7578625 | 7578517 | 7578601 | 1 | Pool1 |
| CHP2_TP53_3 | TGCACAGGGCAGGTCTTG | CCGTCTTCCAGTTGCTTTATCTGT | GENOME_REGION | COSM44550 | hg19 | chr17 | 7578499 | 7578625 | 7578517 | 7578601 | 1 | Pool1 |
| CHP2_TP53_3 | TGCACAGGGCAGGTCTTG | CCGTCTTCCAGTTGCTTTATCTGT | GENOME_REGION | COSM44589 | hg19 | chr17 | 7578499 | 7578625 | 7578517 | 7578601 | 1 | Pool1 |
| CHP2_TP53_3 | TGCACAGGGCAGGTCTTG | CCGTCTTCCAGTTGCTTTATCTGT | GENOME_REGION | COSM44641 | hg19 | chr17 | 7578499 | 7578625 | 7578517 | 7578601 | 1 | Pool1 |
| CHP2_TP53_3 | TGCACAGGGCAGGTCTTG | CCGTCTTCCAGTTGCTTTATCTGT | GENOME_REGION | COSM44643 | hg19 | chr17 | 7578499 | 7578625 | 7578517 | 7578601 | 1 | Pool1 |
| CHP2_TP53_3 | TGCACAGGGCAGGTCTTG | CCGTCTTCCAGTTGCTTTATCTGT | GENOME_REGION | COSM44654 | hg19 | chr17 | 7578499 | 7578625 | 7578517 | 7578601 | 1 | Pool1 |
| CHP2_TP53_3 | TGCACAGGGCAGGTCTTG | CCGTCTTCCAGTTGCTTTATCTGT | GENOME_REGION | COSM44670 | hg19 | chr17 | 7578499 | 7578625 | 7578517 | 7578601 | 1 | Pool1 |
| CHP2_TP53_3 | TGCACAGGGCAGGTCTTG | CCGTCTTCCAGTTGCTTTATCTGT | GENOME_REGION | COSM44687 | hg19 | chr17 | 7578499 | 7578625 | 7578517 | 7578601 | 1 | Pool1 |
| CHP2_TP53_3 | TGCACAGGGCAGGTCTTG | CCGTCTTCCAGTTGCTTTATCTGT | GENOME_REGION | COSM44774 | hg19 | chr17 | 7578499 | 7578625 | 7578517 | 7578601 | 1 | Pool1 |
| CHP2_TP53_3 | TGCACAGGGCAGGTCTTG | CCGTCTTCCAGTTGCTTTATCTGT | GENOME_REGION | COSM44794 | hg19 | chr17 | 7578499 | 7578625 | 7578517 | 7578601 | 1 | Pool1 |
| CHP2_TP53_3 | TGCACAGGGCAGGTCTTG | CCGTCTTCCAGTTGCTTTATCTGT | GENOME_REGION | COSM44829 | hg19 | chr17 | 7578499 | 7578625 | 7578517 | 7578601 | 1 | Pool1 |
| CHP2_TP53_3 | TGCACAGGGCAGGTCTTG | CCGTCTTCCAGTTGCTTTATCTGT | GENOME_REGION | COSM44910 | hg19 | chr17 | 7578499 | 7578625 | 7578517 | 7578601 | 1 | Pool1 |
| CHP2_TP53_3 | TGCACAGGGCAGGTCTTG | CCGTCTTCCAGTTGCTTTATCTGT | GENOME_REGION | COSM44933 | hg19 | chr17 | 7578499 | 7578625 | 7578517 | 7578601 | 1 | Pool1 |
| CHP2_TP53_3 | TGCACAGGGCAGGTCTTG | CCGTCTTCCAGTTGCTTTATCTGT | GENOME_REGION | COSM44966 | hg19 | chr17 | 7578499 | 7578625 | 7578517 | 7578601 | 1 | Pool1 |
| CHP2_TP53_3 | TGCACAGGGCAGGTCTTG | CCGTCTTCCAGTTGCTTTATCTGT | GENOME_REGION | COSM45015 | hg19 | chr17 | 7578499 | 7578625 | 7578517 | 7578601 | 1 | Pool1 |
| CHP2_TP53_3 | TGCACAGGGCAGGTCTTG | CCGTCTTCCAGTTGCTTTATCTGT | GENOME_REGION | COSM45077 | hg19 | chr17 | 7578499 | 7578625 | 7578517 | 7578601 | 1 | Pool1 |
| CHP2_TP53_3 | TGCACAGGGCAGGTCTTG | CCGTCTTCCAGTTGCTTTATCTGT | GENOME_REGION | COSM45089 | hg19 | chr17 | 7578499 | 7578625 | 7578517 | 7578601 | 1 | Pool1 |
| CHP2_TP53_3 | TGCACAGGGCAGGTCTTG | CCGTCTTCCAGTTGCTTTATCTGT | GENOME_REGION | COSM45131 | hg19 | chr17 | 7578499 | 7578625 | 7578517 | 7578601 | 1 | Pool1 |
| CHP2_TP53_3 | TGCACAGGGCAGGTCTTG | CCGTCTTCCAGTTGCTTTATCTGT | GENOME_REGION | COSM45293 | hg19 | chr17 | 7578499 | 7578625 | 7578517 | 7578601 | 1 | Pool1 |
| CHP2_TP53_3 | TGCACAGGGCAGGTCTTG | CCGTCTTCCAGTTGCTTTATCTGT | GENOME_REGION | COSM45364 | hg19 | chr17 | 7578499 | 7578625 | 7578517 | 7578601 | 1 | Pool1 |
| CHP2_TP53_3 | TGCACAGGGCAGGTCTTG | CCGTCTTCCAGTTGCTTTATCTGT | GENOME_REGION | COSM45586 | hg19 | chr17 | 7578499 | 7578625 | 7578517 | 7578601 | 1 | Pool1 |
| CHP2_TP53_3 | TGCACAGGGCAGGTCTTG | CCGTCTTCCAGTTGCTTTATCTGT | GENOME_REGION | COSM45672 | hg19 | chr17 | 7578499 | 7578625 | 7578517 | 7578601 | 1 | Pool1 |
| CHP2_TP53_3 | TGCACAGGGCAGGTCTTG | CCGTCTTCCAGTTGCTTTATCTGT | GENOME_REGION | COSM45809 | hg19 | chr17 | 7578499 | 7578625 | 7578517 | 7578601 | 1 | Pool1 |
| CHP2_TP53_3 | TGCACAGGGCAGGTCTTG | CCGTCTTCCAGTTGCTTTATCTGT | GENOME_REGION | COSM45882 | hg19 | chr17 | 7578499 | 7578625 | 7578517 | 7578601 | 1 | Pool1 |
| CHP2_TP53_3 | TGCACAGGGCAGGTCTTG | CCGTCTTCCAGTTGCTTTATCTGT | GENOME_REGION | COSM46049 | hg19 | chr17 | 7578499 | 7578625 | 7578517 | 7578601 | 1 | Pool1 |
| CHP2_TP53_3 | TGCACAGGGCAGGTCTTG | CCGTCTTCCAGTTGCTTTATCTGT | GENOME_REGION | COSM46114 | hg19 | chr17 | 7578499 | 7578625 | 7578517 | 7578601 | 1 | Pool1 |
| CHP2_TP53_3 | TGCACAGGGCAGGTCTTG | CCGTCTTCCAGTTGCTTTATCTGT | GENOME_REGION | COSM46131 | hg19 | chr17 | 7578499 | 7578625 | 7578517 | 7578601 | 1 | Pool1 |
| CHP2_TP53_3 | TGCACAGGGCAGGTCTTG | CCGTCTTCCAGTTGCTTTATCTGT | GENOME_REGION | COSM53285 | hg19 | chr17 | 7578499 | 7578625 | 7578517 | 7578601 | 1 | Pool1 |
| CHP2_TP53_3 | TGCACAGGGCAGGTCTTG | CCGTCTTCCAGTTGCTTTATCTGT | GENOME_REGION | COSM6900 | hg19 | chr17 | 7578499 | 7578625 | 7578517 | 7578601 | 1 | Pool1 |
| CHP2_TP53_3 | TGCACAGGGCAGGTCTTG | CCGTCTTCCAGTTGCTTTATCTGT | GENOME_REGION | COSM99598 | hg19 | chr17 | 7578499 | 7578625 | 7578517 | 7578601 | 1 | Pool1 |
| CHP2_TP53_3 | TGCACAGGGCAGGTCTTG | CCGTCTTCCAGTTGCTTTATCTGT | GENOME_REGION | COSM99599 | hg19 | chr17 | 7578499 | 7578625 | 7578517 | 7578601 | 1 | Pool1 |
| CHP2_TP53_3 | TGCACAGGGCAGGTCTTG | CCGTCTTCCAGTTGCTTTATCTGT | GENOME_REGION | COSM99600 | hg19 | chr17 | 7578499 | 7578625 | 7578517 | 7578601 | 1 | Pool1 |
| CHP2_TP53_3 | TGCACAGGGCAGGTCTTG | CCGTCTTCCAGTTGCTTTATCTGT | GENOME_REGION | COSM99601 | hg19 | chr17 | 7578499 | 7578625 | 7578517 | 7578601 | 1 | Pool1 |
| CHP2_TP53_3 | TGCACAGGGCAGGTCTTG | CCGTCTTCCAGTTGCTTTATCTGT | GENOME_REGION | COSM99944 | hg19 | chr17 | 7578499 | 7578625 | 7578517 | 7578601 | 1 | Pool1 |
| CHP2_TP53_3 | TGCACAGGGCAGGTCTTG | CCGTCTTCCAGTTGCTTTATCTGT | GENOME_REGION | COSM99945 | hg19 | chr17 | 7578499 | 7578625 | 7578517 | 7578601 | 1 | Pool1 |
| CHP2_TP53_3 | TGCACAGGGCAGGTCTTG | CCGTCTTCCAGTTGCTTTATCTGT | GENOME_REGION | COSM99946 | hg19 | chr17 | 7578499 | 7578625 | 7578517 | 7578601 | 1 | Pool1 |
| CHP2_TP53_4 | ACCAGCCCTGTCGTCTCT | GTGCAGCTGTGGGTTGATTC | GENOME_REGION | COSM10645 | hg19 | chr17 | 7578335 | 7578503 | 7578353 | 7578483 | 1 | Pool1 |
| CHP2_TP53_4 | ACCAGCCCTGTCGTCTCT | GTGCAGCTGTGGGTTGATTC | GENOME_REGION | COSM10648 | hg19 | chr17 | 7578335 | 7578503 | 7578353 | 7578483 | 1 | Pool1 |
| CHP2_TP53_4 | ACCAGCCCTGTCGTCTCT | GTGCAGCTGTGGGTTGATTC | GENOME_REGION | COSM10650 | hg19 | chr17 | 7578335 | 7578503 | 7578353 | 7578483 | 1 | Pool1 |
| CHP2_TP53_4 | ACCAGCCCTGTCGTCTCT | GTGCAGCTGTGGGTTGATTC | GENOME_REGION | COSM10651 | hg19 | chr17 | 7578335 | 7578503 | 7578353 | 7578483 | 1 | Pool1 |
| CHP2_TP53_4 | ACCAGCCCTGTCGTCTCT | GTGCAGCTGTGGGTTGATTC | GENOME_REGION | COSM10670 | hg19 | chr17 | 7578335 | 7578503 | 7578353 | 7578483 | 1 | Pool1 |
| CHP2_TP53_4 | ACCAGCCCTGTCGTCTCT | GTGCAGCTGTGGGTTGATTC | GENOME_REGION | COSM10687 | hg19 | chr17 | 7578335 | 7578503 | 7578353 | 7578483 | 1 | Pool1 |
| CHP2_TP53_4 | ACCAGCCCTGTCGTCTCT | GTGCAGCTGTGGGTTGATTC | GENOME_REGION | COSM10690 | hg19 | chr17 | 7578335 | 7578503 | 7578353 | 7578483 | 1 | Pool1 |
| CHP2_TP53_4 | ACCAGCCCTGTCGTCTCT | GTGCAGCTGTGGGTTGATTC | GENOME_REGION | COSM10706 | hg19 | chr17 | 7578335 | 7578503 | 7578353 | 7578483 | 1 | Pool1 |
| CHP2_TP53_4 | ACCAGCCCTGTCGTCTCT | GTGCAGCTGTGGGTTGATTC | GENOME_REGION | COSM10714 | hg19 | chr17 | 7578335 | 7578503 | 7578353 | 7578483 | 1 | Pool1 |
| CHP2_TP53_4 | ACCAGCCCTGTCGTCTCT | GTGCAGCTGTGGGTTGATTC | GENOME_REGION | COSM10718 | hg19 | chr17 | 7578335 | 7578503 | 7578353 | 7578483 | 1 | Pool1 |
| CHP2_TP53_4 | ACCAGCCCTGTCGTCTCT | GTGCAGCTGTGGGTTGATTC | GENOME_REGION | COSM10738 | hg19 | chr17 | 7578335 | 7578503 | 7578353 | 7578483 | 1 | Pool1 |
| CHP2_TP53_4 | ACCAGCCCTGTCGTCTCT | GTGCAGCTGTGGGTTGATTC | GENOME_REGION | COSM10739 | hg19 | chr17 | 7578335 | 7578503 | 7578353 | 7578483 | 1 | Pool1 |
| CHP2_TP53_4 | ACCAGCCCTGTCGTCTCT | GTGCAGCTGTGGGTTGATTC | GENOME_REGION | COSM10750 | hg19 | chr17 | 7578335 | 7578503 | 7578353 | 7578483 | 1 | Pool1 |
| CHP2_TP53_4 | ACCAGCCCTGTCGTCTCT | GTGCAGCTGTGGGTTGATTC | GENOME_REGION | COSM10760 | hg19 | chr17 | 7578335 | 7578503 | 7578353 | 7578483 | 1 | Pool1 |
| CHP2_TP53_4 | ACCAGCCCTGTCGTCTCT | GTGCAGCTGTGGGTTGATTC | GENOME_REGION | COSM10762 | hg19 | chr17 | 7578335 | 7578503 | 7578353 | 7578483 | 1 | Pool1 |
| CHP2_TP53_4 | ACCAGCCCTGTCGTCTCT | GTGCAGCTGTGGGTTGATTC | GENOME_REGION | COSM10768 | hg19 | chr17 | 7578335 | 7578503 | 7578353 | 7578483 | 1 | Pool1 |
| CHP2_TP53_4 | ACCAGCCCTGTCGTCTCT | GTGCAGCTGTGGGTTGATTC | GENOME_REGION | COSM10790 | hg19 | chr17 | 7578335 | 7578503 | 7578353 | 7578483 | 1 | Pool1 |
| CHP2_TP53_4 | ACCAGCCCTGTCGTCTCT | GTGCAGCTGTGGGTTGATTC | GENOME_REGION | COSM10808 | hg19 | chr17 | 7578335 | 7578503 | 7578353 | 7578483 | 1 | Pool1 |
| CHP2_TP53_4 | ACCAGCCCTGTCGTCTCT | GTGCAGCTGTGGGTTGATTC | GENOME_REGION | COSM10870 | hg19 | chr17 | 7578335 | 7578503 | 7578353 | 7578483 | 1 | Pool1 |
| CHP2_TP53_4 | ACCAGCCCTGTCGTCTCT | GTGCAGCTGTGGGTTGATTC | GENOME_REGION | COSM10889 | hg19 | chr17 | 7578335 | 7578503 | 7578353 | 7578483 | 1 | Pool1 |
| CHP2_TP53_4 | ACCAGCCCTGTCGTCTCT | GTGCAGCTGTGGGTTGATTC | GENOME_REGION | COSM10912 | hg19 | chr17 | 7578335 | 7578503 | 7578353 | 7578483 | 1 | Pool1 |
| CHP2_TP53_4 | ACCAGCCCTGTCGTCTCT | GTGCAGCTGTGGGTTGATTC | GENOME_REGION | COSM10996 | hg19 | chr17 | 7578335 | 7578503 | 7578353 | 7578483 | 1 | Pool1 |
| CHP2_TP53_4 | ACCAGCCCTGTCGTCTCT | GTGCAGCTGTGGGTTGATTC | GENOME_REGION | COSM11084 | hg19 | chr17 | 7578335 | 7578503 | 7578353 | 7578483 | 1 | Pool1 |
| CHP2_TP53_4 | ACCAGCCCTGTCGTCTCT | GTGCAGCTGTGGGTTGATTC | GENOME_REGION | COSM11087 | hg19 | chr17 | 7578335 | 7578503 | 7578353 | 7578483 | 1 | Pool1 |
| CHP2_TP53_4 | ACCAGCCCTGTCGTCTCT | GTGCAGCTGTGGGTTGATTC | GENOME_REGION | COSM11090 | hg19 | chr17 | 7578335 | 7578503 | 7578353 | 7578483 | 1 | Pool1 |
| CHP2_TP53_4 | ACCAGCCCTGTCGTCTCT | GTGCAGCTGTGGGTTGATTC | GENOME_REGION | COSM11114 | hg19 | chr17 | 7578335 | 7578503 | 7578353 | 7578483 | 1 | Pool1 |
| CHP2_TP53_4 | ACCAGCCCTGTCGTCTCT | GTGCAGCTGTGGGTTGATTC | GENOME_REGION | COSM11148 | hg19 | chr17 | 7578335 | 7578503 | 7578353 | 7578483 | 1 | Pool1 |
| CHP2_TP53_4 | ACCAGCCCTGTCGTCTCT | GTGCAGCTGTGGGTTGATTC | GENOME_REGION | COSM111495 | hg19 | chr17 | 7578335 | 7578503 | 7578353 | 7578483 | 1 | Pool1 |
| CHP2_TP53_4 | ACCAGCCCTGTCGTCTCT | GTGCAGCTGTGGGTTGATTC | GENOME_REGION | COSM111496 | hg19 | chr17 | 7578335 | 7578503 | 7578353 | 7578483 | 1 | Pool1 |
| CHP2_TP53_4 | ACCAGCCCTGTCGTCTCT | GTGCAGCTGTGGGTTGATTC | GENOME_REGION | COSM111497 | hg19 | chr17 | 7578335 | 7578503 | 7578353 | 7578483 | 1 | Pool1 |
| CHP2_TP53_4 | ACCAGCCCTGTCGTCTCT | GTGCAGCTGTGGGTTGATTC | GENOME_REGION | COSM111498 | hg19 | chr17 | 7578335 | 7578503 | 7578353 | 7578483 | 1 | Pool1 |
| CHP2_TP53_4 | ACCAGCCCTGTCGTCTCT | GTGCAGCTGTGGGTTGATTC | GENOME_REGION | COSM11218 | hg19 | chr17 | 7578335 | 7578503 | 7578353 | 7578483 | 1 | Pool1 |
| CHP2_TP53_4 | ACCAGCCCTGTCGTCTCT | GTGCAGCTGTGGGTTGATTC | GENOME_REGION | COSM11249 | hg19 | chr17 | 7578335 | 7578503 | 7578353 | 7578483 | 1 | Pool1 |
| CHP2_TP53_4 | ACCAGCCCTGTCGTCTCT | GTGCAGCTGTGGGTTGATTC | GENOME_REGION | COSM11323 | hg19 | chr17 | 7578335 | 7578503 | 7578353 | 7578483 | 1 | Pool1 |
| CHP2_TP53_4 | ACCAGCCCTGTCGTCTCT | GTGCAGCTGTGGGTTGATTC | GENOME_REGION | COSM11333 | hg19 | chr17 | 7578335 | 7578503 | 7578353 | 7578483 | 1 | Pool1 |
| CHP2_TP53_4 | ACCAGCCCTGTCGTCTCT | GTGCAGCTGTGGGTTGATTC | GENOME_REGION | COSM11369 | hg19 | chr17 | 7578335 | 7578503 | 7578353 | 7578483 | 1 | Pool1 |
| CHP2_TP53_4 | ACCAGCCCTGTCGTCTCT | GTGCAGCTGTGGGTTGATTC | GENOME_REGION | COSM11496 | hg19 | chr17 | 7578335 | 7578503 | 7578353 | 7578483 | 1 | Pool1 |
| CHP2_TP53_4 | ACCAGCCCTGTCGTCTCT | GTGCAGCTGTGGGTTGATTC | GENOME_REGION | COSM11508 | hg19 | chr17 | 7578335 | 7578503 | 7578353 | 7578483 | 1 | Pool1 |
| CHP2_TP53_4 | ACCAGCCCTGTCGTCTCT | GTGCAGCTGTGGGTTGATTC | GENOME_REGION | COSM11717 | hg19 | chr17 | 7578335 | 7578503 | 7578353 | 7578483 | 1 | Pool1 |
| CHP2_TP53_4 | ACCAGCCCTGTCGTCTCT | GTGCAGCTGTGGGTTGATTC | GENOME_REGION | COSM117395 | hg19 | chr17 | 7578335 | 7578503 | 7578353 | 7578483 | 1 | Pool1 |
| CHP2_TP53_4 | ACCAGCCCTGTCGTCTCT | GTGCAGCTGTGGGTTGATTC | GENOME_REGION | COSM117396 | hg19 | chr17 | 7578335 | 7578503 | 7578353 | 7578483 | 1 | Pool1 |
| CHP2_TP53_4 | ACCAGCCCTGTCGTCTCT | GTGCAGCTGTGGGTTGATTC | GENOME_REGION | COSM117397 | hg19 | chr17 | 7578335 | 7578503 | 7578353 | 7578483 | 1 | Pool1 |
| CHP2_TP53_4 | ACCAGCCCTGTCGTCTCT | GTGCAGCTGTGGGTTGATTC | GENOME_REGION | COSM117398 | hg19 | chr17 | 7578335 | 7578503 | 7578353 | 7578483 | 1 | Pool1 |
| CHP2_TP53_4 | ACCAGCCCTGTCGTCTCT | GTGCAGCTGTGGGTTGATTC | GENOME_REGION | COSM11966 | hg19 | chr17 | 7578335 | 7578503 | 7578353 | 7578483 | 1 | Pool1 |
| CHP2_TP53_4 | ACCAGCCCTGTCGTCTCT | GTGCAGCTGTGGGTTGATTC | GENOME_REGION | COSM11998 | hg19 | chr17 | 7578335 | 7578503 | 7578353 | 7578483 | 1 | Pool1 |
| CHP2_TP53_4 | ACCAGCCCTGTCGTCTCT | GTGCAGCTGTGGGTTGATTC | GENOME_REGION | COSM121042 | hg19 | chr17 | 7578335 | 7578503 | 7578353 | 7578483 | 1 | Pool1 |
| CHP2_TP53_4 | ACCAGCCCTGTCGTCTCT | GTGCAGCTGTGGGTTGATTC | GENOME_REGION | COSM121043 | hg19 | chr17 | 7578335 | 7578503 | 7578353 | 7578483 | 1 | Pool1 |
| CHP2_TP53_4 | ACCAGCCCTGTCGTCTCT | GTGCAGCTGTGGGTTGATTC | GENOME_REGION | COSM121044 | hg19 | chr17 | 7578335 | 7578503 | 7578353 | 7578483 | 1 | Pool1 |
| CHP2_TP53_4 | ACCAGCCCTGTCGTCTCT | GTGCAGCTGTGGGTTGATTC | GENOME_REGION | COSM121045 | hg19 | chr17 | 7578335 | 7578503 | 7578353 | 7578483 | 1 | Pool1 |
| CHP2_TP53_4 | ACCAGCCCTGTCGTCTCT | GTGCAGCTGTGGGTTGATTC | GENOME_REGION | COSM129848 | hg19 | chr17 | 7578335 | 7578503 | 7578353 | 7578483 | 1 | Pool1 |
| CHP2_TP53_4 | ACCAGCCCTGTCGTCTCT | GTGCAGCTGTGGGTTGATTC | GENOME_REGION | COSM129849 | hg19 | chr17 | 7578335 | 7578503 | 7578353 | 7578483 | 1 | Pool1 |
| CHP2_TP53_4 | ACCAGCCCTGTCGTCTCT | GTGCAGCTGTGGGTTGATTC | GENOME_REGION | COSM129850 | hg19 | chr17 | 7578335 | 7578503 | 7578353 | 7578483 | 1 | Pool1 |
| CHP2_TP53_4 | ACCAGCCCTGTCGTCTCT | GTGCAGCTGTGGGTTGATTC | GENOME_REGION | COSM129851 | hg19 | chr17 | 7578335 | 7578503 | 7578353 | 7578483 | 1 | Pool1 |
| CHP2_TP53_4 | ACCAGCCCTGTCGTCTCT | GTGCAGCTGTGGGTTGATTC | GENOME_REGION | COSM129852 | hg19 | chr17 | 7578335 | 7578503 | 7578353 | 7578483 | 1 | Pool1 |
| CHP2_TP53_4 | ACCAGCCCTGTCGTCTCT | GTGCAGCTGTGGGTTGATTC | GENOME_REGION | COSM129853 | hg19 | chr17 | 7578335 | 7578503 | 7578353 | 7578483 | 1 | Pool1 |
| CHP2_TP53_4 | ACCAGCCCTGTCGTCTCT | GTGCAGCTGTGGGTTGATTC | GENOME_REGION | COSM129854 | hg19 | chr17 | 7578335 | 7578503 | 7578353 | 7578483 | 1 | Pool1 |
| CHP2_TP53_4 | ACCAGCCCTGTCGTCTCT | GTGCAGCTGTGGGTTGATTC | GENOME_REGION | COSM129855 | hg19 | chr17 | 7578335 | 7578503 | 7578353 | 7578483 | 1 | Pool1 |
| CHP2_TP53_4 | ACCAGCCCTGTCGTCTCT | GTGCAGCTGTGGGTTGATTC | GENOME_REGION | COSM129856 | hg19 | chr17 | 7578335 | 7578503 | 7578353 | 7578483 | 1 | Pool1 |
| CHP2_TP53_4 | ACCAGCCCTGTCGTCTCT | GTGCAGCTGTGGGTTGATTC | GENOME_REGION | COSM129857 | hg19 | chr17 | 7578335 | 7578503 | 7578353 | 7578483 | 1 | Pool1 |
| CHP2_TP53_4 | ACCAGCCCTGTCGTCTCT | GTGCAGCTGTGGGTTGATTC | GENOME_REGION | COSM129858 | hg19 | chr17 | 7578335 | 7578503 | 7578353 | 7578483 | 1 | Pool1 |
| CHP2_TP53_4 | ACCAGCCCTGTCGTCTCT | GTGCAGCTGTGGGTTGATTC | GENOME_REGION | COSM129859 | hg19 | chr17 | 7578335 | 7578503 | 7578353 | 7578483 | 1 | Pool1 |
| CHP2_TP53_4 | ACCAGCCCTGTCGTCTCT | GTGCAGCTGTGGGTTGATTC | GENOME_REGION | COSM131480 | hg19 | chr17 | 7578335 | 7578503 | 7578353 | 7578483 | 1 | Pool1 |
| CHP2_TP53_4 | ACCAGCCCTGTCGTCTCT | GTGCAGCTGTGGGTTGATTC | GENOME_REGION | COSM131481 | hg19 | chr17 | 7578335 | 7578503 | 7578353 | 7578483 | 1 | Pool1 |
| CHP2_TP53_4 | ACCAGCCCTGTCGTCTCT | GTGCAGCTGTGGGTTGATTC | GENOME_REGION | COSM131482 | hg19 | chr17 | 7578335 | 7578503 | 7578353 | 7578483 | 1 | Pool1 |
| CHP2_TP53_4 | ACCAGCCCTGTCGTCTCT | GTGCAGCTGTGGGTTGATTC | GENOME_REGION | COSM131483 | hg19 | chr17 | 7578335 | 7578503 | 7578353 | 7578483 | 1 | Pool1 |
| CHP2_TP53_4 | ACCAGCCCTGTCGTCTCT | GTGCAGCTGTGGGTTGATTC | GENOME_REGION | COSM131534 | hg19 | chr17 | 7578335 | 7578503 | 7578353 | 7578483 | 1 | Pool1 |
| CHP2_TP53_4 | ACCAGCCCTGTCGTCTCT | GTGCAGCTGTGGGTTGATTC | GENOME_REGION | COSM131535 | hg19 | chr17 | 7578335 | 7578503 | 7578353 | 7578483 | 1 | Pool1 |
| CHP2_TP53_4 | ACCAGCCCTGTCGTCTCT | GTGCAGCTGTGGGTTGATTC | GENOME_REGION | COSM131536 | hg19 | chr17 | 7578335 | 7578503 | 7578353 | 7578483 | 1 | Pool1 |
| CHP2_TP53_4 | ACCAGCCCTGTCGTCTCT | GTGCAGCTGTGGGTTGATTC | GENOME_REGION | COSM131537 | hg19 | chr17 | 7578335 | 7578503 | 7578353 | 7578483 | 1 | Pool1 |
| CHP2_TP53_4 | ACCAGCCCTGTCGTCTCT | GTGCAGCTGTGGGTTGATTC | GENOME_REGION | COSM179822 | hg19 | chr17 | 7578335 | 7578503 | 7578353 | 7578483 | 1 | Pool1 |
| CHP2_TP53_4 | ACCAGCCCTGTCGTCTCT | GTGCAGCTGTGGGTTGATTC | GENOME_REGION | COSM179823 | hg19 | chr17 | 7578335 | 7578503 | 7578353 | 7578483 | 1 | Pool1 |
| CHP2_TP53_4 | ACCAGCCCTGTCGTCTCT | GTGCAGCTGTGGGTTGATTC | GENOME_REGION | COSM179824 | hg19 | chr17 | 7578335 | 7578503 | 7578353 | 7578483 | 1 | Pool1 |
| CHP2_TP53_4 | ACCAGCCCTGTCGTCTCT | GTGCAGCTGTGGGTTGATTC | GENOME_REGION | COSM179825 | hg19 | chr17 | 7578335 | 7578503 | 7578353 | 7578483 | 1 | Pool1 |
| CHP2_TP53_4 | ACCAGCCCTGTCGTCTCT | GTGCAGCTGTGGGTTGATTC | GENOME_REGION | COSM220778 | hg19 | chr17 | 7578335 | 7578503 | 7578353 | 7578483 | 1 | Pool1 |
| CHP2_TP53_4 | ACCAGCCCTGTCGTCTCT | GTGCAGCTGTGGGTTGATTC | GENOME_REGION | COSM220779 | hg19 | chr17 | 7578335 | 7578503 | 7578353 | 7578483 | 1 | Pool1 |
| CHP2_TP53_4 | ACCAGCCCTGTCGTCTCT | GTGCAGCTGTGGGTTGATTC | GENOME_REGION | COSM220780 | hg19 | chr17 | 7578335 | 7578503 | 7578353 | 7578483 | 1 | Pool1 |
| CHP2_TP53_4 | ACCAGCCCTGTCGTCTCT | GTGCAGCTGTGGGTTGATTC | GENOME_REGION | COSM220781 | hg19 | chr17 | 7578335 | 7578503 | 7578353 | 7578483 | 1 | Pool1 |
| CHP2_TP53_4 | ACCAGCCCTGTCGTCTCT | GTGCAGCTGTGGGTTGATTC | GENOME_REGION | COSM43054 | hg19 | chr17 | 7578335 | 7578503 | 7578353 | 7578483 | 1 | Pool1 |
| CHP2_TP53_4 | ACCAGCCCTGTCGTCTCT | GTGCAGCTGTGGGTTGATTC | GENOME_REGION | COSM43541 | hg19 | chr17 | 7578335 | 7578503 | 7578353 | 7578483 | 1 | Pool1 |
| CHP2_TP53_4 | ACCAGCCCTGTCGTCTCT | GTGCAGCTGTGGGTTGATTC | GENOME_REGION | COSM43545 | hg19 | chr17 | 7578335 | 7578503 | 7578353 | 7578483 | 1 | Pool1 |
| CHP2_TP53_4 | ACCAGCCCTGTCGTCTCT | GTGCAGCTGTGGGTTGATTC | GENOME_REGION | COSM43548 | hg19 | chr17 | 7578335 | 7578503 | 7578353 | 7578483 | 1 | Pool1 |
| CHP2_TP53_4 | ACCAGCCCTGTCGTCTCT | GTGCAGCTGTGGGTTGATTC | GENOME_REGION | COSM43559 | hg19 | chr17 | 7578335 | 7578503 | 7578353 | 7578483 | 1 | Pool1 |
| CHP2_TP53_4 | ACCAGCCCTGTCGTCTCT | GTGCAGCTGTGGGTTGATTC | GENOME_REGION | COSM43570 | hg19 | chr17 | 7578335 | 7578503 | 7578353 | 7578483 | 1 | Pool1 |
| CHP2_TP53_4 | ACCAGCCCTGTCGTCTCT | GTGCAGCTGTGGGTTGATTC | GENOME_REGION | COSM43582 | hg19 | chr17 | 7578335 | 7578503 | 7578353 | 7578483 | 1 | Pool1 |
| CHP2_TP53_4 | ACCAGCCCTGTCGTCTCT | GTGCAGCTGTGGGTTGATTC | GENOME_REGION | COSM43584 | hg19 | chr17 | 7578335 | 7578503 | 7578353 | 7578483 | 1 | Pool1 |
| CHP2_TP53_4 | ACCAGCCCTGTCGTCTCT | GTGCAGCTGTGGGTTGATTC | GENOME_REGION | COSM43597 | hg19 | chr17 | 7578335 | 7578503 | 7578353 | 7578483 | 1 | Pool1 |
| CHP2_TP53_4 | ACCAGCCCTGTCGTCTCT | GTGCAGCTGTGGGTTGATTC | GENOME_REGION | COSM43615 | hg19 | chr17 | 7578335 | 7578503 | 7578353 | 7578483 | 1 | Pool1 |
| CHP2_TP53_4 | ACCAGCCCTGTCGTCTCT | GTGCAGCTGTGGGTTGATTC | GENOME_REGION | COSM43625 | hg19 | chr17 | 7578335 | 7578503 | 7578353 | 7578483 | 1 | Pool1 |
| CHP2_TP53_4 | ACCAGCCCTGTCGTCTCT | GTGCAGCTGTGGGTTGATTC | GENOME_REGION | COSM43626 | hg19 | chr17 | 7578335 | 7578503 | 7578353 | 7578483 | 1 | Pool1 |
| CHP2_TP53_4 | ACCAGCCCTGTCGTCTCT | GTGCAGCTGTGGGTTGATTC | GENOME_REGION | COSM43632 | hg19 | chr17 | 7578335 | 7578503 | 7578353 | 7578483 | 1 | Pool1 |
| CHP2_TP53_4 | ACCAGCCCTGTCGTCTCT | GTGCAGCTGTGGGTTGATTC | GENOME_REGION | COSM43635 | hg19 | chr17 | 7578335 | 7578503 | 7578353 | 7578483 | 1 | Pool1 |
| CHP2_TP53_4 | ACCAGCCCTGTCGTCTCT | GTGCAGCTGTGGGTTGATTC | GENOME_REGION | COSM43666 | hg19 | chr17 | 7578335 | 7578503 | 7578353 | 7578483 | 1 | Pool1 |
| CHP2_TP53_4 | ACCAGCCCTGTCGTCTCT | GTGCAGCTGTGGGTTGATTC | GENOME_REGION | COSM43670 | hg19 | chr17 | 7578335 | 7578503 | 7578353 | 7578483 | 1 | Pool1 |
| CHP2_TP53_4 | ACCAGCCCTGTCGTCTCT | GTGCAGCTGTGGGTTGATTC | GENOME_REGION | COSM43675 | hg19 | chr17 | 7578335 | 7578503 | 7578353 | 7578483 | 1 | Pool1 |
| CHP2_TP53_4 | ACCAGCCCTGTCGTCTCT | GTGCAGCTGTGGGTTGATTC | GENOME_REGION | COSM43679 | hg19 | chr17 | 7578335 | 7578503 | 7578353 | 7578483 | 1 | Pool1 |
| CHP2_TP53_4 | ACCAGCCCTGTCGTCTCT | GTGCAGCTGTGGGTTGATTC | GENOME_REGION | COSM43680 | hg19 | chr17 | 7578335 | 7578503 | 7578353 | 7578483 | 1 | Pool1 |
| CHP2_TP53_4 | ACCAGCCCTGTCGTCTCT | GTGCAGCTGTGGGTTGATTC | GENOME_REGION | COSM43689 | hg19 | chr17 | 7578335 | 7578503 | 7578353 | 7578483 | 1 | Pool1 |
| CHP2_TP53_4 | ACCAGCCCTGTCGTCTCT | GTGCAGCTGTGGGTTGATTC | GENOME_REGION | COSM43692 | hg19 | chr17 | 7578335 | 7578503 | 7578353 | 7578483 | 1 | Pool1 |
| CHP2_TP53_4 | ACCAGCCCTGTCGTCTCT | GTGCAGCTGTGGGTTGATTC | GENOME_REGION | COSM43709 | hg19 | chr17 | 7578335 | 7578503 | 7578353 | 7578483 | 1 | Pool1 |
| CHP2_TP53_4 | ACCAGCCCTGTCGTCTCT | GTGCAGCTGTGGGTTGATTC | GENOME_REGION | COSM43710 | hg19 | chr17 | 7578335 | 7578503 | 7578353 | 7578483 | 1 | Pool1 |
| CHP2_TP53_4 | ACCAGCCCTGTCGTCTCT | GTGCAGCTGTGGGTTGATTC | GENOME_REGION | COSM43728 | hg19 | chr17 | 7578335 | 7578503 | 7578353 | 7578483 | 1 | Pool1 |
| CHP2_TP53_4 | ACCAGCCCTGTCGTCTCT | GTGCAGCTGTGGGTTGATTC | GENOME_REGION | COSM43732 | hg19 | chr17 | 7578335 | 7578503 | 7578353 | 7578483 | 1 | Pool1 |
| CHP2_TP53_4 | ACCAGCCCTGTCGTCTCT | GTGCAGCTGTGGGTTGATTC | GENOME_REGION | COSM43734 | hg19 | chr17 | 7578335 | 7578503 | 7578353 | 7578483 | 1 | Pool1 |
| CHP2_TP53_4 | ACCAGCCCTGTCGTCTCT | GTGCAGCTGTGGGTTGATTC | GENOME_REGION | COSM43739 | hg19 | chr17 | 7578335 | 7578503 | 7578353 | 7578483 | 1 | Pool1 |
| CHP2_TP53_4 | ACCAGCCCTGTCGTCTCT | GTGCAGCTGTGGGTTGATTC | GENOME_REGION | COSM43744 | hg19 | chr17 | 7578335 | 7578503 | 7578353 | 7578483 | 1 | Pool1 |
| CHP2_TP53_4 | ACCAGCCCTGTCGTCTCT | GTGCAGCTGTGGGTTGATTC | GENOME_REGION | COSM43772 | hg19 | chr17 | 7578335 | 7578503 | 7578353 | 7578483 | 1 | Pool1 |
| CHP2_TP53_4 | ACCAGCCCTGTCGTCTCT | GTGCAGCTGTGGGTTGATTC | GENOME_REGION | COSM43781 | hg19 | chr17 | 7578335 | 7578503 | 7578353 | 7578483 | 1 | Pool1 |
| CHP2_TP53_4 | ACCAGCCCTGTCGTCTCT | GTGCAGCTGTGGGTTGATTC | GENOME_REGION | COSM43797 | hg19 | chr17 | 7578335 | 7578503 | 7578353 | 7578483 | 1 | Pool1 |
| CHP2_TP53_4 | ACCAGCCCTGTCGTCTCT | GTGCAGCTGTGGGTTGATTC | GENOME_REGION | COSM43820 | hg19 | chr17 | 7578335 | 7578503 | 7578353 | 7578483 | 1 | Pool1 |
| CHP2_TP53_4 | ACCAGCCCTGTCGTCTCT | GTGCAGCTGTGGGTTGATTC | GENOME_REGION | COSM43828 | hg19 | chr17 | 7578335 | 7578503 | 7578353 | 7578483 | 1 | Pool1 |
| CHP2_TP53_4 | ACCAGCCCTGTCGTCTCT | GTGCAGCTGTGGGTTGATTC | GENOME_REGION | COSM43831 | hg19 | chr17 | 7578335 | 7578503 | 7578353 | 7578483 | 1 | Pool1 |
| CHP2_TP53_4 | ACCAGCCCTGTCGTCTCT | GTGCAGCTGTGGGTTGATTC | GENOME_REGION | COSM43836 | hg19 | chr17 | 7578335 | 7578503 | 7578353 | 7578483 | 1 | Pool1 |
| CHP2_TP53_4 | ACCAGCCCTGTCGTCTCT | GTGCAGCTGTGGGTTGATTC | GENOME_REGION | COSM43846 | hg19 | chr17 | 7578335 | 7578503 | 7578353 | 7578483 | 1 | Pool1 |
| CHP2_TP53_4 | ACCAGCCCTGTCGTCTCT | GTGCAGCTGTGGGTTGATTC | GENOME_REGION | COSM43848 | hg19 | chr17 | 7578335 | 7578503 | 7578353 | 7578483 | 1 | Pool1 |
| CHP2_TP53_4 | ACCAGCCCTGTCGTCTCT | GTGCAGCTGTGGGTTGATTC | GENOME_REGION | COSM43851 | hg19 | chr17 | 7578335 | 7578503 | 7578353 | 7578483 | 1 | Pool1 |
| CHP2_TP53_4 | ACCAGCCCTGTCGTCTCT | GTGCAGCTGTGGGTTGATTC | GENOME_REGION | COSM43861 | hg19 | chr17 | 7578335 | 7578503 | 7578353 | 7578483 | 1 | Pool1 |
| CHP2_TP53_4 | ACCAGCCCTGTCGTCTCT | GTGCAGCTGTGGGTTGATTC | GENOME_REGION | COSM43891 | hg19 | chr17 | 7578335 | 7578503 | 7578353 | 7578483 | 1 | Pool1 |
| CHP2_TP53_4 | ACCAGCCCTGTCGTCTCT | GTGCAGCTGTGGGTTGATTC | GENOME_REGION | COSM43898 | hg19 | chr17 | 7578335 | 7578503 | 7578353 | 7578483 | 1 | Pool1 |
| CHP2_TP53_4 | ACCAGCCCTGTCGTCTCT | GTGCAGCTGTGGGTTGATTC | GENOME_REGION | COSM43903 | hg19 | chr17 | 7578335 | 7578503 | 7578353 | 7578483 | 1 | Pool1 |
| CHP2_TP53_4 | ACCAGCCCTGTCGTCTCT | GTGCAGCTGTGGGTTGATTC | GENOME_REGION | COSM43927 | hg19 | chr17 | 7578335 | 7578503 | 7578353 | 7578483 | 1 | Pool1 |
| CHP2_TP53_4 | ACCAGCCCTGTCGTCTCT | GTGCAGCTGTGGGTTGATTC | GENOME_REGION | COSM43931 | hg19 | chr17 | 7578335 | 7578503 | 7578353 | 7578483 | 1 | Pool1 |
| CHP2_TP53_4 | ACCAGCCCTGTCGTCTCT | GTGCAGCTGTGGGTTGATTC | GENOME_REGION | COSM43934 | hg19 | chr17 | 7578335 | 7578503 | 7578353 | 7578483 | 1 | Pool1 |
| CHP2_TP53_4 | ACCAGCCCTGTCGTCTCT | GTGCAGCTGTGGGTTGATTC | GENOME_REGION | COSM43940 | hg19 | chr17 | 7578335 | 7578503 | 7578353 | 7578483 | 1 | Pool1 |
| CHP2_TP53_4 | ACCAGCCCTGTCGTCTCT | GTGCAGCTGTGGGTTGATTC | GENOME_REGION | COSM43955 | hg19 | chr17 | 7578335 | 7578503 | 7578353 | 7578483 | 1 | Pool1 |
| CHP2_TP53_4 | ACCAGCCCTGTCGTCTCT | GTGCAGCTGTGGGTTGATTC | GENOME_REGION | COSM43964 | hg19 | chr17 | 7578335 | 7578503 | 7578353 | 7578483 | 1 | Pool1 |
| CHP2_TP53_4 | ACCAGCCCTGTCGTCTCT | GTGCAGCTGTGGGTTGATTC | GENOME_REGION | COSM43978 | hg19 | chr17 | 7578335 | 7578503 | 7578353 | 7578483 | 1 | Pool1 |
| CHP2_TP53_4 | ACCAGCCCTGTCGTCTCT | GTGCAGCTGTGGGTTGATTC | GENOME_REGION | COSM44009 | hg19 | chr17 | 7578335 | 7578503 | 7578353 | 7578483 | 1 | Pool1 |
| CHP2_TP53_4 | ACCAGCCCTGTCGTCTCT | GTGCAGCTGTGGGTTGATTC | GENOME_REGION | COSM44026 | hg19 | chr17 | 7578335 | 7578503 | 7578353 | 7578483 | 1 | Pool1 |
| CHP2_TP53_4 | ACCAGCCCTGTCGTCTCT | GTGCAGCTGTGGGTTGATTC | GENOME_REGION | COSM44029 | hg19 | chr17 | 7578335 | 7578503 | 7578353 | 7578483 | 1 | Pool1 |
| CHP2_TP53_4 | ACCAGCCCTGTCGTCTCT | GTGCAGCTGTGGGTTGATTC | GENOME_REGION | COSM44033 | hg19 | chr17 | 7578335 | 7578503 | 7578353 | 7578483 | 1 | Pool1 |
| CHP2_TP53_4 | ACCAGCCCTGTCGTCTCT | GTGCAGCTGTGGGTTGATTC | GENOME_REGION | COSM44035 | hg19 | chr17 | 7578335 | 7578503 | 7578353 | 7578483 | 1 | Pool1 |
| CHP2_TP53_4 | ACCAGCCCTGTCGTCTCT | GTGCAGCTGTGGGTTGATTC | GENOME_REGION | COSM44057 | hg19 | chr17 | 7578335 | 7578503 | 7578353 | 7578483 | 1 | Pool1 |
| CHP2_TP53_4 | ACCAGCCCTGTCGTCTCT | GTGCAGCTGTGGGTTGATTC | GENOME_REGION | COSM44061 | hg19 | chr17 | 7578335 | 7578503 | 7578353 | 7578483 | 1 | Pool1 |
| CHP2_TP53_4 | ACCAGCCCTGTCGTCTCT | GTGCAGCTGTGGGTTGATTC | GENOME_REGION | COSM44068 | hg19 | chr17 | 7578335 | 7578503 | 7578353 | 7578483 | 1 | Pool1 |
| CHP2_TP53_4 | ACCAGCCCTGTCGTCTCT | GTGCAGCTGTGGGTTGATTC | GENOME_REGION | COSM44097 | hg19 | chr17 | 7578335 | 7578503 | 7578353 | 7578483 | 1 | Pool1 |
| CHP2_TP53_4 | ACCAGCCCTGTCGTCTCT | GTGCAGCTGTGGGTTGATTC | GENOME_REGION | COSM44119 | hg19 | chr17 | 7578335 | 7578503 | 7578353 | 7578483 | 1 | Pool1 |
| CHP2_TP53_4 | ACCAGCCCTGTCGTCTCT | GTGCAGCTGTGGGTTGATTC | GENOME_REGION | COSM44120 | hg19 | chr17 | 7578335 | 7578503 | 7578353 | 7578483 | 1 | Pool1 |
| CHP2_TP53_4 | ACCAGCCCTGTCGTCTCT | GTGCAGCTGTGGGTTGATTC | GENOME_REGION | COSM44125 | hg19 | chr17 | 7578335 | 7578503 | 7578353 | 7578483 | 1 | Pool1 |
| CHP2_TP53_4 | ACCAGCCCTGTCGTCTCT | GTGCAGCTGTGGGTTGATTC | GENOME_REGION | COSM44126 | hg19 | chr17 | 7578335 | 7578503 | 7578353 | 7578483 | 1 | Pool1 |
| CHP2_TP53_4 | ACCAGCCCTGTCGTCTCT | GTGCAGCTGTGGGTTGATTC | GENOME_REGION | COSM44130 | hg19 | chr17 | 7578335 | 7578503 | 7578353 | 7578483 | 1 | Pool1 |
| CHP2_TP53_4 | ACCAGCCCTGTCGTCTCT | GTGCAGCTGTGGGTTGATTC | GENOME_REGION | COSM44134 | hg19 | chr17 | 7578335 | 7578503 | 7578353 | 7578483 | 1 | Pool1 |
| CHP2_TP53_4 | ACCAGCCCTGTCGTCTCT | GTGCAGCTGTGGGTTGATTC | GENOME_REGION | COSM44146 | hg19 | chr17 | 7578335 | 7578503 | 7578353 | 7578483 | 1 | Pool1 |
| CHP2_TP53_4 | ACCAGCCCTGTCGTCTCT | GTGCAGCTGTGGGTTGATTC | GENOME_REGION | COSM44151 | hg19 | chr17 | 7578335 | 7578503 | 7578353 | 7578483 | 1 | Pool1 |
| CHP2_TP53_4 | ACCAGCCCTGTCGTCTCT | GTGCAGCTGTGGGTTGATTC | GENOME_REGION | COSM44152 | hg19 | chr17 | 7578335 | 7578503 | 7578353 | 7578483 | 1 | Pool1 |
| CHP2_TP53_4 | ACCAGCCCTGTCGTCTCT | GTGCAGCTGTGGGTTGATTC | GENOME_REGION | COSM44185 | hg19 | chr17 | 7578335 | 7578503 | 7578353 | 7578483 | 1 | Pool1 |
| CHP2_TP53_4 | ACCAGCCCTGTCGTCTCT | GTGCAGCTGTGGGTTGATTC | GENOME_REGION | COSM44202 | hg19 | chr17 | 7578335 | 7578503 | 7578353 | 7578483 | 1 | Pool1 |
| CHP2_TP53_4 | ACCAGCCCTGTCGTCTCT | GTGCAGCTGTGGGTTGATTC | GENOME_REGION | COSM44214 | hg19 | chr17 | 7578335 | 7578503 | 7578353 | 7578483 | 1 | Pool1 |
| CHP2_TP53_4 | ACCAGCCCTGTCGTCTCT | GTGCAGCTGTGGGTTGATTC | GENOME_REGION | COSM44215 | hg19 | chr17 | 7578335 | 7578503 | 7578353 | 7578483 | 1 | Pool1 |
| CHP2_TP53_4 | ACCAGCCCTGTCGTCTCT | GTGCAGCTGTGGGTTGATTC | GENOME_REGION | COSM44216 | hg19 | chr17 | 7578335 | 7578503 | 7578353 | 7578483 | 1 | Pool1 |
| CHP2_TP53_4 | ACCAGCCCTGTCGTCTCT | GTGCAGCTGTGGGTTGATTC | GENOME_REGION | COSM44218 | hg19 | chr17 | 7578335 | 7578503 | 7578353 | 7578483 | 1 | Pool1 |
| CHP2_TP53_4 | ACCAGCCCTGTCGTCTCT | GTGCAGCTGTGGGTTGATTC | GENOME_REGION | COSM44229 | hg19 | chr17 | 7578335 | 7578503 | 7578353 | 7578483 | 1 | Pool1 |
| CHP2_TP53_4 | ACCAGCCCTGTCGTCTCT | GTGCAGCTGTGGGTTGATTC | GENOME_REGION | COSM44230 | hg19 | chr17 | 7578335 | 7578503 | 7578353 | 7578483 | 1 | Pool1 |
| CHP2_TP53_4 | ACCAGCCCTGTCGTCTCT | GTGCAGCTGTGGGTTGATTC | GENOME_REGION | COSM44240 | hg19 | chr17 | 7578335 | 7578503 | 7578353 | 7578483 | 1 | Pool1 |
| CHP2_TP53_4 | ACCAGCCCTGTCGTCTCT | GTGCAGCTGTGGGTTGATTC | GENOME_REGION | COSM44267 | hg19 | chr17 | 7578335 | 7578503 | 7578353 | 7578483 | 1 | Pool1 |
| CHP2_TP53_4 | ACCAGCCCTGTCGTCTCT | GTGCAGCTGTGGGTTGATTC | GENOME_REGION | COSM44268 | hg19 | chr17 | 7578335 | 7578503 | 7578353 | 7578483 | 1 | Pool1 |
| CHP2_TP53_4 | ACCAGCCCTGTCGTCTCT | GTGCAGCTGTGGGTTGATTC | GENOME_REGION | COSM44275 | hg19 | chr17 | 7578335 | 7578503 | 7578353 | 7578483 | 1 | Pool1 |
| CHP2_TP53_4 | ACCAGCCCTGTCGTCTCT | GTGCAGCTGTGGGTTGATTC | GENOME_REGION | COSM44282 | hg19 | chr17 | 7578335 | 7578503 | 7578353 | 7578483 | 1 | Pool1 |
| CHP2_TP53_4 | ACCAGCCCTGTCGTCTCT | GTGCAGCTGTGGGTTGATTC | GENOME_REGION | COSM44289 | hg19 | chr17 | 7578335 | 7578503 | 7578353 | 7578483 | 1 | Pool1 |
| CHP2_TP53_4 | ACCAGCCCTGTCGTCTCT | GTGCAGCTGTGGGTTGATTC | GENOME_REGION | COSM44298 | hg19 | chr17 | 7578335 | 7578503 | 7578353 | 7578483 | 1 | Pool1 |
| CHP2_TP53_4 | ACCAGCCCTGTCGTCTCT | GTGCAGCTGTGGGTTGATTC | GENOME_REGION | COSM44299 | hg19 | chr17 | 7578335 | 7578503 | 7578353 | 7578483 | 1 | Pool1 |
| CHP2_TP53_4 | ACCAGCCCTGTCGTCTCT | GTGCAGCTGTGGGTTGATTC | GENOME_REGION | COSM44300 | hg19 | chr17 | 7578335 | 7578503 | 7578353 | 7578483 | 1 | Pool1 |
| CHP2_TP53_4 | ACCAGCCCTGTCGTCTCT | GTGCAGCTGTGGGTTGATTC | GENOME_REGION | COSM44301 | hg19 | chr17 | 7578335 | 7578503 | 7578353 | 7578483 | 1 | Pool1 |
| CHP2_TP53_4 | ACCAGCCCTGTCGTCTCT | GTGCAGCTGTGGGTTGATTC | GENOME_REGION | COSM44303 | hg19 | chr17 | 7578335 | 7578503 | 7578353 | 7578483 | 1 | Pool1 |
| CHP2_TP53_4 | ACCAGCCCTGTCGTCTCT | GTGCAGCTGTGGGTTGATTC | GENOME_REGION | COSM44305 | hg19 | chr17 | 7578335 | 7578503 | 7578353 | 7578483 | 1 | Pool1 |
| CHP2_TP53_4 | ACCAGCCCTGTCGTCTCT | GTGCAGCTGTGGGTTGATTC | GENOME_REGION | COSM44308 | hg19 | chr17 | 7578335 | 7578503 | 7578353 | 7578483 | 1 | Pool1 |
| CHP2_TP53_4 | ACCAGCCCTGTCGTCTCT | GTGCAGCTGTGGGTTGATTC | GENOME_REGION | COSM44312 | hg19 | chr17 | 7578335 | 7578503 | 7578353 | 7578483 | 1 | Pool1 |
| CHP2_TP53_4 | ACCAGCCCTGTCGTCTCT | GTGCAGCTGTGGGTTGATTC | GENOME_REGION | COSM44320 | hg19 | chr17 | 7578335 | 7578503 | 7578353 | 7578483 | 1 | Pool1 |
| CHP2_TP53_4 | ACCAGCCCTGTCGTCTCT | GTGCAGCTGTGGGTTGATTC | GENOME_REGION | COSM44327 | hg19 | chr17 | 7578335 | 7578503 | 7578353 | 7578483 | 1 | Pool1 |
| CHP2_TP53_4 | ACCAGCCCTGTCGTCTCT | GTGCAGCTGTGGGTTGATTC | GENOME_REGION | COSM44328 | hg19 | chr17 | 7578335 | 7578503 | 7578353 | 7578483 | 1 | Pool1 |
| CHP2_TP53_4 | ACCAGCCCTGTCGTCTCT | GTGCAGCTGTGGGTTGATTC | GENOME_REGION | COSM44329 | hg19 | chr17 | 7578335 | 7578503 | 7578353 | 7578483 | 1 | Pool1 |
| CHP2_TP53_4 | ACCAGCCCTGTCGTCTCT | GTGCAGCTGTGGGTTGATTC | GENOME_REGION | COSM44336 | hg19 | chr17 | 7578335 | 7578503 | 7578353 | 7578483 | 1 | Pool1 |
| CHP2_TP53_4 | ACCAGCCCTGTCGTCTCT | GTGCAGCTGTGGGTTGATTC | GENOME_REGION | COSM44343 | hg19 | chr17 | 7578335 | 7578503 | 7578353 | 7578483 | 1 | Pool1 |
| CHP2_TP53_4 | ACCAGCCCTGTCGTCTCT | GTGCAGCTGTGGGTTGATTC | GENOME_REGION | COSM44367 | hg19 | chr17 | 7578335 | 7578503 | 7578353 | 7578483 | 1 | Pool1 |
| CHP2_TP53_4 | ACCAGCCCTGTCGTCTCT | GTGCAGCTGTGGGTTGATTC | GENOME_REGION | COSM44383 | hg19 | chr17 | 7578335 | 7578503 | 7578353 | 7578483 | 1 | Pool1 |
| CHP2_TP53_4 | ACCAGCCCTGTCGTCTCT | GTGCAGCTGTGGGTTGATTC | GENOME_REGION | COSM44384 | hg19 | chr17 | 7578335 | 7578503 | 7578353 | 7578483 | 1 | Pool1 |
| CHP2_TP53_4 | ACCAGCCCTGTCGTCTCT | GTGCAGCTGTGGGTTGATTC | GENOME_REGION | COSM44387 | hg19 | chr17 | 7578335 | 7578503 | 7578353 | 7578483 | 1 | Pool1 |
| CHP2_TP53_4 | ACCAGCCCTGTCGTCTCT | GTGCAGCTGTGGGTTGATTC | GENOME_REGION | COSM44391 | hg19 | chr17 | 7578335 | 7578503 | 7578353 | 7578483 | 1 | Pool1 |
| CHP2_TP53_4 | ACCAGCCCTGTCGTCTCT | GTGCAGCTGTGGGTTGATTC | GENOME_REGION | COSM44413 | hg19 | chr17 | 7578335 | 7578503 | 7578353 | 7578483 | 1 | Pool1 |
| CHP2_TP53_4 | ACCAGCCCTGTCGTCTCT | GTGCAGCTGTGGGTTGATTC | GENOME_REGION | COSM44416 | hg19 | chr17 | 7578335 | 7578503 | 7578353 | 7578483 | 1 | Pool1 |
| CHP2_TP53_4 | ACCAGCCCTGTCGTCTCT | GTGCAGCTGTGGGTTGATTC | GENOME_REGION | COSM44431 | hg19 | chr17 | 7578335 | 7578503 | 7578353 | 7578483 | 1 | Pool1 |
| CHP2_TP53_4 | ACCAGCCCTGTCGTCTCT | GTGCAGCTGTGGGTTGATTC | GENOME_REGION | COSM44467 | hg19 | chr17 | 7578335 | 7578503 | 7578353 | 7578483 | 1 | Pool1 |
| CHP2_TP53_4 | ACCAGCCCTGTCGTCTCT | GTGCAGCTGTGGGTTGATTC | GENOME_REGION | COSM44495 | hg19 | chr17 | 7578335 | 7578503 | 7578353 | 7578483 | 1 | Pool1 |
| CHP2_TP53_4 | ACCAGCCCTGTCGTCTCT | GTGCAGCTGTGGGTTGATTC | GENOME_REGION | COSM44517 | hg19 | chr17 | 7578335 | 7578503 | 7578353 | 7578483 | 1 | Pool1 |
| CHP2_TP53_4 | ACCAGCCCTGTCGTCTCT | GTGCAGCTGTGGGTTGATTC | GENOME_REGION | COSM44518 | hg19 | chr17 | 7578335 | 7578503 | 7578353 | 7578483 | 1 | Pool1 |
| CHP2_TP53_4 | ACCAGCCCTGTCGTCTCT | GTGCAGCTGTGGGTTGATTC | GENOME_REGION | COSM44521 | hg19 | chr17 | 7578335 | 7578503 | 7578353 | 7578483 | 1 | Pool1 |
| CHP2_TP53_4 | ACCAGCCCTGTCGTCTCT | GTGCAGCTGTGGGTTGATTC | GENOME_REGION | COSM44524 | hg19 | chr17 | 7578335 | 7578503 | 7578353 | 7578483 | 1 | Pool1 |
| CHP2_TP53_4 | ACCAGCCCTGTCGTCTCT | GTGCAGCTGTGGGTTGATTC | GENOME_REGION | COSM44526 | hg19 | chr17 | 7578335 | 7578503 | 7578353 | 7578483 | 1 | Pool1 |
| CHP2_TP53_4 | ACCAGCCCTGTCGTCTCT | GTGCAGCTGTGGGTTGATTC | GENOME_REGION | COSM44546 | hg19 | chr17 | 7578335 | 7578503 | 7578353 | 7578483 | 1 | Pool1 |
| CHP2_TP53_4 | ACCAGCCCTGTCGTCTCT | GTGCAGCTGTGGGTTGATTC | GENOME_REGION | COSM44552 | hg19 | chr17 | 7578335 | 7578503 | 7578353 | 7578483 | 1 | Pool1 |
| CHP2_TP53_4 | ACCAGCCCTGTCGTCTCT | GTGCAGCTGTGGGTTGATTC | GENOME_REGION | COSM44561 | hg19 | chr17 | 7578335 | 7578503 | 7578353 | 7578483 | 1 | Pool1 |
| CHP2_TP53_4 | ACCAGCCCTGTCGTCTCT | GTGCAGCTGTGGGTTGATTC | GENOME_REGION | COSM44563 | hg19 | chr17 | 7578335 | 7578503 | 7578353 | 7578483 | 1 | Pool1 |
| CHP2_TP53_4 | ACCAGCCCTGTCGTCTCT | GTGCAGCTGTGGGTTGATTC | GENOME_REGION | COSM44566 | hg19 | chr17 | 7578335 | 7578503 | 7578353 | 7578483 | 1 | Pool1 |
| CHP2_TP53_4 | ACCAGCCCTGTCGTCTCT | GTGCAGCTGTGGGTTGATTC | GENOME_REGION | COSM44613 | hg19 | chr17 | 7578335 | 7578503 | 7578353 | 7578483 | 1 | Pool1 |
| CHP2_TP53_4 | ACCAGCCCTGTCGTCTCT | GTGCAGCTGTGGGTTGATTC | GENOME_REGION | COSM44623 | hg19 | chr17 | 7578335 | 7578503 | 7578353 | 7578483 | 1 | Pool1 |
| CHP2_TP53_4 | ACCAGCCCTGTCGTCTCT | GTGCAGCTGTGGGTTGATTC | GENOME_REGION | COSM44645 | hg19 | chr17 | 7578335 | 7578503 | 7578353 | 7578483 | 1 | Pool1 |
| CHP2_TP53_4 | ACCAGCCCTGTCGTCTCT | GTGCAGCTGTGGGTTGATTC | GENOME_REGION | COSM44659 | hg19 | chr17 | 7578335 | 7578503 | 7578353 | 7578483 | 1 | Pool1 |
| CHP2_TP53_4 | ACCAGCCCTGTCGTCTCT | GTGCAGCTGTGGGTTGATTC | GENOME_REGION | COSM44692 | hg19 | chr17 | 7578335 | 7578503 | 7578353 | 7578483 | 1 | Pool1 |
| CHP2_TP53_4 | ACCAGCCCTGTCGTCTCT | GTGCAGCTGTGGGTTGATTC | GENOME_REGION | COSM44694 | hg19 | chr17 | 7578335 | 7578503 | 7578353 | 7578483 | 1 | Pool1 |
| CHP2_TP53_4 | ACCAGCCCTGTCGTCTCT | GTGCAGCTGTGGGTTGATTC | GENOME_REGION | COSM44700 | hg19 | chr17 | 7578335 | 7578503 | 7578353 | 7578483 | 1 | Pool1 |
| CHP2_TP53_4 | ACCAGCCCTGTCGTCTCT | GTGCAGCTGTGGGTTGATTC | GENOME_REGION | COSM44714 | hg19 | chr17 | 7578335 | 7578503 | 7578353 | 7578483 | 1 | Pool1 |
| CHP2_TP53_4 | ACCAGCCCTGTCGTCTCT | GTGCAGCTGTGGGTTGATTC | GENOME_REGION | COSM44715 | hg19 | chr17 | 7578335 | 7578503 | 7578353 | 7578483 | 1 | Pool1 |
| CHP2_TP53_4 | ACCAGCCCTGTCGTCTCT | GTGCAGCTGTGGGTTGATTC | GENOME_REGION | COSM44725 | hg19 | chr17 | 7578335 | 7578503 | 7578353 | 7578483 | 1 | Pool1 |
| CHP2_TP53_4 | ACCAGCCCTGTCGTCTCT | GTGCAGCTGTGGGTTGATTC | GENOME_REGION | COSM44726 | hg19 | chr17 | 7578335 | 7578503 | 7578353 | 7578483 | 1 | Pool1 |
| CHP2_TP53_4 | ACCAGCCCTGTCGTCTCT | GTGCAGCTGTGGGTTGATTC | GENOME_REGION | COSM44730 | hg19 | chr17 | 7578335 | 7578503 | 7578353 | 7578483 | 1 | Pool1 |
| CHP2_TP53_4 | ACCAGCCCTGTCGTCTCT | GTGCAGCTGTGGGTTGATTC | GENOME_REGION | COSM44732 | hg19 | chr17 | 7578335 | 7578503 | 7578353 | 7578483 | 1 | Pool1 |
| CHP2_TP53_4 | ACCAGCCCTGTCGTCTCT | GTGCAGCTGTGGGTTGATTC | GENOME_REGION | COSM44742 | hg19 | chr17 | 7578335 | 7578503 | 7578353 | 7578483 | 1 | Pool1 |
| CHP2_TP53_4 | ACCAGCCCTGTCGTCTCT | GTGCAGCTGTGGGTTGATTC | GENOME_REGION | COSM44749 | hg19 | chr17 | 7578335 | 7578503 | 7578353 | 7578483 | 1 | Pool1 |
| CHP2_TP53_4 | ACCAGCCCTGTCGTCTCT | GTGCAGCTGTGGGTTGATTC | GENOME_REGION | COSM44759 | hg19 | chr17 | 7578335 | 7578503 | 7578353 | 7578483 | 1 | Pool1 |
| CHP2_TP53_4 | ACCAGCCCTGTCGTCTCT | GTGCAGCTGTGGGTTGATTC | GENOME_REGION | COSM44776 | hg19 | chr17 | 7578335 | 7578503 | 7578353 | 7578483 | 1 | Pool1 |
| CHP2_TP53_4 | ACCAGCCCTGTCGTCTCT | GTGCAGCTGTGGGTTGATTC | GENOME_REGION | COSM44782 | hg19 | chr17 | 7578335 | 7578503 | 7578353 | 7578483 | 1 | Pool1 |
| CHP2_TP53_4 | ACCAGCCCTGTCGTCTCT | GTGCAGCTGTGGGTTGATTC | GENOME_REGION | COSM44788 | hg19 | chr17 | 7578335 | 7578503 | 7578353 | 7578483 | 1 | Pool1 |
| CHP2_TP53_4 | ACCAGCCCTGTCGTCTCT | GTGCAGCTGTGGGTTGATTC | GENOME_REGION | COSM44793 | hg19 | chr17 | 7578335 | 7578503 | 7578353 | 7578483 | 1 | Pool1 |
| CHP2_TP53_4 | ACCAGCCCTGTCGTCTCT | GTGCAGCTGTGGGTTGATTC | GENOME_REGION | COSM44801 | hg19 | chr17 | 7578335 | 7578503 | 7578353 | 7578483 | 1 | Pool1 |
| CHP2_TP53_4 | ACCAGCCCTGTCGTCTCT | GTGCAGCTGTGGGTTGATTC | GENOME_REGION | COSM44808 | hg19 | chr17 | 7578335 | 7578503 | 7578353 | 7578483 | 1 | Pool1 |
| CHP2_TP53_4 | ACCAGCCCTGTCGTCTCT | GTGCAGCTGTGGGTTGATTC | GENOME_REGION | COSM44818 | hg19 | chr17 | 7578335 | 7578503 | 7578353 | 7578483 | 1 | Pool1 |
| CHP2_TP53_4 | ACCAGCCCTGTCGTCTCT | GTGCAGCTGTGGGTTGATTC | GENOME_REGION | COSM44837 | hg19 | chr17 | 7578335 | 7578503 | 7578353 | 7578483 | 1 | Pool1 |
| CHP2_TP53_4 | ACCAGCCCTGTCGTCTCT | GTGCAGCTGTGGGTTGATTC | GENOME_REGION | COSM44841 | hg19 | chr17 | 7578335 | 7578503 | 7578353 | 7578483 | 1 | Pool1 |
| CHP2_TP53_4 | ACCAGCCCTGTCGTCTCT | GTGCAGCTGTGGGTTGATTC | GENOME_REGION | COSM44842 | hg19 | chr17 | 7578335 | 7578503 | 7578353 | 7578483 | 1 | Pool1 |
| CHP2_TP53_4 | ACCAGCCCTGTCGTCTCT | GTGCAGCTGTGGGTTGATTC | GENOME_REGION | COSM44850 | hg19 | chr17 | 7578335 | 7578503 | 7578353 | 7578483 | 1 | Pool1 |
| CHP2_TP53_4 | ACCAGCCCTGTCGTCTCT | GTGCAGCTGTGGGTTGATTC | GENOME_REGION | COSM44851 | hg19 | chr17 | 7578335 | 7578503 | 7578353 | 7578483 | 1 | Pool1 |
| CHP2_TP53_4 | ACCAGCCCTGTCGTCTCT | GTGCAGCTGTGGGTTGATTC | GENOME_REGION | COSM44861 | hg19 | chr17 | 7578335 | 7578503 | 7578353 | 7578483 | 1 | Pool1 |
| CHP2_TP53_4 | ACCAGCCCTGTCGTCTCT | GTGCAGCTGTGGGTTGATTC | GENOME_REGION | COSM44901 | hg19 | chr17 | 7578335 | 7578503 | 7578353 | 7578483 | 1 | Pool1 |
| CHP2_TP53_4 | ACCAGCCCTGTCGTCTCT | GTGCAGCTGTGGGTTGATTC | GENOME_REGION | COSM44948 | hg19 | chr17 | 7578335 | 7578503 | 7578353 | 7578483 | 1 | Pool1 |
| CHP2_TP53_4 | ACCAGCCCTGTCGTCTCT | GTGCAGCTGTGGGTTGATTC | GENOME_REGION | COSM44971 | hg19 | chr17 | 7578335 | 7578503 | 7578353 | 7578483 | 1 | Pool1 |
| CHP2_TP53_4 | ACCAGCCCTGTCGTCTCT | GTGCAGCTGTGGGTTGATTC | GENOME_REGION | COSM44973 | hg19 | chr17 | 7578335 | 7578503 | 7578353 | 7578483 | 1 | Pool1 |
| CHP2_TP53_4 | ACCAGCCCTGTCGTCTCT | GTGCAGCTGTGGGTTGATTC | GENOME_REGION | COSM44996 | hg19 | chr17 | 7578335 | 7578503 | 7578353 | 7578483 | 1 | Pool1 |
| CHP2_TP53_4 | ACCAGCCCTGTCGTCTCT | GTGCAGCTGTGGGTTGATTC | GENOME_REGION | COSM45019 | hg19 | chr17 | 7578335 | 7578503 | 7578353 | 7578483 | 1 | Pool1 |
| CHP2_TP53_4 | ACCAGCCCTGTCGTCTCT | GTGCAGCTGTGGGTTGATTC | GENOME_REGION | COSM45025 | hg19 | chr17 | 7578335 | 7578503 | 7578353 | 7578483 | 1 | Pool1 |
| CHP2_TP53_4 | ACCAGCCCTGTCGTCTCT | GTGCAGCTGTGGGTTGATTC | GENOME_REGION | COSM45046 | hg19 | chr17 | 7578335 | 7578503 | 7578353 | 7578483 | 1 | Pool1 |
| CHP2_TP53_4 | ACCAGCCCTGTCGTCTCT | GTGCAGCTGTGGGTTGATTC | GENOME_REGION | COSM45047 | hg19 | chr17 | 7578335 | 7578503 | 7578353 | 7578483 | 1 | Pool1 |
| CHP2_TP53_4 | ACCAGCCCTGTCGTCTCT | GTGCAGCTGTGGGTTGATTC | GENOME_REGION | COSM45057 | hg19 | chr17 | 7578335 | 7578503 | 7578353 | 7578483 | 1 | Pool1 |
| CHP2_TP53_4 | ACCAGCCCTGTCGTCTCT | GTGCAGCTGTGGGTTGATTC | GENOME_REGION | COSM45103 | hg19 | chr17 | 7578335 | 7578503 | 7578353 | 7578483 | 1 | Pool1 |
| CHP2_TP53_4 | ACCAGCCCTGTCGTCTCT | GTGCAGCTGTGGGTTGATTC | GENOME_REGION | COSM45111 | hg19 | chr17 | 7578335 | 7578503 | 7578353 | 7578483 | 1 | Pool1 |
| CHP2_TP53_4 | ACCAGCCCTGTCGTCTCT | GTGCAGCTGTGGGTTGATTC | GENOME_REGION | COSM45120 | hg19 | chr17 | 7578335 | 7578503 | 7578353 | 7578483 | 1 | Pool1 |
| CHP2_TP53_4 | ACCAGCCCTGTCGTCTCT | GTGCAGCTGTGGGTTGATTC | GENOME_REGION | COSM45154 | hg19 | chr17 | 7578335 | 7578503 | 7578353 | 7578483 | 1 | Pool1 |
| CHP2_TP53_4 | ACCAGCCCTGTCGTCTCT | GTGCAGCTGTGGGTTGATTC | GENOME_REGION | COSM45187 | hg19 | chr17 | 7578335 | 7578503 | 7578353 | 7578483 | 1 | Pool1 |
| CHP2_TP53_4 | ACCAGCCCTGTCGTCTCT | GTGCAGCTGTGGGTTGATTC | GENOME_REGION | COSM45194 | hg19 | chr17 | 7578335 | 7578503 | 7578353 | 7578483 | 1 | Pool1 |
| CHP2_TP53_4 | ACCAGCCCTGTCGTCTCT | GTGCAGCTGTGGGTTGATTC | GENOME_REGION | COSM45198 | hg19 | chr17 | 7578335 | 7578503 | 7578353 | 7578483 | 1 | Pool1 |
| CHP2_TP53_4 | ACCAGCCCTGTCGTCTCT | GTGCAGCTGTGGGTTGATTC | GENOME_REGION | COSM45275 | hg19 | chr17 | 7578335 | 7578503 | 7578353 | 7578483 | 1 | Pool1 |
| CHP2_TP53_4 | ACCAGCCCTGTCGTCTCT | GTGCAGCTGTGGGTTGATTC | GENOME_REGION | COSM45286 | hg19 | chr17 | 7578335 | 7578503 | 7578353 | 7578483 | 1 | Pool1 |
| CHP2_TP53_4 | ACCAGCCCTGTCGTCTCT | GTGCAGCTGTGGGTTGATTC | GENOME_REGION | COSM45314 | hg19 | chr17 | 7578335 | 7578503 | 7578353 | 7578483 | 1 | Pool1 |
| CHP2_TP53_4 | ACCAGCCCTGTCGTCTCT | GTGCAGCTGTGGGTTGATTC | GENOME_REGION | COSM45326 | hg19 | chr17 | 7578335 | 7578503 | 7578353 | 7578483 | 1 | Pool1 |
| CHP2_TP53_4 | ACCAGCCCTGTCGTCTCT | GTGCAGCTGTGGGTTGATTC | GENOME_REGION | COSM45338 | hg19 | chr17 | 7578335 | 7578503 | 7578353 | 7578483 | 1 | Pool1 |
| CHP2_TP53_4 | ACCAGCCCTGTCGTCTCT | GTGCAGCTGTGGGTTGATTC | GENOME_REGION | COSM45342 | hg19 | chr17 | 7578335 | 7578503 | 7578353 | 7578483 | 1 | Pool1 |
| CHP2_TP53_4 | ACCAGCCCTGTCGTCTCT | GTGCAGCTGTGGGTTGATTC | GENOME_REGION | COSM45372 | hg19 | chr17 | 7578335 | 7578503 | 7578353 | 7578483 | 1 | Pool1 |
| CHP2_TP53_4 | ACCAGCCCTGTCGTCTCT | GTGCAGCTGTGGGTTGATTC | GENOME_REGION | COSM45399 | hg19 | chr17 | 7578335 | 7578503 | 7578353 | 7578483 | 1 | Pool1 |
| CHP2_TP53_4 | ACCAGCCCTGTCGTCTCT | GTGCAGCTGTGGGTTGATTC | GENOME_REGION | COSM45411 | hg19 | chr17 | 7578335 | 7578503 | 7578353 | 7578483 | 1 | Pool1 |
| CHP2_TP53_4 | ACCAGCCCTGTCGTCTCT | GTGCAGCTGTGGGTTGATTC | GENOME_REGION | COSM45416 | hg19 | chr17 | 7578335 | 7578503 | 7578353 | 7578483 | 1 | Pool1 |
| CHP2_TP53_4 | ACCAGCCCTGTCGTCTCT | GTGCAGCTGTGGGTTGATTC | GENOME_REGION | COSM45479 | hg19 | chr17 | 7578335 | 7578503 | 7578353 | 7578483 | 1 | Pool1 |
| CHP2_TP53_4 | ACCAGCCCTGTCGTCTCT | GTGCAGCTGTGGGTTGATTC | GENOME_REGION | COSM45490 | hg19 | chr17 | 7578335 | 7578503 | 7578353 | 7578483 | 1 | Pool1 |
| CHP2_TP53_4 | ACCAGCCCTGTCGTCTCT | GTGCAGCTGTGGGTTGATTC | GENOME_REGION | COSM45505 | hg19 | chr17 | 7578335 | 7578503 | 7578353 | 7578483 | 1 | Pool1 |
| CHP2_TP53_4 | ACCAGCCCTGTCGTCTCT | GTGCAGCTGTGGGTTGATTC | GENOME_REGION | COSM45506 | hg19 | chr17 | 7578335 | 7578503 | 7578353 | 7578483 | 1 | Pool1 |
| CHP2_TP53_4 | ACCAGCCCTGTCGTCTCT | GTGCAGCTGTGGGTTGATTC | GENOME_REGION | COSM45515 | hg19 | chr17 | 7578335 | 7578503 | 7578353 | 7578483 | 1 | Pool1 |
| CHP2_TP53_4 | ACCAGCCCTGTCGTCTCT | GTGCAGCTGTGGGTTGATTC | GENOME_REGION | COSM45541 | hg19 | chr17 | 7578335 | 7578503 | 7578353 | 7578483 | 1 | Pool1 |
| CHP2_TP53_4 | ACCAGCCCTGTCGTCTCT | GTGCAGCTGTGGGTTGATTC | GENOME_REGION | COSM45551 | hg19 | chr17 | 7578335 | 7578503 | 7578353 | 7578483 | 1 | Pool1 |
| CHP2_TP53_4 | ACCAGCCCTGTCGTCTCT | GTGCAGCTGTGGGTTGATTC | GENOME_REGION | COSM45562 | hg19 | chr17 | 7578335 | 7578503 | 7578353 | 7578483 | 1 | Pool1 |
| CHP2_TP53_4 | ACCAGCCCTGTCGTCTCT | GTGCAGCTGTGGGTTGATTC | GENOME_REGION | COSM45583 | hg19 | chr17 | 7578335 | 7578503 | 7578353 | 7578483 | 1 | Pool1 |
| CHP2_TP53_4 | ACCAGCCCTGTCGTCTCT | GTGCAGCTGTGGGTTGATTC | GENOME_REGION | COSM45594 | hg19 | chr17 | 7578335 | 7578503 | 7578353 | 7578483 | 1 | Pool1 |
| CHP2_TP53_4 | ACCAGCCCTGTCGTCTCT | GTGCAGCTGTGGGTTGATTC | GENOME_REGION | COSM45622 | hg19 | chr17 | 7578335 | 7578503 | 7578353 | 7578483 | 1 | Pool1 |
| CHP2_TP53_4 | ACCAGCCCTGTCGTCTCT | GTGCAGCTGTGGGTTGATTC | GENOME_REGION | COSM45626 | hg19 | chr17 | 7578335 | 7578503 | 7578353 | 7578483 | 1 | Pool1 |
| CHP2_TP53_4 | ACCAGCCCTGTCGTCTCT | GTGCAGCTGTGGGTTGATTC | GENOME_REGION | COSM45627 | hg19 | chr17 | 7578335 | 7578503 | 7578353 | 7578483 | 1 | Pool1 |
| CHP2_TP53_4 | ACCAGCCCTGTCGTCTCT | GTGCAGCTGTGGGTTGATTC | GENOME_REGION | COSM45660 | hg19 | chr17 | 7578335 | 7578503 | 7578353 | 7578483 | 1 | Pool1 |
| CHP2_TP53_4 | ACCAGCCCTGTCGTCTCT | GTGCAGCTGTGGGTTGATTC | GENOME_REGION | COSM45671 | hg19 | chr17 | 7578335 | 7578503 | 7578353 | 7578483 | 1 | Pool1 |
| CHP2_TP53_4 | ACCAGCCCTGTCGTCTCT | GTGCAGCTGTGGGTTGATTC | GENOME_REGION | COSM45674 | hg19 | chr17 | 7578335 | 7578503 | 7578353 | 7578483 | 1 | Pool1 |
| CHP2_TP53_4 | ACCAGCCCTGTCGTCTCT | GTGCAGCTGTGGGTTGATTC | GENOME_REGION | COSM45711 | hg19 | chr17 | 7578335 | 7578503 | 7578353 | 7578483 | 1 | Pool1 |
| CHP2_TP53_4 | ACCAGCCCTGTCGTCTCT | GTGCAGCTGTGGGTTGATTC | GENOME_REGION | COSM45751 | hg19 | chr17 | 7578335 | 7578503 | 7578353 | 7578483 | 1 | Pool1 |
| CHP2_TP53_4 | ACCAGCCCTGTCGTCTCT | GTGCAGCTGTGGGTTGATTC | GENOME_REGION | COSM45823 | hg19 | chr17 | 7578335 | 7578503 | 7578353 | 7578483 | 1 | Pool1 |
| CHP2_TP53_4 | ACCAGCCCTGTCGTCTCT | GTGCAGCTGTGGGTTGATTC | GENOME_REGION | COSM45838 | hg19 | chr17 | 7578335 | 7578503 | 7578353 | 7578483 | 1 | Pool1 |
| CHP2_TP53_4 | ACCAGCCCTGTCGTCTCT | GTGCAGCTGTGGGTTGATTC | GENOME_REGION | COSM45896 | hg19 | chr17 | 7578335 | 7578503 | 7578353 | 7578483 | 1 | Pool1 |
| CHP2_TP53_4 | ACCAGCCCTGTCGTCTCT | GTGCAGCTGTGGGTTGATTC | GENOME_REGION | COSM45906 | hg19 | chr17 | 7578335 | 7578503 | 7578353 | 7578483 | 1 | Pool1 |
| CHP2_TP53_4 | ACCAGCCCTGTCGTCTCT | GTGCAGCTGTGGGTTGATTC | GENOME_REGION | COSM46001 | hg19 | chr17 | 7578335 | 7578503 | 7578353 | 7578483 | 1 | Pool1 |
| CHP2_TP53_4 | ACCAGCCCTGTCGTCTCT | GTGCAGCTGTGGGTTGATTC | GENOME_REGION | COSM46095 | hg19 | chr17 | 7578335 | 7578503 | 7578353 | 7578483 | 1 | Pool1 |
| CHP2_TP53_4 | ACCAGCCCTGTCGTCTCT | GTGCAGCTGTGGGTTGATTC | GENOME_REGION | COSM46124 | hg19 | chr17 | 7578335 | 7578503 | 7578353 | 7578483 | 1 | Pool1 |
| CHP2_TP53_4 | ACCAGCCCTGTCGTCTCT | GTGCAGCTGTGGGTTGATTC | GENOME_REGION | COSM46163 | hg19 | chr17 | 7578335 | 7578503 | 7578353 | 7578483 | 1 | Pool1 |
| CHP2_TP53_4 | ACCAGCCCTGTCGTCTCT | GTGCAGCTGTGGGTTGATTC | GENOME_REGION | COSM46288 | hg19 | chr17 | 7578335 | 7578503 | 7578353 | 7578483 | 1 | Pool1 |
| CHP2_TP53_4 | ACCAGCCCTGTCGTCTCT | GTGCAGCTGTGGGTTGATTC | GENOME_REGION | COSM46393 | hg19 | chr17 | 7578335 | 7578503 | 7578353 | 7578483 | 1 | Pool1 |
| CHP2_TP53_4 | ACCAGCCCTGTCGTCTCT | GTGCAGCTGTGGGTTGATTC | GENOME_REGION | COSM51646 | hg19 | chr17 | 7578335 | 7578503 | 7578353 | 7578483 | 1 | Pool1 |
| CHP2_TP53_4 | ACCAGCCCTGTCGTCTCT | GTGCAGCTGTGGGTTGATTC | GENOME_REGION | COSM6815 | hg19 | chr17 | 7578335 | 7578503 | 7578353 | 7578483 | 1 | Pool1 |
| CHP2_TP53_4 | ACCAGCCCTGTCGTCTCT | GTGCAGCTGTGGGTTGATTC | GENOME_REGION | COSM6901 | hg19 | chr17 | 7578335 | 7578503 | 7578353 | 7578483 | 1 | Pool1 |
| CHP2_TP53_4 | ACCAGCCCTGTCGTCTCT | GTGCAGCTGTGGGTTGATTC | GENOME_REGION | COSM98964 | hg19 | chr17 | 7578335 | 7578503 | 7578353 | 7578483 | 1 | Pool1 |
| CHP2_TP53_4 | ACCAGCCCTGTCGTCTCT | GTGCAGCTGTGGGTTGATTC | GENOME_REGION | COSM98965 | hg19 | chr17 | 7578335 | 7578503 | 7578353 | 7578483 | 1 | Pool1 |
| CHP2_TP53_4 | ACCAGCCCTGTCGTCTCT | GTGCAGCTGTGGGTTGATTC | GENOME_REGION | COSM98966 | hg19 | chr17 | 7578335 | 7578503 | 7578353 | 7578483 | 1 | Pool1 |
| CHP2_TP53_4 | ACCAGCCCTGTCGTCTCT | GTGCAGCTGTGGGTTGATTC | GENOME_REGION | COSM99022 | hg19 | chr17 | 7578335 | 7578503 | 7578353 | 7578483 | 1 | Pool1 |
| CHP2_TP53_4 | ACCAGCCCTGTCGTCTCT | GTGCAGCTGTGGGTTGATTC | GENOME_REGION | COSM99023 | hg19 | chr17 | 7578335 | 7578503 | 7578353 | 7578483 | 1 | Pool1 |
| CHP2_TP53_4 | ACCAGCCCTGTCGTCTCT | GTGCAGCTGTGGGTTGATTC | GENOME_REGION | COSM99024 | hg19 | chr17 | 7578335 | 7578503 | 7578353 | 7578483 | 1 | Pool1 |
| CHP2_TP53_4 | ACCAGCCCTGTCGTCTCT | GTGCAGCTGTGGGTTGATTC | GENOME_REGION | COSM99638 | hg19 | chr17 | 7578335 | 7578503 | 7578353 | 7578483 | 1 | Pool1 |
| CHP2_TP53_4 | ACCAGCCCTGTCGTCTCT | GTGCAGCTGTGGGTTGATTC | GENOME_REGION | COSM99639 | hg19 | chr17 | 7578335 | 7578503 | 7578353 | 7578483 | 1 | Pool1 |
| CHP2_TP53_4 | ACCAGCCCTGTCGTCTCT | GTGCAGCTGTGGGTTGATTC | GENOME_REGION | COSM99640 | hg19 | chr17 | 7578335 | 7578503 | 7578353 | 7578483 | 1 | Pool1 |
| CHP2_TP53_4 | ACCAGCCCTGTCGTCTCT | GTGCAGCTGTGGGTTGATTC | GENOME_REGION | COSM99641 | hg19 | chr17 | 7578335 | 7578503 | 7578353 | 7578483 | 1 | Pool1 |
| CHP2_TP53_4 | ACCAGCCCTGTCGTCTCT | GTGCAGCTGTGGGTTGATTC | GENOME_REGION | COSM99914 | hg19 | chr17 | 7578335 | 7578503 | 7578353 | 7578483 | 1 | Pool1 |
| CHP2_TP53_5 | CCAGTTGCAAACCAGACCTCA | AGGCCTCTGATTCCTCACTGAT | GENOME_REGION | COSM10654 | hg19 | chr17 | 7578160 | 7578320 | 7578181 | 7578298 | 1 | Pool1 |
| CHP2_TP53_5 | CCAGTTGCAAACCAGACCTCA | AGGCCTCTGATTCCTCACTGAT | GENOME_REGION | COSM10667 | hg19 | chr17 | 7578160 | 7578320 | 7578181 | 7578298 | 1 | Pool1 |
| CHP2_TP53_5 | CCAGTTGCAAACCAGACCTCA | AGGCCTCTGATTCCTCACTGAT | GENOME_REGION | COSM10672 | hg19 | chr17 | 7578160 | 7578320 | 7578181 | 7578298 | 1 | Pool1 |
| CHP2_TP53_5 | CCAGTTGCAAACCAGACCTCA | AGGCCTCTGATTCCTCACTGAT | GENOME_REGION | COSM10705 | hg19 | chr17 | 7578160 | 7578320 | 7578181 | 7578298 | 1 | Pool1 |
| CHP2_TP53_5 | CCAGTTGCAAACCAGACCTCA | AGGCCTCTGATTCCTCACTGAT | GENOME_REGION | COSM10733 | hg19 | chr17 | 7578160 | 7578320 | 7578181 | 7578298 | 1 | Pool1 |
| CHP2_TP53_5 | CCAGTTGCAAACCAGACCTCA | AGGCCTCTGATTCCTCACTGAT | GENOME_REGION | COSM10735 | hg19 | chr17 | 7578160 | 7578320 | 7578181 | 7578298 | 1 | Pool1 |
| CHP2_TP53_5 | CCAGTTGCAAACCAGACCTCA | AGGCCTCTGATTCCTCACTGAT | GENOME_REGION | COSM10742 | hg19 | chr17 | 7578160 | 7578320 | 7578181 | 7578298 | 1 | Pool1 |
| CHP2_TP53_5 | CCAGTTGCAAACCAGACCTCA | AGGCCTCTGATTCCTCACTGAT | GENOME_REGION | COSM10758 | hg19 | chr17 | 7578160 | 7578320 | 7578181 | 7578298 | 1 | Pool1 |
| CHP2_TP53_5 | CCAGTTGCAAACCAGACCTCA | AGGCCTCTGATTCCTCACTGAT | GENOME_REGION | COSM10804 | hg19 | chr17 | 7578160 | 7578320 | 7578181 | 7578298 | 1 | Pool1 |
| CHP2_TP53_5 | CCAGTTGCAAACCAGACCTCA | AGGCCTCTGATTCCTCACTGAT | GENOME_REGION | COSM10995 | hg19 | chr17 | 7578160 | 7578320 | 7578181 | 7578298 | 1 | Pool1 |
| CHP2_TP53_5 | CCAGTTGCAAACCAGACCTCA | AGGCCTCTGATTCCTCACTGAT | GENOME_REGION | COSM11066 | hg19 | chr17 | 7578160 | 7578320 | 7578181 | 7578298 | 1 | Pool1 |
| CHP2_TP53_5 | CCAGTTGCAAACCAGACCTCA | AGGCCTCTGATTCCTCACTGAT | GENOME_REGION | COSM11089 | hg19 | chr17 | 7578160 | 7578320 | 7578181 | 7578298 | 1 | Pool1 |
| CHP2_TP53_5 | CCAGTTGCAAACCAGACCTCA | AGGCCTCTGATTCCTCACTGAT | GENOME_REGION | COSM111721 | hg19 | chr17 | 7578160 | 7578320 | 7578181 | 7578298 | 1 | Pool1 |
| CHP2_TP53_5 | CCAGTTGCAAACCAGACCTCA | AGGCCTCTGATTCCTCACTGAT | GENOME_REGION | COSM111722 | hg19 | chr17 | 7578160 | 7578320 | 7578181 | 7578298 | 1 | Pool1 |
| CHP2_TP53_5 | CCAGTTGCAAACCAGACCTCA | AGGCCTCTGATTCCTCACTGAT | GENOME_REGION | COSM111723 | hg19 | chr17 | 7578160 | 7578320 | 7578181 | 7578298 | 1 | Pool1 |
| CHP2_TP53_5 | CCAGTTGCAAACCAGACCTCA | AGGCCTCTGATTCCTCACTGAT | GENOME_REGION | COSM111724 | hg19 | chr17 | 7578160 | 7578320 | 7578181 | 7578298 | 1 | Pool1 |
| CHP2_TP53_5 | CCAGTTGCAAACCAGACCTCA | AGGCCTCTGATTCCTCACTGAT | GENOME_REGION | COSM11210 | hg19 | chr17 | 7578160 | 7578320 | 7578181 | 7578298 | 1 | Pool1 |
| CHP2_TP53_5 | CCAGTTGCAAACCAGACCTCA | AGGCCTCTGATTCCTCACTGAT | GENOME_REGION | COSM11290 | hg19 | chr17 | 7578160 | 7578320 | 7578181 | 7578298 | 1 | Pool1 |
| CHP2_TP53_5 | CCAGTTGCAAACCAGACCTCA | AGGCCTCTGATTCCTCACTGAT | GENOME_REGION | COSM11307 | hg19 | chr17 | 7578160 | 7578320 | 7578181 | 7578298 | 1 | Pool1 |
| CHP2_TP53_5 | CCAGTTGCAAACCAGACCTCA | AGGCCTCTGATTCCTCACTGAT | GENOME_REGION | COSM11351 | hg19 | chr17 | 7578160 | 7578320 | 7578181 | 7578298 | 1 | Pool1 |
| CHP2_TP53_5 | CCAGTTGCAAACCAGACCTCA | AGGCCTCTGATTCCTCACTGAT | GENOME_REGION | COSM11450 | hg19 | chr17 | 7578160 | 7578320 | 7578181 | 7578298 | 1 | Pool1 |
| CHP2_TP53_5 | CCAGTTGCAAACCAGACCTCA | AGGCCTCTGATTCCTCACTGAT | GENOME_REGION | COSM117946 | hg19 | chr17 | 7578160 | 7578320 | 7578181 | 7578298 | 1 | Pool1 |
| CHP2_TP53_5 | CCAGTTGCAAACCAGACCTCA | AGGCCTCTGATTCCTCACTGAT | GENOME_REGION | COSM117947 | hg19 | chr17 | 7578160 | 7578320 | 7578181 | 7578298 | 1 | Pool1 |
| CHP2_TP53_5 | CCAGTTGCAAACCAGACCTCA | AGGCCTCTGATTCCTCACTGAT | GENOME_REGION | COSM117948 | hg19 | chr17 | 7578160 | 7578320 | 7578181 | 7578298 | 1 | Pool1 |
| CHP2_TP53_5 | CCAGTTGCAAACCAGACCTCA | AGGCCTCTGATTCCTCACTGAT | GENOME_REGION | COSM117949 | hg19 | chr17 | 7578160 | 7578320 | 7578181 | 7578298 | 1 | Pool1 |
| CHP2_TP53_5 | CCAGTTGCAAACCAGACCTCA | AGGCCTCTGATTCCTCACTGAT | GENOME_REGION | COSM118010 | hg19 | chr17 | 7578160 | 7578320 | 7578181 | 7578298 | 1 | Pool1 |
| CHP2_TP53_5 | CCAGTTGCAAACCAGACCTCA | AGGCCTCTGATTCCTCACTGAT | GENOME_REGION | COSM118011 | hg19 | chr17 | 7578160 | 7578320 | 7578181 | 7578298 | 1 | Pool1 |
| CHP2_TP53_5 | CCAGTTGCAAACCAGACCTCA | AGGCCTCTGATTCCTCACTGAT | GENOME_REGION | COSM118012 | hg19 | chr17 | 7578160 | 7578320 | 7578181 | 7578298 | 1 | Pool1 |
| CHP2_TP53_5 | CCAGTTGCAAACCAGACCTCA | AGGCCTCTGATTCCTCACTGAT | GENOME_REGION | COSM118013 | hg19 | chr17 | 7578160 | 7578320 | 7578181 | 7578298 | 1 | Pool1 |
| CHP2_TP53_5 | CCAGTTGCAAACCAGACCTCA | AGGCCTCTGATTCCTCACTGAT | GENOME_REGION | COSM11847 | hg19 | chr17 | 7578160 | 7578320 | 7578181 | 7578298 | 1 | Pool1 |
| CHP2_TP53_5 | CCAGTTGCAAACCAGACCTCA | AGGCCTCTGATTCCTCACTGAT | GENOME_REGION | COSM11860 | hg19 | chr17 | 7578160 | 7578320 | 7578181 | 7578298 | 1 | Pool1 |
| CHP2_TP53_5 | CCAGTTGCAAACCAGACCTCA | AGGCCTCTGATTCCTCACTGAT | GENOME_REGION | COSM13120 | hg19 | chr17 | 7578160 | 7578320 | 7578181 | 7578298 | 1 | Pool1 |
| CHP2_TP53_5 | CCAGTTGCAAACCAGACCTCA | AGGCCTCTGATTCCTCACTGAT | GENOME_REGION | COSM18657 | hg19 | chr17 | 7578160 | 7578320 | 7578181 | 7578298 | 1 | Pool1 |
| CHP2_TP53_5 | CCAGTTGCAAACCAGACCTCA | AGGCCTCTGATTCCTCACTGAT | GENOME_REGION | COSM241997 | hg19 | chr17 | 7578160 | 7578320 | 7578181 | 7578298 | 1 | Pool1 |
| CHP2_TP53_5 | CCAGTTGCAAACCAGACCTCA | AGGCCTCTGATTCCTCACTGAT | GENOME_REGION | COSM241998 | hg19 | chr17 | 7578160 | 7578320 | 7578181 | 7578298 | 1 | Pool1 |
| CHP2_TP53_5 | CCAGTTGCAAACCAGACCTCA | AGGCCTCTGATTCCTCACTGAT | GENOME_REGION | COSM241999 | hg19 | chr17 | 7578160 | 7578320 | 7578181 | 7578298 | 1 | Pool1 |
| CHP2_TP53_5 | CCAGTTGCAAACCAGACCTCA | AGGCCTCTGATTCCTCACTGAT | GENOME_REGION | COSM242000 | hg19 | chr17 | 7578160 | 7578320 | 7578181 | 7578298 | 1 | Pool1 |
| CHP2_TP53_5 | CCAGTTGCAAACCAGACCTCA | AGGCCTCTGATTCCTCACTGAT | GENOME_REGION | COSM39455 | hg19 | chr17 | 7578160 | 7578320 | 7578181 | 7578298 | 1 | Pool1 |
| CHP2_TP53_5 | CCAGTTGCAAACCAGACCTCA | AGGCCTCTGATTCCTCACTGAT | GENOME_REGION | COSM42811 | hg19 | chr17 | 7578160 | 7578320 | 7578181 | 7578298 | 1 | Pool1 |
| CHP2_TP53_5 | CCAGTTGCAAACCAGACCTCA | AGGCCTCTGATTCCTCACTGAT | GENOME_REGION | COSM43537 | hg19 | chr17 | 7578160 | 7578320 | 7578181 | 7578298 | 1 | Pool1 |
| CHP2_TP53_5 | CCAGTTGCAAACCAGACCTCA | AGGCCTCTGATTCCTCACTGAT | GENOME_REGION | COSM43594 | hg19 | chr17 | 7578160 | 7578320 | 7578181 | 7578298 | 1 | Pool1 |
| CHP2_TP53_5 | CCAGTTGCAAACCAGACCTCA | AGGCCTCTGATTCCTCACTGAT | GENOME_REGION | COSM43599 | hg19 | chr17 | 7578160 | 7578320 | 7578181 | 7578298 | 1 | Pool1 |
| CHP2_TP53_5 | CCAGTTGCAAACCAGACCTCA | AGGCCTCTGATTCCTCACTGAT | GENOME_REGION | COSM43608 | hg19 | chr17 | 7578160 | 7578320 | 7578181 | 7578298 | 1 | Pool1 |
| CHP2_TP53_5 | CCAGTTGCAAACCAGACCTCA | AGGCCTCTGATTCCTCACTGAT | GENOME_REGION | COSM43623 | hg19 | chr17 | 7578160 | 7578320 | 7578181 | 7578298 | 1 | Pool1 |
| CHP2_TP53_5 | CCAGTTGCAAACCAGACCTCA | AGGCCTCTGATTCCTCACTGAT | GENOME_REGION | COSM43641 | hg19 | chr17 | 7578160 | 7578320 | 7578181 | 7578298 | 1 | Pool1 |
| CHP2_TP53_5 | CCAGTTGCAAACCAGACCTCA | AGGCCTCTGATTCCTCACTGAT | GENOME_REGION | COSM43642 | hg19 | chr17 | 7578160 | 7578320 | 7578181 | 7578298 | 1 | Pool1 |
| CHP2_TP53_5 | CCAGTTGCAAACCAGACCTCA | AGGCCTCTGATTCCTCACTGAT | GENOME_REGION | COSM43650 | hg19 | chr17 | 7578160 | 7578320 | 7578181 | 7578298 | 1 | Pool1 |
| CHP2_TP53_5 | CCAGTTGCAAACCAGACCTCA | AGGCCTCTGATTCCTCACTGAT | GENOME_REGION | COSM43657 | hg19 | chr17 | 7578160 | 7578320 | 7578181 | 7578298 | 1 | Pool1 |
| CHP2_TP53_5 | CCAGTTGCAAACCAGACCTCA | AGGCCTCTGATTCCTCACTGAT | GENOME_REGION | COSM43681 | hg19 | chr17 | 7578160 | 7578320 | 7578181 | 7578298 | 1 | Pool1 |
| CHP2_TP53_5 | CCAGTTGCAAACCAGACCTCA | AGGCCTCTGATTCCTCACTGAT | GENOME_REGION | COSM43687 | hg19 | chr17 | 7578160 | 7578320 | 7578181 | 7578298 | 1 | Pool1 |
| CHP2_TP53_5 | CCAGTTGCAAACCAGACCTCA | AGGCCTCTGATTCCTCACTGAT | GENOME_REGION | COSM43690 | hg19 | chr17 | 7578160 | 7578320 | 7578181 | 7578298 | 1 | Pool1 |
| CHP2_TP53_5 | CCAGTTGCAAACCAGACCTCA | AGGCCTCTGATTCCTCACTGAT | GENOME_REGION | COSM43698 | hg19 | chr17 | 7578160 | 7578320 | 7578181 | 7578298 | 1 | Pool1 |
| CHP2_TP53_5 | CCAGTTGCAAACCAGACCTCA | AGGCCTCTGATTCCTCACTGAT | GENOME_REGION | COSM43702 | hg19 | chr17 | 7578160 | 7578320 | 7578181 | 7578298 | 1 | Pool1 |
| CHP2_TP53_5 | CCAGTTGCAAACCAGACCTCA | AGGCCTCTGATTCCTCACTGAT | GENOME_REGION | COSM43749 | hg19 | chr17 | 7578160 | 7578320 | 7578181 | 7578298 | 1 | Pool1 |
| CHP2_TP53_5 | CCAGTTGCAAACCAGACCTCA | AGGCCTCTGATTCCTCACTGAT | GENOME_REGION | COSM43753 | hg19 | chr17 | 7578160 | 7578320 | 7578181 | 7578298 | 1 | Pool1 |
| CHP2_TP53_5 | CCAGTTGCAAACCAGACCTCA | AGGCCTCTGATTCCTCACTGAT | GENOME_REGION | COSM43761 | hg19 | chr17 | 7578160 | 7578320 | 7578181 | 7578298 | 1 | Pool1 |
| CHP2_TP53_5 | CCAGTTGCAAACCAGACCTCA | AGGCCTCTGATTCCTCACTGAT | GENOME_REGION | COSM43777 | hg19 | chr17 | 7578160 | 7578320 | 7578181 | 7578298 | 1 | Pool1 |
| CHP2_TP53_5 | CCAGTTGCAAACCAGACCTCA | AGGCCTCTGATTCCTCACTGAT | GENOME_REGION | COSM43779 | hg19 | chr17 | 7578160 | 7578320 | 7578181 | 7578298 | 1 | Pool1 |
| CHP2_TP53_5 | CCAGTTGCAAACCAGACCTCA | AGGCCTCTGATTCCTCACTGAT | GENOME_REGION | COSM43782 | hg19 | chr17 | 7578160 | 7578320 | 7578181 | 7578298 | 1 | Pool1 |
| CHP2_TP53_5 | CCAGTTGCAAACCAGACCTCA | AGGCCTCTGATTCCTCACTGAT | GENOME_REGION | COSM43793 | hg19 | chr17 | 7578160 | 7578320 | 7578181 | 7578298 | 1 | Pool1 |
| CHP2_TP53_5 | CCAGTTGCAAACCAGACCTCA | AGGCCTCTGATTCCTCACTGAT | GENOME_REGION | COSM43807 | hg19 | chr17 | 7578160 | 7578320 | 7578181 | 7578298 | 1 | Pool1 |
| CHP2_TP53_5 | CCAGTTGCAAACCAGACCTCA | AGGCCTCTGATTCCTCACTGAT | GENOME_REGION | COSM43814 | hg19 | chr17 | 7578160 | 7578320 | 7578181 | 7578298 | 1 | Pool1 |
| CHP2_TP53_5 | CCAGTTGCAAACCAGACCTCA | AGGCCTCTGATTCCTCACTGAT | GENOME_REGION | COSM43827 | hg19 | chr17 | 7578160 | 7578320 | 7578181 | 7578298 | 1 | Pool1 |
| CHP2_TP53_5 | CCAGTTGCAAACCAGACCTCA | AGGCCTCTGATTCCTCACTGAT | GENOME_REGION | COSM43833 | hg19 | chr17 | 7578160 | 7578320 | 7578181 | 7578298 | 1 | Pool1 |
| CHP2_TP53_5 | CCAGTTGCAAACCAGACCTCA | AGGCCTCTGATTCCTCACTGAT | GENOME_REGION | COSM43841 | hg19 | chr17 | 7578160 | 7578320 | 7578181 | 7578298 | 1 | Pool1 |
| CHP2_TP53_5 | CCAGTTGCAAACCAGACCTCA | AGGCCTCTGATTCCTCACTGAT | GENOME_REGION | COSM43844 | hg19 | chr17 | 7578160 | 7578320 | 7578181 | 7578298 | 1 | Pool1 |
| CHP2_TP53_5 | CCAGTTGCAAACCAGACCTCA | AGGCCTCTGATTCCTCACTGAT | GENOME_REGION | COSM43850 | hg19 | chr17 | 7578160 | 7578320 | 7578181 | 7578298 | 1 | Pool1 |
| CHP2_TP53_5 | CCAGTTGCAAACCAGACCTCA | AGGCCTCTGATTCCTCACTGAT | GENOME_REGION | COSM43859 | hg19 | chr17 | 7578160 | 7578320 | 7578181 | 7578298 | 1 | Pool1 |
| CHP2_TP53_5 | CCAGTTGCAAACCAGACCTCA | AGGCCTCTGATTCCTCACTGAT | GENOME_REGION | COSM43872 | hg19 | chr17 | 7578160 | 7578320 | 7578181 | 7578298 | 1 | Pool1 |
| CHP2_TP53_5 | CCAGTTGCAAACCAGACCTCA | AGGCCTCTGATTCCTCACTGAT | GENOME_REGION | COSM43905 | hg19 | chr17 | 7578160 | 7578320 | 7578181 | 7578298 | 1 | Pool1 |
| CHP2_TP53_5 | CCAGTTGCAAACCAGACCTCA | AGGCCTCTGATTCCTCACTGAT | GENOME_REGION | COSM43928 | hg19 | chr17 | 7578160 | 7578320 | 7578181 | 7578298 | 1 | Pool1 |
| CHP2_TP53_5 | CCAGTTGCAAACCAGACCTCA | AGGCCTCTGATTCCTCACTGAT | GENOME_REGION | COSM43929 | hg19 | chr17 | 7578160 | 7578320 | 7578181 | 7578298 | 1 | Pool1 |
| CHP2_TP53_5 | CCAGTTGCAAACCAGACCTCA | AGGCCTCTGATTCCTCACTGAT | GENOME_REGION | COSM43935 | hg19 | chr17 | 7578160 | 7578320 | 7578181 | 7578298 | 1 | Pool1 |
| CHP2_TP53_5 | CCAGTTGCAAACCAGACCTCA | AGGCCTCTGATTCCTCACTGAT | GENOME_REGION | COSM43939 | hg19 | chr17 | 7578160 | 7578320 | 7578181 | 7578298 | 1 | Pool1 |
| CHP2_TP53_5 | CCAGTTGCAAACCAGACCTCA | AGGCCTCTGATTCCTCACTGAT | GENOME_REGION | COSM43947 | hg19 | chr17 | 7578160 | 7578320 | 7578181 | 7578298 | 1 | Pool1 |
| CHP2_TP53_5 | CCAGTTGCAAACCAGACCTCA | AGGCCTCTGATTCCTCACTGAT | GENOME_REGION | COSM43951 | hg19 | chr17 | 7578160 | 7578320 | 7578181 | 7578298 | 1 | Pool1 |
| CHP2_TP53_5 | CCAGTTGCAAACCAGACCTCA | AGGCCTCTGATTCCTCACTGAT | GENOME_REGION | COSM43987 | hg19 | chr17 | 7578160 | 7578320 | 7578181 | 7578298 | 1 | Pool1 |
| CHP2_TP53_5 | CCAGTTGCAAACCAGACCTCA | AGGCCTCTGATTCCTCACTGAT | GENOME_REGION | COSM43989 | hg19 | chr17 | 7578160 | 7578320 | 7578181 | 7578298 | 1 | Pool1 |
| CHP2_TP53_5 | CCAGTTGCAAACCAGACCTCA | AGGCCTCTGATTCCTCACTGAT | GENOME_REGION | COSM43990 | hg19 | chr17 | 7578160 | 7578320 | 7578181 | 7578298 | 1 | Pool1 |
| CHP2_TP53_5 | CCAGTTGCAAACCAGACCTCA | AGGCCTCTGATTCCTCACTGAT | GENOME_REGION | COSM44002 | hg19 | chr17 | 7578160 | 7578320 | 7578181 | 7578298 | 1 | Pool1 |
| CHP2_TP53_5 | CCAGTTGCAAACCAGACCTCA | AGGCCTCTGATTCCTCACTGAT | GENOME_REGION | COSM44004 | hg19 | chr17 | 7578160 | 7578320 | 7578181 | 7578298 | 1 | Pool1 |
| CHP2_TP53_5 | CCAGTTGCAAACCAGACCTCA | AGGCCTCTGATTCCTCACTGAT | GENOME_REGION | COSM44011 | hg19 | chr17 | 7578160 | 7578320 | 7578181 | 7578298 | 1 | Pool1 |
| CHP2_TP53_5 | CCAGTTGCAAACCAGACCTCA | AGGCCTCTGATTCCTCACTGAT | GENOME_REGION | COSM44023 | hg19 | chr17 | 7578160 | 7578320 | 7578181 | 7578298 | 1 | Pool1 |
| CHP2_TP53_5 | CCAGTTGCAAACCAGACCTCA | AGGCCTCTGATTCCTCACTGAT | GENOME_REGION | COSM44074 | hg19 | chr17 | 7578160 | 7578320 | 7578181 | 7578298 | 1 | Pool1 |
| CHP2_TP53_5 | CCAGTTGCAAACCAGACCTCA | AGGCCTCTGATTCCTCACTGAT | GENOME_REGION | COSM44076 | hg19 | chr17 | 7578160 | 7578320 | 7578181 | 7578298 | 1 | Pool1 |
| CHP2_TP53_5 | CCAGTTGCAAACCAGACCTCA | AGGCCTCTGATTCCTCACTGAT | GENOME_REGION | COSM44093 | hg19 | chr17 | 7578160 | 7578320 | 7578181 | 7578298 | 1 | Pool1 |
| CHP2_TP53_5 | CCAGTTGCAAACCAGACCTCA | AGGCCTCTGATTCCTCACTGAT | GENOME_REGION | COSM44102 | hg19 | chr17 | 7578160 | 7578320 | 7578181 | 7578298 | 1 | Pool1 |
| CHP2_TP53_5 | CCAGTTGCAAACCAGACCTCA | AGGCCTCTGATTCCTCACTGAT | GENOME_REGION | COSM44112 | hg19 | chr17 | 7578160 | 7578320 | 7578181 | 7578298 | 1 | Pool1 |
| CHP2_TP53_5 | CCAGTTGCAAACCAGACCTCA | AGGCCTCTGATTCCTCACTGAT | GENOME_REGION | COSM44140 | hg19 | chr17 | 7578160 | 7578320 | 7578181 | 7578298 | 1 | Pool1 |
| CHP2_TP53_5 | CCAGTTGCAAACCAGACCTCA | AGGCCTCTGATTCCTCACTGAT | GENOME_REGION | COSM44157 | hg19 | chr17 | 7578160 | 7578320 | 7578181 | 7578298 | 1 | Pool1 |
| CHP2_TP53_5 | CCAGTTGCAAACCAGACCTCA | AGGCCTCTGATTCCTCACTGAT | GENOME_REGION | COSM44162 | hg19 | chr17 | 7578160 | 7578320 | 7578181 | 7578298 | 1 | Pool1 |
| CHP2_TP53_5 | CCAGTTGCAAACCAGACCTCA | AGGCCTCTGATTCCTCACTGAT | GENOME_REGION | COSM44169 | hg19 | chr17 | 7578160 | 7578320 | 7578181 | 7578298 | 1 | Pool1 |
| CHP2_TP53_5 | CCAGTTGCAAACCAGACCTCA | AGGCCTCTGATTCCTCACTGAT | GENOME_REGION | COSM44172 | hg19 | chr17 | 7578160 | 7578320 | 7578181 | 7578298 | 1 | Pool1 |
| CHP2_TP53_5 | CCAGTTGCAAACCAGACCTCA | AGGCCTCTGATTCCTCACTGAT | GENOME_REGION | COSM44174 | hg19 | chr17 | 7578160 | 7578320 | 7578181 | 7578298 | 1 | Pool1 |
| CHP2_TP53_5 | CCAGTTGCAAACCAGACCTCA | AGGCCTCTGATTCCTCACTGAT | GENOME_REGION | COSM44175 | hg19 | chr17 | 7578160 | 7578320 | 7578181 | 7578298 | 1 | Pool1 |
| CHP2_TP53_5 | CCAGTTGCAAACCAGACCTCA | AGGCCTCTGATTCCTCACTGAT | GENOME_REGION | COSM44198 | hg19 | chr17 | 7578160 | 7578320 | 7578181 | 7578298 | 1 | Pool1 |
| CHP2_TP53_5 | CCAGTTGCAAACCAGACCTCA | AGGCCTCTGATTCCTCACTGAT | GENOME_REGION | COSM44234 | hg19 | chr17 | 7578160 | 7578320 | 7578181 | 7578298 | 1 | Pool1 |
| CHP2_TP53_5 | CCAGTTGCAAACCAGACCTCA | AGGCCTCTGATTCCTCACTGAT | GENOME_REGION | COSM44238 | hg19 | chr17 | 7578160 | 7578320 | 7578181 | 7578298 | 1 | Pool1 |
| CHP2_TP53_5 | CCAGTTGCAAACCAGACCTCA | AGGCCTCTGATTCCTCACTGAT | GENOME_REGION | COSM44239 | hg19 | chr17 | 7578160 | 7578320 | 7578181 | 7578298 | 1 | Pool1 |
| CHP2_TP53_5 | CCAGTTGCAAACCAGACCTCA | AGGCCTCTGATTCCTCACTGAT | GENOME_REGION | COSM44241 | hg19 | chr17 | 7578160 | 7578320 | 7578181 | 7578298 | 1 | Pool1 |
| CHP2_TP53_5 | CCAGTTGCAAACCAGACCTCA | AGGCCTCTGATTCCTCACTGAT | GENOME_REGION | COSM44249 | hg19 | chr17 | 7578160 | 7578320 | 7578181 | 7578298 | 1 | Pool1 |
| CHP2_TP53_5 | CCAGTTGCAAACCAGACCTCA | AGGCCTCTGATTCCTCACTGAT | GENOME_REGION | COSM44274 | hg19 | chr17 | 7578160 | 7578320 | 7578181 | 7578298 | 1 | Pool1 |
| CHP2_TP53_5 | CCAGTTGCAAACCAGACCTCA | AGGCCTCTGATTCCTCACTGAT | GENOME_REGION | COSM44317 | hg19 | chr17 | 7578160 | 7578320 | 7578181 | 7578298 | 1 | Pool1 |
| CHP2_TP53_5 | CCAGTTGCAAACCAGACCTCA | AGGCCTCTGATTCCTCACTGAT | GENOME_REGION | COSM44334 | hg19 | chr17 | 7578160 | 7578320 | 7578181 | 7578298 | 1 | Pool1 |
| CHP2_TP53_5 | CCAGTTGCAAACCAGACCTCA | AGGCCTCTGATTCCTCACTGAT | GENOME_REGION | COSM44349 | hg19 | chr17 | 7578160 | 7578320 | 7578181 | 7578298 | 1 | Pool1 |
| CHP2_TP53_5 | CCAGTTGCAAACCAGACCTCA | AGGCCTCTGATTCCTCACTGAT | GENOME_REGION | COSM44351 | hg19 | chr17 | 7578160 | 7578320 | 7578181 | 7578298 | 1 | Pool1 |
| CHP2_TP53_5 | CCAGTTGCAAACCAGACCTCA | AGGCCTCTGATTCCTCACTGAT | GENOME_REGION | COSM44358 | hg19 | chr17 | 7578160 | 7578320 | 7578181 | 7578298 | 1 | Pool1 |
| CHP2_TP53_5 | CCAGTTGCAAACCAGACCTCA | AGGCCTCTGATTCCTCACTGAT | GENOME_REGION | COSM44365 | hg19 | chr17 | 7578160 | 7578320 | 7578181 | 7578298 | 1 | Pool1 |
| CHP2_TP53_5 | CCAGTTGCAAACCAGACCTCA | AGGCCTCTGATTCCTCACTGAT | GENOME_REGION | COSM44371 | hg19 | chr17 | 7578160 | 7578320 | 7578181 | 7578298 | 1 | Pool1 |
| CHP2_TP53_5 | CCAGTTGCAAACCAGACCTCA | AGGCCTCTGATTCCTCACTGAT | GENOME_REGION | COSM44372 | hg19 | chr17 | 7578160 | 7578320 | 7578181 | 7578298 | 1 | Pool1 |
| CHP2_TP53_5 | CCAGTTGCAAACCAGACCTCA | AGGCCTCTGATTCCTCACTGAT | GENOME_REGION | COSM44375 | hg19 | chr17 | 7578160 | 7578320 | 7578181 | 7578298 | 1 | Pool1 |
| CHP2_TP53_5 | CCAGTTGCAAACCAGACCTCA | AGGCCTCTGATTCCTCACTGAT | GENOME_REGION | COSM44407 | hg19 | chr17 | 7578160 | 7578320 | 7578181 | 7578298 | 1 | Pool1 |
| CHP2_TP53_5 | CCAGTTGCAAACCAGACCTCA | AGGCCTCTGATTCCTCACTGAT | GENOME_REGION | COSM44411 | hg19 | chr17 | 7578160 | 7578320 | 7578181 | 7578298 | 1 | Pool1 |
| CHP2_TP53_5 | CCAGTTGCAAACCAGACCTCA | AGGCCTCTGATTCCTCACTGAT | GENOME_REGION | COSM44424 | hg19 | chr17 | 7578160 | 7578320 | 7578181 | 7578298 | 1 | Pool1 |
| CHP2_TP53_5 | CCAGTTGCAAACCAGACCTCA | AGGCCTCTGATTCCTCACTGAT | GENOME_REGION | COSM44426 | hg19 | chr17 | 7578160 | 7578320 | 7578181 | 7578298 | 1 | Pool1 |
| CHP2_TP53_5 | CCAGTTGCAAACCAGACCTCA | AGGCCTCTGATTCCTCACTGAT | GENOME_REGION | COSM44438 | hg19 | chr17 | 7578160 | 7578320 | 7578181 | 7578298 | 1 | Pool1 |
| CHP2_TP53_5 | CCAGTTGCAAACCAGACCTCA | AGGCCTCTGATTCCTCACTGAT | GENOME_REGION | COSM44439 | hg19 | chr17 | 7578160 | 7578320 | 7578181 | 7578298 | 1 | Pool1 |
| CHP2_TP53_5 | CCAGTTGCAAACCAGACCTCA | AGGCCTCTGATTCCTCACTGAT | GENOME_REGION | COSM44502 | hg19 | chr17 | 7578160 | 7578320 | 7578181 | 7578298 | 1 | Pool1 |
| CHP2_TP53_5 | CCAGTTGCAAACCAGACCTCA | AGGCCTCTGATTCCTCACTGAT | GENOME_REGION | COSM44505 | hg19 | chr17 | 7578160 | 7578320 | 7578181 | 7578298 | 1 | Pool1 |
| CHP2_TP53_5 | CCAGTTGCAAACCAGACCTCA | AGGCCTCTGATTCCTCACTGAT | GENOME_REGION | COSM44537 | hg19 | chr17 | 7578160 | 7578320 | 7578181 | 7578298 | 1 | Pool1 |
| CHP2_TP53_5 | CCAGTTGCAAACCAGACCTCA | AGGCCTCTGATTCCTCACTGAT | GENOME_REGION | COSM44539 | hg19 | chr17 | 7578160 | 7578320 | 7578181 | 7578298 | 1 | Pool1 |
| CHP2_TP53_5 | CCAGTTGCAAACCAGACCTCA | AGGCCTCTGATTCCTCACTGAT | GENOME_REGION | COSM44567 | hg19 | chr17 | 7578160 | 7578320 | 7578181 | 7578298 | 1 | Pool1 |
| CHP2_TP53_5 | CCAGTTGCAAACCAGACCTCA | AGGCCTCTGATTCCTCACTGAT | GENOME_REGION | COSM44569 | hg19 | chr17 | 7578160 | 7578320 | 7578181 | 7578298 | 1 | Pool1 |
| CHP2_TP53_5 | CCAGTTGCAAACCAGACCTCA | AGGCCTCTGATTCCTCACTGAT | GENOME_REGION | COSM44571 | hg19 | chr17 | 7578160 | 7578320 | 7578181 | 7578298 | 1 | Pool1 |
| CHP2_TP53_5 | CCAGTTGCAAACCAGACCTCA | AGGCCTCTGATTCCTCACTGAT | GENOME_REGION | COSM44585 | hg19 | chr17 | 7578160 | 7578320 | 7578181 | 7578298 | 1 | Pool1 |
| CHP2_TP53_5 | CCAGTTGCAAACCAGACCTCA | AGGCCTCTGATTCCTCACTGAT | GENOME_REGION | COSM44599 | hg19 | chr17 | 7578160 | 7578320 | 7578181 | 7578298 | 1 | Pool1 |
| CHP2_TP53_5 | CCAGTTGCAAACCAGACCTCA | AGGCCTCTGATTCCTCACTGAT | GENOME_REGION | COSM44607 | hg19 | chr17 | 7578160 | 7578320 | 7578181 | 7578298 | 1 | Pool1 |
| CHP2_TP53_5 | CCAGTTGCAAACCAGACCTCA | AGGCCTCTGATTCCTCACTGAT | GENOME_REGION | COSM44615 | hg19 | chr17 | 7578160 | 7578320 | 7578181 | 7578298 | 1 | Pool1 |
| CHP2_TP53_5 | CCAGTTGCAAACCAGACCTCA | AGGCCTCTGATTCCTCACTGAT | GENOME_REGION | COSM44633 | hg19 | chr17 | 7578160 | 7578320 | 7578181 | 7578298 | 1 | Pool1 |
| CHP2_TP53_5 | CCAGTTGCAAACCAGACCTCA | AGGCCTCTGATTCCTCACTGAT | GENOME_REGION | COSM44637 | hg19 | chr17 | 7578160 | 7578320 | 7578181 | 7578298 | 1 | Pool1 |
| CHP2_TP53_5 | CCAGTTGCAAACCAGACCTCA | AGGCCTCTGATTCCTCACTGAT | GENOME_REGION | COSM44638 | hg19 | chr17 | 7578160 | 7578320 | 7578181 | 7578298 | 1 | Pool1 |
| CHP2_TP53_5 | CCAGTTGCAAACCAGACCTCA | AGGCCTCTGATTCCTCACTGAT | GENOME_REGION | COSM44661 | hg19 | chr17 | 7578160 | 7578320 | 7578181 | 7578298 | 1 | Pool1 |
| CHP2_TP53_5 | CCAGTTGCAAACCAGACCTCA | AGGCCTCTGATTCCTCACTGAT | GENOME_REGION | COSM44665 | hg19 | chr17 | 7578160 | 7578320 | 7578181 | 7578298 | 1 | Pool1 |
| CHP2_TP53_5 | CCAGTTGCAAACCAGACCTCA | AGGCCTCTGATTCCTCACTGAT | GENOME_REGION | COSM44672 | hg19 | chr17 | 7578160 | 7578320 | 7578181 | 7578298 | 1 | Pool1 |
| CHP2_TP53_5 | CCAGTTGCAAACCAGACCTCA | AGGCCTCTGATTCCTCACTGAT | GENOME_REGION | COSM44682 | hg19 | chr17 | 7578160 | 7578320 | 7578181 | 7578298 | 1 | Pool1 |
| CHP2_TP53_5 | CCAGTTGCAAACCAGACCTCA | AGGCCTCTGATTCCTCACTGAT | GENOME_REGION | COSM44683 | hg19 | chr17 | 7578160 | 7578320 | 7578181 | 7578298 | 1 | Pool1 |
| CHP2_TP53_5 | CCAGTTGCAAACCAGACCTCA | AGGCCTCTGATTCCTCACTGAT | GENOME_REGION | COSM44689 | hg19 | chr17 | 7578160 | 7578320 | 7578181 | 7578298 | 1 | Pool1 |
| CHP2_TP53_5 | CCAGTTGCAAACCAGACCTCA | AGGCCTCTGATTCCTCACTGAT | GENOME_REGION | COSM44695 | hg19 | chr17 | 7578160 | 7578320 | 7578181 | 7578298 | 1 | Pool1 |
| CHP2_TP53_5 | CCAGTTGCAAACCAGACCTCA | AGGCCTCTGATTCCTCACTGAT | GENOME_REGION | COSM44707 | hg19 | chr17 | 7578160 | 7578320 | 7578181 | 7578298 | 1 | Pool1 |
| CHP2_TP53_5 | CCAGTTGCAAACCAGACCTCA | AGGCCTCTGATTCCTCACTGAT | GENOME_REGION | COSM44757 | hg19 | chr17 | 7578160 | 7578320 | 7578181 | 7578298 | 1 | Pool1 |
| CHP2_TP53_5 | CCAGTTGCAAACCAGACCTCA | AGGCCTCTGATTCCTCACTGAT | GENOME_REGION | COSM44817 | hg19 | chr17 | 7578160 | 7578320 | 7578181 | 7578298 | 1 | Pool1 |
| CHP2_TP53_5 | CCAGTTGCAAACCAGACCTCA | AGGCCTCTGATTCCTCACTGAT | GENOME_REGION | COSM44845 | hg19 | chr17 | 7578160 | 7578320 | 7578181 | 7578298 | 1 | Pool1 |
| CHP2_TP53_5 | CCAGTTGCAAACCAGACCTCA | AGGCCTCTGATTCCTCACTGAT | GENOME_REGION | COSM44846 | hg19 | chr17 | 7578160 | 7578320 | 7578181 | 7578298 | 1 | Pool1 |
| CHP2_TP53_5 | CCAGTTGCAAACCAGACCTCA | AGGCCTCTGATTCCTCACTGAT | GENOME_REGION | COSM44848 | hg19 | chr17 | 7578160 | 7578320 | 7578181 | 7578298 | 1 | Pool1 |
| CHP2_TP53_5 | CCAGTTGCAAACCAGACCTCA | AGGCCTCTGATTCCTCACTGAT | GENOME_REGION | COSM44849 | hg19 | chr17 | 7578160 | 7578320 | 7578181 | 7578298 | 1 | Pool1 |
| CHP2_TP53_5 | CCAGTTGCAAACCAGACCTCA | AGGCCTCTGATTCCTCACTGAT | GENOME_REGION | COSM44852 | hg19 | chr17 | 7578160 | 7578320 | 7578181 | 7578298 | 1 | Pool1 |
| CHP2_TP53_5 | CCAGTTGCAAACCAGACCTCA | AGGCCTCTGATTCCTCACTGAT | GENOME_REGION | COSM44853 | hg19 | chr17 | 7578160 | 7578320 | 7578181 | 7578298 | 1 | Pool1 |
| CHP2_TP53_5 | CCAGTTGCAAACCAGACCTCA | AGGCCTCTGATTCCTCACTGAT | GENOME_REGION | COSM44854 | hg19 | chr17 | 7578160 | 7578320 | 7578181 | 7578298 | 1 | Pool1 |
| CHP2_TP53_5 | CCAGTTGCAAACCAGACCTCA | AGGCCTCTGATTCCTCACTGAT | GENOME_REGION | COSM44877 | hg19 | chr17 | 7578160 | 7578320 | 7578181 | 7578298 | 1 | Pool1 |
| CHP2_TP53_5 | CCAGTTGCAAACCAGACCTCA | AGGCCTCTGATTCCTCACTGAT | GENOME_REGION | COSM44887 | hg19 | chr17 | 7578160 | 7578320 | 7578181 | 7578298 | 1 | Pool1 |
| CHP2_TP53_5 | CCAGTTGCAAACCAGACCTCA | AGGCCTCTGATTCCTCACTGAT | GENOME_REGION | COSM44923 | hg19 | chr17 | 7578160 | 7578320 | 7578181 | 7578298 | 1 | Pool1 |
| CHP2_TP53_5 | CCAGTTGCAAACCAGACCTCA | AGGCCTCTGATTCCTCACTGAT | GENOME_REGION | COSM44924 | hg19 | chr17 | 7578160 | 7578320 | 7578181 | 7578298 | 1 | Pool1 |
| CHP2_TP53_5 | CCAGTTGCAAACCAGACCTCA | AGGCCTCTGATTCCTCACTGAT | GENOME_REGION | COSM44925 | hg19 | chr17 | 7578160 | 7578320 | 7578181 | 7578298 | 1 | Pool1 |
| CHP2_TP53_5 | CCAGTTGCAAACCAGACCTCA | AGGCCTCTGATTCCTCACTGAT | GENOME_REGION | COSM44929 | hg19 | chr17 | 7578160 | 7578320 | 7578181 | 7578298 | 1 | Pool1 |
| CHP2_TP53_5 | CCAGTTGCAAACCAGACCTCA | AGGCCTCTGATTCCTCACTGAT | GENOME_REGION | COSM44930 | hg19 | chr17 | 7578160 | 7578320 | 7578181 | 7578298 | 1 | Pool1 |
| CHP2_TP53_5 | CCAGTTGCAAACCAGACCTCA | AGGCCTCTGATTCCTCACTGAT | GENOME_REGION | COSM44979 | hg19 | chr17 | 7578160 | 7578320 | 7578181 | 7578298 | 1 | Pool1 |
| CHP2_TP53_5 | CCAGTTGCAAACCAGACCTCA | AGGCCTCTGATTCCTCACTGAT | GENOME_REGION | COSM45021 | hg19 | chr17 | 7578160 | 7578320 | 7578181 | 7578298 | 1 | Pool1 |
| CHP2_TP53_5 | CCAGTTGCAAACCAGACCTCA | AGGCCTCTGATTCCTCACTGAT | GENOME_REGION | COSM45026 | hg19 | chr17 | 7578160 | 7578320 | 7578181 | 7578298 | 1 | Pool1 |
| CHP2_TP53_5 | CCAGTTGCAAACCAGACCTCA | AGGCCTCTGATTCCTCACTGAT | GENOME_REGION | COSM45029 | hg19 | chr17 | 7578160 | 7578320 | 7578181 | 7578298 | 1 | Pool1 |
| CHP2_TP53_5 | CCAGTTGCAAACCAGACCTCA | AGGCCTCTGATTCCTCACTGAT | GENOME_REGION | COSM45044 | hg19 | chr17 | 7578160 | 7578320 | 7578181 | 7578298 | 1 | Pool1 |
| CHP2_TP53_5 | CCAGTTGCAAACCAGACCTCA | AGGCCTCTGATTCCTCACTGAT | GENOME_REGION | COSM45051 | hg19 | chr17 | 7578160 | 7578320 | 7578181 | 7578298 | 1 | Pool1 |
| CHP2_TP53_5 | CCAGTTGCAAACCAGACCTCA | AGGCCTCTGATTCCTCACTGAT | GENOME_REGION | COSM45110 | hg19 | chr17 | 7578160 | 7578320 | 7578181 | 7578298 | 1 | Pool1 |
| CHP2_TP53_5 | CCAGTTGCAAACCAGACCTCA | AGGCCTCTGATTCCTCACTGAT | GENOME_REGION | COSM45115 | hg19 | chr17 | 7578160 | 7578320 | 7578181 | 7578298 | 1 | Pool1 |
| CHP2_TP53_5 | CCAGTTGCAAACCAGACCTCA | AGGCCTCTGATTCCTCACTGAT | GENOME_REGION | COSM45122 | hg19 | chr17 | 7578160 | 7578320 | 7578181 | 7578298 | 1 | Pool1 |
| CHP2_TP53_5 | CCAGTTGCAAACCAGACCTCA | AGGCCTCTGATTCCTCACTGAT | GENOME_REGION | COSM45140 | hg19 | chr17 | 7578160 | 7578320 | 7578181 | 7578298 | 1 | Pool1 |
| CHP2_TP53_5 | CCAGTTGCAAACCAGACCTCA | AGGCCTCTGATTCCTCACTGAT | GENOME_REGION | COSM45240 | hg19 | chr17 | 7578160 | 7578320 | 7578181 | 7578298 | 1 | Pool1 |
| CHP2_TP53_5 | CCAGTTGCAAACCAGACCTCA | AGGCCTCTGATTCCTCACTGAT | GENOME_REGION | COSM45253 | hg19 | chr17 | 7578160 | 7578320 | 7578181 | 7578298 | 1 | Pool1 |
| CHP2_TP53_5 | CCAGTTGCAAACCAGACCTCA | AGGCCTCTGATTCCTCACTGAT | GENOME_REGION | COSM45257 | hg19 | chr17 | 7578160 | 7578320 | 7578181 | 7578298 | 1 | Pool1 |
| CHP2_TP53_5 | CCAGTTGCAAACCAGACCTCA | AGGCCTCTGATTCCTCACTGAT | GENOME_REGION | COSM45308 | hg19 | chr17 | 7578160 | 7578320 | 7578181 | 7578298 | 1 | Pool1 |
| CHP2_TP53_5 | CCAGTTGCAAACCAGACCTCA | AGGCCTCTGATTCCTCACTGAT | GENOME_REGION | COSM45320 | hg19 | chr17 | 7578160 | 7578320 | 7578181 | 7578298 | 1 | Pool1 |
| CHP2_TP53_5 | CCAGTTGCAAACCAGACCTCA | AGGCCTCTGATTCCTCACTGAT | GENOME_REGION | COSM45341 | hg19 | chr17 | 7578160 | 7578320 | 7578181 | 7578298 | 1 | Pool1 |
| CHP2_TP53_5 | CCAGTTGCAAACCAGACCTCA | AGGCCTCTGATTCCTCACTGAT | GENOME_REGION | COSM45438 | hg19 | chr17 | 7578160 | 7578320 | 7578181 | 7578298 | 1 | Pool1 |
| CHP2_TP53_5 | CCAGTTGCAAACCAGACCTCA | AGGCCTCTGATTCCTCACTGAT | GENOME_REGION | COSM45440 | hg19 | chr17 | 7578160 | 7578320 | 7578181 | 7578298 | 1 | Pool1 |
| CHP2_TP53_5 | CCAGTTGCAAACCAGACCTCA | AGGCCTCTGATTCCTCACTGAT | GENOME_REGION | COSM45441 | hg19 | chr17 | 7578160 | 7578320 | 7578181 | 7578298 | 1 | Pool1 |
| CHP2_TP53_5 | CCAGTTGCAAACCAGACCTCA | AGGCCTCTGATTCCTCACTGAT | GENOME_REGION | COSM45449 | hg19 | chr17 | 7578160 | 7578320 | 7578181 | 7578298 | 1 | Pool1 |
| CHP2_TP53_5 | CCAGTTGCAAACCAGACCTCA | AGGCCTCTGATTCCTCACTGAT | GENOME_REGION | COSM45489 | hg19 | chr17 | 7578160 | 7578320 | 7578181 | 7578298 | 1 | Pool1 |
| CHP2_TP53_5 | CCAGTTGCAAACCAGACCTCA | AGGCCTCTGATTCCTCACTGAT | GENOME_REGION | COSM45511 | hg19 | chr17 | 7578160 | 7578320 | 7578181 | 7578298 | 1 | Pool1 |
| CHP2_TP53_5 | CCAGTTGCAAACCAGACCTCA | AGGCCTCTGATTCCTCACTGAT | GENOME_REGION | COSM45516 | hg19 | chr17 | 7578160 | 7578320 | 7578181 | 7578298 | 1 | Pool1 |
| CHP2_TP53_5 | CCAGTTGCAAACCAGACCTCA | AGGCCTCTGATTCCTCACTGAT | GENOME_REGION | COSM45519 | hg19 | chr17 | 7578160 | 7578320 | 7578181 | 7578298 | 1 | Pool1 |
| CHP2_TP53_5 | CCAGTTGCAAACCAGACCTCA | AGGCCTCTGATTCCTCACTGAT | GENOME_REGION | COSM45685 | hg19 | chr17 | 7578160 | 7578320 | 7578181 | 7578298 | 1 | Pool1 |
| CHP2_TP53_5 | CCAGTTGCAAACCAGACCTCA | AGGCCTCTGATTCCTCACTGAT | GENOME_REGION | COSM45703 | hg19 | chr17 | 7578160 | 7578320 | 7578181 | 7578298 | 1 | Pool1 |
| CHP2_TP53_5 | CCAGTTGCAAACCAGACCTCA | AGGCCTCTGATTCCTCACTGAT | GENOME_REGION | COSM45707 | hg19 | chr17 | 7578160 | 7578320 | 7578181 | 7578298 | 1 | Pool1 |
| CHP2_TP53_5 | CCAGTTGCAAACCAGACCTCA | AGGCCTCTGATTCCTCACTGAT | GENOME_REGION | COSM45777 | hg19 | chr17 | 7578160 | 7578320 | 7578181 | 7578298 | 1 | Pool1 |
| CHP2_TP53_5 | CCAGTTGCAAACCAGACCTCA | AGGCCTCTGATTCCTCACTGAT | GENOME_REGION | COSM45796 | hg19 | chr17 | 7578160 | 7578320 | 7578181 | 7578298 | 1 | Pool1 |
| CHP2_TP53_5 | CCAGTTGCAAACCAGACCTCA | AGGCCTCTGATTCCTCACTGAT | GENOME_REGION | COSM45851 | hg19 | chr17 | 7578160 | 7578320 | 7578181 | 7578298 | 1 | Pool1 |
| CHP2_TP53_5 | CCAGTTGCAAACCAGACCTCA | AGGCCTCTGATTCCTCACTGAT | GENOME_REGION | COSM45995 | hg19 | chr17 | 7578160 | 7578320 | 7578181 | 7578298 | 1 | Pool1 |
| CHP2_TP53_5 | CCAGTTGCAAACCAGACCTCA | AGGCCTCTGATTCCTCACTGAT | GENOME_REGION | COSM46000 | hg19 | chr17 | 7578160 | 7578320 | 7578181 | 7578298 | 1 | Pool1 |
| CHP2_TP53_5 | CCAGTTGCAAACCAGACCTCA | AGGCCTCTGATTCCTCACTGAT | GENOME_REGION | COSM46059 | hg19 | chr17 | 7578160 | 7578320 | 7578181 | 7578298 | 1 | Pool1 |
| CHP2_TP53_5 | CCAGTTGCAAACCAGACCTCA | AGGCCTCTGATTCCTCACTGAT | GENOME_REGION | COSM46074 | hg19 | chr17 | 7578160 | 7578320 | 7578181 | 7578298 | 1 | Pool1 |
| CHP2_TP53_5 | CCAGTTGCAAACCAGACCTCA | AGGCCTCTGATTCCTCACTGAT | GENOME_REGION | COSM46107 | hg19 | chr17 | 7578160 | 7578320 | 7578181 | 7578298 | 1 | Pool1 |
| CHP2_TP53_5 | CCAGTTGCAAACCAGACCTCA | AGGCCTCTGATTCCTCACTGAT | GENOME_REGION | COSM46211 | hg19 | chr17 | 7578160 | 7578320 | 7578181 | 7578298 | 1 | Pool1 |
| CHP2_TP53_5 | CCAGTTGCAAACCAGACCTCA | AGGCCTCTGATTCCTCACTGAT | GENOME_REGION | COSM46212 | hg19 | chr17 | 7578160 | 7578320 | 7578181 | 7578298 | 1 | Pool1 |
| CHP2_TP53_5 | CCAGTTGCAAACCAGACCTCA | AGGCCTCTGATTCCTCACTGAT | GENOME_REGION | COSM46214 | hg19 | chr17 | 7578160 | 7578320 | 7578181 | 7578298 | 1 | Pool1 |
| CHP2_TP53_5 | CCAGTTGCAAACCAGACCTCA | AGGCCTCTGATTCCTCACTGAT | GENOME_REGION | COSM6482 | hg19 | chr17 | 7578160 | 7578320 | 7578181 | 7578298 | 1 | Pool1 |
| CHP2_TP53_5 | CCAGTTGCAAACCAGACCTCA | AGGCCTCTGATTCCTCACTGAT | GENOME_REGION | COSM6496 | hg19 | chr17 | 7578160 | 7578320 | 7578181 | 7578298 | 1 | Pool1 |
| CHP2_TP53_5 | CCAGTTGCAAACCAGACCTCA | AGGCCTCTGATTCCTCACTGAT | GENOME_REGION | COSM96575 | hg19 | chr17 | 7578160 | 7578320 | 7578181 | 7578298 | 1 | Pool1 |
| CHP2_TP53_5 | CCAGTTGCAAACCAGACCTCA | AGGCCTCTGATTCCTCACTGAT | GENOME_REGION | COSM99615 | hg19 | chr17 | 7578160 | 7578320 | 7578181 | 7578298 | 1 | Pool1 |
| CHP2_TP53_5 | CCAGTTGCAAACCAGACCTCA | AGGCCTCTGATTCCTCACTGAT | GENOME_REGION | COSM99616 | hg19 | chr17 | 7578160 | 7578320 | 7578181 | 7578298 | 1 | Pool1 |
| CHP2_TP53_5 | CCAGTTGCAAACCAGACCTCA | AGGCCTCTGATTCCTCACTGAT | GENOME_REGION | COSM99617 | hg19 | chr17 | 7578160 | 7578320 | 7578181 | 7578298 | 1 | Pool1 |
| CHP2_TP53_5 | CCAGTTGCAAACCAGACCTCA | AGGCCTCTGATTCCTCACTGAT | GENOME_REGION | COSM99618 | hg19 | chr17 | 7578160 | 7578320 | 7578181 | 7578298 | 1 | Pool1 |
| CHP2_TP53_5 | CCAGTTGCAAACCAGACCTCA | AGGCCTCTGATTCCTCACTGAT | GENOME_REGION | COSM99665 | hg19 | chr17 | 7578160 | 7578320 | 7578181 | 7578298 | 1 | Pool1 |
| CHP2_TP53_5 | CCAGTTGCAAACCAGACCTCA | AGGCCTCTGATTCCTCACTGAT | GENOME_REGION | COSM99666 | hg19 | chr17 | 7578160 | 7578320 | 7578181 | 7578298 | 1 | Pool1 |
| CHP2_TP53_5 | CCAGTTGCAAACCAGACCTCA | AGGCCTCTGATTCCTCACTGAT | GENOME_REGION | COSM99667 | hg19 | chr17 | 7578160 | 7578320 | 7578181 | 7578298 | 1 | Pool1 |
| CHP2_TP53_5 | CCAGTTGCAAACCAGACCTCA | AGGCCTCTGATTCCTCACTGAT | GENOME_REGION | COSM99668 | hg19 | chr17 | 7578160 | 7578320 | 7578181 | 7578298 | 1 | Pool1 |
| CHP2_TP53_5 | CCAGTTGCAAACCAGACCTCA | AGGCCTCTGATTCCTCACTGAT | GENOME_REGION | COSM99718 | hg19 | chr17 | 7578160 | 7578320 | 7578181 | 7578298 | 1 | Pool1 |
| CHP2_TP53_5 | CCAGTTGCAAACCAGACCTCA | AGGCCTCTGATTCCTCACTGAT | GENOME_REGION | COSM99719 | hg19 | chr17 | 7578160 | 7578320 | 7578181 | 7578298 | 1 | Pool1 |
| CHP2_TP53_5 | CCAGTTGCAAACCAGACCTCA | AGGCCTCTGATTCCTCACTGAT | GENOME_REGION | COSM99720 | hg19 | chr17 | 7578160 | 7578320 | 7578181 | 7578298 | 1 | Pool1 |
| CHP2_TP53_5 | CCAGTTGCAAACCAGACCTCA | AGGCCTCTGATTCCTCACTGAT | GENOME_REGION | COSM99916 | hg19 | chr17 | 7578160 | 7578320 | 7578181 | 7578298 | 1 | Pool1 |
| CHP2_TP53_5 | CCAGTTGCAAACCAGACCTCA | AGGCCTCTGATTCCTCACTGAT | GENOME_REGION | COSM99917 | hg19 | chr17 | 7578160 | 7578320 | 7578181 | 7578298 | 1 | Pool1 |
| CHP2_TP53_5 | CCAGTTGCAAACCAGACCTCA | AGGCCTCTGATTCCTCACTGAT | GENOME_REGION | COSM99918 | hg19 | chr17 | 7578160 | 7578320 | 7578181 | 7578298 | 1 | Pool1 |
| CHP2_TP53_5 | CCAGTTGCAAACCAGACCTCA | AGGCCTCTGATTCCTCACTGAT | GENOME_REGION | COSM99919 | hg19 | chr17 | 7578160 | 7578320 | 7578181 | 7578298 | 1 | Pool1 |
| CHP2_TP53_6 | GGCTCCTGACCTGGAGTCTT | CTCATCTTGGGCCTGTGTTATCTC | GENOME_REGION | COSM10646 | hg19 | chr17 | 7577489 | 7577636 | 7577509 | 7577612 | 1 | Pool1 |
| CHP2_TP53_6 | GGCTCCTGACCTGGAGTCTT | CTCATCTTGGGCCTGTGTTATCTC | GENOME_REGION | COSM10656 | hg19 | chr17 | 7577489 | 7577636 | 7577509 | 7577612 | 1 | Pool1 |
| CHP2_TP53_6 | GGCTCCTGACCTGGAGTCTT | CTCATCTTGGGCCTGTGTTATCTC | GENOME_REGION | COSM10662 | hg19 | chr17 | 7577489 | 7577636 | 7577509 | 7577612 | 1 | Pool1 |
| CHP2_TP53_6 | GGCTCCTGACCTGGAGTCTT | CTCATCTTGGGCCTGTGTTATCTC | GENOME_REGION | COSM10668 | hg19 | chr17 | 7577489 | 7577636 | 7577509 | 7577612 | 1 | Pool1 |
| CHP2_TP53_6 | GGCTCCTGACCTGGAGTCTT | CTCATCTTGGGCCTGTGTTATCTC | GENOME_REGION | COSM10709 | hg19 | chr17 | 7577489 | 7577636 | 7577509 | 7577612 | 1 | Pool1 |
| CHP2_TP53_6 | GGCTCCTGACCTGGAGTCTT | CTCATCTTGGGCCTGTGTTATCTC | GENOME_REGION | COSM10715 | hg19 | chr17 | 7577489 | 7577636 | 7577509 | 7577612 | 1 | Pool1 |
| CHP2_TP53_6 | GGCTCCTGACCTGGAGTCTT | CTCATCTTGGGCCTGTGTTATCTC | GENOME_REGION | COSM10725 | hg19 | chr17 | 7577489 | 7577636 | 7577509 | 7577612 | 1 | Pool1 |
| CHP2_TP53_6 | GGCTCCTGACCTGGAGTCTT | CTCATCTTGGGCCTGTGTTATCTC | GENOME_REGION | COSM10731 | hg19 | chr17 | 7577489 | 7577636 | 7577509 | 7577612 | 1 | Pool1 |
| CHP2_TP53_6 | GGCTCCTGACCTGGAGTCTT | CTCATCTTGGGCCTGTGTTATCTC | GENOME_REGION | COSM10757 | hg19 | chr17 | 7577489 | 7577636 | 7577509 | 7577612 | 1 | Pool1 |
| CHP2_TP53_6 | GGCTCCTGACCTGGAGTCTT | CTCATCTTGGGCCTGTGTTATCTC | GENOME_REGION | COSM10771 | hg19 | chr17 | 7577489 | 7577636 | 7577509 | 7577612 | 1 | Pool1 |
| CHP2_TP53_6 | GGCTCCTGACCTGGAGTCTT | CTCATCTTGGGCCTGTGTTATCTC | GENOME_REGION | COSM10777 | hg19 | chr17 | 7577489 | 7577636 | 7577509 | 7577612 | 1 | Pool1 |
| CHP2_TP53_6 | GGCTCCTGACCTGGAGTCTT | CTCATCTTGGGCCTGTGTTATCTC | GENOME_REGION | COSM10785 | hg19 | chr17 | 7577489 | 7577636 | 7577509 | 7577612 | 1 | Pool1 |
| CHP2_TP53_6 | GGCTCCTGACCTGGAGTCTT | CTCATCTTGGGCCTGTGTTATCTC | GENOME_REGION | COSM10788 | hg19 | chr17 | 7577489 | 7577636 | 7577509 | 7577612 | 1 | Pool1 |
| CHP2_TP53_6 | GGCTCCTGACCTGGAGTCTT | CTCATCTTGGGCCTGTGTTATCTC | GENOME_REGION | COSM10810 | hg19 | chr17 | 7577489 | 7577636 | 7577509 | 7577612 | 1 | Pool1 |
| CHP2_TP53_6 | GGCTCCTGACCTGGAGTCTT | CTCATCTTGGGCCTGTGTTATCTC | GENOME_REGION | COSM10812 | hg19 | chr17 | 7577489 | 7577636 | 7577509 | 7577612 | 1 | Pool1 |
| CHP2_TP53_6 | GGCTCCTGACCTGGAGTCTT | CTCATCTTGGGCCTGTGTTATCTC | GENOME_REGION | COSM10817 | hg19 | chr17 | 7577489 | 7577636 | 7577509 | 7577612 | 1 | Pool1 |
| CHP2_TP53_6 | GGCTCCTGACCTGGAGTCTT | CTCATCTTGGGCCTGTGTTATCTC | GENOME_REGION | COSM10834 | hg19 | chr17 | 7577489 | 7577636 | 7577509 | 7577612 | 1 | Pool1 |
| CHP2_TP53_6 | GGCTCCTGACCTGGAGTCTT | CTCATCTTGGGCCTGTGTTATCTC | GENOME_REGION | COSM10883 | hg19 | chr17 | 7577489 | 7577636 | 7577509 | 7577612 | 1 | Pool1 |
| CHP2_TP53_6 | GGCTCCTGACCTGGAGTCTT | CTCATCTTGGGCCTGTGTTATCTC | GENOME_REGION | COSM10931 | hg19 | chr17 | 7577489 | 7577636 | 7577509 | 7577612 | 1 | Pool1 |
| CHP2_TP53_6 | GGCTCCTGACCTGGAGTCTT | CTCATCTTGGGCCTGTGTTATCTC | GENOME_REGION | COSM10935 | hg19 | chr17 | 7577489 | 7577636 | 7577509 | 7577612 | 1 | Pool1 |
| CHP2_TP53_6 | GGCTCCTGACCTGGAGTCTT | CTCATCTTGGGCCTGTGTTATCTC | GENOME_REGION | COSM10941 | hg19 | chr17 | 7577489 | 7577636 | 7577509 | 7577612 | 1 | Pool1 |
| CHP2_TP53_6 | GGCTCCTGACCTGGAGTCTT | CTCATCTTGGGCCTGTGTTATCTC | GENOME_REGION | COSM10957 | hg19 | chr17 | 7577489 | 7577636 | 7577509 | 7577612 | 1 | Pool1 |
| CHP2_TP53_6 | GGCTCCTGACCTGGAGTCTT | CTCATCTTGGGCCTGTGTTATCTC | GENOME_REGION | COSM11059 | hg19 | chr17 | 7577489 | 7577636 | 7577509 | 7577612 | 1 | Pool1 |
| CHP2_TP53_6 | GGCTCCTGACCTGGAGTCTT | CTCATCTTGGGCCTGTGTTATCTC | GENOME_REGION | COSM11063 | hg19 | chr17 | 7577489 | 7577636 | 7577509 | 7577612 | 1 | Pool1 |
| CHP2_TP53_6 | GGCTCCTGACCTGGAGTCTT | CTCATCTTGGGCCTGTGTTATCTC | GENOME_REGION | COSM11081 | hg19 | chr17 | 7577489 | 7577636 | 7577509 | 7577612 | 1 | Pool1 |
| CHP2_TP53_6 | GGCTCCTGACCTGGAGTCTT | CTCATCTTGGGCCTGTGTTATCTC | GENOME_REGION | COSM11133 | hg19 | chr17 | 7577489 | 7577636 | 7577509 | 7577612 | 1 | Pool1 |
| CHP2_TP53_6 | GGCTCCTGACCTGGAGTCTT | CTCATCTTGGGCCTGTGTTATCTC | GENOME_REGION | COSM11152 | hg19 | chr17 | 7577489 | 7577636 | 7577509 | 7577612 | 1 | Pool1 |
| CHP2_TP53_6 | GGCTCCTGACCTGGAGTCTT | CTCATCTTGGGCCTGTGTTATCTC | GENOME_REGION | COSM11181 | hg19 | chr17 | 7577489 | 7577636 | 7577509 | 7577612 | 1 | Pool1 |
| CHP2_TP53_6 | GGCTCCTGACCTGGAGTCTT | CTCATCTTGGGCCTGTGTTATCTC | GENOME_REGION | COSM11196 | hg19 | chr17 | 7577489 | 7577636 | 7577509 | 7577612 | 1 | Pool1 |
| CHP2_TP53_6 | GGCTCCTGACCTGGAGTCTT | CTCATCTTGGGCCTGTGTTATCTC | GENOME_REGION | COSM11213 | hg19 | chr17 | 7577489 | 7577636 | 7577509 | 7577612 | 1 | Pool1 |
| CHP2_TP53_6 | GGCTCCTGACCTGGAGTCTT | CTCATCTTGGGCCTGTGTTATCTC | GENOME_REGION | COSM11244 | hg19 | chr17 | 7577489 | 7577636 | 7577509 | 7577612 | 1 | Pool1 |
| CHP2_TP53_6 | GGCTCCTGACCTGGAGTCTT | CTCATCTTGGGCCTGTGTTATCTC | GENOME_REGION | COSM11355 | hg19 | chr17 | 7577489 | 7577636 | 7577509 | 7577612 | 1 | Pool1 |
| CHP2_TP53_6 | GGCTCCTGACCTGGAGTCTT | CTCATCTTGGGCCTGTGTTATCTC | GENOME_REGION | COSM11356 | hg19 | chr17 | 7577489 | 7577636 | 7577509 | 7577612 | 1 | Pool1 |
| CHP2_TP53_6 | GGCTCCTGACCTGGAGTCTT | CTCATCTTGGGCCTGTGTTATCTC | GENOME_REGION | COSM11374 | hg19 | chr17 | 7577489 | 7577636 | 7577509 | 7577612 | 1 | Pool1 |
| CHP2_TP53_6 | GGCTCCTGACCTGGAGTCTT | CTCATCTTGGGCCTGTGTTATCTC | GENOME_REGION | COSM11376 | hg19 | chr17 | 7577489 | 7577636 | 7577509 | 7577612 | 1 | Pool1 |
| CHP2_TP53_6 | GGCTCCTGACCTGGAGTCTT | CTCATCTTGGGCCTGTGTTATCTC | GENOME_REGION | COSM11491 | hg19 | chr17 | 7577489 | 7577636 | 7577509 | 7577612 | 1 | Pool1 |
| CHP2_TP53_6 | GGCTCCTGACCTGGAGTCTT | CTCATCTTGGGCCTGTGTTATCTC | GENOME_REGION | COSM11524 | hg19 | chr17 | 7577489 | 7577636 | 7577509 | 7577612 | 1 | Pool1 |
| CHP2_TP53_6 | GGCTCCTGACCTGGAGTCTT | CTCATCTTGGGCCTGTGTTATCTC | GENOME_REGION | COSM11542 | hg19 | chr17 | 7577489 | 7577636 | 7577509 | 7577612 | 1 | Pool1 |
| CHP2_TP53_6 | GGCTCCTGACCTGGAGTCTT | CTCATCTTGGGCCTGTGTTATCTC | GENOME_REGION | COSM11564 | hg19 | chr17 | 7577489 | 7577636 | 7577509 | 7577612 | 1 | Pool1 |
| CHP2_TP53_6 | GGCTCCTGACCTGGAGTCTT | CTCATCTTGGGCCTGTGTTATCTC | GENOME_REGION | COSM116672 | hg19 | chr17 | 7577489 | 7577636 | 7577509 | 7577612 | 1 | Pool1 |
| CHP2_TP53_6 | GGCTCCTGACCTGGAGTCTT | CTCATCTTGGGCCTGTGTTATCTC | GENOME_REGION | COSM116673 | hg19 | chr17 | 7577489 | 7577636 | 7577509 | 7577612 | 1 | Pool1 |
| CHP2_TP53_6 | GGCTCCTGACCTGGAGTCTT | CTCATCTTGGGCCTGTGTTATCTC | GENOME_REGION | COSM116674 | hg19 | chr17 | 7577489 | 7577636 | 7577509 | 7577612 | 1 | Pool1 |
| CHP2_TP53_6 | GGCTCCTGACCTGGAGTCTT | CTCATCTTGGGCCTGTGTTATCTC | GENOME_REGION | COSM11738 | hg19 | chr17 | 7577489 | 7577636 | 7577509 | 7577612 | 1 | Pool1 |
| CHP2_TP53_6 | GGCTCCTGACCTGGAGTCTT | CTCATCTTGGGCCTGTGTTATCTC | GENOME_REGION | COSM11929 | hg19 | chr17 | 7577489 | 7577636 | 7577509 | 7577612 | 1 | Pool1 |
| CHP2_TP53_6 | GGCTCCTGACCTGGAGTCTT | CTCATCTTGGGCCTGTGTTATCTC | GENOME_REGION | COSM120005 | hg19 | chr17 | 7577489 | 7577636 | 7577509 | 7577612 | 1 | Pool1 |
| CHP2_TP53_6 | GGCTCCTGACCTGGAGTCTT | CTCATCTTGGGCCTGTGTTATCTC | GENOME_REGION | COSM120006 | hg19 | chr17 | 7577489 | 7577636 | 7577509 | 7577612 | 1 | Pool1 |
| CHP2_TP53_6 | GGCTCCTGACCTGGAGTCTT | CTCATCTTGGGCCTGTGTTATCTC | GENOME_REGION | COSM120007 | hg19 | chr17 | 7577489 | 7577636 | 7577509 | 7577612 | 1 | Pool1 |
| CHP2_TP53_6 | GGCTCCTGACCTGGAGTCTT | CTCATCTTGGGCCTGTGTTATCTC | GENOME_REGION | COSM12013 | hg19 | chr17 | 7577489 | 7577636 | 7577509 | 7577612 | 1 | Pool1 |
| CHP2_TP53_6 | GGCTCCTGACCTGGAGTCTT | CTCATCTTGGGCCTGTGTTATCTC | GENOME_REGION | COSM121035 | hg19 | chr17 | 7577489 | 7577636 | 7577509 | 7577612 | 1 | Pool1 |
| CHP2_TP53_6 | GGCTCCTGACCTGGAGTCTT | CTCATCTTGGGCCTGTGTTATCTC | GENOME_REGION | COSM121036 | hg19 | chr17 | 7577489 | 7577636 | 7577509 | 7577612 | 1 | Pool1 |
| CHP2_TP53_6 | GGCTCCTGACCTGGAGTCTT | CTCATCTTGGGCCTGTGTTATCTC | GENOME_REGION | COSM121037 | hg19 | chr17 | 7577489 | 7577636 | 7577509 | 7577612 | 1 | Pool1 |
| CHP2_TP53_6 | GGCTCCTGACCTGGAGTCTT | CTCATCTTGGGCCTGTGTTATCTC | GENOME_REGION | COSM131478 | hg19 | chr17 | 7577489 | 7577636 | 7577509 | 7577612 | 1 | Pool1 |
| CHP2_TP53_6 | GGCTCCTGACCTGGAGTCTT | CTCATCTTGGGCCTGTGTTATCTC | GENOME_REGION | COSM131479 | hg19 | chr17 | 7577489 | 7577636 | 7577509 | 7577612 | 1 | Pool1 |
| CHP2_TP53_6 | GGCTCCTGACCTGGAGTCTT | CTCATCTTGGGCCTGTGTTATCTC | GENOME_REGION | COSM165072 | hg19 | chr17 | 7577489 | 7577636 | 7577509 | 7577612 | 1 | Pool1 |
| CHP2_TP53_6 | GGCTCCTGACCTGGAGTCTT | CTCATCTTGGGCCTGTGTTATCTC | GENOME_REGION | COSM165073 | hg19 | chr17 | 7577489 | 7577636 | 7577509 | 7577612 | 1 | Pool1 |
| CHP2_TP53_6 | GGCTCCTGACCTGGAGTCTT | CTCATCTTGGGCCTGTGTTATCTC | GENOME_REGION | COSM165074 | hg19 | chr17 | 7577489 | 7577636 | 7577509 | 7577612 | 1 | Pool1 |
| CHP2_TP53_6 | GGCTCCTGACCTGGAGTCTT | CTCATCTTGGGCCTGTGTTATCTC | GENOME_REGION | COSM179805 | hg19 | chr17 | 7577489 | 7577636 | 7577509 | 7577612 | 1 | Pool1 |
| CHP2_TP53_6 | GGCTCCTGACCTGGAGTCTT | CTCATCTTGGGCCTGTGTTATCTC | GENOME_REGION | COSM179806 | hg19 | chr17 | 7577489 | 7577636 | 7577509 | 7577612 | 1 | Pool1 |
| CHP2_TP53_6 | GGCTCCTGACCTGGAGTCTT | CTCATCTTGGGCCTGTGTTATCTC | GENOME_REGION | COSM179807 | hg19 | chr17 | 7577489 | 7577636 | 7577509 | 7577612 | 1 | Pool1 |
| CHP2_TP53_6 | GGCTCCTGACCTGGAGTCTT | CTCATCTTGGGCCTGTGTTATCTC | GENOME_REGION | COSM39293 | hg19 | chr17 | 7577489 | 7577636 | 7577509 | 7577612 | 1 | Pool1 |
| CHP2_TP53_6 | GGCTCCTGACCTGGAGTCTT | CTCATCTTGGGCCTGTGTTATCTC | GENOME_REGION | COSM43550 | hg19 | chr17 | 7577489 | 7577636 | 7577509 | 7577612 | 1 | Pool1 |
| CHP2_TP53_6 | GGCTCCTGACCTGGAGTCTT | CTCATCTTGGGCCTGTGTTATCTC | GENOME_REGION | COSM43555 | hg19 | chr17 | 7577489 | 7577636 | 7577509 | 7577612 | 1 | Pool1 |
| CHP2_TP53_6 | GGCTCCTGACCTGGAGTCTT | CTCATCTTGGGCCTGTGTTATCTC | GENOME_REGION | COSM43564 | hg19 | chr17 | 7577489 | 7577636 | 7577509 | 7577612 | 1 | Pool1 |
| CHP2_TP53_6 | GGCTCCTGACCTGGAGTCTT | CTCATCTTGGGCCTGTGTTATCTC | GENOME_REGION | COSM43588 | hg19 | chr17 | 7577489 | 7577636 | 7577509 | 7577612 | 1 | Pool1 |
| CHP2_TP53_6 | GGCTCCTGACCTGGAGTCTT | CTCATCTTGGGCCTGTGTTATCTC | GENOME_REGION | COSM43602 | hg19 | chr17 | 7577489 | 7577636 | 7577509 | 7577612 | 1 | Pool1 |
| CHP2_TP53_6 | GGCTCCTGACCTGGAGTCTT | CTCATCTTGGGCCTGTGTTATCTC | GENOME_REGION | COSM43606 | hg19 | chr17 | 7577489 | 7577636 | 7577509 | 7577612 | 1 | Pool1 |
| CHP2_TP53_6 | GGCTCCTGACCTGGAGTCTT | CTCATCTTGGGCCTGTGTTATCTC | GENOME_REGION | COSM43616 | hg19 | chr17 | 7577489 | 7577636 | 7577509 | 7577612 | 1 | Pool1 |
| CHP2_TP53_6 | GGCTCCTGACCTGGAGTCTT | CTCATCTTGGGCCTGTGTTATCTC | GENOME_REGION | COSM43629 | hg19 | chr17 | 7577489 | 7577636 | 7577509 | 7577612 | 1 | Pool1 |
| CHP2_TP53_6 | GGCTCCTGACCTGGAGTCTT | CTCATCTTGGGCCTGTGTTATCTC | GENOME_REGION | COSM43645 | hg19 | chr17 | 7577489 | 7577636 | 7577509 | 7577612 | 1 | Pool1 |
| CHP2_TP53_6 | GGCTCCTGACCTGGAGTCTT | CTCATCTTGGGCCTGTGTTATCTC | GENOME_REGION | COSM43648 | hg19 | chr17 | 7577489 | 7577636 | 7577509 | 7577612 | 1 | Pool1 |
| CHP2_TP53_6 | GGCTCCTGACCTGGAGTCTT | CTCATCTTGGGCCTGTGTTATCTC | GENOME_REGION | COSM43651 | hg19 | chr17 | 7577489 | 7577636 | 7577509 | 7577612 | 1 | Pool1 |
| CHP2_TP53_6 | GGCTCCTGACCTGGAGTCTT | CTCATCTTGGGCCTGTGTTATCTC | GENOME_REGION | COSM43652 | hg19 | chr17 | 7577489 | 7577636 | 7577509 | 7577612 | 1 | Pool1 |
| CHP2_TP53_6 | GGCTCCTGACCTGGAGTCTT | CTCATCTTGGGCCTGTGTTATCTC | GENOME_REGION | COSM43656 | hg19 | chr17 | 7577489 | 7577636 | 7577509 | 7577612 | 1 | Pool1 |
| CHP2_TP53_6 | GGCTCCTGACCTGGAGTCTT | CTCATCTTGGGCCTGTGTTATCTC | GENOME_REGION | COSM43660 | hg19 | chr17 | 7577489 | 7577636 | 7577509 | 7577612 | 1 | Pool1 |
| CHP2_TP53_6 | GGCTCCTGACCTGGAGTCTT | CTCATCTTGGGCCTGTGTTATCTC | GENOME_REGION | COSM43665 | hg19 | chr17 | 7577489 | 7577636 | 7577509 | 7577612 | 1 | Pool1 |
| CHP2_TP53_6 | GGCTCCTGACCTGGAGTCTT | CTCATCTTGGGCCTGTGTTATCTC | GENOME_REGION | COSM43683 | hg19 | chr17 | 7577489 | 7577636 | 7577509 | 7577612 | 1 | Pool1 |
| CHP2_TP53_6 | GGCTCCTGACCTGGAGTCTT | CTCATCTTGGGCCTGTGTTATCTC | GENOME_REGION | COSM43684 | hg19 | chr17 | 7577489 | 7577636 | 7577509 | 7577612 | 1 | Pool1 |
| CHP2_TP53_6 | GGCTCCTGACCTGGAGTCTT | CTCATCTTGGGCCTGTGTTATCTC | GENOME_REGION | COSM43695 | hg19 | chr17 | 7577489 | 7577636 | 7577509 | 7577612 | 1 | Pool1 |
| CHP2_TP53_6 | GGCTCCTGACCTGGAGTCTT | CTCATCTTGGGCCTGTGTTATCTC | GENOME_REGION | COSM43700 | hg19 | chr17 | 7577489 | 7577636 | 7577509 | 7577612 | 1 | Pool1 |
| CHP2_TP53_6 | GGCTCCTGACCTGGAGTCTT | CTCATCTTGGGCCTGTGTTATCTC | GENOME_REGION | COSM43726 | hg19 | chr17 | 7577489 | 7577636 | 7577509 | 7577612 | 1 | Pool1 |
| CHP2_TP53_6 | GGCTCCTGACCTGGAGTCTT | CTCATCTTGGGCCTGTGTTATCTC | GENOME_REGION | COSM43765 | hg19 | chr17 | 7577489 | 7577636 | 7577509 | 7577612 | 1 | Pool1 |
| CHP2_TP53_6 | GGCTCCTGACCTGGAGTCTT | CTCATCTTGGGCCTGTGTTATCTC | GENOME_REGION | COSM43768 | hg19 | chr17 | 7577489 | 7577636 | 7577509 | 7577612 | 1 | Pool1 |
| CHP2_TP53_6 | GGCTCCTGACCTGGAGTCTT | CTCATCTTGGGCCTGTGTTATCTC | GENOME_REGION | COSM43778 | hg19 | chr17 | 7577489 | 7577636 | 7577509 | 7577612 | 1 | Pool1 |
| CHP2_TP53_6 | GGCTCCTGACCTGGAGTCTT | CTCATCTTGGGCCTGTGTTATCTC | GENOME_REGION | COSM43801 | hg19 | chr17 | 7577489 | 7577636 | 7577509 | 7577612 | 1 | Pool1 |
| CHP2_TP53_6 | GGCTCCTGACCTGGAGTCTT | CTCATCTTGGGCCTGTGTTATCTC | GENOME_REGION | COSM43806 | hg19 | chr17 | 7577489 | 7577636 | 7577509 | 7577612 | 1 | Pool1 |
| CHP2_TP53_6 | GGCTCCTGACCTGGAGTCTT | CTCATCTTGGGCCTGTGTTATCTC | GENOME_REGION | COSM43826 | hg19 | chr17 | 7577489 | 7577636 | 7577509 | 7577612 | 1 | Pool1 |
| CHP2_TP53_6 | GGCTCCTGACCTGGAGTCTT | CTCATCTTGGGCCTGTGTTATCTC | GENOME_REGION | COSM43829 | hg19 | chr17 | 7577489 | 7577636 | 7577509 | 7577612 | 1 | Pool1 |
| CHP2_TP53_6 | GGCTCCTGACCTGGAGTCTT | CTCATCTTGGGCCTGTGTTATCTC | GENOME_REGION | COSM43853 | hg19 | chr17 | 7577489 | 7577636 | 7577509 | 7577612 | 1 | Pool1 |
| CHP2_TP53_6 | GGCTCCTGACCTGGAGTCTT | CTCATCTTGGGCCTGTGTTATCTC | GENOME_REGION | COSM43860 | hg19 | chr17 | 7577489 | 7577636 | 7577509 | 7577612 | 1 | Pool1 |
| CHP2_TP53_6 | GGCTCCTGACCTGGAGTCTT | CTCATCTTGGGCCTGTGTTATCTC | GENOME_REGION | COSM43862 | hg19 | chr17 | 7577489 | 7577636 | 7577509 | 7577612 | 1 | Pool1 |
| CHP2_TP53_6 | GGCTCCTGACCTGGAGTCTT | CTCATCTTGGGCCTGTGTTATCTC | GENOME_REGION | COSM43864 | hg19 | chr17 | 7577489 | 7577636 | 7577509 | 7577612 | 1 | Pool1 |
| CHP2_TP53_6 | GGCTCCTGACCTGGAGTCTT | CTCATCTTGGGCCTGTGTTATCTC | GENOME_REGION | COSM43865 | hg19 | chr17 | 7577489 | 7577636 | 7577509 | 7577612 | 1 | Pool1 |
| CHP2_TP53_6 | GGCTCCTGACCTGGAGTCTT | CTCATCTTGGGCCTGTGTTATCTC | GENOME_REGION | COSM43868 | hg19 | chr17 | 7577489 | 7577636 | 7577509 | 7577612 | 1 | Pool1 |
| CHP2_TP53_6 | GGCTCCTGACCTGGAGTCTT | CTCATCTTGGGCCTGTGTTATCTC | GENOME_REGION | COSM43871 | hg19 | chr17 | 7577489 | 7577636 | 7577509 | 7577612 | 1 | Pool1 |
| CHP2_TP53_6 | GGCTCCTGACCTGGAGTCTT | CTCATCTTGGGCCTGTGTTATCTC | GENOME_REGION | COSM43881 | hg19 | chr17 | 7577489 | 7577636 | 7577509 | 7577612 | 1 | Pool1 |
| CHP2_TP53_6 | GGCTCCTGACCTGGAGTCTT | CTCATCTTGGGCCTGTGTTATCTC | GENOME_REGION | COSM43889 | hg19 | chr17 | 7577489 | 7577636 | 7577509 | 7577612 | 1 | Pool1 |
| CHP2_TP53_6 | GGCTCCTGACCTGGAGTCTT | CTCATCTTGGGCCTGTGTTATCTC | GENOME_REGION | COSM43920 | hg19 | chr17 | 7577489 | 7577636 | 7577509 | 7577612 | 1 | Pool1 |
| CHP2_TP53_6 | GGCTCCTGACCTGGAGTCTT | CTCATCTTGGGCCTGTGTTATCTC | GENOME_REGION | COSM43952 | hg19 | chr17 | 7577489 | 7577636 | 7577509 | 7577612 | 1 | Pool1 |
| CHP2_TP53_6 | GGCTCCTGACCTGGAGTCTT | CTCATCTTGGGCCTGTGTTATCTC | GENOME_REGION | COSM43956 | hg19 | chr17 | 7577489 | 7577636 | 7577509 | 7577612 | 1 | Pool1 |
| CHP2_TP53_6 | GGCTCCTGACCTGGAGTCTT | CTCATCTTGGGCCTGTGTTATCTC | GENOME_REGION | COSM43957 | hg19 | chr17 | 7577489 | 7577636 | 7577509 | 7577612 | 1 | Pool1 |
| CHP2_TP53_6 | GGCTCCTGACCTGGAGTCTT | CTCATCTTGGGCCTGTGTTATCTC | GENOME_REGION | COSM43960 | hg19 | chr17 | 7577489 | 7577636 | 7577509 | 7577612 | 1 | Pool1 |
| CHP2_TP53_6 | GGCTCCTGACCTGGAGTCTT | CTCATCTTGGGCCTGTGTTATCTC | GENOME_REGION | COSM43965 | hg19 | chr17 | 7577489 | 7577636 | 7577509 | 7577612 | 1 | Pool1 |
| CHP2_TP53_6 | GGCTCCTGACCTGGAGTCTT | CTCATCTTGGGCCTGTGTTATCTC | GENOME_REGION | COSM43967 | hg19 | chr17 | 7577489 | 7577636 | 7577509 | 7577612 | 1 | Pool1 |
| CHP2_TP53_6 | GGCTCCTGACCTGGAGTCTT | CTCATCTTGGGCCTGTGTTATCTC | GENOME_REGION | COSM43973 | hg19 | chr17 | 7577489 | 7577636 | 7577509 | 7577612 | 1 | Pool1 |
| CHP2_TP53_6 | GGCTCCTGACCTGGAGTCTT | CTCATCTTGGGCCTGTGTTATCTC | GENOME_REGION | COSM43980 | hg19 | chr17 | 7577489 | 7577636 | 7577509 | 7577612 | 1 | Pool1 |
| CHP2_TP53_6 | GGCTCCTGACCTGGAGTCTT | CTCATCTTGGGCCTGTGTTATCTC | GENOME_REGION | COSM43995 | hg19 | chr17 | 7577489 | 7577636 | 7577509 | 7577612 | 1 | Pool1 |
| CHP2_TP53_6 | GGCTCCTGACCTGGAGTCTT | CTCATCTTGGGCCTGTGTTATCTC | GENOME_REGION | COSM44030 | hg19 | chr17 | 7577489 | 7577636 | 7577509 | 7577612 | 1 | Pool1 |
| CHP2_TP53_6 | GGCTCCTGACCTGGAGTCTT | CTCATCTTGGGCCTGTGTTATCTC | GENOME_REGION | COSM44054 | hg19 | chr17 | 7577489 | 7577636 | 7577509 | 7577612 | 1 | Pool1 |
| CHP2_TP53_6 | GGCTCCTGACCTGGAGTCTT | CTCATCTTGGGCCTGTGTTATCTC | GENOME_REGION | COSM44058 | hg19 | chr17 | 7577489 | 7577636 | 7577509 | 7577612 | 1 | Pool1 |
| CHP2_TP53_6 | GGCTCCTGACCTGGAGTCTT | CTCATCTTGGGCCTGTGTTATCTC | GENOME_REGION | COSM44064 | hg19 | chr17 | 7577489 | 7577636 | 7577509 | 7577612 | 1 | Pool1 |
| CHP2_TP53_6 | GGCTCCTGACCTGGAGTCTT | CTCATCTTGGGCCTGTGTTATCTC | GENOME_REGION | COSM44067 | hg19 | chr17 | 7577489 | 7577636 | 7577509 | 7577612 | 1 | Pool1 |
| CHP2_TP53_6 | GGCTCCTGACCTGGAGTCTT | CTCATCTTGGGCCTGTGTTATCTC | GENOME_REGION | COSM44072 | hg19 | chr17 | 7577489 | 7577636 | 7577509 | 7577612 | 1 | Pool1 |
| CHP2_TP53_6 | GGCTCCTGACCTGGAGTCTT | CTCATCTTGGGCCTGTGTTATCTC | GENOME_REGION | COSM44091 | hg19 | chr17 | 7577489 | 7577636 | 7577509 | 7577612 | 1 | Pool1 |
| CHP2_TP53_6 | GGCTCCTGACCTGGAGTCTT | CTCATCTTGGGCCTGTGTTATCTC | GENOME_REGION | COSM44094 | hg19 | chr17 | 7577489 | 7577636 | 7577509 | 7577612 | 1 | Pool1 |
| CHP2_TP53_6 | GGCTCCTGACCTGGAGTCTT | CTCATCTTGGGCCTGTGTTATCTC | GENOME_REGION | COSM44096 | hg19 | chr17 | 7577489 | 7577636 | 7577509 | 7577612 | 1 | Pool1 |
| CHP2_TP53_6 | GGCTCCTGACCTGGAGTCTT | CTCATCTTGGGCCTGTGTTATCTC | GENOME_REGION | COSM44103 | hg19 | chr17 | 7577489 | 7577636 | 7577509 | 7577612 | 1 | Pool1 |
| CHP2_TP53_6 | GGCTCCTGACCTGGAGTCTT | CTCATCTTGGGCCTGTGTTATCTC | GENOME_REGION | COSM44113 | hg19 | chr17 | 7577489 | 7577636 | 7577509 | 7577612 | 1 | Pool1 |
| CHP2_TP53_6 | GGCTCCTGACCTGGAGTCTT | CTCATCTTGGGCCTGTGTTATCTC | GENOME_REGION | COSM44124 | hg19 | chr17 | 7577489 | 7577636 | 7577509 | 7577612 | 1 | Pool1 |
| CHP2_TP53_6 | GGCTCCTGACCTGGAGTCTT | CTCATCTTGGGCCTGTGTTATCTC | GENOME_REGION | COSM44129 | hg19 | chr17 | 7577489 | 7577636 | 7577509 | 7577612 | 1 | Pool1 |
| CHP2_TP53_6 | GGCTCCTGACCTGGAGTCTT | CTCATCTTGGGCCTGTGTTATCTC | GENOME_REGION | COSM44132 | hg19 | chr17 | 7577489 | 7577636 | 7577509 | 7577612 | 1 | Pool1 |
| CHP2_TP53_6 | GGCTCCTGACCTGGAGTCTT | CTCATCTTGGGCCTGTGTTATCTC | GENOME_REGION | COSM44135 | hg19 | chr17 | 7577489 | 7577636 | 7577509 | 7577612 | 1 | Pool1 |
| CHP2_TP53_6 | GGCTCCTGACCTGGAGTCTT | CTCATCTTGGGCCTGTGTTATCTC | GENOME_REGION | COSM44183 | hg19 | chr17 | 7577489 | 7577636 | 7577509 | 7577612 | 1 | Pool1 |
| CHP2_TP53_6 | GGCTCCTGACCTGGAGTCTT | CTCATCTTGGGCCTGTGTTATCTC | GENOME_REGION | COSM44217 | hg19 | chr17 | 7577489 | 7577636 | 7577509 | 7577612 | 1 | Pool1 |
| CHP2_TP53_6 | GGCTCCTGACCTGGAGTCTT | CTCATCTTGGGCCTGTGTTATCTC | GENOME_REGION | COSM44221 | hg19 | chr17 | 7577489 | 7577636 | 7577509 | 7577612 | 1 | Pool1 |
| CHP2_TP53_6 | GGCTCCTGACCTGGAGTCTT | CTCATCTTGGGCCTGTGTTATCTC | GENOME_REGION | COSM44224 | hg19 | chr17 | 7577489 | 7577636 | 7577509 | 7577612 | 1 | Pool1 |
| CHP2_TP53_6 | GGCTCCTGACCTGGAGTCTT | CTCATCTTGGGCCTGTGTTATCTC | GENOME_REGION | COSM44247 | hg19 | chr17 | 7577489 | 7577636 | 7577509 | 7577612 | 1 | Pool1 |
| CHP2_TP53_6 | GGCTCCTGACCTGGAGTCTT | CTCATCTTGGGCCTGTGTTATCTC | GENOME_REGION | COSM44271 | hg19 | chr17 | 7577489 | 7577636 | 7577509 | 7577612 | 1 | Pool1 |
| CHP2_TP53_6 | GGCTCCTGACCTGGAGTCTT | CTCATCTTGGGCCTGTGTTATCTC | GENOME_REGION | COSM44290 | hg19 | chr17 | 7577489 | 7577636 | 7577509 | 7577612 | 1 | Pool1 |
| CHP2_TP53_6 | GGCTCCTGACCTGGAGTCTT | CTCATCTTGGGCCTGTGTTATCTC | GENOME_REGION | COSM44310 | hg19 | chr17 | 7577489 | 7577636 | 7577509 | 7577612 | 1 | Pool1 |
| CHP2_TP53_6 | GGCTCCTGACCTGGAGTCTT | CTCATCTTGGGCCTGTGTTATCTC | GENOME_REGION | COSM44313 | hg19 | chr17 | 7577489 | 7577636 | 7577509 | 7577612 | 1 | Pool1 |
| CHP2_TP53_6 | GGCTCCTGACCTGGAGTCTT | CTCATCTTGGGCCTGTGTTATCTC | GENOME_REGION | COSM44321 | hg19 | chr17 | 7577489 | 7577636 | 7577509 | 7577612 | 1 | Pool1 |
| CHP2_TP53_6 | GGCTCCTGACCTGGAGTCTT | CTCATCTTGGGCCTGTGTTATCTC | GENOME_REGION | COSM44322 | hg19 | chr17 | 7577489 | 7577636 | 7577509 | 7577612 | 1 | Pool1 |
| CHP2_TP53_6 | GGCTCCTGACCTGGAGTCTT | CTCATCTTGGGCCTGTGTTATCTC | GENOME_REGION | COSM44326 | hg19 | chr17 | 7577489 | 7577636 | 7577509 | 7577612 | 1 | Pool1 |
| CHP2_TP53_6 | GGCTCCTGACCTGGAGTCTT | CTCATCTTGGGCCTGTGTTATCTC | GENOME_REGION | COSM44350 | hg19 | chr17 | 7577489 | 7577636 | 7577509 | 7577612 | 1 | Pool1 |
| CHP2_TP53_6 | GGCTCCTGACCTGGAGTCTT | CTCATCTTGGGCCTGTGTTATCTC | GENOME_REGION | COSM44360 | hg19 | chr17 | 7577489 | 7577636 | 7577509 | 7577612 | 1 | Pool1 |
| CHP2_TP53_6 | GGCTCCTGACCTGGAGTCTT | CTCATCTTGGGCCTGTGTTATCTC | GENOME_REGION | COSM44378 | hg19 | chr17 | 7577489 | 7577636 | 7577509 | 7577612 | 1 | Pool1 |
| CHP2_TP53_6 | GGCTCCTGACCTGGAGTCTT | CTCATCTTGGGCCTGTGTTATCTC | GENOME_REGION | COSM44398 | hg19 | chr17 | 7577489 | 7577636 | 7577509 | 7577612 | 1 | Pool1 |
| CHP2_TP53_6 | GGCTCCTGACCTGGAGTCTT | CTCATCTTGGGCCTGTGTTATCTC | GENOME_REGION | COSM44399 | hg19 | chr17 | 7577489 | 7577636 | 7577509 | 7577612 | 1 | Pool1 |
| CHP2_TP53_6 | GGCTCCTGACCTGGAGTCTT | CTCATCTTGGGCCTGTGTTATCTC | GENOME_REGION | COSM44415 | hg19 | chr17 | 7577489 | 7577636 | 7577509 | 7577612 | 1 | Pool1 |
| CHP2_TP53_6 | GGCTCCTGACCTGGAGTCTT | CTCATCTTGGGCCTGTGTTATCTC | GENOME_REGION | COSM44428 | hg19 | chr17 | 7577489 | 7577636 | 7577509 | 7577612 | 1 | Pool1 |
| CHP2_TP53_6 | GGCTCCTGACCTGGAGTCTT | CTCATCTTGGGCCTGTGTTATCTC | GENOME_REGION | COSM44457 | hg19 | chr17 | 7577489 | 7577636 | 7577509 | 7577612 | 1 | Pool1 |
| CHP2_TP53_6 | GGCTCCTGACCTGGAGTCTT | CTCATCTTGGGCCTGTGTTATCTC | GENOME_REGION | COSM44458 | hg19 | chr17 | 7577489 | 7577636 | 7577509 | 7577612 | 1 | Pool1 |
| CHP2_TP53_6 | GGCTCCTGACCTGGAGTCTT | CTCATCTTGGGCCTGTGTTATCTC | GENOME_REGION | COSM44460 | hg19 | chr17 | 7577489 | 7577636 | 7577509 | 7577612 | 1 | Pool1 |
| CHP2_TP53_6 | GGCTCCTGACCTGGAGTCTT | CTCATCTTGGGCCTGTGTTATCTC | GENOME_REGION | COSM44464 | hg19 | chr17 | 7577489 | 7577636 | 7577509 | 7577612 | 1 | Pool1 |
| CHP2_TP53_6 | GGCTCCTGACCTGGAGTCTT | CTCATCTTGGGCCTGTGTTATCTC | GENOME_REGION | COSM44476 | hg19 | chr17 | 7577489 | 7577636 | 7577509 | 7577612 | 1 | Pool1 |
| CHP2_TP53_6 | GGCTCCTGACCTGGAGTCTT | CTCATCTTGGGCCTGTGTTATCTC | GENOME_REGION | COSM44510 | hg19 | chr17 | 7577489 | 7577636 | 7577509 | 7577612 | 1 | Pool1 |
| CHP2_TP53_6 | GGCTCCTGACCTGGAGTCTT | CTCATCTTGGGCCTGTGTTATCTC | GENOME_REGION | COSM44511 | hg19 | chr17 | 7577489 | 7577636 | 7577509 | 7577612 | 1 | Pool1 |
| CHP2_TP53_6 | GGCTCCTGACCTGGAGTCTT | CTCATCTTGGGCCTGTGTTATCTC | GENOME_REGION | COSM44512 | hg19 | chr17 | 7577489 | 7577636 | 7577509 | 7577612 | 1 | Pool1 |
| CHP2_TP53_6 | GGCTCCTGACCTGGAGTCTT | CTCATCTTGGGCCTGTGTTATCTC | GENOME_REGION | COSM44513 | hg19 | chr17 | 7577489 | 7577636 | 7577509 | 7577612 | 1 | Pool1 |
| CHP2_TP53_6 | GGCTCCTGACCTGGAGTCTT | CTCATCTTGGGCCTGTGTTATCTC | GENOME_REGION | COSM44514 | hg19 | chr17 | 7577489 | 7577636 | 7577509 | 7577612 | 1 | Pool1 |
| CHP2_TP53_6 | GGCTCCTGACCTGGAGTCTT | CTCATCTTGGGCCTGTGTTATCTC | GENOME_REGION | COSM44525 | hg19 | chr17 | 7577489 | 7577636 | 7577509 | 7577612 | 1 | Pool1 |
| CHP2_TP53_6 | GGCTCCTGACCTGGAGTCTT | CTCATCTTGGGCCTGTGTTATCTC | GENOME_REGION | COSM44535 | hg19 | chr17 | 7577489 | 7577636 | 7577509 | 7577612 | 1 | Pool1 |
| CHP2_TP53_6 | GGCTCCTGACCTGGAGTCTT | CTCATCTTGGGCCTGTGTTATCTC | GENOME_REGION | COSM44536 | hg19 | chr17 | 7577489 | 7577636 | 7577509 | 7577612 | 1 | Pool1 |
| CHP2_TP53_6 | GGCTCCTGACCTGGAGTCTT | CTCATCTTGGGCCTGTGTTATCTC | GENOME_REGION | COSM44541 | hg19 | chr17 | 7577489 | 7577636 | 7577509 | 7577612 | 1 | Pool1 |
| CHP2_TP53_6 | GGCTCCTGACCTGGAGTCTT | CTCATCTTGGGCCTGTGTTATCTC | GENOME_REGION | COSM44544 | hg19 | chr17 | 7577489 | 7577636 | 7577509 | 7577612 | 1 | Pool1 |
| CHP2_TP53_6 | GGCTCCTGACCTGGAGTCTT | CTCATCTTGGGCCTGTGTTATCTC | GENOME_REGION | COSM44547 | hg19 | chr17 | 7577489 | 7577636 | 7577509 | 7577612 | 1 | Pool1 |
| CHP2_TP53_6 | GGCTCCTGACCTGGAGTCTT | CTCATCTTGGGCCTGTGTTATCTC | GENOME_REGION | COSM44578 | hg19 | chr17 | 7577489 | 7577636 | 7577509 | 7577612 | 1 | Pool1 |
| CHP2_TP53_6 | GGCTCCTGACCTGGAGTCTT | CTCATCTTGGGCCTGTGTTATCTC | GENOME_REGION | COSM44601 | hg19 | chr17 | 7577489 | 7577636 | 7577509 | 7577612 | 1 | Pool1 |
| CHP2_TP53_6 | GGCTCCTGACCTGGAGTCTT | CTCATCTTGGGCCTGTGTTATCTC | GENOME_REGION | COSM44609 | hg19 | chr17 | 7577489 | 7577636 | 7577509 | 7577612 | 1 | Pool1 |
| CHP2_TP53_6 | GGCTCCTGACCTGGAGTCTT | CTCATCTTGGGCCTGTGTTATCTC | GENOME_REGION | COSM44621 | hg19 | chr17 | 7577489 | 7577636 | 7577509 | 7577612 | 1 | Pool1 |
| CHP2_TP53_6 | GGCTCCTGACCTGGAGTCTT | CTCATCTTGGGCCTGTGTTATCTC | GENOME_REGION | COSM44622 | hg19 | chr17 | 7577489 | 7577636 | 7577509 | 7577612 | 1 | Pool1 |
| CHP2_TP53_6 | GGCTCCTGACCTGGAGTCTT | CTCATCTTGGGCCTGTGTTATCTC | GENOME_REGION | COSM44625 | hg19 | chr17 | 7577489 | 7577636 | 7577509 | 7577612 | 1 | Pool1 |
| CHP2_TP53_6 | GGCTCCTGACCTGGAGTCTT | CTCATCTTGGGCCTGTGTTATCTC | GENOME_REGION | COSM44642 | hg19 | chr17 | 7577489 | 7577636 | 7577509 | 7577612 | 1 | Pool1 |
| CHP2_TP53_6 | GGCTCCTGACCTGGAGTCTT | CTCATCTTGGGCCTGTGTTATCTC | GENOME_REGION | COSM44647 | hg19 | chr17 | 7577489 | 7577636 | 7577509 | 7577612 | 1 | Pool1 |
| CHP2_TP53_6 | GGCTCCTGACCTGGAGTCTT | CTCATCTTGGGCCTGTGTTATCTC | GENOME_REGION | COSM44650 | hg19 | chr17 | 7577489 | 7577636 | 7577509 | 7577612 | 1 | Pool1 |
| CHP2_TP53_6 | GGCTCCTGACCTGGAGTCTT | CTCATCTTGGGCCTGTGTTATCTC | GENOME_REGION | COSM44653 | hg19 | chr17 | 7577489 | 7577636 | 7577509 | 7577612 | 1 | Pool1 |
| CHP2_TP53_6 | GGCTCCTGACCTGGAGTCTT | CTCATCTTGGGCCTGTGTTATCTC | GENOME_REGION | COSM44657 | hg19 | chr17 | 7577489 | 7577636 | 7577509 | 7577612 | 1 | Pool1 |
| CHP2_TP53_6 | GGCTCCTGACCTGGAGTCTT | CTCATCTTGGGCCTGTGTTATCTC | GENOME_REGION | COSM44662 | hg19 | chr17 | 7577489 | 7577636 | 7577509 | 7577612 | 1 | Pool1 |
| CHP2_TP53_6 | GGCTCCTGACCTGGAGTCTT | CTCATCTTGGGCCTGTGTTATCTC | GENOME_REGION | COSM44664 | hg19 | chr17 | 7577489 | 7577636 | 7577509 | 7577612 | 1 | Pool1 |
| CHP2_TP53_6 | GGCTCCTGACCTGGAGTCTT | CTCATCTTGGGCCTGTGTTATCTC | GENOME_REGION | COSM44676 | hg19 | chr17 | 7577489 | 7577636 | 7577509 | 7577612 | 1 | Pool1 |
| CHP2_TP53_6 | GGCTCCTGACCTGGAGTCTT | CTCATCTTGGGCCTGTGTTATCTC | GENOME_REGION | COSM44693 | hg19 | chr17 | 7577489 | 7577636 | 7577509 | 7577612 | 1 | Pool1 |
| CHP2_TP53_6 | GGCTCCTGACCTGGAGTCTT | CTCATCTTGGGCCTGTGTTATCTC | GENOME_REGION | COSM44705 | hg19 | chr17 | 7577489 | 7577636 | 7577509 | 7577612 | 1 | Pool1 |
| CHP2_TP53_6 | GGCTCCTGACCTGGAGTCTT | CTCATCTTGGGCCTGTGTTATCTC | GENOME_REGION | COSM44769 | hg19 | chr17 | 7577489 | 7577636 | 7577509 | 7577612 | 1 | Pool1 |
| CHP2_TP53_6 | GGCTCCTGACCTGGAGTCTT | CTCATCTTGGGCCTGTGTTATCTC | GENOME_REGION | COSM44784 | hg19 | chr17 | 7577489 | 7577636 | 7577509 | 7577612 | 1 | Pool1 |
| CHP2_TP53_6 | GGCTCCTGACCTGGAGTCTT | CTCATCTTGGGCCTGTGTTATCTC | GENOME_REGION | COSM44787 | hg19 | chr17 | 7577489 | 7577636 | 7577509 | 7577612 | 1 | Pool1 |
| CHP2_TP53_6 | GGCTCCTGACCTGGAGTCTT | CTCATCTTGGGCCTGTGTTATCTC | GENOME_REGION | COSM44820 | hg19 | chr17 | 7577489 | 7577636 | 7577509 | 7577612 | 1 | Pool1 |
| CHP2_TP53_6 | GGCTCCTGACCTGGAGTCTT | CTCATCTTGGGCCTGTGTTATCTC | GENOME_REGION | COSM44838 | hg19 | chr17 | 7577489 | 7577636 | 7577509 | 7577612 | 1 | Pool1 |
| CHP2_TP53_6 | GGCTCCTGACCTGGAGTCTT | CTCATCTTGGGCCTGTGTTATCTC | GENOME_REGION | COSM44843 | hg19 | chr17 | 7577489 | 7577636 | 7577509 | 7577612 | 1 | Pool1 |
| CHP2_TP53_6 | GGCTCCTGACCTGGAGTCTT | CTCATCTTGGGCCTGTGTTATCTC | GENOME_REGION | COSM44844 | hg19 | chr17 | 7577489 | 7577636 | 7577509 | 7577612 | 1 | Pool1 |
| CHP2_TP53_6 | GGCTCCTGACCTGGAGTCTT | CTCATCTTGGGCCTGTGTTATCTC | GENOME_REGION | COSM44900 | hg19 | chr17 | 7577489 | 7577636 | 7577509 | 7577612 | 1 | Pool1 |
| CHP2_TP53_6 | GGCTCCTGACCTGGAGTCTT | CTCATCTTGGGCCTGTGTTATCTC | GENOME_REGION | COSM44903 | hg19 | chr17 | 7577489 | 7577636 | 7577509 | 7577612 | 1 | Pool1 |
| CHP2_TP53_6 | GGCTCCTGACCTGGAGTCTT | CTCATCTTGGGCCTGTGTTATCTC | GENOME_REGION | COSM44908 | hg19 | chr17 | 7577489 | 7577636 | 7577509 | 7577612 | 1 | Pool1 |
| CHP2_TP53_6 | GGCTCCTGACCTGGAGTCTT | CTCATCTTGGGCCTGTGTTATCTC | GENOME_REGION | COSM44916 | hg19 | chr17 | 7577489 | 7577636 | 7577509 | 7577612 | 1 | Pool1 |
| CHP2_TP53_6 | GGCTCCTGACCTGGAGTCTT | CTCATCTTGGGCCTGTGTTATCTC | GENOME_REGION | COSM44920 | hg19 | chr17 | 7577489 | 7577636 | 7577509 | 7577612 | 1 | Pool1 |
| CHP2_TP53_6 | GGCTCCTGACCTGGAGTCTT | CTCATCTTGGGCCTGTGTTATCTC | GENOME_REGION | COSM44921 | hg19 | chr17 | 7577489 | 7577636 | 7577509 | 7577612 | 1 | Pool1 |
| CHP2_TP53_6 | GGCTCCTGACCTGGAGTCTT | CTCATCTTGGGCCTGTGTTATCTC | GENOME_REGION | COSM44935 | hg19 | chr17 | 7577489 | 7577636 | 7577509 | 7577612 | 1 | Pool1 |
| CHP2_TP53_6 | GGCTCCTGACCTGGAGTCTT | CTCATCTTGGGCCTGTGTTATCTC | GENOME_REGION | COSM44940 | hg19 | chr17 | 7577489 | 7577636 | 7577509 | 7577612 | 1 | Pool1 |
| CHP2_TP53_6 | GGCTCCTGACCTGGAGTCTT | CTCATCTTGGGCCTGTGTTATCTC | GENOME_REGION | COSM44953 | hg19 | chr17 | 7577489 | 7577636 | 7577509 | 7577612 | 1 | Pool1 |
| CHP2_TP53_6 | GGCTCCTGACCTGGAGTCTT | CTCATCTTGGGCCTGTGTTATCTC | GENOME_REGION | COSM44960 | hg19 | chr17 | 7577489 | 7577636 | 7577509 | 7577612 | 1 | Pool1 |
| CHP2_TP53_6 | GGCTCCTGACCTGGAGTCTT | CTCATCTTGGGCCTGTGTTATCTC | GENOME_REGION | COSM44964 | hg19 | chr17 | 7577489 | 7577636 | 7577509 | 7577612 | 1 | Pool1 |
| CHP2_TP53_6 | GGCTCCTGACCTGGAGTCTT | CTCATCTTGGGCCTGTGTTATCTC | GENOME_REGION | COSM44965 | hg19 | chr17 | 7577489 | 7577636 | 7577509 | 7577612 | 1 | Pool1 |
| CHP2_TP53_6 | GGCTCCTGACCTGGAGTCTT | CTCATCTTGGGCCTGTGTTATCTC | GENOME_REGION | COSM45005 | hg19 | chr17 | 7577489 | 7577636 | 7577509 | 7577612 | 1 | Pool1 |
| CHP2_TP53_6 | GGCTCCTGACCTGGAGTCTT | CTCATCTTGGGCCTGTGTTATCTC | GENOME_REGION | COSM45017 | hg19 | chr17 | 7577489 | 7577636 | 7577509 | 7577612 | 1 | Pool1 |
| CHP2_TP53_6 | GGCTCCTGACCTGGAGTCTT | CTCATCTTGGGCCTGTGTTATCTC | GENOME_REGION | COSM45028 | hg19 | chr17 | 7577489 | 7577636 | 7577509 | 7577612 | 1 | Pool1 |
| CHP2_TP53_6 | GGCTCCTGACCTGGAGTCTT | CTCATCTTGGGCCTGTGTTATCTC | GENOME_REGION | COSM45032 | hg19 | chr17 | 7577489 | 7577636 | 7577509 | 7577612 | 1 | Pool1 |
| CHP2_TP53_6 | GGCTCCTGACCTGGAGTCTT | CTCATCTTGGGCCTGTGTTATCTC | GENOME_REGION | COSM45034 | hg19 | chr17 | 7577489 | 7577636 | 7577509 | 7577612 | 1 | Pool1 |
| CHP2_TP53_6 | GGCTCCTGACCTGGAGTCTT | CTCATCTTGGGCCTGTGTTATCTC | GENOME_REGION | COSM45035 | hg19 | chr17 | 7577489 | 7577636 | 7577509 | 7577612 | 1 | Pool1 |
| CHP2_TP53_6 | GGCTCCTGACCTGGAGTCTT | CTCATCTTGGGCCTGTGTTATCTC | GENOME_REGION | COSM45045 | hg19 | chr17 | 7577489 | 7577636 | 7577509 | 7577612 | 1 | Pool1 |
| CHP2_TP53_6 | GGCTCCTGACCTGGAGTCTT | CTCATCTTGGGCCTGTGTTATCTC | GENOME_REGION | COSM45055 | hg19 | chr17 | 7577489 | 7577636 | 7577509 | 7577612 | 1 | Pool1 |
| CHP2_TP53_6 | GGCTCCTGACCTGGAGTCTT | CTCATCTTGGGCCTGTGTTATCTC | GENOME_REGION | COSM45084 | hg19 | chr17 | 7577489 | 7577636 | 7577509 | 7577612 | 1 | Pool1 |
| CHP2_TP53_6 | GGCTCCTGACCTGGAGTCTT | CTCATCTTGGGCCTGTGTTATCTC | GENOME_REGION | COSM45091 | hg19 | chr17 | 7577489 | 7577636 | 7577509 | 7577612 | 1 | Pool1 |
| CHP2_TP53_6 | GGCTCCTGACCTGGAGTCTT | CTCATCTTGGGCCTGTGTTATCTC | GENOME_REGION | COSM45114 | hg19 | chr17 | 7577489 | 7577636 | 7577509 | 7577612 | 1 | Pool1 |
| CHP2_TP53_6 | GGCTCCTGACCTGGAGTCTT | CTCATCTTGGGCCTGTGTTATCTC | GENOME_REGION | COSM45116 | hg19 | chr17 | 7577489 | 7577636 | 7577509 | 7577612 | 1 | Pool1 |
| CHP2_TP53_6 | GGCTCCTGACCTGGAGTCTT | CTCATCTTGGGCCTGTGTTATCTC | GENOME_REGION | COSM45134 | hg19 | chr17 | 7577489 | 7577636 | 7577509 | 7577612 | 1 | Pool1 |
| CHP2_TP53_6 | GGCTCCTGACCTGGAGTCTT | CTCATCTTGGGCCTGTGTTATCTC | GENOME_REGION | COSM45157 | hg19 | chr17 | 7577489 | 7577636 | 7577509 | 7577612 | 1 | Pool1 |
| CHP2_TP53_6 | GGCTCCTGACCTGGAGTCTT | CTCATCTTGGGCCTGTGTTATCTC | GENOME_REGION | COSM45162 | hg19 | chr17 | 7577489 | 7577636 | 7577509 | 7577612 | 1 | Pool1 |
| CHP2_TP53_6 | GGCTCCTGACCTGGAGTCTT | CTCATCTTGGGCCTGTGTTATCTC | GENOME_REGION | COSM45168 | hg19 | chr17 | 7577489 | 7577636 | 7577509 | 7577612 | 1 | Pool1 |
| CHP2_TP53_6 | GGCTCCTGACCTGGAGTCTT | CTCATCTTGGGCCTGTGTTATCTC | GENOME_REGION | COSM45172 | hg19 | chr17 | 7577489 | 7577636 | 7577509 | 7577612 | 1 | Pool1 |
| CHP2_TP53_6 | GGCTCCTGACCTGGAGTCTT | CTCATCTTGGGCCTGTGTTATCTC | GENOME_REGION | COSM45261 | hg19 | chr17 | 7577489 | 7577636 | 7577509 | 7577612 | 1 | Pool1 |
| CHP2_TP53_6 | GGCTCCTGACCTGGAGTCTT | CTCATCTTGGGCCTGTGTTATCTC | GENOME_REGION | COSM45322 | hg19 | chr17 | 7577489 | 7577636 | 7577509 | 7577612 | 1 | Pool1 |
| CHP2_TP53_6 | GGCTCCTGACCTGGAGTCTT | CTCATCTTGGGCCTGTGTTATCTC | GENOME_REGION | COSM45329 | hg19 | chr17 | 7577489 | 7577636 | 7577509 | 7577612 | 1 | Pool1 |
| CHP2_TP53_6 | GGCTCCTGACCTGGAGTCTT | CTCATCTTGGGCCTGTGTTATCTC | GENOME_REGION | COSM45394 | hg19 | chr17 | 7577489 | 7577636 | 7577509 | 7577612 | 1 | Pool1 |
| CHP2_TP53_6 | GGCTCCTGACCTGGAGTCTT | CTCATCTTGGGCCTGTGTTATCTC | GENOME_REGION | COSM45407 | hg19 | chr17 | 7577489 | 7577636 | 7577509 | 7577612 | 1 | Pool1 |
| CHP2_TP53_6 | GGCTCCTGACCTGGAGTCTT | CTCATCTTGGGCCTGTGTTATCTC | GENOME_REGION | COSM45410 | hg19 | chr17 | 7577489 | 7577636 | 7577509 | 7577612 | 1 | Pool1 |
| CHP2_TP53_6 | GGCTCCTGACCTGGAGTCTT | CTCATCTTGGGCCTGTGTTATCTC | GENOME_REGION | COSM45529 | hg19 | chr17 | 7577489 | 7577636 | 7577509 | 7577612 | 1 | Pool1 |
| CHP2_TP53_6 | GGCTCCTGACCTGGAGTCTT | CTCATCTTGGGCCTGTGTTATCTC | GENOME_REGION | COSM45543 | hg19 | chr17 | 7577489 | 7577636 | 7577509 | 7577612 | 1 | Pool1 |
| CHP2_TP53_6 | GGCTCCTGACCTGGAGTCTT | CTCATCTTGGGCCTGTGTTATCTC | GENOME_REGION | COSM45548 | hg19 | chr17 | 7577489 | 7577636 | 7577509 | 7577612 | 1 | Pool1 |
| CHP2_TP53_6 | GGCTCCTGACCTGGAGTCTT | CTCATCTTGGGCCTGTGTTATCTC | GENOME_REGION | COSM45607 | hg19 | chr17 | 7577489 | 7577636 | 7577509 | 7577612 | 1 | Pool1 |
| CHP2_TP53_6 | GGCTCCTGACCTGGAGTCTT | CTCATCTTGGGCCTGTGTTATCTC | GENOME_REGION | COSM45612 | hg19 | chr17 | 7577489 | 7577636 | 7577509 | 7577612 | 1 | Pool1 |
| CHP2_TP53_6 | GGCTCCTGACCTGGAGTCTT | CTCATCTTGGGCCTGTGTTATCTC | GENOME_REGION | COSM45620 | hg19 | chr17 | 7577489 | 7577636 | 7577509 | 7577612 | 1 | Pool1 |
| CHP2_TP53_6 | GGCTCCTGACCTGGAGTCTT | CTCATCTTGGGCCTGTGTTATCTC | GENOME_REGION | COSM45631 | hg19 | chr17 | 7577489 | 7577636 | 7577509 | 7577612 | 1 | Pool1 |
| CHP2_TP53_6 | GGCTCCTGACCTGGAGTCTT | CTCATCTTGGGCCTGTGTTATCTC | GENOME_REGION | COSM45632 | hg19 | chr17 | 7577489 | 7577636 | 7577509 | 7577612 | 1 | Pool1 |
| CHP2_TP53_6 | GGCTCCTGACCTGGAGTCTT | CTCATCTTGGGCCTGTGTTATCTC | GENOME_REGION | COSM45647 | hg19 | chr17 | 7577489 | 7577636 | 7577509 | 7577612 | 1 | Pool1 |
| CHP2_TP53_6 | GGCTCCTGACCTGGAGTCTT | CTCATCTTGGGCCTGTGTTATCTC | GENOME_REGION | COSM45654 | hg19 | chr17 | 7577489 | 7577636 | 7577509 | 7577612 | 1 | Pool1 |
| CHP2_TP53_6 | GGCTCCTGACCTGGAGTCTT | CTCATCTTGGGCCTGTGTTATCTC | GENOME_REGION | COSM45677 | hg19 | chr17 | 7577489 | 7577636 | 7577509 | 7577612 | 1 | Pool1 |
| CHP2_TP53_6 | GGCTCCTGACCTGGAGTCTT | CTCATCTTGGGCCTGTGTTATCTC | GENOME_REGION | COSM45691 | hg19 | chr17 | 7577489 | 7577636 | 7577509 | 7577612 | 1 | Pool1 |
| CHP2_TP53_6 | GGCTCCTGACCTGGAGTCTT | CTCATCTTGGGCCTGTGTTATCTC | GENOME_REGION | COSM45735 | hg19 | chr17 | 7577489 | 7577636 | 7577509 | 7577612 | 1 | Pool1 |
| CHP2_TP53_6 | GGCTCCTGACCTGGAGTCTT | CTCATCTTGGGCCTGTGTTATCTC | GENOME_REGION | COSM45739 | hg19 | chr17 | 7577489 | 7577636 | 7577509 | 7577612 | 1 | Pool1 |
| CHP2_TP53_6 | GGCTCCTGACCTGGAGTCTT | CTCATCTTGGGCCTGTGTTATCTC | GENOME_REGION | COSM45784 | hg19 | chr17 | 7577489 | 7577636 | 7577509 | 7577612 | 1 | Pool1 |
| CHP2_TP53_6 | GGCTCCTGACCTGGAGTCTT | CTCATCTTGGGCCTGTGTTATCTC | GENOME_REGION | COSM45786 | hg19 | chr17 | 7577489 | 7577636 | 7577509 | 7577612 | 1 | Pool1 |
| CHP2_TP53_6 | GGCTCCTGACCTGGAGTCTT | CTCATCTTGGGCCTGTGTTATCTC | GENOME_REGION | COSM45862 | hg19 | chr17 | 7577489 | 7577636 | 7577509 | 7577612 | 1 | Pool1 |
| CHP2_TP53_6 | GGCTCCTGACCTGGAGTCTT | CTCATCTTGGGCCTGTGTTATCTC | GENOME_REGION | COSM45868 | hg19 | chr17 | 7577489 | 7577636 | 7577509 | 7577612 | 1 | Pool1 |
| CHP2_TP53_6 | GGCTCCTGACCTGGAGTCTT | CTCATCTTGGGCCTGTGTTATCTC | GENOME_REGION | COSM45870 | hg19 | chr17 | 7577489 | 7577636 | 7577509 | 7577612 | 1 | Pool1 |
| CHP2_TP53_6 | GGCTCCTGACCTGGAGTCTT | CTCATCTTGGGCCTGTGTTATCTC | GENOME_REGION | COSM45959 | hg19 | chr17 | 7577489 | 7577636 | 7577509 | 7577612 | 1 | Pool1 |
| CHP2_TP53_6 | GGCTCCTGACCTGGAGTCTT | CTCATCTTGGGCCTGTGTTATCTC | GENOME_REGION | COSM45980 | hg19 | chr17 | 7577489 | 7577636 | 7577509 | 7577612 | 1 | Pool1 |
| CHP2_TP53_6 | GGCTCCTGACCTGGAGTCTT | CTCATCTTGGGCCTGTGTTATCTC | GENOME_REGION | COSM45992 | hg19 | chr17 | 7577489 | 7577636 | 7577509 | 7577612 | 1 | Pool1 |
| CHP2_TP53_6 | GGCTCCTGACCTGGAGTCTT | CTCATCTTGGGCCTGTGTTATCTC | GENOME_REGION | COSM46031 | hg19 | chr17 | 7577489 | 7577636 | 7577509 | 7577612 | 1 | Pool1 |
| CHP2_TP53_6 | GGCTCCTGACCTGGAGTCTT | CTCATCTTGGGCCTGTGTTATCTC | GENOME_REGION | COSM46136 | hg19 | chr17 | 7577489 | 7577636 | 7577509 | 7577612 | 1 | Pool1 |
| CHP2_TP53_6 | GGCTCCTGACCTGGAGTCTT | CTCATCTTGGGCCTGTGTTATCTC | GENOME_REGION | COSM46228 | hg19 | chr17 | 7577489 | 7577636 | 7577509 | 7577612 | 1 | Pool1 |
| CHP2_TP53_6 | GGCTCCTGACCTGGAGTCTT | CTCATCTTGGGCCTGTGTTATCTC | GENOME_REGION | COSM46336 | hg19 | chr17 | 7577489 | 7577636 | 7577509 | 7577612 | 1 | Pool1 |
| CHP2_TP53_6 | GGCTCCTGACCTGGAGTCTT | CTCATCTTGGGCCTGTGTTATCTC | GENOME_REGION | COSM6530 | hg19 | chr17 | 7577489 | 7577636 | 7577509 | 7577612 | 1 | Pool1 |
| CHP2_TP53_6 | GGCTCCTGACCTGGAGTCTT | CTCATCTTGGGCCTGTGTTATCTC | GENOME_REGION | COSM6545 | hg19 | chr17 | 7577489 | 7577636 | 7577509 | 7577612 | 1 | Pool1 |
| CHP2_TP53_6 | GGCTCCTGACCTGGAGTCTT | CTCATCTTGGGCCTGTGTTATCTC | GENOME_REGION | COSM6546 | hg19 | chr17 | 7577489 | 7577636 | 7577509 | 7577612 | 1 | Pool1 |
| CHP2_TP53_6 | GGCTCCTGACCTGGAGTCTT | CTCATCTTGGGCCTGTGTTATCTC | GENOME_REGION | COSM6549 | hg19 | chr17 | 7577489 | 7577636 | 7577509 | 7577612 | 1 | Pool1 |
| CHP2_TP53_6 | GGCTCCTGACCTGGAGTCTT | CTCATCTTGGGCCTGTGTTATCTC | GENOME_REGION | COSM69195 | hg19 | chr17 | 7577489 | 7577636 | 7577509 | 7577612 | 1 | Pool1 |
| CHP2_TP53_6 | GGCTCCTGACCTGGAGTCTT | CTCATCTTGGGCCTGTGTTATCTC | GENOME_REGION | COSM6932 | hg19 | chr17 | 7577489 | 7577636 | 7577509 | 7577612 | 1 | Pool1 |
| CHP2_TP53_6 | GGCTCCTGACCTGGAGTCTT | CTCATCTTGGGCCTGTGTTATCTC | GENOME_REGION | COSM99020 | hg19 | chr17 | 7577489 | 7577636 | 7577509 | 7577612 | 1 | Pool1 |
| CHP2_TP53_6 | GGCTCCTGACCTGGAGTCTT | CTCATCTTGGGCCTGTGTTATCTC | GENOME_REGION | COSM99021 | hg19 | chr17 | 7577489 | 7577636 | 7577509 | 7577612 | 1 | Pool1 |
| CHP2_TP53_6 | GGCTCCTGACCTGGAGTCTT | CTCATCTTGGGCCTGTGTTATCTC | GENOME_REGION | COSM99602 | hg19 | chr17 | 7577489 | 7577636 | 7577509 | 7577612 | 1 | Pool1 |
| CHP2_TP53_6 | GGCTCCTGACCTGGAGTCTT | CTCATCTTGGGCCTGTGTTATCTC | GENOME_REGION | COSM99624 | hg19 | chr17 | 7577489 | 7577636 | 7577509 | 7577612 | 1 | Pool1 |
| CHP2_TP53_6 | GGCTCCTGACCTGGAGTCTT | CTCATCTTGGGCCTGTGTTATCTC | GENOME_REGION | COSM99625 | hg19 | chr17 | 7577489 | 7577636 | 7577509 | 7577612 | 1 | Pool1 |
| CHP2_TP53_6 | GGCTCCTGACCTGGAGTCTT | CTCATCTTGGGCCTGTGTTATCTC | GENOME_REGION | COSM99626 | hg19 | chr17 | 7577489 | 7577636 | 7577509 | 7577612 | 1 | Pool1 |
| CHP2_TP53_6 | GGCTCCTGACCTGGAGTCTT | CTCATCTTGGGCCTGTGTTATCTC | GENOME_REGION | COSM99646 | hg19 | chr17 | 7577489 | 7577636 | 7577509 | 7577612 | 1 | Pool1 |
| CHP2_TP53_6 | GGCTCCTGACCTGGAGTCTT | CTCATCTTGGGCCTGTGTTATCTC | GENOME_REGION | COSM99647 | hg19 | chr17 | 7577489 | 7577636 | 7577509 | 7577612 | 1 | Pool1 |
| CHP2_TP53_6 | GGCTCCTGACCTGGAGTCTT | CTCATCTTGGGCCTGTGTTATCTC | GENOME_REGION | COSM99648 | hg19 | chr17 | 7577489 | 7577636 | 7577509 | 7577612 | 1 | Pool1 |
| CHP2_TP53_7 | CGCTTCTTGTCCTGCTTGCT | TTCTCTTTTCCTATCCTGAGTAGTGGT | GENOME_REGION | COSM10659 | hg19 | chr17 | 7576996 | 7577178 | 7577016 | 7577151 | 1 | Pool1 |
| CHP2_TP53_7 | CGCTTCTTGTCCTGCTTGCT | TTCTCTTTTCCTATCCTGAGTAGTGGT | GENOME_REGION | COSM10660 | hg19 | chr17 | 7576996 | 7577178 | 7577016 | 7577151 | 1 | Pool1 |
| CHP2_TP53_7 | CGCTTCTTGTCCTGCTTGCT | TTCTCTTTTCCTATCCTGAGTAGTGGT | GENOME_REGION | COSM10663 | hg19 | chr17 | 7576996 | 7577178 | 7577016 | 7577151 | 1 | Pool1 |
| CHP2_TP53_7 | CGCTTCTTGTCCTGCTTGCT | TTCTCTTTTCCTATCCTGAGTAGTGGT | GENOME_REGION | COSM10701 | hg19 | chr17 | 7576996 | 7577178 | 7577016 | 7577151 | 1 | Pool1 |
| CHP2_TP53_7 | CGCTTCTTGTCCTGCTTGCT | TTCTCTTTTCCTATCCTGAGTAGTGGT | GENOME_REGION | COSM10704 | hg19 | chr17 | 7576996 | 7577178 | 7577016 | 7577151 | 1 | Pool1 |
| CHP2_TP53_7 | CGCTTCTTGTCCTGCTTGCT | TTCTCTTTTCCTATCCTGAGTAGTGGT | GENOME_REGION | COSM10710 | hg19 | chr17 | 7576996 | 7577178 | 7577016 | 7577151 | 1 | Pool1 |
| CHP2_TP53_7 | CGCTTCTTGTCCTGCTTGCT | TTCTCTTTTCCTATCCTGAGTAGTGGT | GENOME_REGION | COSM10719 | hg19 | chr17 | 7576996 | 7577178 | 7577016 | 7577151 | 1 | Pool1 |
| CHP2_TP53_7 | CGCTTCTTGTCCTGCTTGCT | TTCTCTTTTCCTATCCTGAGTAGTGGT | GENOME_REGION | COSM10722 | hg19 | chr17 | 7576996 | 7577178 | 7577016 | 7577151 | 1 | Pool1 |
| CHP2_TP53_7 | CGCTTCTTGTCCTGCTTGCT | TTCTCTTTTCCTATCCTGAGTAGTGGT | GENOME_REGION | COSM10724 | hg19 | chr17 | 7576996 | 7577178 | 7577016 | 7577151 | 1 | Pool1 |
| CHP2_TP53_7 | CGCTTCTTGTCCTGCTTGCT | TTCTCTTTTCCTATCCTGAGTAGTGGT | GENOME_REGION | COSM10726 | hg19 | chr17 | 7576996 | 7577178 | 7577016 | 7577151 | 1 | Pool1 |
| CHP2_TP53_7 | CGCTTCTTGTCCTGCTTGCT | TTCTCTTTTCCTATCCTGAGTAGTGGT | GENOME_REGION | COSM10728 | hg19 | chr17 | 7576996 | 7577178 | 7577016 | 7577151 | 1 | Pool1 |
| CHP2_TP53_7 | CGCTTCTTGTCCTGCTTGCT | TTCTCTTTTCCTATCCTGAGTAGTGGT | GENOME_REGION | COSM10743 | hg19 | chr17 | 7576996 | 7577178 | 7577016 | 7577151 | 1 | Pool1 |
| CHP2_TP53_7 | CGCTTCTTGTCCTGCTTGCT | TTCTCTTTTCCTATCCTGAGTAGTGGT | GENOME_REGION | COSM10749 | hg19 | chr17 | 7576996 | 7577178 | 7577016 | 7577151 | 1 | Pool1 |
| CHP2_TP53_7 | CGCTTCTTGTCCTGCTTGCT | TTCTCTTTTCCTATCCTGAGTAGTGGT | GENOME_REGION | COSM10756 | hg19 | chr17 | 7576996 | 7577178 | 7577016 | 7577151 | 1 | Pool1 |
| CHP2_TP53_7 | CGCTTCTTGTCCTGCTTGCT | TTCTCTTTTCCTATCCTGAGTAGTGGT | GENOME_REGION | COSM10769 | hg19 | chr17 | 7576996 | 7577178 | 7577016 | 7577151 | 1 | Pool1 |
| CHP2_TP53_7 | CGCTTCTTGTCCTGCTTGCT | TTCTCTTTTCCTATCCTGAGTAGTGGT | GENOME_REGION | COSM10779 | hg19 | chr17 | 7576996 | 7577178 | 7577016 | 7577151 | 1 | Pool1 |
| CHP2_TP53_7 | CGCTTCTTGTCCTGCTTGCT | TTCTCTTTTCCTATCCTGAGTAGTGGT | GENOME_REGION | COSM10794 | hg19 | chr17 | 7576996 | 7577178 | 7577016 | 7577151 | 1 | Pool1 |
| CHP2_TP53_7 | CGCTTCTTGTCCTGCTTGCT | TTCTCTTTTCCTATCCTGAGTAGTGGT | GENOME_REGION | COSM10814 | hg19 | chr17 | 7576996 | 7577178 | 7577016 | 7577151 | 1 | Pool1 |
| CHP2_TP53_7 | CGCTTCTTGTCCTGCTTGCT | TTCTCTTTTCCTATCCTGAGTAGTGGT | GENOME_REGION | COSM10856 | hg19 | chr17 | 7576996 | 7577178 | 7577016 | 7577151 | 1 | Pool1 |
| CHP2_TP53_7 | CGCTTCTTGTCCTGCTTGCT | TTCTCTTTTCCTATCCTGAGTAGTGGT | GENOME_REGION | COSM10859 | hg19 | chr17 | 7576996 | 7577178 | 7577016 | 7577151 | 1 | Pool1 |
| CHP2_TP53_7 | CGCTTCTTGTCCTGCTTGCT | TTCTCTTTTCCTATCCTGAGTAGTGGT | GENOME_REGION | COSM10863 | hg19 | chr17 | 7576996 | 7577178 | 7577016 | 7577151 | 1 | Pool1 |
| CHP2_TP53_7 | CGCTTCTTGTCCTGCTTGCT | TTCTCTTTTCCTATCCTGAGTAGTGGT | GENOME_REGION | COSM10867 | hg19 | chr17 | 7576996 | 7577178 | 7577016 | 7577151 | 1 | Pool1 |
| CHP2_TP53_7 | CGCTTCTTGTCCTGCTTGCT | TTCTCTTTTCCTATCCTGAGTAGTGGT | GENOME_REGION | COSM10887 | hg19 | chr17 | 7576996 | 7577178 | 7577016 | 7577151 | 1 | Pool1 |
| CHP2_TP53_7 | CGCTTCTTGTCCTGCTTGCT | TTCTCTTTTCCTATCCTGAGTAGTGGT | GENOME_REGION | COSM10891 | hg19 | chr17 | 7576996 | 7577178 | 7577016 | 7577151 | 1 | Pool1 |
| CHP2_TP53_7 | CGCTTCTTGTCCTGCTTGCT | TTCTCTTTTCCTATCCTGAGTAGTGGT | GENOME_REGION | COSM10893 | hg19 | chr17 | 7576996 | 7577178 | 7577016 | 7577151 | 1 | Pool1 |
| CHP2_TP53_7 | CGCTTCTTGTCCTGCTTGCT | TTCTCTTTTCCTATCCTGAGTAGTGGT | GENOME_REGION | COSM10911 | hg19 | chr17 | 7576996 | 7577178 | 7577016 | 7577151 | 1 | Pool1 |
| CHP2_TP53_7 | CGCTTCTTGTCCTGCTTGCT | TTCTCTTTTCCTATCCTGAGTAGTGGT | GENOME_REGION | COSM10939 | hg19 | chr17 | 7576996 | 7577178 | 7577016 | 7577151 | 1 | Pool1 |
| CHP2_TP53_7 | CGCTTCTTGTCCTGCTTGCT | TTCTCTTTTCCTATCCTGAGTAGTGGT | GENOME_REGION | COSM10943 | hg19 | chr17 | 7576996 | 7577178 | 7577016 | 7577151 | 1 | Pool1 |
| CHP2_TP53_7 | CGCTTCTTGTCCTGCTTGCT | TTCTCTTTTCCTATCCTGAGTAGTGGT | GENOME_REGION | COSM10958 | hg19 | chr17 | 7576996 | 7577178 | 7577016 | 7577151 | 1 | Pool1 |
| CHP2_TP53_7 | CGCTTCTTGTCCTGCTTGCT | TTCTCTTTTCCTATCCTGAGTAGTGGT | GENOME_REGION | COSM10992 | hg19 | chr17 | 7576996 | 7577178 | 7577016 | 7577151 | 1 | Pool1 |
| CHP2_TP53_7 | CGCTTCTTGTCCTGCTTGCT | TTCTCTTTTCCTATCCTGAGTAGTGGT | GENOME_REGION | COSM11011 | hg19 | chr17 | 7576996 | 7577178 | 7577016 | 7577151 | 1 | Pool1 |
| CHP2_TP53_7 | CGCTTCTTGTCCTGCTTGCT | TTCTCTTTTCCTATCCTGAGTAGTGGT | GENOME_REGION | COSM11123 | hg19 | chr17 | 7576996 | 7577178 | 7577016 | 7577151 | 1 | Pool1 |
| CHP2_TP53_7 | CGCTTCTTGTCCTGCTTGCT | TTCTCTTTTCCTATCCTGAGTAGTGGT | GENOME_REGION | COSM11183 | hg19 | chr17 | 7576996 | 7577178 | 7577016 | 7577151 | 1 | Pool1 |
| CHP2_TP53_7 | CGCTTCTTGTCCTGCTTGCT | TTCTCTTTTCCTATCCTGAGTAGTGGT | GENOME_REGION | COSM11205 | hg19 | chr17 | 7576996 | 7577178 | 7577016 | 7577151 | 1 | Pool1 |
| CHP2_TP53_7 | CGCTTCTTGTCCTGCTTGCT | TTCTCTTTTCCTATCCTGAGTAGTGGT | GENOME_REGION | COSM11232 | hg19 | chr17 | 7576996 | 7577178 | 7577016 | 7577151 | 1 | Pool1 |
| CHP2_TP53_7 | CGCTTCTTGTCCTGCTTGCT | TTCTCTTTTCCTATCCTGAGTAGTGGT | GENOME_REGION | COSM11287 | hg19 | chr17 | 7576996 | 7577178 | 7577016 | 7577151 | 1 | Pool1 |
| CHP2_TP53_7 | CGCTTCTTGTCCTGCTTGCT | TTCTCTTTTCCTATCCTGAGTAGTGGT | GENOME_REGION | COSM11305 | hg19 | chr17 | 7576996 | 7577178 | 7577016 | 7577151 | 1 | Pool1 |
| CHP2_TP53_7 | CGCTTCTTGTCCTGCTTGCT | TTCTCTTTTCCTATCCTGAGTAGTGGT | GENOME_REGION | COSM11392 | hg19 | chr17 | 7576996 | 7577178 | 7577016 | 7577151 | 1 | Pool1 |
| CHP2_TP53_7 | CGCTTCTTGTCCTGCTTGCT | TTCTCTTTTCCTATCCTGAGTAGTGGT | GENOME_REGION | COSM11483 | hg19 | chr17 | 7576996 | 7577178 | 7577016 | 7577151 | 1 | Pool1 |
| CHP2_TP53_7 | CGCTTCTTGTCCTGCTTGCT | TTCTCTTTTCCTATCCTGAGTAGTGGT | GENOME_REGION | COSM11501 | hg19 | chr17 | 7576996 | 7577178 | 7577016 | 7577151 | 1 | Pool1 |
| CHP2_TP53_7 | CGCTTCTTGTCCTGCTTGCT | TTCTCTTTTCCTATCCTGAGTAGTGGT | GENOME_REGION | COSM11516 | hg19 | chr17 | 7576996 | 7577178 | 7577016 | 7577151 | 1 | Pool1 |
| CHP2_TP53_7 | CGCTTCTTGTCCTGCTTGCT | TTCTCTTTTCCTATCCTGAGTAGTGGT | GENOME_REGION | COSM11665 | hg19 | chr17 | 7576996 | 7577178 | 7577016 | 7577151 | 1 | Pool1 |
| CHP2_TP53_7 | CGCTTCTTGTCCTGCTTGCT | TTCTCTTTTCCTATCCTGAGTAGTGGT | GENOME_REGION | COSM126981 | hg19 | chr17 | 7576996 | 7577178 | 7577016 | 7577151 | 1 | Pool1 |
| CHP2_TP53_7 | CGCTTCTTGTCCTGCTTGCT | TTCTCTTTTCCTATCCTGAGTAGTGGT | GENOME_REGION | COSM129830 | hg19 | chr17 | 7576996 | 7577178 | 7577016 | 7577151 | 1 | Pool1 |
| CHP2_TP53_7 | CGCTTCTTGTCCTGCTTGCT | TTCTCTTTTCCTATCCTGAGTAGTGGT | GENOME_REGION | COSM129831 | hg19 | chr17 | 7576996 | 7577178 | 7577016 | 7577151 | 1 | Pool1 |
| CHP2_TP53_7 | CGCTTCTTGTCCTGCTTGCT | TTCTCTTTTCCTATCCTGAGTAGTGGT | GENOME_REGION | COSM13421 | hg19 | chr17 | 7576996 | 7577178 | 7577016 | 7577151 | 1 | Pool1 |
| CHP2_TP53_7 | CGCTTCTTGTCCTGCTTGCT | TTCTCTTTTCCTATCCTGAGTAGTGGT | GENOME_REGION | COSM137087 | hg19 | chr17 | 7576996 | 7577178 | 7577016 | 7577151 | 1 | Pool1 |
| CHP2_TP53_7 | CGCTTCTTGTCCTGCTTGCT | TTCTCTTTTCCTATCCTGAGTAGTGGT | GENOME_REGION | COSM139044 | hg19 | chr17 | 7576996 | 7577178 | 7577016 | 7577151 | 1 | Pool1 |
| CHP2_TP53_7 | CGCTTCTTGTCCTGCTTGCT | TTCTCTTTTCCTATCCTGAGTAGTGGT | GENOME_REGION | COSM146240 | hg19 | chr17 | 7576996 | 7577178 | 7577016 | 7577151 | 1 | Pool1 |
| CHP2_TP53_7 | CGCTTCTTGTCCTGCTTGCT | TTCTCTTTTCCTATCCTGAGTAGTGGT | GENOME_REGION | COSM165075 | hg19 | chr17 | 7576996 | 7577178 | 7577016 | 7577151 | 1 | Pool1 |
| CHP2_TP53_7 | CGCTTCTTGTCCTGCTTGCT | TTCTCTTTTCCTATCCTGAGTAGTGGT | GENOME_REGION | COSM165084 | hg19 | chr17 | 7576996 | 7577178 | 7577016 | 7577151 | 1 | Pool1 |
| CHP2_TP53_7 | CGCTTCTTGTCCTGCTTGCT | TTCTCTTTTCCTATCCTGAGTAGTGGT | GENOME_REGION | COSM179804 | hg19 | chr17 | 7576996 | 7577178 | 7577016 | 7577151 | 1 | Pool1 |
| CHP2_TP53_7 | CGCTTCTTGTCCTGCTTGCT | TTCTCTTTTCCTATCCTGAGTAGTGGT | GENOME_REGION | COSM43565 | hg19 | chr17 | 7576996 | 7577178 | 7577016 | 7577151 | 1 | Pool1 |
| CHP2_TP53_7 | CGCTTCTTGTCCTGCTTGCT | TTCTCTTTTCCTATCCTGAGTAGTGGT | GENOME_REGION | COSM43585 | hg19 | chr17 | 7576996 | 7577178 | 7577016 | 7577151 | 1 | Pool1 |
| CHP2_TP53_7 | CGCTTCTTGTCCTGCTTGCT | TTCTCTTTTCCTATCCTGAGTAGTGGT | GENOME_REGION | COSM43587 | hg19 | chr17 | 7576996 | 7577178 | 7577016 | 7577151 | 1 | Pool1 |
| CHP2_TP53_7 | CGCTTCTTGTCCTGCTTGCT | TTCTCTTTTCCTATCCTGAGTAGTGGT | GENOME_REGION | COSM43596 | hg19 | chr17 | 7576996 | 7577178 | 7577016 | 7577151 | 1 | Pool1 |
| CHP2_TP53_7 | CGCTTCTTGTCCTGCTTGCT | TTCTCTTTTCCTATCCTGAGTAGTGGT | GENOME_REGION | COSM43614 | hg19 | chr17 | 7576996 | 7577178 | 7577016 | 7577151 | 1 | Pool1 |
| CHP2_TP53_7 | CGCTTCTTGTCCTGCTTGCT | TTCTCTTTTCCTATCCTGAGTAGTGGT | GENOME_REGION | COSM43621 | hg19 | chr17 | 7576996 | 7577178 | 7577016 | 7577151 | 1 | Pool1 |
| CHP2_TP53_7 | CGCTTCTTGTCCTGCTTGCT | TTCTCTTTTCCTATCCTGAGTAGTGGT | GENOME_REGION | COSM43624 | hg19 | chr17 | 7576996 | 7577178 | 7577016 | 7577151 | 1 | Pool1 |
| CHP2_TP53_7 | CGCTTCTTGTCCTGCTTGCT | TTCTCTTTTCCTATCCTGAGTAGTGGT | GENOME_REGION | COSM43663 | hg19 | chr17 | 7576996 | 7577178 | 7577016 | 7577151 | 1 | Pool1 |
| CHP2_TP53_7 | CGCTTCTTGTCCTGCTTGCT | TTCTCTTTTCCTATCCTGAGTAGTGGT | GENOME_REGION | COSM43667 | hg19 | chr17 | 7576996 | 7577178 | 7577016 | 7577151 | 1 | Pool1 |
| CHP2_TP53_7 | CGCTTCTTGTCCTGCTTGCT | TTCTCTTTTCCTATCCTGAGTAGTGGT | GENOME_REGION | COSM43674 | hg19 | chr17 | 7576996 | 7577178 | 7577016 | 7577151 | 1 | Pool1 |
| CHP2_TP53_7 | CGCTTCTTGTCCTGCTTGCT | TTCTCTTTTCCTATCCTGAGTAGTGGT | GENOME_REGION | COSM43697 | hg19 | chr17 | 7576996 | 7577178 | 7577016 | 7577151 | 1 | Pool1 |
| CHP2_TP53_7 | CGCTTCTTGTCCTGCTTGCT | TTCTCTTTTCCTATCCTGAGTAGTGGT | GENOME_REGION | COSM43706 | hg19 | chr17 | 7576996 | 7577178 | 7577016 | 7577151 | 1 | Pool1 |
| CHP2_TP53_7 | CGCTTCTTGTCCTGCTTGCT | TTCTCTTTTCCTATCCTGAGTAGTGGT | GENOME_REGION | COSM43714 | hg19 | chr17 | 7576996 | 7577178 | 7577016 | 7577151 | 1 | Pool1 |
| CHP2_TP53_7 | CGCTTCTTGTCCTGCTTGCT | TTCTCTTTTCCTATCCTGAGTAGTGGT | GENOME_REGION | COSM43725 | hg19 | chr17 | 7576996 | 7577178 | 7577016 | 7577151 | 1 | Pool1 |
| CHP2_TP53_7 | CGCTTCTTGTCCTGCTTGCT | TTCTCTTTTCCTATCCTGAGTAGTGGT | GENOME_REGION | COSM43737 | hg19 | chr17 | 7576996 | 7577178 | 7577016 | 7577151 | 1 | Pool1 |
| CHP2_TP53_7 | CGCTTCTTGTCCTGCTTGCT | TTCTCTTTTCCTATCCTGAGTAGTGGT | GENOME_REGION | COSM43743 | hg19 | chr17 | 7576996 | 7577178 | 7577016 | 7577151 | 1 | Pool1 |
| CHP2_TP53_7 | CGCTTCTTGTCCTGCTTGCT | TTCTCTTTTCCTATCCTGAGTAGTGGT | GENOME_REGION | COSM43746 | hg19 | chr17 | 7576996 | 7577178 | 7577016 | 7577151 | 1 | Pool1 |
| CHP2_TP53_7 | CGCTTCTTGTCCTGCTTGCT | TTCTCTTTTCCTATCCTGAGTAGTGGT | GENOME_REGION | COSM43747 | hg19 | chr17 | 7576996 | 7577178 | 7577016 | 7577151 | 1 | Pool1 |
| CHP2_TP53_7 | CGCTTCTTGTCCTGCTTGCT | TTCTCTTTTCCTATCCTGAGTAGTGGT | GENOME_REGION | COSM43750 | hg19 | chr17 | 7576996 | 7577178 | 7577016 | 7577151 | 1 | Pool1 |
| CHP2_TP53_7 | CGCTTCTTGTCCTGCTTGCT | TTCTCTTTTCCTATCCTGAGTAGTGGT | GENOME_REGION | COSM43755 | hg19 | chr17 | 7576996 | 7577178 | 7577016 | 7577151 | 1 | Pool1 |
| CHP2_TP53_7 | CGCTTCTTGTCCTGCTTGCT | TTCTCTTTTCCTATCCTGAGTAGTGGT | GENOME_REGION | COSM43766 | hg19 | chr17 | 7576996 | 7577178 | 7577016 | 7577151 | 1 | Pool1 |
| CHP2_TP53_7 | CGCTTCTTGTCCTGCTTGCT | TTCTCTTTTCCTATCCTGAGTAGTGGT | GENOME_REGION | COSM43773 | hg19 | chr17 | 7576996 | 7577178 | 7577016 | 7577151 | 1 | Pool1 |
| CHP2_TP53_7 | CGCTTCTTGTCCTGCTTGCT | TTCTCTTTTCCTATCCTGAGTAGTGGT | GENOME_REGION | COSM43776 | hg19 | chr17 | 7576996 | 7577178 | 7577016 | 7577151 | 1 | Pool1 |
| CHP2_TP53_7 | CGCTTCTTGTCCTGCTTGCT | TTCTCTTTTCCTATCCTGAGTAGTGGT | GENOME_REGION | COSM43809 | hg19 | chr17 | 7576996 | 7577178 | 7577016 | 7577151 | 1 | Pool1 |
| CHP2_TP53_7 | CGCTTCTTGTCCTGCTTGCT | TTCTCTTTTCCTATCCTGAGTAGTGGT | GENOME_REGION | COSM43823 | hg19 | chr17 | 7576996 | 7577178 | 7577016 | 7577151 | 1 | Pool1 |
| CHP2_TP53_7 | CGCTTCTTGTCCTGCTTGCT | TTCTCTTTTCCTATCCTGAGTAGTGGT | GENOME_REGION | COSM43837 | hg19 | chr17 | 7576996 | 7577178 | 7577016 | 7577151 | 1 | Pool1 |
| CHP2_TP53_7 | CGCTTCTTGTCCTGCTTGCT | TTCTCTTTTCCTATCCTGAGTAGTGGT | GENOME_REGION | COSM43843 | hg19 | chr17 | 7576996 | 7577178 | 7577016 | 7577151 | 1 | Pool1 |
| CHP2_TP53_7 | CGCTTCTTGTCCTGCTTGCT | TTCTCTTTTCCTATCCTGAGTAGTGGT | GENOME_REGION | COSM43879 | hg19 | chr17 | 7576996 | 7577178 | 7577016 | 7577151 | 1 | Pool1 |
| CHP2_TP53_7 | CGCTTCTTGTCCTGCTTGCT | TTCTCTTTTCCTATCCTGAGTAGTGGT | GENOME_REGION | COSM43896 | hg19 | chr17 | 7576996 | 7577178 | 7577016 | 7577151 | 1 | Pool1 |
| CHP2_TP53_7 | CGCTTCTTGTCCTGCTTGCT | TTCTCTTTTCCTATCCTGAGTAGTGGT | GENOME_REGION | COSM43902 | hg19 | chr17 | 7576996 | 7577178 | 7577016 | 7577151 | 1 | Pool1 |
| CHP2_TP53_7 | CGCTTCTTGTCCTGCTTGCT | TTCTCTTTTCCTATCCTGAGTAGTGGT | GENOME_REGION | COSM43906 | hg19 | chr17 | 7576996 | 7577178 | 7577016 | 7577151 | 1 | Pool1 |
| CHP2_TP53_7 | CGCTTCTTGTCCTGCTTGCT | TTCTCTTTTCCTATCCTGAGTAGTGGT | GENOME_REGION | COSM43909 | hg19 | chr17 | 7576996 | 7577178 | 7577016 | 7577151 | 1 | Pool1 |
| CHP2_TP53_7 | CGCTTCTTGTCCTGCTTGCT | TTCTCTTTTCCTATCCTGAGTAGTGGT | GENOME_REGION | COSM43915 | hg19 | chr17 | 7576996 | 7577178 | 7577016 | 7577151 | 1 | Pool1 |
| CHP2_TP53_7 | CGCTTCTTGTCCTGCTTGCT | TTCTCTTTTCCTATCCTGAGTAGTGGT | GENOME_REGION | COSM43918 | hg19 | chr17 | 7576996 | 7577178 | 7577016 | 7577151 | 1 | Pool1 |
| CHP2_TP53_7 | CGCTTCTTGTCCTGCTTGCT | TTCTCTTTTCCTATCCTGAGTAGTGGT | GENOME_REGION | COSM43919 | hg19 | chr17 | 7576996 | 7577178 | 7577016 | 7577151 | 1 | Pool1 |
| CHP2_TP53_7 | CGCTTCTTGTCCTGCTTGCT | TTCTCTTTTCCTATCCTGAGTAGTGGT | GENOME_REGION | COSM43923 | hg19 | chr17 | 7576996 | 7577178 | 7577016 | 7577151 | 1 | Pool1 |
| CHP2_TP53_7 | CGCTTCTTGTCCTGCTTGCT | TTCTCTTTTCCTATCCTGAGTAGTGGT | GENOME_REGION | COSM43936 | hg19 | chr17 | 7576996 | 7577178 | 7577016 | 7577151 | 1 | Pool1 |
| CHP2_TP53_7 | CGCTTCTTGTCCTGCTTGCT | TTCTCTTTTCCTATCCTGAGTAGTGGT | GENOME_REGION | COSM43945 | hg19 | chr17 | 7576996 | 7577178 | 7577016 | 7577151 | 1 | Pool1 |
| CHP2_TP53_7 | CGCTTCTTGTCCTGCTTGCT | TTCTCTTTTCCTATCCTGAGTAGTGGT | GENOME_REGION | COSM43958 | hg19 | chr17 | 7576996 | 7577178 | 7577016 | 7577151 | 1 | Pool1 |
| CHP2_TP53_7 | CGCTTCTTGTCCTGCTTGCT | TTCTCTTTTCCTATCCTGAGTAGTGGT | GENOME_REGION | COSM43962 | hg19 | chr17 | 7576996 | 7577178 | 7577016 | 7577151 | 1 | Pool1 |
| CHP2_TP53_7 | CGCTTCTTGTCCTGCTTGCT | TTCTCTTTTCCTATCCTGAGTAGTGGT | GENOME_REGION | COSM43968 | hg19 | chr17 | 7576996 | 7577178 | 7577016 | 7577151 | 1 | Pool1 |
| CHP2_TP53_7 | CGCTTCTTGTCCTGCTTGCT | TTCTCTTTTCCTATCCTGAGTAGTGGT | GENOME_REGION | COSM43977 | hg19 | chr17 | 7576996 | 7577178 | 7577016 | 7577151 | 1 | Pool1 |
| CHP2_TP53_7 | CGCTTCTTGTCCTGCTTGCT | TTCTCTTTTCCTATCCTGAGTAGTGGT | GENOME_REGION | COSM43979 | hg19 | chr17 | 7576996 | 7577178 | 7577016 | 7577151 | 1 | Pool1 |
| CHP2_TP53_7 | CGCTTCTTGTCCTGCTTGCT | TTCTCTTTTCCTATCCTGAGTAGTGGT | GENOME_REGION | COSM43986 | hg19 | chr17 | 7576996 | 7577178 | 7577016 | 7577151 | 1 | Pool1 |
| CHP2_TP53_7 | CGCTTCTTGTCCTGCTTGCT | TTCTCTTTTCCTATCCTGAGTAGTGGT | GENOME_REGION | COSM43988 | hg19 | chr17 | 7576996 | 7577178 | 7577016 | 7577151 | 1 | Pool1 |
| CHP2_TP53_7 | CGCTTCTTGTCCTGCTTGCT | TTCTCTTTTCCTATCCTGAGTAGTGGT | GENOME_REGION | COSM44005 | hg19 | chr17 | 7576996 | 7577178 | 7577016 | 7577151 | 1 | Pool1 |
| CHP2_TP53_7 | CGCTTCTTGTCCTGCTTGCT | TTCTCTTTTCCTATCCTGAGTAGTGGT | GENOME_REGION | COSM44017 | hg19 | chr17 | 7576996 | 7577178 | 7577016 | 7577151 | 1 | Pool1 |
| CHP2_TP53_7 | CGCTTCTTGTCCTGCTTGCT | TTCTCTTTTCCTATCCTGAGTAGTGGT | GENOME_REGION | COSM44092 | hg19 | chr17 | 7576996 | 7577178 | 7577016 | 7577151 | 1 | Pool1 |
| CHP2_TP53_7 | CGCTTCTTGTCCTGCTTGCT | TTCTCTTTTCCTATCCTGAGTAGTGGT | GENOME_REGION | COSM44114 | hg19 | chr17 | 7576996 | 7577178 | 7577016 | 7577151 | 1 | Pool1 |
| CHP2_TP53_7 | CGCTTCTTGTCCTGCTTGCT | TTCTCTTTTCCTATCCTGAGTAGTGGT | GENOME_REGION | COSM44127 | hg19 | chr17 | 7576996 | 7577178 | 7577016 | 7577151 | 1 | Pool1 |
| CHP2_TP53_7 | CGCTTCTTGTCCTGCTTGCT | TTCTCTTTTCCTATCCTGAGTAGTGGT | GENOME_REGION | COSM44128 | hg19 | chr17 | 7576996 | 7577178 | 7577016 | 7577151 | 1 | Pool1 |
| CHP2_TP53_7 | CGCTTCTTGTCCTGCTTGCT | TTCTCTTTTCCTATCCTGAGTAGTGGT | GENOME_REGION | COSM44131 | hg19 | chr17 | 7576996 | 7577178 | 7577016 | 7577151 | 1 | Pool1 |
| CHP2_TP53_7 | CGCTTCTTGTCCTGCTTGCT | TTCTCTTTTCCTATCCTGAGTAGTGGT | GENOME_REGION | COSM44133 | hg19 | chr17 | 7576996 | 7577178 | 7577016 | 7577151 | 1 | Pool1 |
| CHP2_TP53_7 | CGCTTCTTGTCCTGCTTGCT | TTCTCTTTTCCTATCCTGAGTAGTGGT | GENOME_REGION | COSM44156 | hg19 | chr17 | 7576996 | 7577178 | 7577016 | 7577151 | 1 | Pool1 |
| CHP2_TP53_7 | CGCTTCTTGTCCTGCTTGCT | TTCTCTTTTCCTATCCTGAGTAGTGGT | GENOME_REGION | COSM44165 | hg19 | chr17 | 7576996 | 7577178 | 7577016 | 7577151 | 1 | Pool1 |
| CHP2_TP53_7 | CGCTTCTTGTCCTGCTTGCT | TTCTCTTTTCCTATCCTGAGTAGTGGT | GENOME_REGION | COSM44167 | hg19 | chr17 | 7576996 | 7577178 | 7577016 | 7577151 | 1 | Pool1 |
| CHP2_TP53_7 | CGCTTCTTGTCCTGCTTGCT | TTCTCTTTTCCTATCCTGAGTAGTGGT | GENOME_REGION | COSM44171 | hg19 | chr17 | 7576996 | 7577178 | 7577016 | 7577151 | 1 | Pool1 |
| CHP2_TP53_7 | CGCTTCTTGTCCTGCTTGCT | TTCTCTTTTCCTATCCTGAGTAGTGGT | GENOME_REGION | COSM44207 | hg19 | chr17 | 7576996 | 7577178 | 7577016 | 7577151 | 1 | Pool1 |
| CHP2_TP53_7 | CGCTTCTTGTCCTGCTTGCT | TTCTCTTTTCCTATCCTGAGTAGTGGT | GENOME_REGION | COSM44225 | hg19 | chr17 | 7576996 | 7577178 | 7577016 | 7577151 | 1 | Pool1 |
| CHP2_TP53_7 | CGCTTCTTGTCCTGCTTGCT | TTCTCTTTTCCTATCCTGAGTAGTGGT | GENOME_REGION | COSM44227 | hg19 | chr17 | 7576996 | 7577178 | 7577016 | 7577151 | 1 | Pool1 |
| CHP2_TP53_7 | CGCTTCTTGTCCTGCTTGCT | TTCTCTTTTCCTATCCTGAGTAGTGGT | GENOME_REGION | COSM44233 | hg19 | chr17 | 7576996 | 7577178 | 7577016 | 7577151 | 1 | Pool1 |
| CHP2_TP53_7 | CGCTTCTTGTCCTGCTTGCT | TTCTCTTTTCCTATCCTGAGTAGTGGT | GENOME_REGION | COSM44236 | hg19 | chr17 | 7576996 | 7577178 | 7577016 | 7577151 | 1 | Pool1 |
| CHP2_TP53_7 | CGCTTCTTGTCCTGCTTGCT | TTCTCTTTTCCTATCCTGAGTAGTGGT | GENOME_REGION | COSM44237 | hg19 | chr17 | 7576996 | 7577178 | 7577016 | 7577151 | 1 | Pool1 |
| CHP2_TP53_7 | CGCTTCTTGTCCTGCTTGCT | TTCTCTTTTCCTATCCTGAGTAGTGGT | GENOME_REGION | COSM44250 | hg19 | chr17 | 7576996 | 7577178 | 7577016 | 7577151 | 1 | Pool1 |
| CHP2_TP53_7 | CGCTTCTTGTCCTGCTTGCT | TTCTCTTTTCCTATCCTGAGTAGTGGT | GENOME_REGION | COSM44262 | hg19 | chr17 | 7576996 | 7577178 | 7577016 | 7577151 | 1 | Pool1 |
| CHP2_TP53_7 | CGCTTCTTGTCCTGCTTGCT | TTCTCTTTTCCTATCCTGAGTAGTGGT | GENOME_REGION | COSM44292 | hg19 | chr17 | 7576996 | 7577178 | 7577016 | 7577151 | 1 | Pool1 |
| CHP2_TP53_7 | CGCTTCTTGTCCTGCTTGCT | TTCTCTTTTCCTATCCTGAGTAGTGGT | GENOME_REGION | COSM44294 | hg19 | chr17 | 7576996 | 7577178 | 7577016 | 7577151 | 1 | Pool1 |
| CHP2_TP53_7 | CGCTTCTTGTCCTGCTTGCT | TTCTCTTTTCCTATCCTGAGTAGTGGT | GENOME_REGION | COSM44306 | hg19 | chr17 | 7576996 | 7577178 | 7577016 | 7577151 | 1 | Pool1 |
| CHP2_TP53_7 | CGCTTCTTGTCCTGCTTGCT | TTCTCTTTTCCTATCCTGAGTAGTGGT | GENOME_REGION | COSM44338 | hg19 | chr17 | 7576996 | 7577178 | 7577016 | 7577151 | 1 | Pool1 |
| CHP2_TP53_7 | CGCTTCTTGTCCTGCTTGCT | TTCTCTTTTCCTATCCTGAGTAGTGGT | GENOME_REGION | COSM44345 | hg19 | chr17 | 7576996 | 7577178 | 7577016 | 7577151 | 1 | Pool1 |
| CHP2_TP53_7 | CGCTTCTTGTCCTGCTTGCT | TTCTCTTTTCCTATCCTGAGTAGTGGT | GENOME_REGION | COSM44346 | hg19 | chr17 | 7576996 | 7577178 | 7577016 | 7577151 | 1 | Pool1 |
| CHP2_TP53_7 | CGCTTCTTGTCCTGCTTGCT | TTCTCTTTTCCTATCCTGAGTAGTGGT | GENOME_REGION | COSM44352 | hg19 | chr17 | 7576996 | 7577178 | 7577016 | 7577151 | 1 | Pool1 |
| CHP2_TP53_7 | CGCTTCTTGTCCTGCTTGCT | TTCTCTTTTCCTATCCTGAGTAGTGGT | GENOME_REGION | COSM44388 | hg19 | chr17 | 7576996 | 7577178 | 7577016 | 7577151 | 1 | Pool1 |
| CHP2_TP53_7 | CGCTTCTTGTCCTGCTTGCT | TTCTCTTTTCCTATCCTGAGTAGTGGT | GENOME_REGION | COSM44390 | hg19 | chr17 | 7576996 | 7577178 | 7577016 | 7577151 | 1 | Pool1 |
| CHP2_TP53_7 | CGCTTCTTGTCCTGCTTGCT | TTCTCTTTTCCTATCCTGAGTAGTGGT | GENOME_REGION | COSM44393 | hg19 | chr17 | 7576996 | 7577178 | 7577016 | 7577151 | 1 | Pool1 |
| CHP2_TP53_7 | CGCTTCTTGTCCTGCTTGCT | TTCTCTTTTCCTATCCTGAGTAGTGGT | GENOME_REGION | COSM44412 | hg19 | chr17 | 7576996 | 7577178 | 7577016 | 7577151 | 1 | Pool1 |
| CHP2_TP53_7 | CGCTTCTTGTCCTGCTTGCT | TTCTCTTTTCCTATCCTGAGTAGTGGT | GENOME_REGION | COSM44417 | hg19 | chr17 | 7576996 | 7577178 | 7577016 | 7577151 | 1 | Pool1 |
| CHP2_TP53_7 | CGCTTCTTGTCCTGCTTGCT | TTCTCTTTTCCTATCCTGAGTAGTGGT | GENOME_REGION | COSM44433 | hg19 | chr17 | 7576996 | 7577178 | 7577016 | 7577151 | 1 | Pool1 |
| CHP2_TP53_7 | CGCTTCTTGTCCTGCTTGCT | TTCTCTTTTCCTATCCTGAGTAGTGGT | GENOME_REGION | COSM44441 | hg19 | chr17 | 7576996 | 7577178 | 7577016 | 7577151 | 1 | Pool1 |
| CHP2_TP53_7 | CGCTTCTTGTCCTGCTTGCT | TTCTCTTTTCCTATCCTGAGTAGTGGT | GENOME_REGION | COSM44443 | hg19 | chr17 | 7576996 | 7577178 | 7577016 | 7577151 | 1 | Pool1 |
| CHP2_TP53_7 | CGCTTCTTGTCCTGCTTGCT | TTCTCTTTTCCTATCCTGAGTAGTGGT | GENOME_REGION | COSM44446 | hg19 | chr17 | 7576996 | 7577178 | 7577016 | 7577151 | 1 | Pool1 |
| CHP2_TP53_7 | CGCTTCTTGTCCTGCTTGCT | TTCTCTTTTCCTATCCTGAGTAGTGGT | GENOME_REGION | COSM44448 | hg19 | chr17 | 7576996 | 7577178 | 7577016 | 7577151 | 1 | Pool1 |
| CHP2_TP53_7 | CGCTTCTTGTCCTGCTTGCT | TTCTCTTTTCCTATCCTGAGTAGTGGT | GENOME_REGION | COSM44451 | hg19 | chr17 | 7576996 | 7577178 | 7577016 | 7577151 | 1 | Pool1 |
| CHP2_TP53_7 | CGCTTCTTGTCCTGCTTGCT | TTCTCTTTTCCTATCCTGAGTAGTGGT | GENOME_REGION | COSM44463 | hg19 | chr17 | 7576996 | 7577178 | 7577016 | 7577151 | 1 | Pool1 |
| CHP2_TP53_7 | CGCTTCTTGTCCTGCTTGCT | TTCTCTTTTCCTATCCTGAGTAGTGGT | GENOME_REGION | COSM44469 | hg19 | chr17 | 7576996 | 7577178 | 7577016 | 7577151 | 1 | Pool1 |
| CHP2_TP53_7 | CGCTTCTTGTCCTGCTTGCT | TTCTCTTTTCCTATCCTGAGTAGTGGT | GENOME_REGION | COSM44470 | hg19 | chr17 | 7576996 | 7577178 | 7577016 | 7577151 | 1 | Pool1 |
| CHP2_TP53_7 | CGCTTCTTGTCCTGCTTGCT | TTCTCTTTTCCTATCCTGAGTAGTGGT | GENOME_REGION | COSM44475 | hg19 | chr17 | 7576996 | 7577178 | 7577016 | 7577151 | 1 | Pool1 |
| CHP2_TP53_7 | CGCTTCTTGTCCTGCTTGCT | TTCTCTTTTCCTATCCTGAGTAGTGGT | GENOME_REGION | COSM44522 | hg19 | chr17 | 7576996 | 7577178 | 7577016 | 7577151 | 1 | Pool1 |
| CHP2_TP53_7 | CGCTTCTTGTCCTGCTTGCT | TTCTCTTTTCCTATCCTGAGTAGTGGT | GENOME_REGION | COSM44523 | hg19 | chr17 | 7576996 | 7577178 | 7577016 | 7577151 | 1 | Pool1 |
| CHP2_TP53_7 | CGCTTCTTGTCCTGCTTGCT | TTCTCTTTTCCTATCCTGAGTAGTGGT | GENOME_REGION | COSM44565 | hg19 | chr17 | 7576996 | 7577178 | 7577016 | 7577151 | 1 | Pool1 |
| CHP2_TP53_7 | CGCTTCTTGTCCTGCTTGCT | TTCTCTTTTCCTATCCTGAGTAGTGGT | GENOME_REGION | COSM44568 | hg19 | chr17 | 7576996 | 7577178 | 7577016 | 7577151 | 1 | Pool1 |
| CHP2_TP53_7 | CGCTTCTTGTCCTGCTTGCT | TTCTCTTTTCCTATCCTGAGTAGTGGT | GENOME_REGION | COSM44580 | hg19 | chr17 | 7576996 | 7577178 | 7577016 | 7577151 | 1 | Pool1 |
| CHP2_TP53_7 | CGCTTCTTGTCCTGCTTGCT | TTCTCTTTTCCTATCCTGAGTAGTGGT | GENOME_REGION | COSM44603 | hg19 | chr17 | 7576996 | 7577178 | 7577016 | 7577151 | 1 | Pool1 |
| CHP2_TP53_7 | CGCTTCTTGTCCTGCTTGCT | TTCTCTTTTCCTATCCTGAGTAGTGGT | GENOME_REGION | COSM44639 | hg19 | chr17 | 7576996 | 7577178 | 7577016 | 7577151 | 1 | Pool1 |
| CHP2_TP53_7 | CGCTTCTTGTCCTGCTTGCT | TTCTCTTTTCCTATCCTGAGTAGTGGT | GENOME_REGION | COSM44651 | hg19 | chr17 | 7576996 | 7577178 | 7577016 | 7577151 | 1 | Pool1 |
| CHP2_TP53_7 | CGCTTCTTGTCCTGCTTGCT | TTCTCTTTTCCTATCCTGAGTAGTGGT | GENOME_REGION | COSM44701 | hg19 | chr17 | 7576996 | 7577178 | 7577016 | 7577151 | 1 | Pool1 |
| CHP2_TP53_7 | CGCTTCTTGTCCTGCTTGCT | TTCTCTTTTCCTATCCTGAGTAGTGGT | GENOME_REGION | COSM44709 | hg19 | chr17 | 7576996 | 7577178 | 7577016 | 7577151 | 1 | Pool1 |
| CHP2_TP53_7 | CGCTTCTTGTCCTGCTTGCT | TTCTCTTTTCCTATCCTGAGTAGTGGT | GENOME_REGION | COSM44724 | hg19 | chr17 | 7576996 | 7577178 | 7577016 | 7577151 | 1 | Pool1 |
| CHP2_TP53_7 | CGCTTCTTGTCCTGCTTGCT | TTCTCTTTTCCTATCCTGAGTAGTGGT | GENOME_REGION | COSM44729 | hg19 | chr17 | 7576996 | 7577178 | 7577016 | 7577151 | 1 | Pool1 |
| CHP2_TP53_7 | CGCTTCTTGTCCTGCTTGCT | TTCTCTTTTCCTATCCTGAGTAGTGGT | GENOME_REGION | COSM44735 | hg19 | chr17 | 7576996 | 7577178 | 7577016 | 7577151 | 1 | Pool1 |
| CHP2_TP53_7 | CGCTTCTTGTCCTGCTTGCT | TTCTCTTTTCCTATCCTGAGTAGTGGT | GENOME_REGION | COSM44737 | hg19 | chr17 | 7576996 | 7577178 | 7577016 | 7577151 | 1 | Pool1 |
| CHP2_TP53_7 | CGCTTCTTGTCCTGCTTGCT | TTCTCTTTTCCTATCCTGAGTAGTGGT | GENOME_REGION | COSM44750 | hg19 | chr17 | 7576996 | 7577178 | 7577016 | 7577151 | 1 | Pool1 |
| CHP2_TP53_7 | CGCTTCTTGTCCTGCTTGCT | TTCTCTTTTCCTATCCTGAGTAGTGGT | GENOME_REGION | COSM44753 | hg19 | chr17 | 7576996 | 7577178 | 7577016 | 7577151 | 1 | Pool1 |
| CHP2_TP53_7 | CGCTTCTTGTCCTGCTTGCT | TTCTCTTTTCCTATCCTGAGTAGTGGT | GENOME_REGION | COSM44835 | hg19 | chr17 | 7576996 | 7577178 | 7577016 | 7577151 | 1 | Pool1 |
| CHP2_TP53_7 | CGCTTCTTGTCCTGCTTGCT | TTCTCTTTTCCTATCCTGAGTAGTGGT | GENOME_REGION | COSM44868 | hg19 | chr17 | 7576996 | 7577178 | 7577016 | 7577151 | 1 | Pool1 |
| CHP2_TP53_7 | CGCTTCTTGTCCTGCTTGCT | TTCTCTTTTCCTATCCTGAGTAGTGGT | GENOME_REGION | COSM44870 | hg19 | chr17 | 7576996 | 7577178 | 7577016 | 7577151 | 1 | Pool1 |
| CHP2_TP53_7 | CGCTTCTTGTCCTGCTTGCT | TTCTCTTTTCCTATCCTGAGTAGTGGT | GENOME_REGION | COSM44871 | hg19 | chr17 | 7576996 | 7577178 | 7577016 | 7577151 | 1 | Pool1 |
| CHP2_TP53_7 | CGCTTCTTGTCCTGCTTGCT | TTCTCTTTTCCTATCCTGAGTAGTGGT | GENOME_REGION | COSM44886 | hg19 | chr17 | 7576996 | 7577178 | 7577016 | 7577151 | 1 | Pool1 |
| CHP2_TP53_7 | CGCTTCTTGTCCTGCTTGCT | TTCTCTTTTCCTATCCTGAGTAGTGGT | GENOME_REGION | COSM44891 | hg19 | chr17 | 7576996 | 7577178 | 7577016 | 7577151 | 1 | Pool1 |
| CHP2_TP53_7 | CGCTTCTTGTCCTGCTTGCT | TTCTCTTTTCCTATCCTGAGTAGTGGT | GENOME_REGION | COSM44896 | hg19 | chr17 | 7576996 | 7577178 | 7577016 | 7577151 | 1 | Pool1 |
| CHP2_TP53_7 | CGCTTCTTGTCCTGCTTGCT | TTCTCTTTTCCTATCCTGAGTAGTGGT | GENOME_REGION | COSM44897 | hg19 | chr17 | 7576996 | 7577178 | 7577016 | 7577151 | 1 | Pool1 |
| CHP2_TP53_7 | CGCTTCTTGTCCTGCTTGCT | TTCTCTTTTCCTATCCTGAGTAGTGGT | GENOME_REGION | COSM44918 | hg19 | chr17 | 7576996 | 7577178 | 7577016 | 7577151 | 1 | Pool1 |
| CHP2_TP53_7 | CGCTTCTTGTCCTGCTTGCT | TTCTCTTTTCCTATCCTGAGTAGTGGT | GENOME_REGION | COSM44956 | hg19 | chr17 | 7576996 | 7577178 | 7577016 | 7577151 | 1 | Pool1 |
| CHP2_TP53_7 | CGCTTCTTGTCCTGCTTGCT | TTCTCTTTTCCTATCCTGAGTAGTGGT | GENOME_REGION | COSM44972 | hg19 | chr17 | 7576996 | 7577178 | 7577016 | 7577151 | 1 | Pool1 |
| CHP2_TP53_7 | CGCTTCTTGTCCTGCTTGCT | TTCTCTTTTCCTATCCTGAGTAGTGGT | GENOME_REGION | COSM45050 | hg19 | chr17 | 7576996 | 7577178 | 7577016 | 7577151 | 1 | Pool1 |
| CHP2_TP53_7 | CGCTTCTTGTCCTGCTTGCT | TTCTCTTTTCCTATCCTGAGTAGTGGT | GENOME_REGION | COSM45069 | hg19 | chr17 | 7576996 | 7577178 | 7577016 | 7577151 | 1 | Pool1 |
| CHP2_TP53_7 | CGCTTCTTGTCCTGCTTGCT | TTCTCTTTTCCTATCCTGAGTAGTGGT | GENOME_REGION | COSM45074 | hg19 | chr17 | 7576996 | 7577178 | 7577016 | 7577151 | 1 | Pool1 |
| CHP2_TP53_7 | CGCTTCTTGTCCTGCTTGCT | TTCTCTTTTCCTATCCTGAGTAGTGGT | GENOME_REGION | COSM45109 | hg19 | chr17 | 7576996 | 7577178 | 7577016 | 7577151 | 1 | Pool1 |
| CHP2_TP53_7 | CGCTTCTTGTCCTGCTTGCT | TTCTCTTTTCCTATCCTGAGTAGTGGT | GENOME_REGION | COSM45128 | hg19 | chr17 | 7576996 | 7577178 | 7577016 | 7577151 | 1 | Pool1 |
| CHP2_TP53_7 | CGCTTCTTGTCCTGCTTGCT | TTCTCTTTTCCTATCCTGAGTAGTGGT | GENOME_REGION | COSM45138 | hg19 | chr17 | 7576996 | 7577178 | 7577016 | 7577151 | 1 | Pool1 |
| CHP2_TP53_7 | CGCTTCTTGTCCTGCTTGCT | TTCTCTTTTCCTATCCTGAGTAGTGGT | GENOME_REGION | COSM45178 | hg19 | chr17 | 7576996 | 7577178 | 7577016 | 7577151 | 1 | Pool1 |
| CHP2_TP53_7 | CGCTTCTTGTCCTGCTTGCT | TTCTCTTTTCCTATCCTGAGTAGTGGT | GENOME_REGION | COSM45184 | hg19 | chr17 | 7576996 | 7577178 | 7577016 | 7577151 | 1 | Pool1 |
| CHP2_TP53_7 | CGCTTCTTGTCCTGCTTGCT | TTCTCTTTTCCTATCCTGAGTAGTGGT | GENOME_REGION | COSM45188 | hg19 | chr17 | 7576996 | 7577178 | 7577016 | 7577151 | 1 | Pool1 |
| CHP2_TP53_7 | CGCTTCTTGTCCTGCTTGCT | TTCTCTTTTCCTATCCTGAGTAGTGGT | GENOME_REGION | COSM45233 | hg19 | chr17 | 7576996 | 7577178 | 7577016 | 7577151 | 1 | Pool1 |
| CHP2_TP53_7 | CGCTTCTTGTCCTGCTTGCT | TTCTCTTTTCCTATCCTGAGTAGTGGT | GENOME_REGION | COSM45248 | hg19 | chr17 | 7576996 | 7577178 | 7577016 | 7577151 | 1 | Pool1 |
| CHP2_TP53_7 | CGCTTCTTGTCCTGCTTGCT | TTCTCTTTTCCTATCCTGAGTAGTGGT | GENOME_REGION | COSM45268 | hg19 | chr17 | 7576996 | 7577178 | 7577016 | 7577151 | 1 | Pool1 |
| CHP2_TP53_7 | CGCTTCTTGTCCTGCTTGCT | TTCTCTTTTCCTATCCTGAGTAGTGGT | GENOME_REGION | COSM45277 | hg19 | chr17 | 7576996 | 7577178 | 7577016 | 7577151 | 1 | Pool1 |
| CHP2_TP53_7 | CGCTTCTTGTCCTGCTTGCT | TTCTCTTTTCCTATCCTGAGTAGTGGT | GENOME_REGION | COSM45284 | hg19 | chr17 | 7576996 | 7577178 | 7577016 | 7577151 | 1 | Pool1 |
| CHP2_TP53_7 | CGCTTCTTGTCCTGCTTGCT | TTCTCTTTTCCTATCCTGAGTAGTGGT | GENOME_REGION | COSM45297 | hg19 | chr17 | 7576996 | 7577178 | 7577016 | 7577151 | 1 | Pool1 |
| CHP2_TP53_7 | CGCTTCTTGTCCTGCTTGCT | TTCTCTTTTCCTATCCTGAGTAGTGGT | GENOME_REGION | COSM45299 | hg19 | chr17 | 7576996 | 7577178 | 7577016 | 7577151 | 1 | Pool1 |
| CHP2_TP53_7 | CGCTTCTTGTCCTGCTTGCT | TTCTCTTTTCCTATCCTGAGTAGTGGT | GENOME_REGION | COSM45306 | hg19 | chr17 | 7576996 | 7577178 | 7577016 | 7577151 | 1 | Pool1 |
| CHP2_TP53_7 | CGCTTCTTGTCCTGCTTGCT | TTCTCTTTTCCTATCCTGAGTAGTGGT | GENOME_REGION | COSM45311 | hg19 | chr17 | 7576996 | 7577178 | 7577016 | 7577151 | 1 | Pool1 |
| CHP2_TP53_7 | CGCTTCTTGTCCTGCTTGCT | TTCTCTTTTCCTATCCTGAGTAGTGGT | GENOME_REGION | COSM45332 | hg19 | chr17 | 7576996 | 7577178 | 7577016 | 7577151 | 1 | Pool1 |
| CHP2_TP53_7 | CGCTTCTTGTCCTGCTTGCT | TTCTCTTTTCCTATCCTGAGTAGTGGT | GENOME_REGION | COSM45393 | hg19 | chr17 | 7576996 | 7577178 | 7577016 | 7577151 | 1 | Pool1 |
| CHP2_TP53_7 | CGCTTCTTGTCCTGCTTGCT | TTCTCTTTTCCTATCCTGAGTAGTGGT | GENOME_REGION | COSM45413 | hg19 | chr17 | 7576996 | 7577178 | 7577016 | 7577151 | 1 | Pool1 |
| CHP2_TP53_7 | CGCTTCTTGTCCTGCTTGCT | TTCTCTTTTCCTATCCTGAGTAGTGGT | GENOME_REGION | COSM45417 | hg19 | chr17 | 7576996 | 7577178 | 7577016 | 7577151 | 1 | Pool1 |
| CHP2_TP53_7 | CGCTTCTTGTCCTGCTTGCT | TTCTCTTTTCCTATCCTGAGTAGTGGT | GENOME_REGION | COSM45446 | hg19 | chr17 | 7576996 | 7577178 | 7577016 | 7577151 | 1 | Pool1 |
| CHP2_TP53_7 | CGCTTCTTGTCCTGCTTGCT | TTCTCTTTTCCTATCCTGAGTAGTGGT | GENOME_REGION | COSM45459 | hg19 | chr17 | 7576996 | 7577178 | 7577016 | 7577151 | 1 | Pool1 |
| CHP2_TP53_7 | CGCTTCTTGTCCTGCTTGCT | TTCTCTTTTCCTATCCTGAGTAGTGGT | GENOME_REGION | COSM45467 | hg19 | chr17 | 7576996 | 7577178 | 7577016 | 7577151 | 1 | Pool1 |
| CHP2_TP53_7 | CGCTTCTTGTCCTGCTTGCT | TTCTCTTTTCCTATCCTGAGTAGTGGT | GENOME_REGION | COSM45487 | hg19 | chr17 | 7576996 | 7577178 | 7577016 | 7577151 | 1 | Pool1 |
| CHP2_TP53_7 | CGCTTCTTGTCCTGCTTGCT | TTCTCTTTTCCTATCCTGAGTAGTGGT | GENOME_REGION | COSM45488 | hg19 | chr17 | 7576996 | 7577178 | 7577016 | 7577151 | 1 | Pool1 |
| CHP2_TP53_7 | CGCTTCTTGTCCTGCTTGCT | TTCTCTTTTCCTATCCTGAGTAGTGGT | GENOME_REGION | COSM45491 | hg19 | chr17 | 7576996 | 7577178 | 7577016 | 7577151 | 1 | Pool1 |
| CHP2_TP53_7 | CGCTTCTTGTCCTGCTTGCT | TTCTCTTTTCCTATCCTGAGTAGTGGT | GENOME_REGION | COSM45494 | hg19 | chr17 | 7576996 | 7577178 | 7577016 | 7577151 | 1 | Pool1 |
| CHP2_TP53_7 | CGCTTCTTGTCCTGCTTGCT | TTCTCTTTTCCTATCCTGAGTAGTGGT | GENOME_REGION | COSM45507 | hg19 | chr17 | 7576996 | 7577178 | 7577016 | 7577151 | 1 | Pool1 |
| CHP2_TP53_7 | CGCTTCTTGTCCTGCTTGCT | TTCTCTTTTCCTATCCTGAGTAGTGGT | GENOME_REGION | COSM45534 | hg19 | chr17 | 7576996 | 7577178 | 7577016 | 7577151 | 1 | Pool1 |
| CHP2_TP53_7 | CGCTTCTTGTCCTGCTTGCT | TTCTCTTTTCCTATCCTGAGTAGTGGT | GENOME_REGION | COSM45546 | hg19 | chr17 | 7576996 | 7577178 | 7577016 | 7577151 | 1 | Pool1 |
| CHP2_TP53_7 | CGCTTCTTGTCCTGCTTGCT | TTCTCTTTTCCTATCCTGAGTAGTGGT | GENOME_REGION | COSM45611 | hg19 | chr17 | 7576996 | 7577178 | 7577016 | 7577151 | 1 | Pool1 |
| CHP2_TP53_7 | CGCTTCTTGTCCTGCTTGCT | TTCTCTTTTCCTATCCTGAGTAGTGGT | GENOME_REGION | COSM45649 | hg19 | chr17 | 7576996 | 7577178 | 7577016 | 7577151 | 1 | Pool1 |
| CHP2_TP53_7 | CGCTTCTTGTCCTGCTTGCT | TTCTCTTTTCCTATCCTGAGTAGTGGT | GENOME_REGION | COSM45670 | hg19 | chr17 | 7576996 | 7577178 | 7577016 | 7577151 | 1 | Pool1 |
| CHP2_TP53_7 | CGCTTCTTGTCCTGCTTGCT | TTCTCTTTTCCTATCCTGAGTAGTGGT | GENOME_REGION | COSM45679 | hg19 | chr17 | 7576996 | 7577178 | 7577016 | 7577151 | 1 | Pool1 |
| CHP2_TP53_7 | CGCTTCTTGTCCTGCTTGCT | TTCTCTTTTCCTATCCTGAGTAGTGGT | GENOME_REGION | COSM45688 | hg19 | chr17 | 7576996 | 7577178 | 7577016 | 7577151 | 1 | Pool1 |
| CHP2_TP53_7 | CGCTTCTTGTCCTGCTTGCT | TTCTCTTTTCCTATCCTGAGTAGTGGT | GENOME_REGION | COSM45695 | hg19 | chr17 | 7576996 | 7577178 | 7577016 | 7577151 | 1 | Pool1 |
| CHP2_TP53_7 | CGCTTCTTGTCCTGCTTGCT | TTCTCTTTTCCTATCCTGAGTAGTGGT | GENOME_REGION | COSM45706 | hg19 | chr17 | 7576996 | 7577178 | 7577016 | 7577151 | 1 | Pool1 |
| CHP2_TP53_7 | CGCTTCTTGTCCTGCTTGCT | TTCTCTTTTCCTATCCTGAGTAGTGGT | GENOME_REGION | COSM45728 | hg19 | chr17 | 7576996 | 7577178 | 7577016 | 7577151 | 1 | Pool1 |
| CHP2_TP53_7 | CGCTTCTTGTCCTGCTTGCT | TTCTCTTTTCCTATCCTGAGTAGTGGT | GENOME_REGION | COSM45729 | hg19 | chr17 | 7576996 | 7577178 | 7577016 | 7577151 | 1 | Pool1 |
| CHP2_TP53_7 | CGCTTCTTGTCCTGCTTGCT | TTCTCTTTTCCTATCCTGAGTAGTGGT | GENOME_REGION | COSM45803 | hg19 | chr17 | 7576996 | 7577178 | 7577016 | 7577151 | 1 | Pool1 |
| CHP2_TP53_7 | CGCTTCTTGTCCTGCTTGCT | TTCTCTTTTCCTATCCTGAGTAGTGGT | GENOME_REGION | COSM45820 | hg19 | chr17 | 7576996 | 7577178 | 7577016 | 7577151 | 1 | Pool1 |
| CHP2_TP53_7 | CGCTTCTTGTCCTGCTTGCT | TTCTCTTTTCCTATCCTGAGTAGTGGT | GENOME_REGION | COSM45824 | hg19 | chr17 | 7576996 | 7577178 | 7577016 | 7577151 | 1 | Pool1 |
| CHP2_TP53_7 | CGCTTCTTGTCCTGCTTGCT | TTCTCTTTTCCTATCCTGAGTAGTGGT | GENOME_REGION | COSM45843 | hg19 | chr17 | 7576996 | 7577178 | 7577016 | 7577151 | 1 | Pool1 |
| CHP2_TP53_7 | CGCTTCTTGTCCTGCTTGCT | TTCTCTTTTCCTATCCTGAGTAGTGGT | GENOME_REGION | COSM45891 | hg19 | chr17 | 7576996 | 7577178 | 7577016 | 7577151 | 1 | Pool1 |
| CHP2_TP53_7 | CGCTTCTTGTCCTGCTTGCT | TTCTCTTTTCCTATCCTGAGTAGTGGT | GENOME_REGION | COSM45898 | hg19 | chr17 | 7576996 | 7577178 | 7577016 | 7577151 | 1 | Pool1 |
| CHP2_TP53_7 | CGCTTCTTGTCCTGCTTGCT | TTCTCTTTTCCTATCCTGAGTAGTGGT | GENOME_REGION | COSM45998 | hg19 | chr17 | 7576996 | 7577178 | 7577016 | 7577151 | 1 | Pool1 |
| CHP2_TP53_7 | CGCTTCTTGTCCTGCTTGCT | TTCTCTTTTCCTATCCTGAGTAGTGGT | GENOME_REGION | COSM46032 | hg19 | chr17 | 7576996 | 7577178 | 7577016 | 7577151 | 1 | Pool1 |
| CHP2_TP53_7 | CGCTTCTTGTCCTGCTTGCT | TTCTCTTTTCCTATCCTGAGTAGTGGT | GENOME_REGION | COSM46035 | hg19 | chr17 | 7576996 | 7577178 | 7577016 | 7577151 | 1 | Pool1 |
| CHP2_TP53_7 | CGCTTCTTGTCCTGCTTGCT | TTCTCTTTTCCTATCCTGAGTAGTGGT | GENOME_REGION | COSM46207 | hg19 | chr17 | 7576996 | 7577178 | 7577016 | 7577151 | 1 | Pool1 |
| CHP2_TP53_7 | CGCTTCTTGTCCTGCTTGCT | TTCTCTTTTCCTATCCTGAGTAGTGGT | GENOME_REGION | COSM46208 | hg19 | chr17 | 7576996 | 7577178 | 7577016 | 7577151 | 1 | Pool1 |
| CHP2_TP53_7 | CGCTTCTTGTCCTGCTTGCT | TTCTCTTTTCCTATCCTGAGTAGTGGT | GENOME_REGION | COSM46224 | hg19 | chr17 | 7576996 | 7577178 | 7577016 | 7577151 | 1 | Pool1 |
| CHP2_TP53_7 | CGCTTCTTGTCCTGCTTGCT | TTCTCTTTTCCTATCCTGAGTAGTGGT | GENOME_REGION | COSM46284 | hg19 | chr17 | 7576996 | 7577178 | 7577016 | 7577151 | 1 | Pool1 |
| CHP2_TP53_7 | CGCTTCTTGTCCTGCTTGCT | TTCTCTTTTCCTATCCTGAGTAGTGGT | GENOME_REGION | COSM6621 | hg19 | chr17 | 7576996 | 7577178 | 7577016 | 7577151 | 1 | Pool1 |
| CHP2_TP53_7 | CGCTTCTTGTCCTGCTTGCT | TTCTCTTTTCCTATCCTGAGTAGTGGT | GENOME_REGION | COSM87513 | hg19 | chr17 | 7576996 | 7577178 | 7577016 | 7577151 | 1 | Pool1 |
| CHP2_TP53_7 | CGCTTCTTGTCCTGCTTGCT | TTCTCTTTTCCTATCCTGAGTAGTGGT | GENOME_REGION | COSM99725 | hg19 | chr17 | 7576996 | 7577178 | 7577016 | 7577151 | 1 | Pool1 |
| CHP2_TP53_7 | CGCTTCTTGTCCTGCTTGCT | TTCTCTTTTCCTATCCTGAGTAGTGGT | GENOME_REGION | COSM99729 | hg19 | chr17 | 7576996 | 7577178 | 7577016 | 7577151 | 1 | Pool1 |
| CHP2_TP53_7 | CGCTTCTTGTCCTGCTTGCT | TTCTCTTTTCCTATCCTGAGTAGTGGT | GENOME_REGION | COSM99924 | hg19 | chr17 | 7576996 | 7577178 | 7577016 | 7577151 | 1 | Pool1 |
| CHP2_TP53_7 | CGCTTCTTGTCCTGCTTGCT | TTCTCTTTTCCTATCCTGAGTAGTGGT | GENOME_REGION | COSM99925 | hg19 | chr17 | 7576996 | 7577178 | 7577016 | 7577151 | 1 | Pool1 |
| CHP2_TP53_7 | CGCTTCTTGTCCTGCTTGCT | TTCTCTTTTCCTATCCTGAGTAGTGGT | GENOME_REGION | COSM99932 | hg19 | chr17 | 7576996 | 7577178 | 7577016 | 7577151 | 1 | Pool1 |
| CHP2_TP53_7 | CGCTTCTTGTCCTGCTTGCT | TTCTCTTTTCCTATCCTGAGTAGTGGT | GENOME_REGION | COSM99933 | hg19 | chr17 | 7576996 | 7577178 | 7577016 | 7577151 | 1 | Pool1 |
| CHP2_TP53_7 | CGCTTCTTGTCCTGCTTGCT | TTCTCTTTTCCTATCCTGAGTAGTGGT | GENOME_REGION | COSM99947 | hg19 | chr17 | 7576996 | 7577178 | 7577016 | 7577151 | 1 | Pool1 |
| CHP2_TP53_7 | CGCTTCTTGTCCTGCTTGCT | TTCTCTTTTCCTATCCTGAGTAGTGGT | GENOME_REGION | COSM99950 | hg19 | chr17 | 7576996 | 7577178 | 7577016 | 7577151 | 1 | Pool1 |
| CHP2_TP53_7 | CGCTTCTTGTCCTGCTTGCT | TTCTCTTTTCCTATCCTGAGTAGTGGT | GENOME_REGION | COSM99952 | hg19 | chr17 | 7576996 | 7577178 | 7577016 | 7577151 | 1 | Pool1 |
| CHP2_TP53_8 | GGAAGGGGCTGAGGTCACT | CCCCTCCTCTGTTGCTGC | GENOME_REGION | COSM10770 | hg19 | chr17 | 7573905 | 7574053 | 7573924 | 7574035 | 1 | Pool1 |
| CHP2_TP53_8 | GGAAGGGGCTGAGGTCACT | CCCCTCCTCTGTTGCTGC | GENOME_REGION | COSM11071 | hg19 | chr17 | 7573905 | 7574053 | 7573924 | 7574035 | 1 | Pool1 |
| CHP2_TP53_8 | GGAAGGGGCTGAGGTCACT | CCCCTCCTCTGTTGCTGC | GENOME_REGION | COSM11073 | hg19 | chr17 | 7573905 | 7574053 | 7573924 | 7574035 | 1 | Pool1 |
| CHP2_TP53_8 | GGAAGGGGCTGAGGTCACT | CCCCTCCTCTGTTGCTGC | GENOME_REGION | COSM11078 | hg19 | chr17 | 7573905 | 7574053 | 7573924 | 7574035 | 1 | Pool1 |
| CHP2_TP53_8 | GGAAGGGGCTGAGGTCACT | CCCCTCCTCTGTTGCTGC | GENOME_REGION | COSM11286 | hg19 | chr17 | 7573905 | 7574053 | 7573924 | 7574035 | 1 | Pool1 |
| CHP2_TP53_8 | GGAAGGGGCTGAGGTCACT | CCCCTCCTCTGTTGCTGC | GENOME_REGION | COSM11291 | hg19 | chr17 | 7573905 | 7574053 | 7573924 | 7574035 | 1 | Pool1 |
| CHP2_TP53_8 | GGAAGGGGCTGAGGTCACT | CCCCTCCTCTGTTGCTGC | GENOME_REGION | COSM11411 | hg19 | chr17 | 7573905 | 7574053 | 7573924 | 7574035 | 1 | Pool1 |
| CHP2_TP53_8 | GGAAGGGGCTGAGGTCACT | CCCCTCCTCTGTTGCTGC | GENOME_REGION | COSM11514 | hg19 | chr17 | 7573905 | 7574053 | 7573924 | 7574035 | 1 | Pool1 |
| CHP2_TP53_8 | GGAAGGGGCTGAGGTCACT | CCCCTCCTCTGTTGCTGC | GENOME_REGION | COSM18597 | hg19 | chr17 | 7573905 | 7574053 | 7573924 | 7574035 | 1 | Pool1 |
| CHP2_TP53_8 | GGAAGGGGCTGAGGTCACT | CCCCTCCTCTGTTGCTGC | GENOME_REGION | COSM43795 | hg19 | chr17 | 7573905 | 7574053 | 7573924 | 7574035 | 1 | Pool1 |
| CHP2_TP53_8 | GGAAGGGGCTGAGGTCACT | CCCCTCCTCTGTTGCTGC | GENOME_REGION | COSM43882 | hg19 | chr17 | 7573905 | 7574053 | 7573924 | 7574035 | 1 | Pool1 |
| CHP2_TP53_8 | GGAAGGGGCTGAGGTCACT | CCCCTCCTCTGTTGCTGC | GENOME_REGION | COSM44070 | hg19 | chr17 | 7573905 | 7574053 | 7573924 | 7574035 | 1 | Pool1 |
| CHP2_TP53_8 | GGAAGGGGCTGAGGTCACT | CCCCTCCTCTGTTGCTGC | GENOME_REGION | COSM44830 | hg19 | chr17 | 7573905 | 7574053 | 7573924 | 7574035 | 1 | Pool1 |
| CHP2_TP53_8 | GGAAGGGGCTGAGGTCACT | CCCCTCCTCTGTTGCTGC | GENOME_REGION | COSM44832 | hg19 | chr17 | 7573905 | 7574053 | 7573924 | 7574035 | 1 | Pool1 |
| CHP2_TP53_8 | GGAAGGGGCTGAGGTCACT | CCCCTCCTCTGTTGCTGC | GENOME_REGION | COSM44879 | hg19 | chr17 | 7573905 | 7574053 | 7573924 | 7574035 | 1 | Pool1 |
| CHP2_TP53_8 | GGAAGGGGCTGAGGTCACT | CCCCTCCTCTGTTGCTGC | GENOME_REGION | COSM45276 | hg19 | chr17 | 7573905 | 7574053 | 7573924 | 7574035 | 1 | Pool1 |
| CHP2_TP53_8 | GGAAGGGGCTGAGGTCACT | CCCCTCCTCTGTTGCTGC | GENOME_REGION | COSM45278 | hg19 | chr17 | 7573905 | 7574053 | 7573924 | 7574035 | 1 | Pool1 |
| CHP2_TP53_8 | GGAAGGGGCTGAGGTCACT | CCCCTCCTCTGTTGCTGC | GENOME_REGION | COSM45536 | hg19 | chr17 | 7573905 | 7574053 | 7573924 | 7574035 | 1 | Pool1 |
| CHP2_TP53_8 | GGAAGGGGCTGAGGTCACT | CCCCTCCTCTGTTGCTGC | GENOME_REGION | COSM45639 | hg19 | chr17 | 7573905 | 7574053 | 7573924 | 7574035 | 1 | Pool1 |
| CHP2_TP53_8 | GGAAGGGGCTGAGGTCACT | CCCCTCCTCTGTTGCTGC | GENOME_REGION | COSM46015 | hg19 | chr17 | 7573905 | 7574053 | 7573924 | 7574035 | 1 | Pool1 |
| CHP2_TP53_8 | GGAAGGGGCTGAGGTCACT | CCCCTCCTCTGTTGCTGC | GENOME_REGION | COSM46348 | hg19 | chr17 | 7573905 | 7574053 | 7573924 | 7574035 | 1 | Pool1 |
| CHP2_TP53_8 | GGAAGGGGCTGAGGTCACT | CCCCTCCTCTGTTGCTGC | GENOME_REGION | COSM99721 | hg19 | chr17 | 7573905 | 7574053 | 7573924 | 7574035 | 1 | Pool1 |
| Hotspot_1.17678 | CAAATGCTGAAAGCTGTACCATACC | AAAAGACTCGGATGATGTACCTATGG | GENOME_REGION | P117_NRAS | hg19 | chr1 | 115252166 | 1,2E+08 | 115252191 | 115252305 | 1 | Pool1 |
| Hotspot_1.17678 | CAAATGCTGAAAGCTGTACCATACC | AAAAGACTCGGATGATGTACCTATGG | GENOME_REGION | pA146T_NRAS | hg19 | chr1 | 115252166 | 1,2E+08 | 115252191 | 115252305 | 1 | Pool1 |
| Hotspot_2.61436 | TGAGGCAGTCTTTACTCACCTG | TTCCTTTCTTCCCAGAGACATTGC | GENOME_REGION | G1269A_ALK | hg19 | chr2 | 29432633 | 2,9E+07 | 29432655 | 29432735 | 1 | Pool1 |
| Hotspot_3.51716 | GCAAAGACTGGTTCTCACTCACC | ACATCCCTCTCTGCTCTGCA | GENOME_REGION | COSM28054 | hg19 | chr2 | 29443550 | 2,9E+07 | 29443573 | 29443702 | 1 | Pool1 |
| Hotspot_3.51716 | GCAAAGACTGGTTCTCACTCACC | ACATCCCTCTCTGCTCTGCA | GENOME_REGION | COSM28055 | hg19 | chr2 | 29443550 | 2,9E+07 | 29443573 | 29443702 | 1 | Pool1 |
| Hotspot_3.51716 | GCAAAGACTGGTTCTCACTCACC | ACATCCCTCTCTGCTCTGCA | GENOME_REGION | COSM28057 | hg19 | chr2 | 29443550 | 2,9E+07 | 29443573 | 29443702 | 1 | Pool1 |
| Hotspot_3.51716 | GCAAAGACTGGTTCTCACTCACC | ACATCCCTCTCTGCTCTGCA | GENOME_REGION | COSM28059 | hg19 | chr2 | 29443550 | 2,9E+07 | 29443573 | 29443702 | 1 | Pool1 |
| Hotspot_3.51716 | GCAAAGACTGGTTCTCACTCACC | ACATCCCTCTCTGCTCTGCA | GENOME_REGION | COSM28061 | hg19 | chr2 | 29443550 | 2,9E+07 | 29443573 | 29443702 | 1 | Pool1 |
| Hotspot_3.51716 | GCAAAGACTGGTTCTCACTCACC | ACATCCCTCTCTGCTCTGCA | GENOME_REGION | COSM28491 | hg19 | chr2 | 29443550 | 2,9E+07 | 29443573 | 29443702 | 1 | Pool1 |
| Hotspot_3.51716 | GCAAAGACTGGTTCTCACTCACC | ACATCCCTCTCTGCTCTGCA | GENOME_REGION | COSM99137 | hg19 | chr2 | 29443550 | 2,9E+07 | 29443573 | 29443702 | 1 | Pool1 |
| Hotspot_3.51716 | GCAAAGACTGGTTCTCACTCACC | ACATCCCTCTCTGCTCTGCA | GENOME_REGION | D1203N_ALK | hg19 | chr2 | 29443550 | 2,9E+07 | 29443573 | 29443702 | 1 | Pool1 |
| Hotspot_3.51716 | GCAAAGACTGGTTCTCACTCACC | ACATCCCTCTCTGCTCTGCA | GENOME_REGION | p.F1174C_ALK | hg19 | chr2 | 29443550 | 2,9E+07 | 29443573 | 29443702 | 1 | Pool1 |
| Hotspot_3.51716 | GCAAAGACTGGTTCTCACTCACC | ACATCCCTCTCTGCTCTGCA | GENOME_REGION | p.G1202R_ALK | hg19 | chr2 | 29443550 | 2,9E+07 | 29443573 | 29443702 | 1 | Pool1 |
| Hotspot_3.51716 | GCAAAGACTGGTTCTCACTCACC | ACATCCCTCTCTGCTCTGCA | GENOME_REGION | p.L1196M_ALK | hg19 | chr2 | 29443550 | 2,9E+07 | 29443573 | 29443702 | 1 | Pool1 |
| Hotspot_3.51716 | GCAAAGACTGGTTCTCACTCACC | ACATCCCTCTCTGCTCTGCA | GENOME_REGION | p.S1206Y_ALK | hg19 | chr2 | 29443550 | 2,9E+07 | 29443573 | 29443702 | 1 | Pool1 |
| ON_ALK_0 | AGGGTGTCTCTCTGTGGCTTTA | GACTCTGTAGGCTGCAGTTCTC | GENOME_REGION | COSM98478 | hg19 | chr2 | 29445187 | 2,9E+07 | 29445209 | 29445320 | 1 | Pool1 |
| ON_ALK_0 | AGGGTGTCTCTCTGTGGCTTTA | GACTCTGTAGGCTGCAGTTCTC | GENOME_REGION | p.C1156Y_ALK | hg19 | chr2 | 29445187 | 2,9E+07 | 29445209 | 29445320 | 1 | Pool1 |
| ON_ALK_0 | AGGGTGTCTCTCTGTGGCTTTA | GACTCTGTAGGCTGCAGTTCTC | GENOME_REGION | p.L1152R_ALK | hg19 | chr2 | 29445187 | 2,9E+07 | 29445209 | 29445320 | 1 | Pool1 |
| ON_ALK_0 | AGGGTGTCTCTCTGTGGCTTTA | GACTCTGTAGGCTGCAGTTCTC | GENOME_REGION | p.T1151_L1152insT_ALK | hg19 | chr2 | 29445187 | 2,9E+07 | 29445209 | 29445320 | 1 | Pool1 |
| ON_DDR2_1 | CAGTGAAAACTGTGGCAAGAACC | AGGCTCCATCCCCTTCTTCTG | GENOME_REGION | CHR1_162724353 | hg19 | chr1 | 162724267 | 1,6E+08 | 162724290 | 162724421 | 1 | Pool1 |
| ON_DDR2_2 | GTTTCTGCAGATTGACTTGCACA | CTTCTTCCTACCTGTTTCCCATGAC | GENOME_REGION | COSM12821 | hg19 | chr1 | 162724483 | 1,6E+08 | 162724506 | 162724631 | 1 | Pool1 |
| ON_DDR2_2 | GTTTCTGCAGATTGACTTGCACA | CTTCTTCCTACCTGTTTCCCATGAC | GENOME_REGION | COSM173712 | hg19 | chr1 | 162724483 | 1,6E+08 | 162724506 | 162724631 | 1 | Pool1 |
| ON_DDR2_3 | CCTGAGCAGCATGACAGAAG | AACATGATCTCAATGTAGCCATTGGT | GENOME_REGION | COSM140388 | hg19 | chr1 | 162729577 | 1,6E+08 | 162729597 | 162729706 | 1 | Pool1 |
| ON_DDR2_4 | CTCGGAGGATGCTGGATGATG | AGTTTTCGTATCAGCCTGGATGG | GENOME_REGION | COSM48314 | hg19 | chr1 | 162740096 | 1,6E+08 | 162740117 | 162740247 | 1 | Pool1 |
| ON_DDR2_5 | CTCCAAAGACACTCCACGGAA | GACGGCAGGCACTGAGTA | GENOME_REGION | COSM140389 | hg19 | chr1 | 162741765 | 1,6E+08 | 162741786 | 162741920 | 1 | Pool1 |
| ON_DDR2_5 | CTCCAAAGACACTCCACGGAA | GACGGCAGGCACTGAGTA | GENOME_REGION | COSM140390 | hg19 | chr1 | 162741765 | 1,6E+08 | 162741786 | 162741920 | 1 | Pool1 |
| ON_DDR2_6 | CAGCAAGAGTACTGAGACATCTTCA | TCTGCTCGGAGCATTTTCACA | GENOME_REGION | COSM94126 | hg19 | chr1 | 162743194 | 1,6E+08 | 162743219 | 162743347 | 1 | Pool1 |
| ON_DDR2_7 | TGTCTGTATCCTCCCAAGGAATGA | CTCGTGGCGGGAAAGAAAC | GENOME_REGION | CHR1_162745511 | hg19 | chr1 | 162745424 | 1,6E+08 | 162745448 | 162745576 | 1 | Pool1 |
| ON_DDR2_8 | GGTGTTGTTGTGCACAGGTTAT | CTGTTCATCTGACAGCTGGGAA | GENOME_REGION | CHR1_162748394 | hg19 | chr1 | 162748315 | 1,6E+08 | 162748337 | 162748452 | 1 | Pool1 |
| ON_EGFR_2A | TCAGGAAACAAAAATTTGTGCTATGCAA | GGACCCATTAGAACCAACTCCAT | GENOME_REGION | COSM236670 | hg19 | chr7 | 55227923 | 5,5E+07 | 55227951 | 55228057 | 1 | Pool1 |
| ON_MAP2K1_1 | GCTGGAGGAGCTAGAGCTTGAT | GGCTTGTGGGAGACCTTGAAC | GENOME_REGION | COSM236154 | hg19 | chr15 | 66727392 | 6,7E+07 | 66727414 | 66727529 | 1 | Pool1 |
